# Oral Health Research in the WHO African Region between 2011 and 2022: A Scoping Review

**DOI:** 10.1177/00220345241272024

**Published:** 2024-10-29

**Authors:** T.F. Labarca, D. Ortuño, L. Neira, G. Andrade, F.J. Bravo, C.R. Cantarutti, M. Dallaserra, A. Gatarayiha, J. Karajgikar, R.J. Kulchar, X. Liu, C.C. Martins-Pfeifer, N. Olivares, L. Pilcher, S. Pahlke, C. Pirela, J.M. Sanchez, A. Song, O. Urquhart, J.P. Vargas, C. Véliz, F. Verdugo-Paiva, P. Vergara, V. Zaffiri, J. Zuñiga, Y. Makino, M. Glick, A. Carrasco-Labra

**Affiliations:** 1 Faculty of Dentistry, Universidad de los Andes, Monseñor Álvaro del Portillo, Santiago, Las Condes, Región Metropolitana, Chile; 2 School of Dentistry, Faculty of Medicine, Pontificia Universidad Católica de Chile, Vicuña Mackenna, Santiago, Región Metropolitana, Chile; 3 Faculty of Dentistry, Department of Oral and Maxillofacial Surgery, Universidad de Chile, Olivos, Independencia, Región Metropolitana, Chile; 4 School of Dentistry, College of Medicine and Health Sciences, University of Rwanda, Kigali, Rwanda; 5 Applied Data Science Librarian, Research Data and Digital Scholarship, University of Pennsylvania Libraries, Philadelphia, PA, USA; 6 Center for Integrative Global Oral Health, School of Dental Medicine, University of Pennsylvania, Philadelphia, PA, USA; 7 Research Data and Digital Scholarship, Graduate School of Education, University of Pennsylvania, Philadelphia, PA, USA; 8 Department of Pediatric Dentistry, School of Dentistry, Universidade Federal de Minas Gerais, Pampulha, Belo Horizonte, Brazil; 9 Quality Initiatives, American Academy of Pediatrics, Itasca, IL, USA; 10 Clinical Affairs and Practice Guidelines, Infectious Diseases Society of America, Arlington, VA, USA; 11 Faculty of Dentistry, Department of Conservative Dentistry, Universidad de Chile, Independencia, Región Metropolitana, Chile; 12 Mean Data Corporation, FL, USA; 13 Epistemonikos Foundation, Mariano Sanchez Fontecilla, Santiago, Chile; 14 Orofacial Pain & TMD Program, Facultad de Odontología, Universidad Andrés Bello, Santiago, Chile; 15 Noncommunicable Diseases Management team, WHO Regional Office for Africa, Brazzaville, Congo

**Keywords:** research design, epidemiology, public health, health services research, policy, evidence-based dentistry

## Abstract

The status of oral health research in the World Health Organization (WHO) African region is unclear, yet the need for such information is central to moving an oral health agenda forward. Such an agenda is essential for effectively translating research into actionable practices and supporting regional strategies. The aim of this scoping review was to provide data on the scope and output of oral health research in the WHO African region to be used as a starting point for establishing a research agenda that can affect oral health in the region. We conducted a systematic search in PubMed; EMBASE; Epistemonikos; Scopus; the International Association for Dental, Oral, and Craniofacial Research General and Regional Sessions; ProQUEST; PROSPERO; and African regional databases such as Regional African Index Medicus and the African Journal Online. We included primary and secondary studies published in English, French, or Portuguese between January 1, 2011, and December 31, 2022, addressing oral health–related research having individuals, groups, or populations as units of analysis. These reports either addressed a topic relevant to the WHO African region assessed using the title and study objective or were conducted in a country in the region. We excluded in vitro and in vivo studies focusing on cells, biomarkers, or animals. We assessed 24,014 records, and 1,379 proved eligible. Our findings indicate a preference for particular research designs less suitable for evidence-informed practice guidelines and oral policies, a limited scope of oral health research topics, and important regional differences in research capacity. Furthermore, publications by researchers in the WHO African region tend to be published in journals with a limited readership. A discussion of our findings among oral health researchers at academic institutions in the WHO African region on how to create within- and across-country collaborations could potentially improve both health and oral health in the region.

## Introduction

Oral diseases are among the most common noncommunicable diseases (NCDs), with impact at social, economic, and health system levels. An estimated 3.5 billion individuals have active oral disease, giving rise to pain, disfigurement, social seclusion, emotional anguish, and, in severe cases, even death (Global Burden of Disease 2019 [GBD 2019] 2020). The burden of oral diseases significantly overshadows the collective prevalence of all 5 major NCDs—mental disorders, cardiovascular disease, diabetes mellitus, chronic respiratory diseases, and cancers—underscoring the critical importance of addressing oral health globally (Benzian et al. 2023; World Health Organization 2023b).

The African region is one of the World Health Organization’s (WHO’s) 6 regions worldwide and comprises 47 Member States. Due to various socioeconomic, cultural, and environmental factors (NCD Countdown 2030 Collaborators 2020), oral health remains a significant public health concern in the region. Around 44% of the population was estimated to suffer from oral diseases in 2019, with the most significant increase in major oral disease cases in the previous 30 y among all WHO regions (World Health Organization 2023b). The most prevalent and severe oral diseases include dental caries, periodontal diseases, oral and oropharyngeal cancer, orofacial trauma, oral manifestations of HIV infection, congenital disabilities, and noma. The burden of these oral diseases creates notable disparities, impacting marginalized communities throughout their life span (Petersen et al. 2005).

Despite efforts to enhance the region’s oral health status, the prevailing actions in recent decades have been characterized by fragmented and specialized approaches rather than implementing integrated and economically efficient strategies. The WHO Regional Oral Health Strategy 2016–2025, addressing oral diseases as part of NCDs, was published in 2016 (Regional Office for Africa 2022). This publication puts forth strategies to aid Member States in prioritizing oral health by incorporating oral diseases into the prevention and management of NCDs within the framework of Universal Health Coverage (UHC) (Regional Committee for Africa et al. 2016). One strategic objective of the Regional Oral Health strategy is “to improve integrated surveillance of oral diseases, monitoring, and evaluation of programmes and research” (Regional Office for Africa 2022). Beyond the scope of regional strategies, the global political guidance for oral health also underscored the significance of oral health research, particularly emphasizing the requirement for contextually relevant and constantly updated research that centers on the public health side of oral health (Seventy-fifth World Health Assembly and World Health Organization 2022; World Health Organization 2023a).

Although the number of publications by countries in the WHO African region has recently increased (Kanoute et al. 2012), authorities still report a lack of evidence for decision-making in oral health strategies (Etiaba et al. 2015). The aim of this scoping review was to determine the oral health research scope and output in the WHO African region between January 2011 and December 2022.

## Methods

The reporting of this scoping review follows the Preferred Reporting Items for Systematic Reviews and Meta-Analyses Extension for Scoping Reviews (PRISMA-ScR) checklist (Appendix Table 1) (Tricco et al. 2018). The protocol was registered on the Open Science Framework (Labarca et al. 2023), and the search strategy was registered in the CABI Digital Library (Ortuño et al. 2023).

### Eligibility Criteria

We included primary and secondary studies published in English, French, or Portuguese between January 1, 2011, and December 31, 2022, addressing oral health–related research having individuals, groups, or populations as their unit of analysis. We excluded in vitro and in vivo studies focusing on cells, biomarkers, or animals. Eligible studies included clinical trials, cohort studies, case-control studies, cross-sectional studies, ecological studies, systematic reviews (with or without meta-analysis), policy briefs, and evidence-informed guidelines. These reports either addressed a topic relevant to the WHO African region assessed using the title and study objective or were conducted in a country in the region.

### Search Strategy and Information Sources

We conducted a systematic search in the following electronic databases: PubMed, EMBASE, Epistemonikos (curating primary and secondary studies from the Cochrane Database of Systematic Reviews [CDSR], PubMed, EMBASE, CINAHL [Cumulative Index to Nursing and Allied Health Literature], PsycINFO, LILACS, Database of Abstracts of Reviews of Effects [DARE], the Campbell Collaboration online library, the JBI Database of Systematic Reviews and Implementation Reports, and the EPPI-Centre Evidence Library), Scopus, and African regional databases such as Regional African Index Medicus and the African Journal Online (Appendix Table 2). We adapted the PubMed search strategy to the syntax and thesauri of other databases. In addition, to identify articles that might have been missed in the electronic searches, we conducted cross-citation searches in Google Scholar and Microsoft Academic, using each included study as the index reference. We reviewed the reference list of each included study and the abstracts of the International Association for Dental, Oral, and Craniofacial Research (IADR) General and Regional Sessions. We searched dissertations and theses in the ProQUEST database and for ongoing or recently completed systematic reviews in PROSPERO. The retrieved records were uploaded into Covidence to ensure an efficient workflow and remove overlapping records across databases.

### Selection of Studies

We screened the retrieved records using title and abstract and, in the second stage, using full-text reports independently and in duplicate by 15 previously calibrated reviewers (κ = 0.91). We solved disagreements by consensus. If differences persisted, a third external reviewer acted as a referee. If information was missing to ascertain eligibility, we contacted the study’s corresponding author via e-mail.

### Data Extraction and Analysis

Ten reviewers, independently and in duplicate, extracted data from the included studies using a piloted data extraction template. Reviewers were trained to ensure consistency in data extraction (κ = 0.81). Disagreements in the extracted data were solved by consensus or adjudication by an additional third reviewer when consensus was elusive. We extracted variables associated with the journal of publication (name, country, editorial focus on African-oriented research [determined by the journal description and editorial scope reported in the journal’s webpage, indices, and aggregators]), study authors (number of authors, country of the study, affiliations, collaborations within and outside the WHO African region, for African authors their position in the article and corresponding assignment), primary and secondary studies (title, year of publication, keywords, and number of citations in Google Scholar, funding source, declaration of conflict of interest). In addition, we classified the studies according to 4 taxonomies:

1. Research type as defined by WHO in their systematic approach for undertaking a research priority–setting exercise (World Health Organization 2020):
a. Problem: research to measure the size of the health problem through epidemiology, estimating the disease burden, and other forms of data collection
b. Cause: research to understand the causal agents, risk factors, and determinants of the health issue (this research may include, for instance, a study of infection cycles, vectors, the role of socioeconomic factors, environment, diet, and the interaction of multiple factors)
c. Solution: research to develop new interventions, including therapeutics, devices, procedures, policy interventions, public health campaigns, and so on
d. Implementation: research to translate new interventions into policy and practice and understand the barriers to delivering known interventions
e. Evaluation: research to monitor and evaluate the effectiveness or health impact of an intervention or program
2. Research designs: clinical trials, cohort studies, case-control studies, cross-sectional studies, ecological studies, systematic reviews (with or without meta-analysis), policy briefs, and evidence-informed guidelines
3. Study disciplines as categorized by the American Dental Association (ADA): dental public health, general dentistry, pediatric dentistry, oral and maxillofacial surgery, oral and maxillofacial pathology, oral medicine, periodontics, orthodontics and dentofacial orthopedics, oral and maxillofacial radiology, endodontics, prosthodontics, basic science–related research (translational research), orofacial pain, and dental anesthesiology
4. Oral health topics and subcategories. In an iterative process, we extracted the study’s primary disease, condition, or specific research topic and subcategories that further described the topic by reviewing the title, keywords, and full-text article.

We used descriptive statistics (frequencies and percentages) to summarize quantitative data. A country-level impact factor was determined by calculating the ratio of the total number of citations received by records from a particular country to the overall number of studies conducted within that country. Each record covering diverse topics was individually classified for each relevant theme. This approach was implemented to maintain transparency and methodological clarity in handling multifaceted records, allowing for a nuanced analysis of each record and topic. In addition, we conducted a collaboration mapping analysis. We created a database including all relevant study citation data (title, abstract, year of publication), authors’ names, academic or research institution (affiliation), city, and country of the institution. These data were obtained using publicly available Application Programming Interface (API) from the US National Library of Medicine and Elsevier. We retrieved records based on a query including eligible studies’ PubMed Identifier (PMID) and other database-specific identifiers using Python (Van Rossum and Drake 2009). The list of articles was exported using a .csv file. A new script was created using C# to read the .csv file and prepare the data set for analysis. We conducted an iterative deserialization process to transform the original output generated from the APIs into human-readable text and identify different source data formats. We implemented exception functions to standardize the data set and stored it in a Structure Query Language (SQL) relational database for transactional purposes. The data set was used to create an interactive data visualization tool in the form of a collaboration map, using Shiny, a web application framework for R developed by RStudio (Chang et al. 2023). Additional information regarding the collaboration map is available on GitHub (Liu et al. 2023).

## Results

We identified 24,014 records across databases. After removing duplicates, 18,992 remained for title and abstract screening. Out of 1,720 records selected for full-text screening, 1,379 proved eligible (Appendix Fig. 1 and Appendix Table 3). The average number of articles published per year between 2011 and 2022 was 115 ± 11.7 (SD), with a trend line that suggests no significant fluctuations during the past decade (Appendix Fig. 2).

### Type of Research, Designs, and Study Discipline

**Figure 1.**
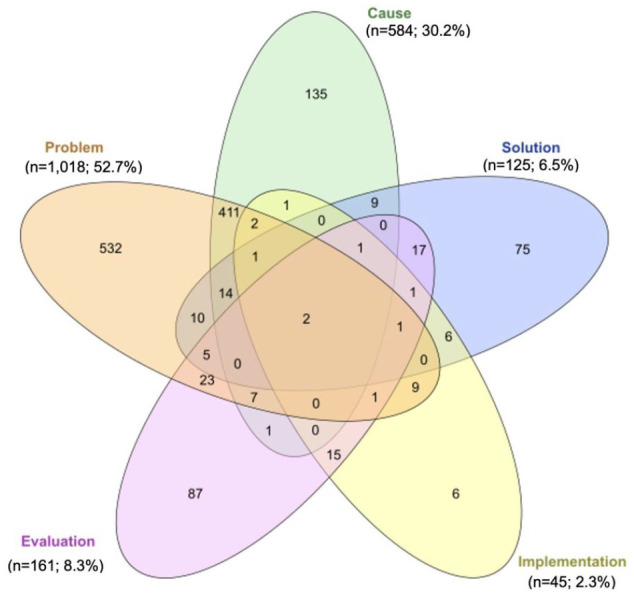
Venn diagram: Distribution of oral health publications in the World Health Organization (WHO) African region countries between 2011 and 2022 according to research type (*n* = 1,933)*. The 5 categories of research type were defined as follows: Problem—research to measure the size of the health problem through epidemiology, estimating the burden of disease and other forms of data collection. Cause—research to understand the causal agents, risk factors, and determinants of the health issue (this research may include, for instance, a study of infection cycles, vectors, the role of socioeconomic factors, environment, diet, and the interaction of multiple factors). Solution—research to develop new interventions, including therapeutics, devices, and procedures and also policy interventions, public health campaigns, and so on. Implementation—research to translate new interventions into policy and practice and understanding the barriers to delivering known interventions. Evaluation—research to monitor and evaluate the effectiveness or health impact of an intervention or program. (A systematic approach for undertaking a research priority-setting exercise. Guidance for WHO staff. Geneva: World Health Organization; 2020. License: CC BY-NC-SA 3.0 IGO). *A single article may have addressed more than one research type category.

**Figure 2.**
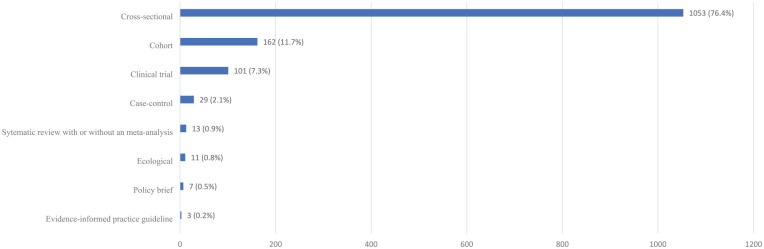
Study design distribution of oral health publications conducted in the World Health Organization African region countries between 2011 and 2022 (*n* = 1,379).

According to the WHO research type classification (*n* = 1,933 [more than 1 research type category was addressed in a single article]), more than half of the entire oral health research output of the region (*n* = 1,018, 52.7%) focused on measuring the magnitude of a health problem, estimating the burden of disease (problem). One-third (*n* = 584, 30.2%) of the research output aimed to identify causal agents, risk factors, and determinants of health (cause). Research addressing solution, implementation, and evaluation categories combined did not account for more than 17.1% (*n* = 331) of the total research output (Fig. 1). When classifying articles according to their design, more than three-quarters were cross-sectional studies (*n* = 1,053, 76.4%), while cohort, case-control, and clinical trials accounted for 21.1% (*n* = 292) of the total research output. No more than 1.4% of all articles focused on evidence synthesis (systematic reviews with or without meta-analysis and policy briefs). Implementation tools like evidence-informed practice guidelines were infrequently published (*n* = 3, 0.2%) (Fig. 2). Across disciplines, more than half of the entire research output concerned dental public health (*n* = 503, 21.5%), general dentistry (*n* = 473, 20.2%), and pediatric dentistry (*n* = 280, 12.0%) (Appendix Fig. 3).

### Research Efforts (Number of Investigators) per Oral Health Topic

**Figure 3.**
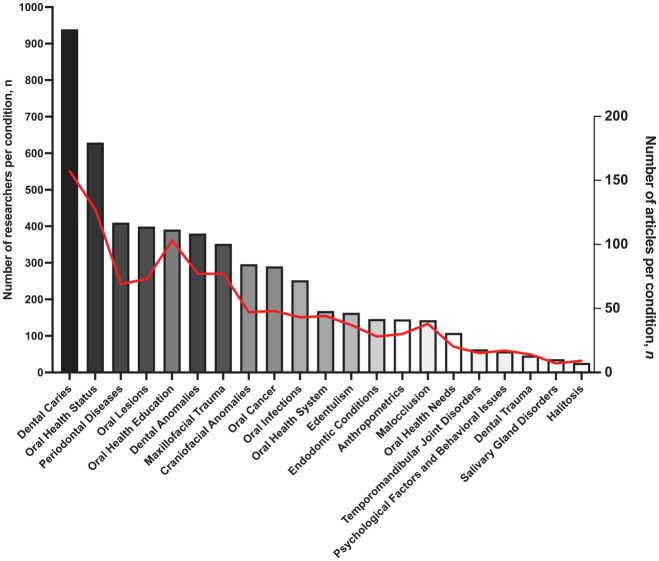
Number of researchers (*n* = 5,440) who were authors in oral health–related studies in the World Health Organization (WHO) African region countries between 2011 and 2022, according to disease, condition, or topic addressed by the study and the number of articles produced (*n* = 1,080). The discrepancy with the total number of included studies (*n* = 1,379) is due to the availability of studies’ meta-data from the US National Library of Medicine and Elsevier’s Application Programming Interfaces to retrieve authors’ affiliations. The bars indicate the number of researchers studying a disease, condition, or topic. The red line indicates the number of articles per disease, condition, or topic.

The classification of articles per oral health topic across investigators (*n* = 5,440) indicated that approximately half (*n* = 2,768, 50.9%) of the research efforts focused on dental caries (939 investigators, 17.3%), oral health status assessment (629 investigators, 11.6%), periodontal disease (410 investigators, 7.5%), oral mucosa lesions (399 investigators, 7.3%), and oral health education (391 investigators, 7.2%). Noma was addressed in 6 studies in our sample (Fig. 3 and Appendix Table 4). In addition, we identified 294 unique articles (27.2%) that focused on public health–related topics (e.g., oral health status, oral health education, oral health systems, and oral health needs) conducted by 1,296 investigators. For this topic, most research efforts were dedicated to the following subcategories: assessment of oral health situation (epidemiological studies) (524 investigators, 40.4%) and health care professionals’ education (173 investigators, 13.3%). Relevant areas to inform policy and system-level interventions, including community outreach and promotion (84 investigators, 6.5%), oral health services (79 investigators, 6.1%), oral health care access (58 investigators, 4.5%), and workforce studies (26 investigators, 2.0%), did not individually reach beyond 6.5% of total research efforts (Appendix Fig. 4).

### Journals Publishing African Oral Health Research

Articles about oral health research in the WHO African region were published in African and non-African dental journals. One-third of the articles (*n* = 497, 36.04%) were published in 75 journals with an editorial focus on Africa. Of the 20 journals with the most publications, 12 (60%) had an African editorial scope. The journals with the most publications were *BMC Oral Health*, the *Nigerian Journal of Clinical Practice*, and the *Pan African Medical Journal* (Appendix Table 5).

### Research Efforts per Country and across Collaborations

Nigeria and South Africa had the highest participation in oral health research studies (i.e., where the study was conducted) in the WHO African region (*n* = 912, 55.3%). The individual research output contribution of the remaining countries ranged between 1.1% and 4.6%. The most prolific research partnerships included authors from multiple countries in the region (*n* = 1,195, 57.7%), while national collaborations represented 18.2% (*n* = 2,070) of the total partnerships. A total of 73 articles addressed research topics pertaining to the WHO African region and were neither published by researchers affiliated with any entity within the WHO African region nor involved collaboration with African researchers (Table, Collaboration map [https://tmdapp.shinyapps.io/dental-map-affiliations/]).

**Table.** Summary of Oral Health Research Produced in the WHO African Region Countries between 2011 and 2022.

Country in WHO African Region	Participation in Research Studies, *n* (%)	Citations, *n*	Citations/Number of Articles	Partnership^a^			No Author from WHO African Region,^b^ *n*
				National	WHO African Region	Other	
Nigeria	760 (46.16)	12,738	16.76	227	518	77	3
South Africa	152 (9.15)	2,472	16.26	49	102	78	1
United Republic of Tanzania	79 (4.63)	1,714	21.69	9	63	33	2
Ethiopia	74 (4.39)	1,627	21.98	5	60	24	3
Uganda	69 (4.09)	1,537	22.27	9	44	32	6
Kenya	59 (3.48)	231	3.91	7	42	24	2
Senegal	50 (2.93)	1,032	20.64	11	31	18	2
Ghana	40 (2.32)	577	14.42	6	26	19	3
Cameroon	30 (1.71)	599	19.96	4	20	9	2
Cote d’ Ivoire	28 (1.59)	239	8.53	5	19	12	1
Rwanda	28 (1.59)	338	12.07	1	22	12	1
Burkina Faso	25 (1.40)	209	8.36	2	19	12	1
Mali	20 (1.10)	204	10.20	0	16	7	2
Others^c^	253 (15.49)	4,756	18.79	43	213	140	44
Total	1,665 (100)^d^	28,273	16.98	378	1,195	497	73

WHO, World Health Organization.

a Partnerships were classified according to the nature of the collaboration between entities. 1) National: This refers to a collaboration between 2 different entities within the same country. 2) WHO African region: This classification represents collaborations between entities from different countries within the WHO African region. 3) Other: This classification encompasses collaborations that extend beyond the WHO African region (global).

b Articles published by authors not affiliated with any entity within the WHO African region or involving collaboration with a WHO African author but addressed an oral health research topic pertaining to the WHO African region.

c This category includes countries with a publication output below 1%.

d There were 31 articles reporting research conducted in more than 1 WHO African country. Each study involving more than 1 country was considered in the individual count separately, explaining the difference between the total number of articles identified in the search (*n* = 1,379) and the number of per-country participations in research studies.

## Discussion

This study identified the oral health research scope and output in the WHO African region between 2011 and 2022. We estimated that nearly 50% of scientific production originated from Nigeria. Although having a very high rate of production of research publications, Nigeria also has, by far, the most dental schools of any country in the WHO African region. In addition, collaboration networks among authors and institutions within the region and globally were predominantly linked to Nigeria and South Africa, which could be explained, in part, by the number of dental schools in these countries. A significant portion of publications are found in journals without a specific focus on the WHO African region. Most studies were cross-sectional and focused primarily on dental public health, general dentistry, and pediatric dentistry. Most publications from the region centered on the most prevalent oral diseases, including dental caries, periodontal diseases, other oral cavity lesions, and dental and oral cavity anomalies. On the other hand, noma, a disease associated with high mortality and currently a public health priority in several countries in the region (Non-Communicable Disease Cluster et al. 2016), was only addressed in 6 studies in our sample.

The large disparity in dental research output among countries in the region was consistent with a previous study that indicated that among all African countries, South Africa, Egypt, and Nigeria accounted for 60% of the articles indexed in PubMed between 1996 and 2005 (Uthman and Uthman 2007). A difference in our study compared to theirs is that we considered the regional definition by WHO, which excludes Egypt. A more recent overview evaluated oral health publications across different African subregions between 2005 and 2010 and concluded that of 935 publications, encompassing all types of articles except editorials and letters, around 68% originated from Nigeria and South Africa (Kanoute et al. 2012). The lack of scientific production and dissemination in low-income African countries is troubling, considering their high disease burdens, pronounced health inequities, and pressing need for scientific data (Lee and Divaris 2014; El Tantawi et al. 2023). Research published in European or North American journals often excludes topics relevant to low-income African countries (Horton 2000).

When considering the research type according to WHO’s research prioritization criteria, approximately 53% were related to the “problem” category and 30% to the “cause” category. In our study, the number of publications addressing “solution,” “evaluation,” and “implementation” categories was scant, highlighting the need to redirect efforts toward generating evidence on the effectiveness, safety, and impact of oral health interventions. Our findings differ from a previous WHO report on general health research globally, which showed that out of 2,145 publications between 2002 and 2017, the highest proportion of the 5 categories (cause, problem, solution, evaluation, and implementation) corresponded to implementation (43%) (World Health Organization 2020). Previous studies have emphasized the need to incorporate aspects related to dissemination and implementation into research agendas in clinical, community, and policy environments in dentistry (Randall 2023).

In our review, approximately 76% of the included articles were cross-sectional, aligning with research aimed at describing a “problem,” measuring health issues extent through epidemiology, estimating disease burden, and other data collection forms (World Health Organization 2020). A bibliometric study that analyzed articles published in PubMed-indexed journals between 2008 and 2018 in the WHO African region also identified a prevalence of cross-sectional designs in half of the articles (Bennani et al. 2021).

Our results also showed that oral health research in the region primarily originates from university-based dental schools. This poses a challenge for a region where 11 countries lack dental schools (Gallagher et al. 2023). While these countries might have medical or public health schools partially addressing oral health issues, the absence of dental schools can still pose a substantial challenge in fostering dedicated dental research (McCauley 2017). A recent study suggested that none of the 3,300 most-cited articles in dentistry originated from countries in the WHO Africa region (Daryakenari and Batooli 2022). The number of publications in African journals presents an opportunity to reach local audiences on regional interest topics. A factor contributing to the limited visibility of local publications is that these journals are not included in large databases like the US National Library of Medicine (MEDLINE) (Tillett 2005) and the region lacks a country- or regional-based knowledge translation platform to share these local publications (Regional Committee for Africa and Organization 2023). Local and global evidence are integral to the knowledge base informing oral health policies (Listl et al. 2023). The limited visibility problem is not solely related to the source of scientific information and its dissemination but also the ability to access such information. While Internet users in Africa have increased, the region still has the lowest Internet penetration rates globally, with highly unequal distribution between the WHO African region and northern African countries (Manyazewal et al. 2023).

One strength of this review was the implementation of a comprehensive search strategy across major health databases, including African regional databases such as Regional African Index Medicus and the African Journal Online. This approach allowed us to identify scientific articles published in journals from the WHO Africa region, not only in English but also in French and Portuguese, the working languages of the WHO Regional Committee in the African region. Additionally, including multilingual articles enhances the representation of diverse research outputs. Another strength was identifying countries with the highest production of scientific publications, pinpointing collaborations with authors outside the WHO Africa region, and identifying prevalent research topics. Our review also has some limitations. Age groups other than those typically classified under pediatric dentistry or special subpopulations were not specifically categorized. This issue may limit the generalizability of the findings to specific age ranges within populations. In addition, we cannot rule out the possible exclusion of nonindexed predatory journals known to impact the scientific production of the region. Although we have included scientific articles written in the working languages of the WHO Regional Committee in the African region, it is possible that this choice may have inadvertently omitted research from countries and journals utilizing languages other than the aforementioned. Another limitation of our study is the absence of information regarding the specific expertise of the authors of the reviewed papers. Although not a standard in scoping reviews (Munn et al. 2018), our review did not include an assessment of the methodological quality of the included studies, which may limit the ability to make further inferences about investigators’ training and research design competence.

The findings of this study highlight the necessity of strengthening oral health research in the WHO African region. It emphasizes the importance of establishing core evidence-producing hubs for decision-making originating from local universities and other regional institutions. Such an approach ensures that decisions remain rooted in local expertise, reducing reliance on external collaborations that may risk scientific colonization. Research hubs must be led and driven by African investigators. This is not a call for deprioritizing international partnerships but an opportunity for oral health research organizations in the WHO African region to define their destiny (Lakati and Masibo 2023). The recent WHO Oral Health Action Plan includes, as one of its strategic objectives, the continuous development and updating of context-specific research focusing on public health aspects related to oral health (World Health Organization 2023a). The lack of funding and infrastructure and insufficient collaboration among local researchers are possible explanations for our results (Kanoute et al. 2014). Opportunities exist in the region to enhance the capacity for oral health research. For example, “Research for Health: A Strategy for the African Region 2016–2025” exists, which supports Member States in fostering the development of a functional national health research system by establishing research for health governance, improving building and sustenance of human, physical, and institutional research capacities; strengthening production and use of research to enhance the performance of health systems; establishing sustainable research for health financing; and establishing mechanisms for tracking health research investments. In addition, a research network exists, such as the Consortium for Advanced Research Training in Africa (CARTA), a program designed to strengthen research infrastructure and capacity at African universities. CARTA aims to develop local research capacity to comprehend the determinants of population health and effectively intervene to enhance health outcomes (Ezeh et al. 2010). This places science in the service of public health functions (D’Souza et al. 2022).

The WHO political guidance document on oral health highlights the importance of research to create and continuously update a local body of research evidence to inform oral health policies in the WHO African region (Seventy-fifth World Health Assembly and World Health Organization 2022). This essential body of evidence is noticeably lacking in most countries. Strengthening research capacity and training in population and public health in the WHO African region can only realize its full potential when bolstered by robust governmental and institutional backing. This support is crucial to create and sustain research hubs of excellence to conduct state-of-the-art, locally relevant African-led research initiatives. The results of this research should be disseminated in locally curated knowledge translation platforms that are easily accessible to stakeholders.

## Conclusion

Our results suggest that the oral health research output of the WHO African region has remained constant in the past 10 y, and it has focused on the conduct of cross-sectional studies describing the magnitude of oral health issues. Other primary study designs are infrequently conducted. This paucity of research evidence is also apparent in secondary (evidence synthesis) studies, including systematic reviews and policy briefs, essential bricks to support the creation of national and regional evidence-informed clinical practice guidelines and oral health policies. Research studies informing the effect of interventions to create locally tailored solutions to oral health issues and the assessment of the implementation and evaluation of those solutions remain understudied. Significant variation in research capabilities across countries represents an opportunity for oral health researchers from academic entities in the WHO African area to engage in dialogues about establishing intra- and intercountry partnerships. These collaborations can serve as the knowledge engine to inform and define African oral health research agendas, producing the necessary direct evidence to accelerate the creation, dissemination, implementation, monitoring, and evaluation of oral health policies.

## Author Contributions

T.F. Labarca, D. Ortuño, contributed to conception, protocol writing, and research design, created screening, data extraction forms, and instructions, contributed to citation screening and data extraction, arbiter for defining final eligibility, analyzed data, designed and created tables and figures, produced the first draft of the manuscript, reviewed and provided input into the manuscript. L. Neira, F.J. Bravo, C.R. Cantarutti, M. Dallaserra, A. Gatarayiha, R.J. Kulchar, C.C. Martins-Pfeifer, L. Pilcher, S. Pahlke, C. Pirela, A. Song, J.P. Vargas, C. Véliz, F. Verdugo-Paiva, P. Vergara, V. Zaffiri, J. Zuñiga, N. Olivares, contributed to citation screening and data extraction, reviewed and provided input into the manuscript. G. Andrade, M. Res, contributed to conception, protocol writing, and research design, contributed to citation screening and data extraction, reviewed and provided input into the manuscript. J. Karajgikar, X. Liu, designed and created the data visualization tool for the collaboration map, reviewed and provided input into the manuscript. J.M. Sanchez, retrieved citations and affiliations to inform the collaboration map using APIs, contributed to citation screening and data extraction, reviewed and provided input into the manuscript. O. Urquhart, contributed to conception, protocol writing, and research design, created screening, data extraction forms, and instructions, analyzed data, designed and created tables and figures, reviewed and provided input into the manuscript. Y. Makino, contributed to conception, protocol writing, and research design, analyzed data, designed and created tables and figures, reviewed and provided input into the manuscript. M. Glick, A. Carrasco-Labra, contributed to conception, protocol writing, and research design, created screening, data extraction forms, and instructions, analyzed data, designed and created tables and figures, produced the first draft of the manuscript, reviewed and provided input into the manuscript. All authors gave final approval and agreed to be accountable for all aspects of the work.

## Supplemental Material

sj-docx-1-jdr-10.1177_00220345241272024 – Supplemental material for Oral Health Research in the WHO African Region between 2011 and 2022: A Scoping Review Supplemental material, sj-docx-1-jdr-10.1177_00220345241272024 for Oral Health Research in the WHO African Region between 2011 and 2022: A Scoping Review by T.F. Labarca, D. Ortuño, L. Neira, G. Andrade, F.J. Bravo, C.R. Cantarutti, M. Dallaserra, A. Gatarayiha, J. Karajgikar, R.J. Kulchar, X. Liu, C.C. Martins-Pfeifer, N. Olivares, L. Pilcher, S. Pahlke, C. Pirela, J.M. Sanchez, A. Song, O. Urquhart, J.P. Vargas, C. Véliz, F. Verdugo-Paiva, P. Vergara, V. Zaffiri, J. Zuñiga, Y. Makino, M. Glick and A. Carrasco-Labra in Journal of Dental Research
