# Supplementary material for: Oral Health Research in the WHO African Region between 2011 and 2022: A Scoping Review
Source: J Dent Res. 2024 Oct 29;103(12):1209–17. doi: 10.1177/00220345241272024 (PMC11562290; doi:10.1177/00220345241272024)
Supplement: sj-docx-1-jdr-10.1177_00220345241272024 – Supplemental material for Oral Health Research in the WHO African Region between 2011 and 2022: A Scoping Review [file sj-docx-1-jdr-10.1177_00220345241272024.docx]

**Oral Health Research in the WHO African Region between 2011 and 2022: A scoping review.**

1. Tomás F. Labarca, M.Res.

Faculty of Dentistry, Universidad de los Andes, Monseñor Álvaro del Portillo 12455, Santiago, Las Condes, Región Metropolitana, Chile.

1. Duniel Ortuño, M.Sc.

Faculty of Dentistry, Universidad de los Andes, Monseñor Álvaro del Portillo 12455, Santiago, Las Condes, Región Metropolitana, Chile.

1. Luisa Neira, D.D.S.

Faculty of Dentistry, Universidad de los Andes, Monseñor Álvaro del Portillo 12455, Santiago, Las Condes, Región Metropolitana, Chile.

1. Gabriel Andrade, M.Res.

Faculty of Dentistry, Universidad de los Andes, Monseñor Álvaro del Portillo 12455, Santiago, Las Condes, Región Metropolitana, Chile

1. Francisco J. Bravo, D.D.S

Faculty of Dentistry, Universidad de los Andes, Monseñor Álvaro del Portillo 12455, Santiago, Las Condes, Región Metropolitana, Chile

1. Cynthia R. Cantarutti, M.P.H.

School of Dentistry, Faculty of Medicine, Pontificia Universidad Católica de Chile, Vicuña Mackenna 4860, Santiago, Región Metropolitana, Chile.

1. Matias Dallaserra, M.P.H.

Faculty of Dentistry, Department of Oral and Maxillofacial Surgery, Universidad de Chile, Olivos 943, Independencia, Región Metropolitana, Chile.

1. Agnes Gatarayiha, M.Sc.

School of Dentistry, College of Medicine and Health Sciences, University of Rwanda, KG 11 Ave 47, Remera Campus, Kigali, Rwanda.

1. Jajwalya Karajgikar, M.S.

Applied Data Science Librarian, Research Data and Digital Scholarship, University of Pennsylvania Libraries, 3420 Walnut St, Philadelphia, PA 19104.

1. Rachel J. Kulchar, B.A.

Center for Integrative Global Oral Health, School of Dental Medicine, University of Pennsylvania. 240 S 40th St, Philadelphia, PA 19104, United States.

1. Xiner Liu, M.S.Ed.

Text and Data Mining Assistant, Research Data and Digital Scholarship. Graduate School of Education, University of Pennsylvania. 240 S 40th St, Philadelphia, PA 19104, United States.

1. Carolina Castro Martins-Pfeifer, Ph.D.

Department of Pediatric Dentistry, School of Dentistry, Universidade Federal de Minas Gerais, Avenida Antônio Carlos 6627, Pampulha, Belo Horizonte, 31270-901, Brazil.

1. Nicolas Olivares, D.D.S.

School of Dentistry, Faculty of Medicine, Pontificia Universidad Católica de Chile, Vicuña Mackenna 4860, Santiago, Región Metropolitana, Chile.

1. Lauren Pilcher, M.S.P.H.

Quality Initiatives, American Academy of Pediatrics, 345 Park Boulevard, Itasca, IL, 60143, United States.

1. Sarah Pahlke, M.S.

Clinical Affairs and Practice Guidelines, Infectious Diseases Society of America, 4040 Wilson Boulevard, Suite 300, Arlington, VA, 22203. United States.

1. Carmen Pirela, D.D.S.

Faculty of Dentistry, Department of Conservative Dentistry, Universidad de Chile, Olivos 943, Independencia, Región Metropolitana, Chile.

1. Jorge M. Sanchez, M.Sc.

Mean Data Corporation, 8763 NW 140^TH^ LN Miami Lakes, FL 33018, United States.

1. Ana Song, B.S.

Center for Integrative Global Oral Health, School of Dental Medicine, University of Pennsylvania. 240 S 40th St, Philadelphia, PA 19104, United States.

1. Olivia Urquhart, M.P.H.

Center for Integrative Global Oral Health, School of Dental Medicine, University of Pennsylvania. 240 S 40th St, Philadelphia, PA 19104, United States.

1. Juan Pablo Vargas, D.D.S.

School of Dentistry, Faculty of Medicine, Pontificia Universidad Católica de Chile, Vicuña Mackenna 4860, Santiago, Región Metropolitana, Chile.

1. Claudia Véliz, M.P.H.

School of Dentistry, Faculty of Medicine, Pontificia Universidad Católica de Chile, Vicuña Mackenna 4860, Santiago, Región Metropolitana, Chile.

1. Francisca Verdugo-Paiva, M.Sc.

Epistemonikos Foundation, Mariano Sanchez Fontecilla 530, Santiago, Chile.

Orofacial Pain & TMD Program, Facultad de Odontología, Universidad Andrés Bello, Echaurren 237 Room #8, Santiago, Chile.

1. Pedro Vergara, D.D.S.

Faculty of Dentistry, Universidad de los Andes, Monseñor Álvaro del Portillo 12455, Santiago, Las Condes, Región Metropolitana, Chile

1. Valentina Zaffiri, D.D.S.

Faculty of Dentistry, Universidad de los Andes, Monseñor Álvaro del Portillo 12455, Santiago, Las Condes, Región Metropolitana, Chile

1. Javiera Zuñiga, B.S.

Faculty of Dentistry, Universidad de los Andes, Monseñor Álvaro del Portillo 12455, Santiago, Las Condes, Región Metropolitana, Chile

1. Yuka Makino, Ph.D.

Noncommunicable Diseases Management team, WHO Regional Office for Africa, Brazzaville, Congo.

27. Michael Glick, D.M.D.

Center for Integrative Global Oral Health, School of Dental Medicine, University of Pennsylvania. 240 S 40th St, Philadelphia, PA 19104, United States.

28. Alonso Carrasco-Labra, Ph.D.*

Center for Integrative Global Oral Health, School of Dental Medicine, University of Pennsylvania. 240 S 40th St, Philadelphia, PA 19104, United States.

*Corresponding author

Alonso Carrasco-Labra, Ph.D.

Center for Integrative Global Oral Health, School of Dental Medicine, University of Pennsylvania. 240 S 40th St, Philadelphia, PA 19104, United States.

Email: [carrascl@upenn.edu](mailto:carrascl@upenn.edu), Phone: +1 (215) 746-2944

ORCID iD: https://orcid.org/0000-0003-3546-3526

**Supplementary file**

[Figure 1. Prisma diagram of study selection. 5](#_Toc146588047)

[Figure 2. Number of oral health-related articles in the WHO African region published by year, during 2011-2022. 6](#_Toc146588048)

[Figure 3. Distribution of study discipline across included studies (n=2,337) 7](#_Toc146588049)

[Figure 4. Number of researchers who are authors in public health research-related studies in the WHO African region according to the subtopic addressed by the study (n= 1,296) and the number of articles produced (n=294). 8](#_Toc146588050)

[Table 1. Preferred Reporting Items for Systematic Reviews and Meta-analysis extension for scoping reviews (PRISMA-ScR) checklist. 9](#_Toc146588051)

[Table 2. Search strategies conducted in December 2022. 11](#_Toc146588052)

[Table 3. Citations of included articles published between 2011 and 2022 in the WHO African region. 12](#_Toc146588053)

[Table 4. Taxonomy of oral health topics and subcategories of articles published between 2011 and 2022 in WHO African region countries. 128](#_Toc146588054)

[Table 5. Summary of oral health research articles and the top 20 journals with the highest number of publications in the WHO African region between 2011 and 2022. 131](#_Toc146588055)

# **Appendix Figure 1. Prisma diagram of study selection.**


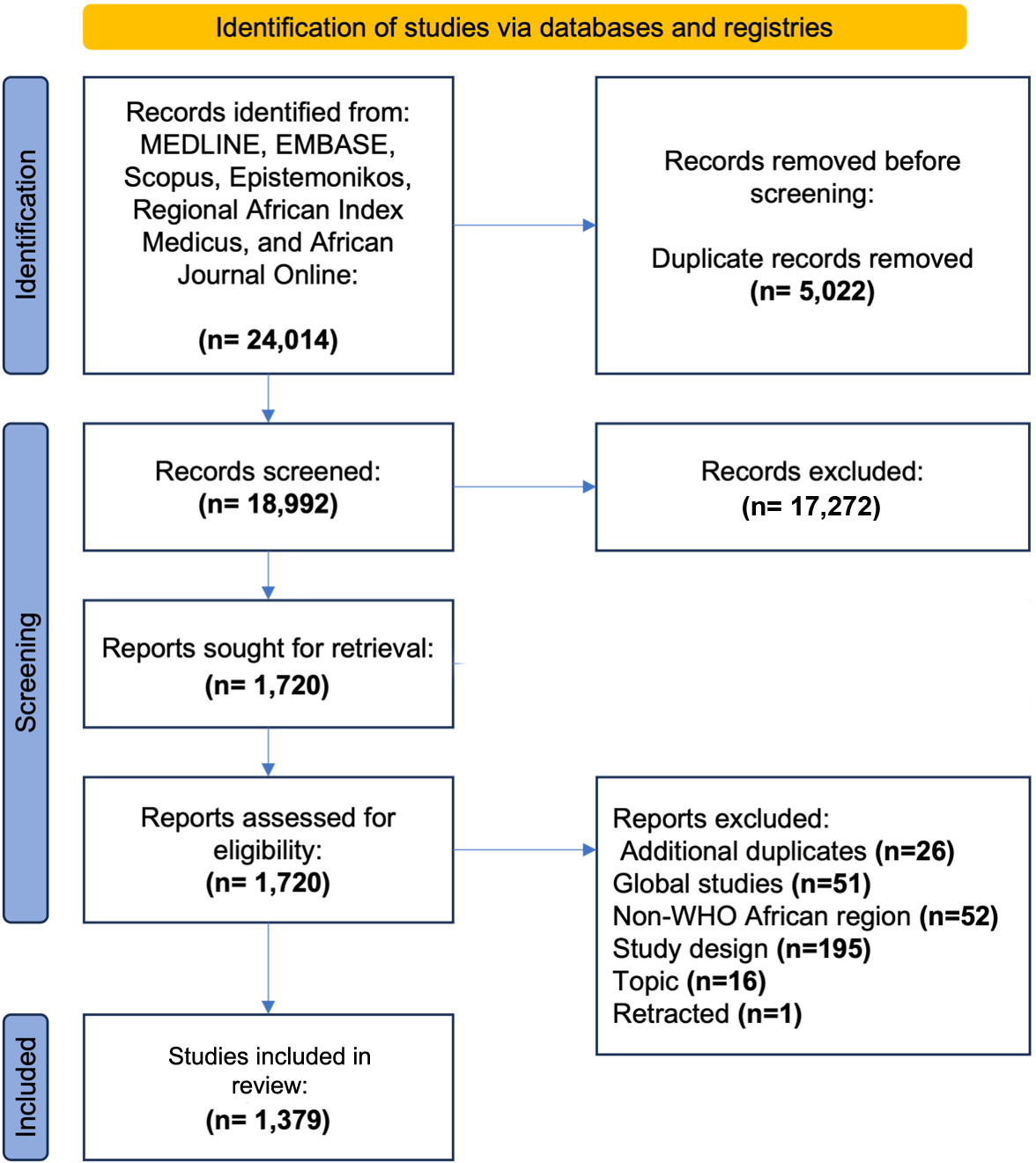


# **Appendix Figure 2. Number of oral health-related articles published in WHO African region countries by year, between 2011-2022.**

#
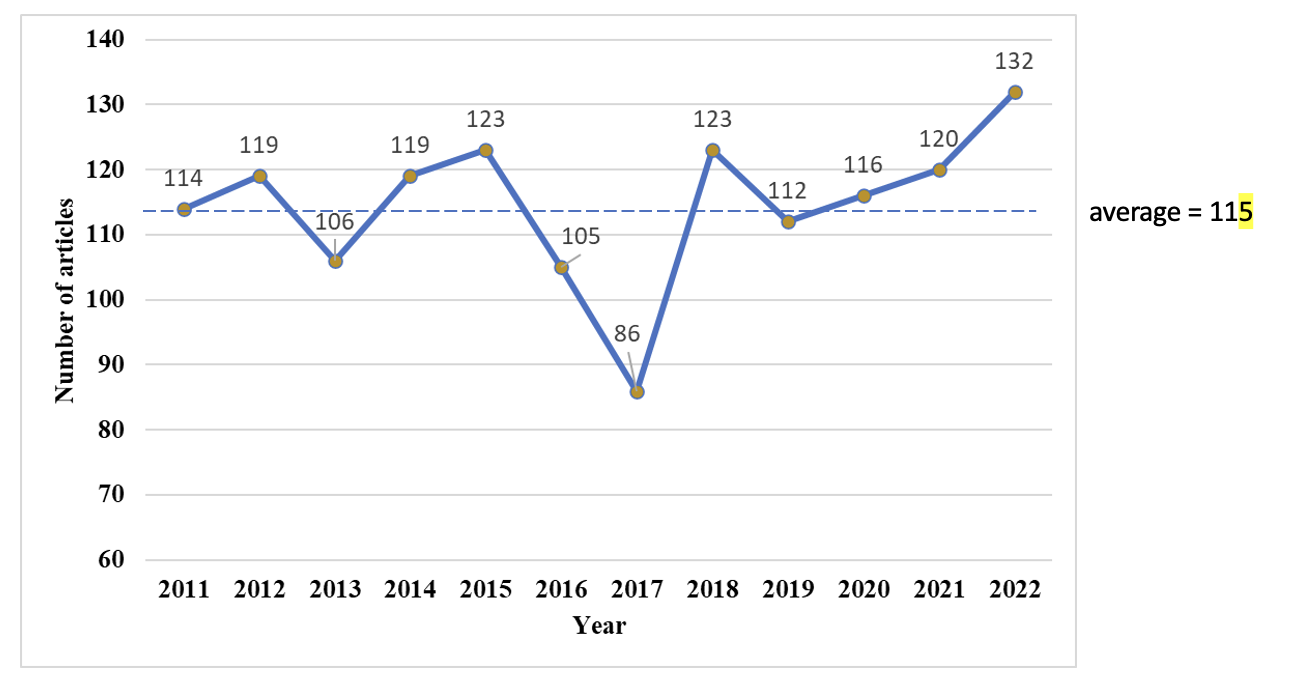


# **Appendix Figure 3. Distribution of study disciplines across included studies in the WHO African region countries between 2011-2022 (n=2,337)*.**


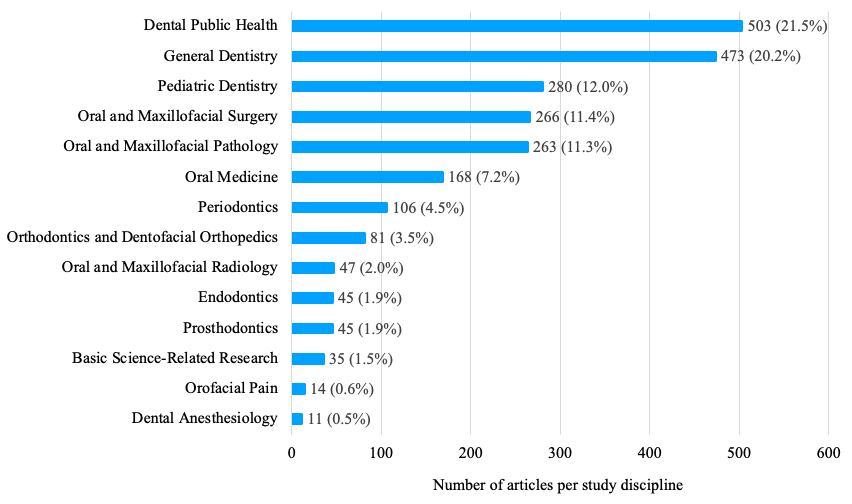


*Some studies were classified into more than one discipline, which explains the discrepancy between the identified studies from the search (n=1,379) and the disciplines presented in the figure.

**Appendix Figure 4. Number of researchers (n= 1,296)* who are authors of public health research-related studies in the WHO African region countries between 2011-2022, according to the subtopic addressed by the study and the number of articles produced (n=294)** †**.**


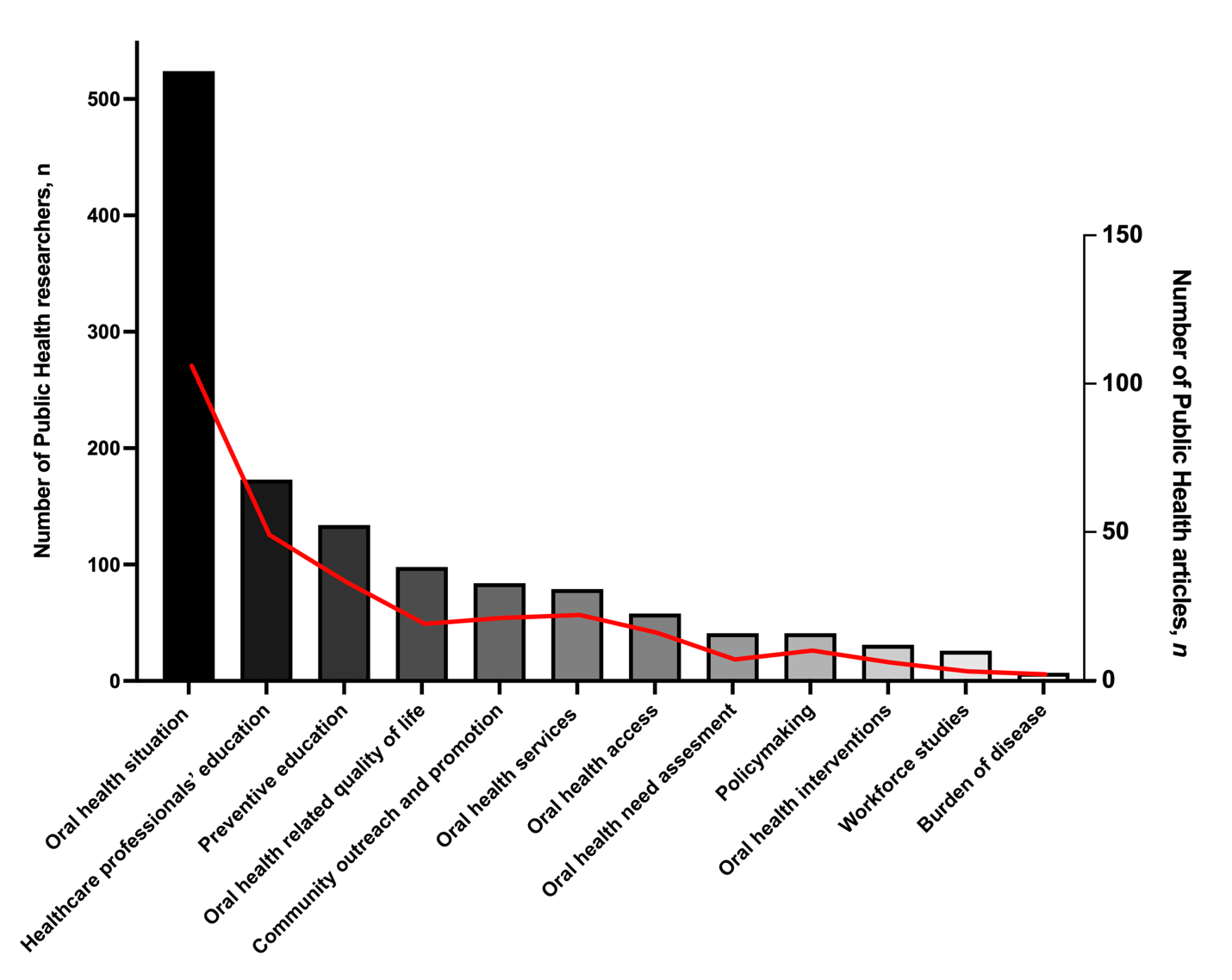


* This number represents the total number of appearances of investigators in public health research studies. One investigator may have contributed to more than one study.

† Number of unique public health research-related studies. The red line indicates the number of articles per subtopic.

**Appendix Table 1. Preferred Reporting Items for Systematic Reviews and Meta-analysis extension for scoping reviews (PRISMA-ScR) checklist.**

| **SECTION** | **ITEM** | **PRISMA-ScR CHECKLIST ITEM** | **REPORTED ON PAGE #** |
| --- | --- | --- | --- |
| **TITLE** | | | |
| Title | 1 | Identify the report as a scoping review. | 1 |
| **ABSTRACT** | | | |
| Structured summary | 2 | Provide a structured summary that includes (as applicable): background, objectives, eligibility criteria, sources of evidence, charting methods, results, and conclusions that relate to the review questions and objectives. | 5 |
| **INTRODUCTION** | | | |
| Rationale | 3 | Describe the rationale for the review in the context of what is already known. Explain why the review questions/objectives lend themselves to a scoping review approach. | 6-7 |
| Objectives | 4 | Provide an explicit statement of the questions and objectives being addressed with reference to their key elements (e.g., population or participants, concepts, and context) or other relevant key elements used to conceptualize the review questions and/or objectives. | 7 |
| **METHODS** | | | |
| Protocol and registration | 5 | Indicate whether a review protocol exists; state if and where it can be accessed (e.g., a Web address); and if available, provide registration information, including the registration number. | 8 |
| Eligibility criteria | 6 | Specify characteristics of the sources of evidence used as eligibility criteria (e.g., years considered, language, and publication status), and provide a rationale. | 8 |
| Information sources* | 7 | Describe all information sources in the search (e.g., databases with dates of coverage and contact with authors to identify additional sources), as well as the date the most recent search was executed. | 8-9 |
| Search | 8 | Present the full electronic search strategy for at least 1 database, including any limits used, such that it could be repeated. | Supplementary Table 2 |
| Selection of sources of evidence† | 9 | State the process for selecting sources of evidence (i.e., screening and eligibility) included in the scoping review. | 9 |
| Data charting process‡ | 10 | Describe the methods of charting data from the included sources of evidence (e.g., calibrated forms or forms that have been tested by the team before their use, and whether data charting was done independently or in duplicate) and any processes for obtaining and confirming data from investigators. | 9-11 |
| Data items | 11 | List and define all variables for which data were sought and any assumptions and simplifications made. | 9-11 |
| Critical appraisal of individual sources of evidence§ | 12 | If done, provide a rationale for conducting a critical appraisal of included sources of evidence; describe the methods used and how this information was used in any data synthesis (if appropriate). | N/A |
| Synthesis of results | 13 | Describe the methods of handling and summarizing the data that were charted. | 9-11 |
| **RESULTS** | | | |
| Selection of sources of evidence | 14 | Give numbers of sources of evidence screened, assessed for eligibility, and included in the review, with reasons for exclusions at each stage, ideally using a flow diagram. | 12 |
| Characteristics of sources of evidence | 15 | For each source of evidence, present characteristics for which data were charted and provide the citations. | 12 |
| Critical appraisal within sources of evidence | 16 | If done, present data on critical appraisal of included sources of evidence (see item 12). | N/A |
| Results of individual sources of evidence | 17 | For each included source of evidence, present the relevant data that were charted that relate to the review questions and objectives. | 12-13 |
| Synthesis of results | 18 | Summarize and/or present the charting results as they relate to the review questions and objectives. | 12 |
| **DISCUSSION** | | | |
| Summary of evidence | 19 | Summarize the main results (including an overview of concepts, themes, and types of evidence available), link to the review questions and objectives, and consider the relevance to key groups. | 14 |
| Limitations | 20 | Discuss the limitations of the scoping review process. | 18 |
| Conclusions | 21 | Provide a general interpretation of the results with respect to the review questions and objectives, as well as potential implications and/or next steps. | 19 |
| **FUNDING** | | | |
| Funding | 22 | Describe sources of funding for the included sources of evidence, as well as sources of funding for the scoping review. Describe the role of the funders of the scoping review. | 20 |

**Appendix Table 2. Search strategies, December 2022.**

PubMed search strategy

| #1 | (dentist* OR dental* OR dentin* OR dentate* OR dentition* OR teeth* OR tooth* OR odonto* OR molar* OR maxilla* OR maxillo* OR maxille* OR mandib* OR jaw OR temporomandib* OR "temporo-mandibular" OR "temporo mandibular" OR tmj OR tmd OR craniomandib* OR "cranio-mandibular" OR "cranio mandibular" OR craniomaxillo* OR craniofacial* OR "cranio-facial" OR "cranio facial" OR prosthodont* OR edentul* OR exodont* OR "oral health" OR "oral care" OR "oral hygiene" OR "oral cancer" OR "oral surgery" OR orofacial* OR "oro-facial" OR "oro facial" OR gingivit* OR periodont* OR endodontic* OR "root canal" OR orthodont* OR caries* OR carious* OR mouth* OR bruxism* OR dentur* OR cariology* OR teledentist* OR gerodontology OR keratocyst* OR "odontogenic cysts" OR "dentofacial anomalies" OR "noma disease" OR NOMA OR "oral disease" OR "Oral Health"[Mesh] OR "Dentistry"[Mesh] OR "Public Health Dentistry"[Mesh] OR "Preventive Dentistry"[Mesh] OR "Pediatric Dentistry"[Mesh] OR "Forensic Dentistry"[Mesh] OR "Geriatric Dentistry"[Mesh] OR "Community Dentistry"[Mesh] OR "Dentistry, Operative"[Mesh] OR "Dental Care for Chronically Ill"[Mesh] OR "Dental Care for Disabled"[Mesh] OR "Dental Care for Children"[Mesh] OR "Esthetics, Dental"[Mesh] OR "Dental Caries"[Mesh] OR "Tooth Diseases"[Mesh] OR "Noma"[Mesh]) |
| --- | --- |
| #2 | (Algeria OR Angola OR Benin OR Botswana OR "Burkina Faso" OR Burundi OR "Cabo Verde" OR Cameroon OR "Central African Republic" OR Chad OR Comoros OR Congo OR "Cote d'Ivoire" OR Congo OR "Equatorial Guinea" OR Eritrea OR Eswatini OR Ethiopia OR Gabon OR Gambia OR Ghana OR Guinea OR "Guinea Bissau" OR Kenya OR Lesotho OR Liberia OR Madagascar OR Malawi OR Mali OR Mauritania OR Mauritius OR Mozambique OR Namibia OR Niger OR Nigeria OR Rwanda OR "Sao Tome and Principe" OR Senegal OR Seychelles OR "Sierra Leone" OR "South Africa" OR "South Sudan" OR Togo OR Uganda OR Tanzania OR Zambia OR Zimbabwe OR Africa) |
| #3 | #1 AND #2 |
| #4 | limit #3 to yr=2011-2022 |

**Appendix Table 3. Citations of included articles published between 2011 and 2022 in the WHO African region.**

| 1 |
| --- |
| Ojukwu BT, Balarabe SA, Abdullahi MAS, *et al.* Workplace assault and its’ impact to service delivery amongst dental professionals at Tertiary Hospitals in Northern Nigeria. *Niger J Dent Res* 2020; **5**: 123–30. |
| 2 |
| Gothey AI, Lembariti BS. Work-related Musculoskeletal Disorders and Associated Factors among Dental Professionals in Tanzania. *International Journal of Prosthodontics and Restorative Dentistry* 2022; **16**: 26–9. |
| 3 |
| Kutesa A, Nkamba EM, Muwazi L, Buwembo W, Rwenyonyi CM. Weight, height and eruption times of permanent teeth of children aged 4-15 years in Kampala, Uganda. *BMC Oral Health* 2013; **13**. DOI:[10.1186/1472-6831-13-15](https://doi.org/10.1186/1472-6831-13-15). |
| 4 |
| Faye D, Mbacké Lo CM, Kanouté A. Waste management from dental care in the health districts of Dakar, Senegal. *Medecine et Sante Tropicales* 2014; **24**: 403–8. |
| 5 |
| Owotade FJ, Patel M. Virulence of oral Candida isolated from HIV-positive women with oral candidiasis and asymptomatic carriers. *Oral Surg Oral Med Oral Pathol Oral Radiol* 2014; **118**: 455–60. |
| 6 |
| Reddy M, Singh S. Viability in delivering oral health promotion activities within the Health Promoting Schools Initiative in KwaZulu-Natal. *SAJCH South African Journal of Child Health* 2015; **9**: 93–7. |
| 7 |
| Eigbobo JO, Sote EO, Oredugba FA. Variations of crown dimensions of permanent dentitions in a selected population of Nigerian children. *Nigerian quarterly journal of hospital medicine* 2011; **21**: 163–8. |
| 8 |
| Kihara E, Galic I, Nyamunga D, Mehdi F, Velandia Palacio LA, Cameriere R. Validation of the Italian, European, North German, Malaysian, and South African black formulas on Cameriere method using panoramic radiographs in Kenyan children. *International Journal of Legal Medicine* 2022; **136**: 1495–506. |
| 9 |
| Cadenas de Llano-Pérula M, Kihara E, Thevissen P, *et al.* Validating dental age estimation in Kenyan black children and adolescents using the Willems method. *Medicine, Science and the Law* 2021; **61**: 180–5. |
| 10 |
| Akinboboye BO, Akeredolu PA, Sofola O, Ogunrinde BO, Oremosu OA. Utilization of Teeth Replacement Service among the Elderly Attending Teaching Hospitals in Lagos; Nigeria. *Ann med health sci res (Online)* 2014; **4**: 57–60. |
| 11 |
| Makanjuola JO, Uti OG, Sofola OO. Utilization of Oral Health Care Services by University Undergraduates in Lagos, Nigeria. *Nigerian quarterly journal of hospital medicine* 2015; **25**: 106–11. |
| 12 |
| Eigbobo JO, Obiajunwa CC. Utilization of dental services among secondary school students in Port Harcourt, Nigeria. *European Journal of General Dentistry* 2016; **5**: 74–9. |
| 13 |
| Arigbede A, Denloye O, Dosumu O. Use of simulators in operative dental education: Experience in southern Nigeria. *African Health Sciences* 2015; **15**: 269–77. |
| 14 |
| Ranjarisoa LN, Razanamihaja N, Rafatro H. Use of plants in oral health care by the population of Mahajanga, Madagascar. *Journal of Ethnopharmacology* 2016; **193**: 179–94. |
| 15 |
| Anyanechi C, Osunde O, Bassey G. Use of oral mucoperiosteal and pterygo-masseteric muscle flaps as interposition material in surgery of temporomandibular joint ankylosis: a comparative study. *Ann Med Health Sci Res* 2015; **5**: 30–5. |
| 16 |
| Ariemba RM, Maina SW, Gathece LW. Use of nasal width to estimate the mesio-distal widths of maxillary anterior teeth in a Kenyan population of African descent. *East African Medical Journal* 2016; **93**: 398–405. |
| 17 |
| Folayan MO, Kolawole KA, Chukwumah NM, *et al.* Use of caries prevention tools and associated caries risk in a suburban population of children in Nigeria. *European Archives of Paediatric Dentistry* 2016; **17**: 187–93. |
| 18 |
| Olusanya AA, Adeleye AO, Aladelusi TO, Fasola AO. Updates on the epidemiology and pattern of traumatic maxillofacial injuries in a nigerian university teaching hospital: a 12-month prospective cohort in-hospital outcome study. *Craniomaxillofac Trauma Reconstr* 2015; **8**: 50–8. |
| 19 |
| Ghotane SG, Challacombe SJ, Don-Davis P, Kamara D, Gallagher JE. Unmet need in Sierra Leone: a national oral health survey of schoolchildren. *BDJ Open* 2022; **8**. DOI:[10.1038/s41405-022-00107-7](https://doi.org/10.1038/s41405-022-00107-7). |
| 20 |
| Beukes J, Reyneke JP, Damstra J. Unilateral sagittal split mandibular ramus osteotomy: indications and geometry. *Br J Oral Maxillofac Surg* 2016; **54**: 219–23. |
| 21 |
| Adetayo AM, Adetayo MO, Adeyemo WL, James OO, Adeyemi MO. Unilateral cleft lip: evaluation and comparison of treatment outcome with two surgical techniques based on qualitative (subject/guardian and professional) assessment. *J Korean Assoc Oral Maxillofac Surg* 2019; **45**: 141–51. |
| 22 |
| Adetayo AM, James O, Adeyemo WL, Ogunlewe MO, Butali A. Unilateral cleft lip repair: a comparison of treatment outcome with two surgical techniques using quantitative (anthropometry) assessment. *J Korean Assoc Oral Maxillofac Surg* 2018; **44**: 3–11. |
| 23 |
| Isiekwe GI, Umeizudike KA, Abah AA, Fadeju AD. Undergraduate dental education in Nigeria: perceptions of dental students and recent dental graduates. *Odonto-stomatologie tropicale = Tropical dental journal* 2016; **39**: 15–23. |
| 24 |
| Broder HL, Tormeti D, Kurtz AL, *et al.* Type II diabetes and oral health: Perceptions among adults with diabetes and oral/health care providers in Ghana. *Community Dental Health* 2014; **31**: 158–62. |
| 25 |
| Mercy O. Type and prevalence of oral lesions seen in a teaching hospital. *Journal of Medicine and Biomedical Research* 2015; **14**: 151–6. |
| 26 |
| Ibiyemi O, Oke GA, Jeboda SO. Two years survival rate of occlusal ART restorations placed without tooth surface conditioning in a primary oral health care centre. *African Journal of Biomedical Research* 2012; **15**: 65–70. |
| 27 |
| Teshome A, Andualem G, Tsegie R, Seifu S. Two years retrospective study of maxillofacial trauma at a tertiary center in North West Ethiopia. *BMC Research Notes* 2017; **10**. DOI:[10.1186/s13104-017-2670-1](https://doi.org/10.1186/s13104-017-2670-1). |
| 28 |
| Ayo-Yusuf OA, Lalloo R, Johnson NW. Trends and ethnic disparities in oral and oro-pharyngeal cancers in South Africa, 1992-2001. *SADJ : journal of the South African Dental Association = tydskrif van die Suid-Afrikaanse Tandheelkundige Vereniging* 2013; **68**: 168–73. |
| 29 |
| Gbadebo SO, Anifowose OO, Ogunrinde TJ. Trend of posterior teeth restoration at ibadan, Nigeria. *J West Afr Coll Surg* 2013; **3**: 70–83. |
| 30 |
| Ogunrinde TJ, Gbadebo SO, Sulaiman AO. Trend in prosthetic rehabilitation of partially edentulous patients in a Nigerian teaching hospital. *J West Afr Coll Surg* 2015; **5**: 84–99. |
| 31 |
| Ajayi YO, Nwachukwu N, Akinboboye BO. Treatment outcome of short dental implants. *J West Afr Coll Surg* 2017; **7**: 52–71. |
| 32 |
| Makanjuola JO, Umesi DC, Oderinu OH. Treatment outcome of manual versus rotary techniques in single-visit endodontics for patients in a nigerian teaching hospital: a randomized clinical trial. *J West Afr Coll Surg* 2018; **8**: 44–75. |
| 33 |
| Makanjuola JO, Oderinu OH, Umesi DC. Treatment Outcome and Root Canal Preparation Techniques: 5-Year Follow-Up. *Int Dent J* 2022; **72**: 811–8. |
| 34 |
| Agbara R, Fomete B, Omeje KU, Obiadazie AC, Ajike SO. Treatment of complications following orofacial gangrenous infection in a resource limited setting: Experiences and a proposed classification. *Egyptian Journal of Ear, Nose, Throat and Allied Sciences* 2018; **19**: 58–63. |
| 35 |
| Famurewa BA, Aregbesola SB, Alade OT, Akinniyi TA. Treatment costs of mandibular fractures in a Nigerian hospital. *Oral and Maxillofacial Surgery* 2022; **26**: 417–22. |
| 36 |
| Nzomiwu CL, Sote EO, Oredugba FA. Translation and Validation of the Nigerian Pidgin English Version of the Early Childhood Oral Health Impact Scale (NAIJA ECOHIS). *West Afr J Med* 2018; **35**: 102–8. |
| 37 |
| Nwafor CC, Forae GD, Aligbe JU. Topographic pattern distribution of head and neck squamous cell carcinoma in Benin-City, Nigeria: How common are these lesions? *Journal of Medicine and Biomedical Research* 2015; **14**: 50–5. |
| 38 |
| Anyanechi C, Saheeb B. Toothache and self-medication practices: a study of patients attending a niger delta tertiary hospital in Nigeria. *Ann Med Health Sci Res* 2014; **4**: 884–8. |
| 39 |
| Azodo CC, Ololo O. Toothache among dental patients attending a Nigerian secondary healthcare setting. *Stomatologija / issued by public institution ‘Odontologijos studija’ . [et al]* 2013; **15**: 135–40. |
| 40 |
| Ajayi DM, Gbadebo SO, Abiodun-Solanke IMF. Tooth reattachment: Knowledge and practice of Nigerian dentists in postgraduate training. *Journal of Stomatology* 2017; **70**: 679–85. |
| 41 |
| Okoje VN, Dosumu OO, Alonge TO, Onyeaso C. Tooth loss: Are the patients prepared? *Nigerian Journal of Clinical Practice* 2012; **15**: 172–5. |
| 42 |
| Akpata E, Otoh E, Enwonwu C, Adeleke O, Joshipura K. Tooth loss, chewing habits, and food choices among older Nigerians in Plateau State: A preliminary study. *Community Dentistry and Oral Epidemiology* 2011; **39**: 409–15. |
| 43 |
| Idowu EA, Taiwo OO, Fakuade BO, Afolabi AO. Tooth loss awareness and reasons for non-replacement of missing teeth among nurses at Jos University Teaching Hospital, Jos, Nigeria. *Niger J Dent Res* 2020; **5**: 136–44. |
| 44 |
| Ibiyemi O, Idiga E. Tooth loss among the elders in an inner city area of Ibadan, Nigeria. *Gerodontology* 2017; **34**: 264–71. |
| 45 |
| Taiwo AO, Ibikunle AA, Braimah RO, Sulaiman OA, Gbotolorun OM. Tooth extraction: Pattern and etiology from extreme Northwestern Nigeria. *European Journal of Dentistry* 2017; **11**: 335–9. |
| 46 |
| Ouedraogo Y, Kabore WA, Konsem T, *et al.* Tooth decay: epidemiological and therapeutic aspects in dental service of University Teaching Hospital Yalgado Ouedraogo and Municipal Centre of Oral Health. *Odonto-stomatologie tropicale = Tropical dental journal* 2015; **38**: 49–55. |
| 47 |
| Azodo CC, Ogbomo AC, Agbor MA. Tooth bleaching and young adults in Nigeria: knowledge, experiences and intention. *Odonto-stomatologie tropicale = Tropical dental journal* 2012; **35**: 47–54. |
| 48 |
| Azodo CC, Omili M. Tobacco use, alcohol consumption and self-rated oral health among Nigerian prison officials. *International Journal of Preventive Medicine* 2014; **5**: 1364–71. |
| 49 |
| Wood NH, Ayo-Yusuf OA, Gugushe TS, Bogers J-P. Tobacco use and oral sex practice among dental clinic attendees. *PLoS ONE* 2019; **14**. DOI:[10.1371/journal.pone.0213729](https://doi.org/10.1371/journal.pone.0213729). |
| 50 |
| Akaji EA, Folaranmi N. Tobacco use and oral health of inmates in a nigerian prison. *Nigerian Journal of Clinical Practice* 2013; **16**: 473–7. |
| 51 |
| Folayan MO, Oyedele TA, Oziegbe E. Time expended on managing molar incisor hypomineralization in a pediatric dental clinic in Nigeria. *Brazilian Oral Research* 2018; **32**. DOI:[10.1590/1807-3107BOR-2018.VOL32.0079](https://doi.org/10.1590/1807-3107BOR-2018.VOL32.0079). |
| 52 |
| Cavrić J, Galić I, Vodanović M, *et al.* Third molar maturity index (I3M) for assessing age of majority in a black African population in Botswana. *International Journal of Legal Medicine* 2016; **130**: 1109–20. |
| 53 |
| Esan T, Schepartz LA. Third molar impaction and agenesis: influence on anterior crowding. *Annals of Human Biology* 2017; **44**: 46–52. |
| 54 |
| Ucheonye IJ, Tokunbo AA, Donald OO. The Wits appraisal among a Nigerian sub-population: An assessment of dental base geometric factors. *Brazilian Journal of Oral Sciences* 2013; **12**: 275–9. |
| 55 |
| Ogunmuyiwa SA, Fatusi OA, Ugboko VI, Ayoola OO, Maaji SM. The validity of ultrasonography in the diagnosis of zygomaticomaxillary complex fractures. *International Journal of Oral and Maxillofacial Surgery* 2012; **41**: 500–5. |
| 56 |
| Pearce EC, Mainthia R, Freeman KL, Mueller JL, Rohde SL, Netterville JL. The usefulness of a yearly head and neck surgery trip to rural Kenya. *Otolaryngology - Head and Neck Surgery (United States)* 2013; **149**: 727–32. |
| 57 |
| Moshy JR, Sohal KS. The types, incidence and demographic distribution of benign oral and maxillofacial neoplasms among patients attending Muhimbili National hospital in Tanzania, 2008-2013. *Tanzania Journal of Health Research* 2016; **18**. DOI:[10.4314/thrb.v18i2.2](https://doi.org/10.4314/thrb.v18i2.2). |
| 58 |
| Esan TA, Schepartz LA. The timing of permanent tooth development in a Black Southern African population using the Demirjian method. *International Journal of Legal Medicine* 2019; **133**: 257–68. |
| 59 |
| Eigbobo JO, Nzomiwu CL, Amobi EO, Etim SS. The standard of playgrounds and safety measures in prevention of traumatic dental injuries in nigerian primary schools. *J West Afr Coll Surg* 2014; **4**: 82–99. |
| 60 |
| Aikins EA, Eigbobo JO, Onyeaso CO. The Spectrum of Pulpal Diseases and Therapies in Children in a Tertiary Centre in Nigeria. *port harcourt med J* 2013; **7**: 16–23. |
| 61 |
| Blake N, Edeling H, Bekker D, *et al.* The spectrum of orofacial clefts and treatment logistics at Universitas Academic Hospital, Bloemfontein, South Africa. *South African Journal of Surgery* 2021; **59**: 57–61. |
| 62 |
| Ramdial S, Madaree A. The spectrum of intraoral bacteria seen in patients with cleft palates in an African setting. *MicrobiologyOpen* 2019; **8**. DOI:[10.1002/mbo3.679](https://doi.org/10.1002/mbo3.679). |
| 63 |
| Agbor AM, Naidoo S, Mbia AM. The role of traditional healers in tooth extractions in Lekie Division, Cameroon. *Journal of Ethnobiology and Ethnomedicine* 2011; **7**. DOI:[10.1186/1746-4269-7-15](https://doi.org/10.1186/1746-4269-7-15). |
| 64 |
| Ndiaye C, Alemany L, Diop Y, *et al.* The role of human papillomavirus in head and neck cancer in Senegal. *Infectious Agents and Cancer* 2013; **8**. DOI:[10.1186/1750-9378-8-14](https://doi.org/10.1186/1750-9378-8-14). |
| 65 |
| Eshete M, Butali A, Abate F, *et al.* The Role of Environmental Factors in the Etiology of Nonsyndromic Orofacial Clefts. *J Craniofac Surg* 2020; **31**: 113–6. |
| 66 |
| Akpata O, Omoregie OF, Ibhawoh LO. The role of dentists as a stop-gap in the initial management of patients with delusional halitosis. *West African journal of medicine* 2013; **32**: 291–6. |
| 67 |
| Mucheto P, Makoni F, Mahachi L, *et al.* The role of dental practitioners in Provider Initiated HIV Counseling and Testing (PITC) for patients attending dental practices in Harare, Zimbabwe. *The Central African journal of medicine* 2012; **58**: 39–43. |
| 68 |
| Okolo CC, Oredugba FA, Denloye OO, Adeyemo YI. The risk factors and pattern of traumatic dental injuries in 10-12-year olds in Kano, Nigeria. *The Nigerian postgraduate medical journal* 2022; **29**: 272–7. |
| 69 |
| Nyamuryekung’e KK, Lahti SM, Tuominen RJ. The relative patient costs and availability of dental services, materials and equipment in public oral care facilities in Tanzania. *BMC Oral Health* 2015; **15**. DOI:[10.1186/s12903-015-0061-3](https://doi.org/10.1186/s12903-015-0061-3). |
| 70 |
| Naidoo S, Sheiham A, Tsakos G. The relation between oral impacts on daily performances and perceived clinical oral conditions in primary school children in the Ugu District, Kwazulu Natal, South Africa. *SADJ : journal of the South African Dental Association = tydskrif van die Suid-Afrikaanse Tandheelkundige Vereniging* 2013; **68**: 214–8. |
| 71 |
| Reddy M, Singh S. The promotion of oral health in health-promoting schools in Kwazulu-Natal Province, South Africa. *SAJCH South African Journal of Child Health* 2017; **11**: 16–20. |
| 72 |
| Ambe NF, Longdoh NA, Tebid P, *et al.* The prevalence, risk factors and antifungal sensitivity pattern of oral candidiasis in hiv/aids patients in Kumba District Hospital, South West Region, Cameroon. *Pan African Medical Journal* 2020; **36**: 1–14. |
| 73 |
| Temilola DO, Folayan MO, Fatusi O, *et al.* The prevalence, pattern and clinical presentation of developmental dental hard-tissue anomalies in children with primary and mix dentition from Ile-Ife, Nigeria. *BMC Oral Health* 2014; **14**. DOI:[10.1186/1472-6831-14-125](https://doi.org/10.1186/1472-6831-14-125). |
| 74 |
| Fernandes NA, Herbst D, Postma TC, Bunn BK. The prevalence of second canals in the mesiobuccal root of maxillary molars: A cone beam computed tomography study. *Aust Endod J* 2019; **45**: 46–50. |
| 75 |
| Farley E, Oyemakinde MJ, Schuurmans J, *et al.* The prevalence of noma in northwest Nigeria. *BMJ Global Health* 2020; **5**. DOI:[10.1136/bmjgh-2019-002141](https://doi.org/10.1136/bmjgh-2019-002141). |
| 76 |
| Olatosi OO, Inem V, Sofola OO, Prakash P, Sote EO. The prevalence of early childhood caries and its associated risk factors among preschool children referred to a tertiary care institution. *Nigerian Journal of Clinical Practice* 2015; **18**: 493–501. |
| 77 |
| Ogunrinde TJ, Olawale OF. The prevalence of denture related mucosa lesions among patients managed in a Nigerian teaching hospital. *The Pan African medical journal* 2020; **37**: 358. |
| 78 |
| Tefera AT, Girma B, Adane A, *et al.* The prevalence of dental caries and associated factors among students living with disability in the Amhara region, Ethiopia. *Clinical and Experimental Dental Research* 2022. DOI:[10.1002/cre2.646](https://doi.org/10.1002/cre2.646). |
| 79 |
| Birungi N, Fadnes LT, Engebretsen IMS, Tumwine JK, Åstrøm AN. The prevalence and socio-behavioural and clinical covariates of oral health related quality of life in Ugandan mothers with and without HIV-1. *Health and Quality of Life Outcomes* 2021; **19**. DOI:[10.1186/s12955-021-01844-3](https://doi.org/10.1186/s12955-021-01844-3). |
| 80 |
| Natto ZS, Petersen FF, Niccola Q. The prevalence and severity of dental caries in Chad: a pilot study. *The Nigerian postgraduate medical journal* 2014; **21**: 241–4. |
| 81 |
| Temilola OD, Folayan MO, Oyedele T. The prevalence and pattern of deciduous molar hypomineralization and molar-incisor hypomineralization in children from a suburban population in Nigeria. *BMC Oral Health* 2015; **15**. DOI:[10.1186/s12903-015-0059-x](https://doi.org/10.1186/s12903-015-0059-x). |
| 82 |
| Teshome A, Derese K, Andualem G. The prevalence and determinant factors of oral halitosis in northwest ethiopia: A cross-sectional study. *Clinical, Cosmetic and Investigational Dentistry* 2021; **13**: 173–9. |
| 83 |
| Eigbobo JO, Etim SS. The pattern of traumatic dental injuries in children in a tertiary health care facility in Nigeria. *Journal of International Dental and Medical Research* 2016; **9**: 33–8. |
| 84 |
| Eigbobo JO, Etim SS. The pattern of dental caries in children in port harcourt, nigeria. *J West Afr Coll Surg* 2015; **5**: 20–41. |
| 85 |
| Butt FMA, Guthua SW, Awange DA, Dimba EAO, MacIgo FG. The pattern and occurrence of ameloblastoma in adolescents treated at a university teaching hospital, in Kenya: A 13-year study. *Journal of Cranio-Maxillofacial Surgery* 2012; **40**: e39–45. |
| 86 |
| Ojahanon PI, Akionbare O, Umoh AO. The oral hygiene status of institution dwelling orphans in Benin City, Nigeria. *Nigerian Journal of Clinical Practice* 2013; **16**: 41–4. |
| 87 |
| Naidoo M, Singh S. The Oral health status of children with autism Spectrum disorder in KwaZulu-Nata, South Africa. *BMC Oral Health* 2018; **18**. DOI:[10.1186/s12903-018-0632-1](https://doi.org/10.1186/s12903-018-0632-1). |
| 88 |
| Ernesta K. The oral health status of children aged 12 years in Seychelles in the years 1994 and 2000. *NeuroToxicology* 2020; **81**: 254–8. |
| 89 |
| Zumpe L, Bensel T, Wienke A, Mtaya-Mlangwa M, Hey J. The oral health situation of 12-year-old school children in the rural region of ilembula in Southwestern Tanzania: A cross-sectional study. *International Journal of Environmental Research and Public Health* 2021; **18**. DOI:[10.3390/ijerph182212237](https://doi.org/10.3390/ijerph182212237). |
| 90 |
| Ogunrinde TJ, Dosumu OO, Shaba OP, Akeredolu PA, Ajayi MD. The influence of the design of mandibular major connectors on gingival health. *African journal of medicine and medical sciences* 2014; **43**: 29–33. |
| 91 |
| Olze A, Van Niekerk P, Schulz R, Ribbecke S, Schmeling A. The influence of impaction on the rate of third molar mineralisation in male black Africans. *International Journal of Legal Medicine* 2012; **126**: 869–74. |
| 92 |
| Ogunrinde TJ, Dosumu OO. The influence of demographic factors and medical conditions on patients complaints with complete dentures. *Ann Ib Postgrad Med* 2012; **10**: 16–21. |
| 93 |
| Eshete M, Gravenm PE, Topstad T, Befikadu S. The incidence of cleft lip and palate in Addis Ababa, Ethiopia. *Ethiopian Medical Journal* 2011; **49**: 1–5. |
| 94 |
| Bhayat A, Vergotine G, Yengopal V, Rudolph MJ. The impact of service-learning on two groups of South African dental students. *Journal of Dental Education* 2011; **75**: 1482–8. |
| 95 |
| Adeyemi TE, Otuyemi OD. The impact of playing wind musical instruments on the dental arch dimensions in a male west african population. *Niger J Clin Pract* 2020; **23**: 1120–6. |
| 96 |
| Andersson P, Kavakure J, Lingström P. The impact of oral health on daily performances and its association with clinical variables in a population in Zambia. *International Journal of Dental Hygiene* 2017; **15**: 128–34. |
| 97 |
| Agbor MA, Azodo CC. The impact of dental auxiliaries in oral health delivery in Cameroon. *Annals of African Surgery* 2012; **9**: 84–6. |
| 98 |
| Moosa UK, Bhayat A. The Ergonomic Knowledge and Practice of Dental Students in a Tertiary Institution in South Africa. *International Journal of Dentistry* 2022; **2022**. DOI:[10.1155/2022/4415709](https://doi.org/10.1155/2022/4415709). |
| 99 |
| Anyanechi CE, Saheeb BD. The efficacy of tincture of benzoin compound in the management of extraction sockets of mesio-angularly impacted mandibular third molars. *Oral Surgery* 2013; **6**: 137–41. |
| 100 |
| Menakaya IN, Adegbulugbe IC, Oderinu OH, Shaba OP. The Efficacy of Calcium Hydroxide Powder mixed with 0.2% Chlorhexidine Digluconate or mixed with Normal Saline as Intracanal Medicament in the Treatment of Apical Periodontitis. *J Contemp Dent Pract* 2015; **16**: 657–64. |
| 101 |
| Kemoli AM. The effects of ambient temperature and mixing time of glass ionomer cement material on the survival rate of proximal ART restorations in primary molars. *Contemp Clin Dent* 2014; **5**: 31–6. |
| 102 |
| Lawal FB, Nasiru WO, Taiwo JO. The effectiveness of oral health education conducted at a rural community market setting. *J West Afr Coll Surg* 2013; **3**: 53–69. |
| 103 |
| Kravchenko J, Rango T, Akushevich I, *et al.* The effect of non-fluoride factors on risk of dental fluorosis: Evidence from rural populations of the Main Ethiopian Rift. *Science of the Total Environment* 2014; **488–489**: 595–606. |
| 104 |
| Salem YM, Osman YI. The effect of in-office vital bleaching and patient perception of the shade change. *SADJ* 2011; **66**: 70, 72–6. |
| 105 |
| Suleiman A, Efunkoya A, Omeje K, Amole I. The effect of dental anxiety on surgical time of mandibular third molar disimpaction. *Nigerian Journal of Clinical Practice* 2021; **24**: 1430–7. |
| 106 |
| Umoh AO, Igunma OS, Azodo CC. The effect of chewing stick use on oral hygiene and gingival health of young adults in Nigeria. *Niger J Dent Res* 2020; **5**: 155–60. |
| 107 |
| Seminario AL, Kemoli A, Fuentes W, *et al.* The effect of antiretroviral therapy initiation on vitamin D levels and four oral diseases among Kenyan children and adolescents living with HIV. *PLoS ONE* 2022; **17**. DOI:[10.1371/journal.pone.0275663](https://doi.org/10.1371/journal.pone.0275663). |
| 108 |
| Kemoli AM, van Amerongen WE. The dilemma of selecting suitable proximal carious lesions in primary molars for restoration using ART technique. *Community Dental Health* 2011; **28**: 12–6. |
| 109 |
| Teshome A, Assefa B. The dental complications of canine tooth bud removal in 2-12 years old children in Northwest Ethiopia. *BMC Research Notes* 2019; **12**. DOI:[10.1186/s13104-019-4743-9](https://doi.org/10.1186/s13104-019-4743-9). |
| 110 |
| Uguru C, Umeanuka O, Uguru NP, Adigun O, Edafioghor O. The delusion of halitosis: experience at an eastern Nigerian tertiary hospital. *Nigerian journal of medicine : journal of the National Association of Resident Doctors of Nigeria* 2011; **20**: 236–40. |
| 111 |
| Isiekwe. The COVID-19 pandemic and orthodontic practice in Nigeria. *journal of orthodontic science* 2021; **10**: 5. |
| 112 |
| Charles EA, Birch DS. The clinical presentation and management of zygomatic complex fractures in a Nigeria Teaching Hospital. *Nigerian journal of medicine : journal of the National Association of Resident Doctors of Nigeria* 2012; **21**: 308–12. |
| 113 |
| Sithole PA, Motshabi-Chakane P, Muteba MK. The characteristics and perioperative outcomes of children with orofacial clefts managed at an academic hospital in Johannesburg, South Africa. *BMC Pediatrics* 2022; **22**. DOI:[10.1186/s12887-022-03267-5](https://doi.org/10.1186/s12887-022-03267-5). |
| 114 |
| Avoaka-Boni MC, Djolé SX, Désiré Kaboré WA, D Gnagne-Koffi YN, E Koffi AF. The causes of failure and the longevity of direct coronal restorations: A survey among dental surgeons of the town of Abidjan, Côte d’Ivoire. *J Conserv Dent* 2019; **22**: 270–4. |
| 115 |
| Eigbobo JO, Nzomiwu CL, Etim SS, Amobi EO. The care of traumatic dental injuries in primary schools in Southern Nigeria. *European journal of paediatric dentistry : official journal of European Academy of Paediatric Dentistry* 2015; **16**: 197–200. |
| 116 |
| Kolisa YM, Yengopal V, Shumba K, Igumbor J. The burden of oral conditions among adolescents living with HIV at a clinic in Johannesburg, South Africa. *PLoS ONE* 2019; **14**. DOI:[10.1371/journal.pone.0222568](https://doi.org/10.1371/journal.pone.0222568). |
| 117 |
| Peerbhay F, Barrie RB. The burden of early childhood caries in the Western Cape Public Service in relation to dental general anaesthesia: implications for prevention. *SADJ : journal of the South African Dental Association = tydskrif van die Suid-Afrikaanse Tandheelkundige Vereniging* 2012; **67**: 14–6, 18. |
| 118 |
| Gbolahan OO, Amiede OS, Samuel OA. The Burden and Perceived Stress on Family Caregivers of Patients With Orofacial Cleft Deformities in The Perioperative Period of Cleft Repair. *J Patient Exp* 2020; **7**: 1602–9. |
| 119 |
| Amusa AY, Sulaiman OA. The awareness of dental check-up and the value of aesthetic restoration amongst undergraduates at the University of Ibadan, Southwest Nigeria. *African Journal of Biomedical Research* 2020; **23**: 93–7. |
| 120 |
| Astatkie A, Demissie M, Berhane Y. The association of khat (catha edulis) chewing and orodental health: A systematic review and meta-analysis. *South African Medical Journal* 2014; **104**: 773–9. |
| 121 |
| Gesase N, Miranda-Rius J, Brunet-Llobet L, Lahor-Soler E, Mahande MJ, Masenga G. The association between periodontal disease and adverse pregnancy outcomes in Northern Tanzania: A cross-sectional study. *African Health Sciences* 2018; **18**: 601–11. |
| 122 |
| Anthony OE, Rachel O. The assessment of CD4 lymphocyte counts in patients with chronic periodontitis in Benin City, Nigeria. *Asian Pacific Journal of Tropical Disease* 2012; **2**: S639–41. |
| 123 |
| Muwazi LM, Kamulegeya A. The 5-year prevalence of maxillofacial fibro-osseous lesions in Uganda. *Oral Diseases* 2015; **21**: e79–85. |
| 124 |
| Eweka OM, Ogundana OM, Agbelusi GA. Temporomandibular pain dysfunction syndrome in patients attending lagos university teaching hospital, lagos, nigeria. *J West Afr Coll Surg* 2016; **6**: 70–87. |
| 125 |
| Oyetola EO, Adesina OM, Oluwadaisi A, Adewale A, Adewole O, Anizoba E. Temporomandibular Joint Pain: Clinical Presentations and Response to Conservative Treatments in a Nigerian Tertiary Hospital. *J Int Soc Prev Community Dent* 2017; **7**: 98–103. |
| 126 |
| Agbara R, Fomete B, Obiadazie AC, Idehen K, Okeke U. Temporomandibular joint dislocation: experiences from Zaria, Nigeria. *J Korean Assoc Oral Maxillofac Surg* 2014; **40**: 111–6. |
| 127 |
| Anyanechi CE. Temporomandibular joint ankylosis caused by condylar fractures: A retrospective analysis of cases at an urban teaching hospital in Nigeria. *International Journal of Oral and Maxillofacial Surgery* 2015; **44**: 1027–33. |
| 128 |
| Mekonnen D, Gizaw A, Kebede B. Temporomandibular Joint Ankylosis among Patients at Saint Paul’s Hospital Millennium Medical College, Ethiopia: A 9-Year Retrospective Study. *International Journal of Dentistry* 2021; **2021**. DOI:[10.1155/2021/6695664](https://doi.org/10.1155/2021/6695664). |
| 129 |
| Bankole OO, Lawal FB. Teething: Misconceptions and unhealthy practices among residents of a rural community in Nigeria. *International Quarterly of Community Health Education* 2017; **37**: 99–106. |
| 130 |
| Aliyu I, Adewale A, Teslim LO. Teething myths among nursing mothers in North-Western Nigeria. *Medical Journal of Dr DY Patil University* 2015; **8**: 144–8. |
| 131 |
| Olatunya OS, Adeniyi AT, Babatola AO, *et al.* Teething beliefs, misconceptions, and practices among mothers attending a tertiary hospital in Nigeria: Implications for child health and clinical practice. *Journal of Nepal Paediatric Society* 2020; **40**: 247–54. |
| 132 |
| Adebayo ET, Ahaji LE, Nnachetta RN, *et al.* Technical quality of root canal fillings done in a Nigerian general dental clinic. *BMC Oral Health* 2012; **12**. DOI:[10.1186/1472-6831-12-42](https://doi.org/10.1186/1472-6831-12-42). |
| 133 |
| Enabulele JE, Omo JO. Teaching of dental implantology to undergraduate dental students: The Nigerian experience. *European Journal of Dental Education* 2020; **24**: 476–82. |
| 134 |
| Chetty M, Roomaney IA, Beighton P. Taurodontism in dental genetics. *BDJ Open* 2021; **7**. DOI:[10.1038/s41405-021-00081-6](https://doi.org/10.1038/s41405-021-00081-6). |
| 135 |
| Malele-Kolisa Y, Yengopal V, Igumbor J, Nqcobo CB, Ralephenya TRD. Systematic review of factors influencing oral health-related quality of life in children in Africa. *African Journal of Primary Health Care and Family Medicine* 2019; **11**. DOI:[10.4102/phcfm.v11i1.1943](https://doi.org/10.4102/phcfm.v11i1.1943). |
| 136 |
| Owusu-Afriyie O, Owiredu WKBA, Oti AA, *et al.* Survival rates of head and neck cancers in Ghana: a retrospective study at the Komfo Anokye Teaching Hospital. *BMC Res Notes* 2020; **13**: 392. |
| 137 |
| Asio J, Kamulegeya A, Banura C. Survival and associated factors among patients with oral squamous cell carcinoma (OSCC) in Mulago hospital, Kampala, Uganda. *Cancers of the head & neck* 2018; **3**: 9. |
| 138 |
| Anyanechi C, Chukwuneke F. Survey of the Reasons for Dental Extraction in Eastern Nigeria. *Ann med health sci res (Online)* 2012; **2**: 129–33. |
| 139 |
| Olasoji HO, Hassan A, Adeyemo WL. Survey of management of children with cleft lip and palate in teaching and specialist hospitals in Nigeria. *Cleft Palate-Craniofacial Journal* 2011; **48**: 150–5. |
| 140 |
| Randriamanantena T, Rakotoarison RA, Rakotoarimanana DF, Razafimbahoaka T, Randriamanantenasoa H, Razafindrabe JAB. Surgical treatment for cellulitis of odontogenic origin in the Oral and Maxillofacial Surgery Service of the University Hospital of Antananarivo. *Medecine Buccale Chirurgie Buccale* 2011; **17**: 195–201. |
| 141 |
| Abdurrazaq TO, Micheal AO, Lanre AW, Olugbenga OM, Akin LL. Surgical outcome and complications following cleft lip and palate repair in a teaching hospital in Nigeria. *Afr J Paediatr Surg* 2013; **10**: 345–57. |
| 142 |
| Macigo FG, James RM, Ogunbodede E, Gathece LW. Sugar consumption and dental caries experience in Kenya. *International Dental Journal* 2016; **66**: 158–62. |
| 143 |
| Carneiro LC, Sembiko S, Masalu JR. Subjective prosthodontic treatment need, tooth loss and associated factors among dental patients in Dar es salaam, Tanzania. *African Health Sciences* 2021; **21**: 1905–13. |
| 144 |
| Ogunrinde TJ, Dosumu OO, Shaba OP. Subjective assessment of lingual bar and plate as major connectors in removable partial dentures. *Journal of Stomatology* 2015; **68**: 191–6. |
| 145 |
| Dahlén G, Claesson R, Åberg CH, Haubek D, Johansson A, Kwamin F. Subgingival bacteria in Ghanaian adolescents with or without progression of attachment loss. *Journal of Oral Microbiology* 2014; **6**. DOI:[10.3402/jom.v6.23977](https://doi.org/10.3402/jom.v6.23977). |
| 146 |
| Essobozou PP, Malick N, Evelyne D, *et al.* Sub-mandibular tumors: epidemiological and histological profiles. *The Pan African medical journal* 2014; **18**: 64. |
| 147 |
| Goyal S. Study of palatal rugae pattern of rwandan patients attending the dental department at King Faisal hospital, kigali, Rwanda: A preliminary study. *Rwanda Medical Journal* 2013; **70**: 19–25. |
| 148 |
| Rakotoarison RA, Rakotoarivony AE, Ralaiarimanana FL, Andriambololo-Nivo R, Féki A. Study of dental abnormalities in cleft lip and palate: About 85 cases. *Medecine Buccale Chirurgie Buccale* 2011; **17**: 7–14. |
| 149 |
| Folayan MO, Sofola OO, Khami MR, *et al.* Study motives, career choices and interest in paediatric dentistry among final year dental students in Nigeria. *BMC Medical Education* 2014; **14**. DOI:[10.1186/1472-6920-14-130](https://doi.org/10.1186/1472-6920-14-130). |
| 150 |
| Diouf M, Basse A, Ndiaye M, Cisse D, Lo CM, Faye D. Stroke and periodontal disease in Senegal: Case-control study. *Public Health* 2015; **129**: 1669–73. |
| 151 |
| Amedari MI, Ogunbodede EO, Uti OG, Aborisade AO, Amedari IK. Strengthening the oral health system in Nigeria: A health systems building block approach. *The Nigerian postgraduate medical journal* 2022; **29**: 173–82. |
| 152 |
| Turton M, Naidoo S. Stigma and disclosure as barriers to regular dental care for people living with HIV/AIDS in Kwazulu-Natal and Western Cape, South Africa. *Ethnicity and Inequalities in Health and Social Care* 2014; **7**: 49–59. |
| 153 |
| Enabulele J, Omo J. Sterilization in endodontics: Knowledge, attitude, and practice of dental assistants in training in Nigeria-A cross-sectional study. *Saudi Endodontic Journal* 2018; **8**: 106–10. |
| 154 |
| Sede MA, Audu O, Azodo CC. Stem cells in Dentistry: Knowledge and attitude of Nigerian Dentists. *BMC Oral Health* 2013; **13**. DOI:[10.1186/1472-6831-13-27](https://doi.org/10.1186/1472-6831-13-27). |
| 155 |
| Ministry of Health. Standard guidelines on infection prevention control in dental surgery. 2012. <https://search.bvsalud.org/aimafro/resource/en/biblio-1277991>. |
| 156 |
| Junior TM, Muasya MK, Ngesa JL. Sports-related dentofacial trauma among high school students in nairobi. *East African Medical Journal* 2016; **93**: 671–7. |
| 157 |
| Lawal FB, Oladayo AM. Sources and Factors Related to Oral Health-Care Information Among Dental Patients of a Teaching Hospital in Ibadan, Nigeria. *International Quarterly of Community Health Education* 2019; **40**: 17–22. |
| 158 |
| Okeke UA, Igashi JB, Hamza MA, Ajike SO, Saheeb BD. Sonographic Diagnosis of Metastatic Cervical Lymph Nodes in Primary Orofacial Malignancies: Role of the Radiologist’s Experience. *West Afr J Med* 2021; **38**: 24–7. |
| 159 |
| Ibiyemi O, Taiwo JO. Some socio-demographic attributes as covariates in tooth wear among males in a rural community in Nigeria. *Ethiop J Health Sci* 2012; **22**: 189–95. |
| 160 |
| Firempong C, Nsiah K, Awunyo-Vitor D, Dongsogo J. Soluble fluoride levels in drinking water-a major risk factor of dental fluorosis among children in Bongo community of Ghana. *Ghana medical journal* 2013; **47**: 16–23. |
| 161 |
| Adams S, Lentin R, Hendricks R, Hudson D, Breugem CC. Soft Palate Mucosal Adhesion with Muscle Release: An Option in ‘Wide’ Cleft Palates in a Cohort in Southern Africa. *J Craniofac Surg* 2022; **33**: 1388–93. |
| 162 |
| Konadu AB, Ampofo PC, Hewlett S, Nyako EA, Ndanu TA. Sodium hypochlorite and its use as root canal irrigant: a survey among ghanaian dental practitioners. *Postgrad Med J Ghana* 2018; **7**: 1–7. |
| 163 |
| Kutesa AM, Ndagire B, Nabaggala GS, Mwesigwa CL, Kalyango J, Rwenyonyi CM. Socioeconomic and nutritional factors associated with age of eruption of third molar tooth among Ugandan adolescents. *J Forensic Dent Sci* 2019; **11**: 22–7. |
| 164 |
| Ajayi DM, Abiodun-Solanke IF. Sociobehavioural risk factors of dental caries among selected adolescents in Ibadan, Nigeria. *Pediatric Dental Journal* 2014; **24**: 33–8. |
| 165 |
| Olutola BG, Ayo-Yusuf OA. Socio-environmental factors associated with self-rated oral health in South Africa: A multilevel effects model. *International Journal of Environmental Research and Public Health* 2012; **9**: 3465–83. |
| 166 |
| Masalu JR, Kikwilu EN, Kahabuka FK, Mtaya M, Senkoro AR. Socio-demographic and behavioural correlates of oral health related quality of life among tanzanian adults: A national pathfinder survey. *Tanzania Journal of Health Research* 2012; **14**. DOI:[10.4314/thrb.v14i3.8](https://doi.org/10.4314/thrb.v14i3.8). |
| 167 |
| Kimotho SG, Macharia FN. Social stigma and cultural beliefs associated with cleft lip and/or palate: parental perceptions of their experience in Kenya. *Humanities and Social Sciences Communications* 2020; **7**. DOI:[10.1057/s41599-020-00677-7](https://doi.org/10.1057/s41599-020-00677-7). |
| 168 |
| Oyedele TA, Folayan MO, Chukwumah NM, Onyejaka NK. Social predictors of oral hygiene status in school children from suburban Nigeria. *Brazilian Oral Research* 2019; **33**. DOI:[10.1590/1807-3107BOR-2019.VOL33.0022](https://doi.org/10.1590/1807-3107BOR-2019.VOL33.0022). |
| 169 |
| Ayo-Yusuf IJ, Naidoo S. Social gradient in the cost of oral pain and related dental service utilisation among South African adults. *BMC Oral Health* 2016; **16**. DOI:[10.1186/s12903-016-0313-x](https://doi.org/10.1186/s12903-016-0313-x). |
| 170 |
| Azodo CC, Ogbebor OG. Social distance towards halitosis sufferers. *Swiss Dent J* 2019; **129**: 1026–30. |
| 171 |
| Agbor MA, Azodo CC, Tefouet TSM. Smokeless tobacco use, tooth loss and oral health issues among adults in Cameroon. *African Health Sciences* 2013; **13**: 785–90. |
| 172 |
| Edionwe JI, Shaba OP, Umesi DC. Single visit root canal treatment: a prospective study. *Niger J Clin Pract* 2014; **17**: 276–81. |
| 173 |
| Buchanan GD, Gamieldien1 MY, Tredoux S, Vorster M, Warren N, Van Der Vyfer PJ. Should South Africa train specialist endodontists? A cross-sectional online survey. *Saudi Endodontic Journal* 2021; **11**: 369–74. |
| 174 |
| Govender U, Bhayat A, Rudolph M. Services rendered and barriers faced by public sector oral hygienists in two provinces of South Africa. *SADJ : journal of the South African Dental Association = tydskrif van die Suid-Afrikaanse Tandheelkundige Vereniging* 2013; **68**: 156, 158–60. |
| 175 |
| Adeyemi BF, Kolude B, Lawal AO. Serum Uric Acid Levels in Oral Cancer Patients Seen at Tertiary Institution in Nigeria. *Annals of Ibadan Postgraduate Medicine* 2012; **10**: 9–12. |
| 176 |
| Lawal AO, Kolude B, Adeyemi BF, Lawoyin JO, Akang EE. Serum antioxidant vitamins and the risk of oral cancer in patients seen at a tertiary institution in Nigeria. *Niger J Clin Pract* 2012; **15**: 30–3. |
| 177 |
| Alade GO, Orikpete EV. Self-reported Treatment Needs and Utilization of Dental Services among Dental Students and Dental Technology Students. *International Journal of Prosthodontics and Restorative Dentistry* 2022; **16**: 41–4. |
| 178 |
| Omo JO, Enabulele JE. Self-reported prevalence of missing teeth and unmet prosthetic treatment needs among a population of Nigerian undergraduate students. *Kanem Journal of Medical Sciences* 2020; **17**: 1–9. |
| 179 |
| Siziya S, Muula A, Rudatsikira E. Self-reported poor oral hygiene among in-school adolescents in Zambia. *BMC Research Notes* 2011; **4**. DOI:[10.1186/1756-0500-4-255](https://doi.org/10.1186/1756-0500-4-255). |
| 180 |
| Sorunke ME, Onigbinde OO, Oyapero A, Coker OA. Self-Reported Periodontal Disease and its Association with Dental Anxiety in Lagos, Nigeria. *Pesquisa Brasileira em Odontopediatria e Clinica Integrada* 2022; **22**. DOI:[10.1590/pboci.2022.008](https://doi.org/10.1590/pboci.2022.008). |
| 181 |
| Madiba T, Bhayat A, Nkambule N. Self-reported knowledge, attitude and consumption of sugar-sweetened beverages among undergraduate oral health students at a university in South Africa. *Journal of International Society of Preventive and Community Dentistry* 2017; **7**: S137–42. |
| 182 |
| Kayombo CM, Mumghamba EG. Self-Reported Halitosis in relation to Oral Hygiene Practices, Oral Health Status, General Health Problems, and Multifactorial Characteristics among Workers in Ilala and Temeke Municipals, Tanzania. *International Journal of Dentistry* 2017; **2017**. DOI:[10.1155/2017/8682010](https://doi.org/10.1155/2017/8682010). |
| 183 |
| Alade O, Ajoloko E, Dedeke A, Uti O, Sofola O. Self-reported halitosis and oral health related quality of life in adolescent students from a suburban community in Nigeria. *African Health Sciences* 2020; **20**: 2044–9. |
| 184 |
| Adeniyi AA, Odusanya OO. Self-reported dental pain and dental caries among 8-12-year-old school children: An exploratory survey in Lagos, Nigeria. *The Nigerian postgraduate medical journal* 2017; **24**: 37–43. |
| 185 |
| Lawal FB, Dosumu EB. Self-reported and clinically evident gingival bleeding and impact on oral health-related quality of life in young adolescents: A comparative study. *Malawi Medical Journal* 2021; **33**: 121–6. |
| 186 |
| Olusile AO, Adeniyi AA, Orebanjo O. Self-rated oral health status, oral health service utilization, and oral hygiene practices among adult Nigerians. *BMC Oral Health* 2014; **14**. DOI:[10.1186/1472-6831-14-140](https://doi.org/10.1186/1472-6831-14-140). |
| 187 |
| Pengpid S, Peltzer K. Self-rated oral health status and social and health determinants among community dwelling adults in Kenya. *African Health Sciences* 2019; **19**: 3146–53. |
| 188 |
| Aikins EA, Dacosta OO, Onyeaso CO, Isiekwe MC. Self-perception of malocclusion among Nigerian adolescents using the aesthetic component of the IOTN. *Open Dentistry Journal* 2012; **6**: 61–6. |
| 189 |
| Isiekwe GI, Aikins EA. Self-perception of dental appearance and aesthetics in a student population. *International Orthodontics* 2019; **17**: 506–12. |
| 190 |
| Idowu EA, Afolabi AO, Fakuade BO, Akintububo OB, Ibiyemi O. Self-medication profile of dental patients attending a north eastern tertiary hospital in nigeria. *Ann Ib Postgrad Med* 2019; **17**: 173–80. |
| 191 |
| Kaboré WAD, Ouédraogo CDW, Konaté A, *et al.* Self-medication for oral diseases in the city of Ouagadougou, Burkina Faso. *Medecine Buccale Chirurgie Buccale* 2016; **22**: 277–84. |
| 192 |
| Azodo C, Ogbomo A. Self-Evaluated Dental Appearance Satisfaction among Young Adults. *Ann Med Health Sci Res* 2014; **4**: 603–7. |
| 193 |
| Braimah RO, Ukpong DI, Ndukwe KC, Akinyoola L. Self-esteem following maxillofacial and orthopedic injuries: preliminary observations in sub-Saharan Africans. *Oral and Maxillofacial Surgery* 2019; **23**: 71–6. |
| 194 |
| Nwhator SO, Olojede CO, Ijarogbe O, Agbaje MO. Self-assessed dental health knowledge of Nigerian doctors. *East African Medical Journal* 2013; **90**: 147–55. |
| 195 |
| Gbadebo SO, Ajayi DM. Self reported tooth discolorations among patients seen at dental center university college hospital ibadan. *J West Afr Coll Surg* 2015; **5**: 66–77. |
| 196 |
| Lawal FB, Olawole WO, Sigbeku OF. Self rating of oral health status by student dental surgeon assistants in ibadan, Nigeria - a pilot survey. *Ann Ib Postgrad Med* 2013; **11**: 12–7. |
| 197 |
| Agbor MA, Azodo CC. Self medication for oral health problems in Cameroon. *International Dental Journal* 2011; **61**: 204–9. |
| 198 |
| Ogunsuji OO, Dosumu EB, Dairo MD, Ogunsuji AI. Self assessment of oral health and risk factors affecting oral hygiene status in adolescents attending dental clinic in university college hospital, ibadan. *Ann Ib Postgrad Med* 2021; **19**: 70–7. |
| 199 |
| Sofianos C, Christofides EA, Phiri SE. Seasonal Variation of Orofacial Clefts. *Journal of Craniofacial Surgery* 2018; **29**: 368–71. |
| 200 |
| Folayan MO, Haire BG, Adeniyi AA, Adeyemo WL. Screening Children for Caries: An Ethical Dilemma in Nigeria. *New Bioethics* 2018; **24**: 135–49. |
| 201 |
| Lawal FB, Oke GA. Satisfaction with dental condition and oral health-related quality of life of school-age children with dental pain in Ibadan, Nigeria. *SAGE Open Med* 2021; **9**: 20503121211025944. |
| 202 |
| Enabulele JE, Adayonfo EO. Satisfaction with dental appearance and personality traitsamong a population of Nigerian dental patients. *The international journal of esthetic dentistry* 2019; **14**: 64–75. |
| 203 |
| Orhue VE, Ehizele AO, Akhionbare O, Ojehanon P. Salivary lactoferrin levels, disease severity and correlates in patients with chronic periodontitis presenting to a tertiary health facility in Nigeria. *Niger J Dent Res (Online)* 2022; **7**: 60–6. |
| 204 |
| Olayanju OA, Rahamon SK, Joseph IO, Arinola OG. Salivary immunoglobulin classes in Nigerian smokers with periodontitis. *World J Biol Chem* 2012; **3**: 180–3. |
| 205 |
| Parkins GE, Blankson PK, Affum A, Boamah MO, Sackeyfio J. Salivary gland neoplasms: A 10-year review of a major referral center in Ghana. *Oral Surg Oral Med Oral Pathol Oral Radiol* 2021; **131**: 161–5. |
| 206 |
| Emeke U, Obontu TJ, Olushola I, Akinyele A. Salivary Fluoride retention: A comparative analysis between fluoride containing chewing sticks and a non herbal fluoridated toothpaste. *Journal of Contemporary Dental Practice* 2019; **20**: 370–6. |
| 207 |
| Oyetola EO, Owotade FJ, Agbelusi GA, Fatusi O, Sanusi A, Adesina OM. Salivary Flow Rates of Nigerian Patients with Chronic Kidney Disease: A Case-control Study. *Journal of Contemporary Dental Practice* 2015; **16**: 264–9. |
| 208 |
| Oyetola EO, Awosusi OO, Agho ET, Abdullahi MAS, Suleiman IK, Egunjobi S. Salivary bacterial count and its implications on the prevalence of oral conditions. *Journal of Contemporary Dental Practice* 2019; **20**: 184–9. |
| 209 |
| Omondi BI, Ocholla TJ. Routine radiographic findings in clinically healthy edentulous jaw bones of patients seeking their first set of complete denture prostheses. *East Afr Med J* 2012; **89**: 258–62. |
| 210 |
| Oginni AO, Adeleke AA, Chandler NP. Root canal treatment and prevalence of apical periodontitis in a Nigerian adult subpopulation: A radiographic study. *Oral Health and Preventive Dentistry* 2015; **13**: 85–90. |
| 211 |
| Buchanan GD, Gamieldien MY, Fabris-Rotelli I, van Schoor A, Uys A. Root and canal morphology of the permanent anterior dentition in a Black South African population using cone-beam computed tomography and two classification systems. *Journal of Oral Science* 2022; **64**: 218–23. |
| 212 |
| Taiwo OO, Panas R. Roles of community pharmacists in improving oral health awareness in Plateau State, Northern Nigeria. *International Dental Journal* 2018; **68**: 287–94. |
| 213 |
| Osunde OD, Bassey GO. Role of warm saline mouth rinse in prevention of alveolar osteitis: a randomized controlled trial. *Niger J Med* 2015; **24**: 28–31. |
| 214 |
| Obimakinde OS, Okoje VN, Ijarogbe OA, Obimakinde AM. Role of Patients’ Demographic Characteristics and Spatial Orientation in Predicting Operative Difficulty of Impacted Mandibular Third Molar. *Ann med health sci res (Online)* 2013; **3**: 81–4. |
| 215 |
| Gadkaree SK, Tollefson TT, Fuller JC, Muchemwa FC, Gonga A, Shaye DA. Role of mobile health on patient enrollment for cleft lip-palate surgery: A comparative study using SMS blast text messaging in zimbabwe. *Laryngoscope Investigative Otolaryngology* 2019; **4**: 383–6. |
| 216 |
| Sid-Dick FM, Sidikiba S, Fapeingou TA, Badara NA, Tiranké K, Mouctar DA. Risques traumatiques alvéolo-dentaires des enfants présentant la proalvéolie. *African Journal of Dentistry and Implantology* 2020; **17**: 7–13. |
| 217 |
| Folayan MO, Adeniyi AA, Arowolo O, Maureen CN, Alade MA, Tantawi ME. Risk indicators for dental caries, and gingivitis among 6–11-year-old children in Nigeria: a household-based survey. *BMC Oral Health* 2022; **22**. DOI:[10.1186/s12903-022-02470-1](https://doi.org/10.1186/s12903-022-02470-1). |
| 218 |
| Onyejaka NK, Amobi EO. Risk factors of early childhood caries among children in Enugu, Nigeria. *Pesquisa Brasileira em Odontopediatria e Clinica Integrada* 2016; **16**: 381–91. |
| 219 |
| Farley E, Lenglet A, Ariti C, *et al.* Risk factors for diagnosed noma in northwest Nigeria: A case-control study, 2017. *PLoS Negl Trop Dis* 2018; **12**: e0006631. |
| 220 |
| Oginni AO, Adeleke AA, Mejabi MO, Sotunde OA. Risk Factors for Apical Periodontitis Sub-Urban Adult Population. *Niger Postgrad Med J* 2015; **22**: 105–9. |
| 221 |
| Buckle GC, Mmbaga EJ, Paciorek A, *et al.* Risk Factors Associated With Early-Onset Esophageal Cancer in Tanzania. *JCO Global Oncology* 2022. DOI:[10.1200/GO.21.00256](https://doi.org/10.1200/GO.21.00256). |
| 222 |
| Adesina OM, Soyele OO, Oyetola EO, Fatusi OA. Review of 109 cases of primary malignant orofacial lesions seen at a Nigerian Tertiary Hospital. *The Nigerian postgraduate medical journal* 2018; **25**: 246–51. |
| 223 |
| White MC, Horner KC, Lai PS. Retrospective review of the anaesthetic management of maxillectomies and mandibulectomies for benign tumours in sub-Saharan Africa. *PLoS ONE* 2016; **11**. DOI:[10.1371/journal.pone.0165090](https://doi.org/10.1371/journal.pone.0165090). |
| 224 |
| Obimakinde OS, Okoje VN, Akinmoladun VI, Fasola AO, Arotiba JT. Retrospective evaluation of necrotizing fasciitis in University College Hospital, Ibadan. *Nigerian Journal of Clinical Practice* 2012; **15**: 344–8. |
| 225 |
| Enabulele J, Ibhawoh L. Resident obstetricians’ awareness of the oral health component in management of nausea and vomiting in pregnancy. *BMC Pregnancy and Childbirth* 2014; **14**. DOI:[10.1186/s12884-014-0388-9](https://doi.org/10.1186/s12884-014-0388-9). |
| 226 |
| Olatosi OO, Li M, Alade AA, *et al.* Replication of GWAS significant loci in a sub-Saharan African Cohort with early childhood caries: a pilot study. *BMC Oral Health* 2021; **21**. DOI:[10.1186/s12903-021-01623-y](https://doi.org/10.1186/s12903-021-01623-y). |
| 227 |
| Umeizudike KA, Ayanbadejo PO, Umeizudike TI, Isiekwe GI, Savage KO. Relevance of routine blood pressure assessment among dental patients in Lagos, Nigeria. *Journal of Contemporary Dental Practice* 2013; **14**: 1145–50. |
| 228 |
| Diouf JS, Ouedraogo Y, Seck K, *et al.* [Relationships between the size of the adenoids and the dental arch measurements]. *Orthod Fr* 2018; **89**: 411–20. |
| 229 |
| Okoh M, Saheeb B, Agbelusi G, Omoregie F. Relationships between CD4+ Counts and the Presence of Oral Lesions in Human Immunodeficiency Virus Positive Women in Nigeria. *Ann Med Health Sci Res* 2014; **4**: 572–7. |
| 230 |
| Ehizele AO, Ojehanon PI. Relationship Between the Concentration of Volatile Sulphur Compound and Periodontal Disease Severity in Nigerian Young Adults. *Niger med j (Online)* 2013; **54**: 149–52. |
| 231 |
| Onyejaka NK, Eboh OF, Amobi EO, Nwamba NP. Relationship between socio-demographic profile, parity and dental caries among a group of nursing mothers in south east, Nigeria. *Pesquisa Brasileira em Odontopediatria e Clinica Integrada* 2020; **21**: 1–8. |
| 232 |
| Adeyemi TE, Otuyemi OD. Relationship between Playing of Wind Musical Instruments and Symptoms of Temporomandibular Joint Disorders in a Male Nigerian Adult Population. *West Afr J Med* 2019; **36**: 262–6. |
| 233 |
| Ngoude JXE, Moor VJA, Nadia-Flore TT, *et al.* Relationship between periodontal diseases and newly-diagnosed metabolic syndrome components in a sub-Saharan population: a cross sectional study. *BMC oral health* 2021; **21**: 326. |
| 234 |
| Sede MA, Ehizele AO. Relationship between obesity and oral diseases. *Nigerian Journal of Clinical Practice* 2014; **17**: 683–90. |
| 235 |
| Soyoye OA, Otuyemi OD, Kolawole KA, Ayoola OO. Relationship between masseter muscle thickness and maxillofacial morphology in pre-orthodontic treatment patients. *Int Orthod* 2018; **16**: 698–711. |
| 236 |
| Diomande M, Beugre J-B, Koueita MKK, Vaysse F. Relationship between Angular Measurements and Facial Shape of Young Ivorians with Normal Dental Occlusion. *Scientific World Journal* 2018; **2018**. DOI:[10.1155/2018/6395910](https://doi.org/10.1155/2018/6395910). |
| 237 |
| Peters A, Brandt K, Wienke A, Schaller H-G. Regional Disparities in Caries Experience and Associating Factors of Ghanaian Children Aged 3 to 13 Years in Urban Accra and Rural Kpando. *International Journal of Environmental Research and Public Health* 2022; **19**. DOI:[10.3390/ijerph19095771](https://doi.org/10.3390/ijerph19095771). |
| 238 |
| Nwoga M. Recurrent tumours of ameloblastoma: Clinicopathologic features and diagnostic outcome. *Nigerian Journal of Clinical Practice* 2022; **25**: 1529–34. |
| 239 |
| Nwoga MC. Recurrent tumors of ameloblastoma: Clinicopathologic features and diagnostic outcome. *Niger J Clin Pract* 2022; **25**: 1771–7. |
| 240 |
| Aluko-Olokun B, Olaitan AA, Aluko-Olokun OA. Reconstruction of tooth-bearing portion of mandible using polyglactin 910 sutures for internal fixation in the third-world: functional and cosmetic outcome. *Oral Maxillofac Surg* 2017; **21**: 13–20. |
| 241 |
| Ndukwe KC, Aregbesola SB, Ikem IC, *et al.* Reconstruction of mandibular defects using nonvascularized autogenous bone graft in nigerians. *Niger J Surg* 2014; **20**: 87–91. |
| 242 |
| Craig L, Lutz A, Berry KA, Yang W. Recommendations for fluoride limits in drinking water based on estimated daily fluoride intake in the Upper East Region, Ghana. *Science of the Total Environment* 2015; **532**: 127–37. |
| 243 |
| Anyanechi CE, Saheeb BD. Reasons underlying failure to seek early dental treatment among patients presenting in a Nigeria Tertiary Hospital. *Journal of Medicine and Biomedical Research* 2013; **12**: 37–45. |
| 244 |
| Ogbebor OGA Clement Chinedu. Reasons for Seeking Dental Healthcare Services in a Nigerian Missionary Hospital. *Sahel medical journal (Print)* 2016; **19**: 38–43. |
| 245 |
| Osunde OD, Efunkoya AA, Omeje KU. Reasons for loss of the permanent teeth in patients in kano, north western nigeria. *J West Afr Coll Surg* 2017; **7**: 47–64. |
| 246 |
| Sachedina T, Sohal KS, Owibingire SS, Hamza OJM. Reasons for Delay in Seeking Treatment for Dental Caries in Tanzania. *International Dental Journal* 2022. DOI:[10.1016/j.identj.2022.07.012](https://doi.org/10.1016/j.identj.2022.07.012). |
| 247 |
| Saheeb BD, Sede MA. Reasons and pattern of tooth mortality in a Nigerian Urban teaching hospital. *Annals of African Medicine* 2013; **12**: 110–4. |
| 248 |
| Taiwo OA, Alabi OA, Yusuf OM, Ololo O, Olawole WO, Adeyemo WI. Reasons and pattern of tooth extraction among patients presenting at a Nigerian semi-rural specialist hospital. *Nigerian quarterly journal of hospital medicine* 2012; **22**: 200–4. |
| 249 |
| Adeosun OO, Ogah SA. Rare craniofacial cleft in a dark-skinned African population. *Niger Med J* 2017; **58**: 21–5. |
| 250 |
| Kamulegeya A, Okello SM. Ranulas: possible signs for HIV/AIDS? 1 year Ugandan descriptive study. *Acta Odontol Scand* 2012; **70**: 149–53. |
| 251 |
| Gbolahan OO, Osinaike BB, Udoye CI, Olawole OW. Range of mouth opening among three major ethnic groups in nigeria. *Ann Ib Postgrad Med* 2019; **17**: 130–7. |
| 252 |
| Azuoru MO, Ashiwaju MO, Edomwonyi A, Oyapero A, Obisesan B, Omotuyole A. Randomized controlled trial on the effectiveness of silver diamine fluoride in arresting caries in Lagos, Nigeria. *Brazilian Journal of Oral Sciences* 2022; **21**. DOI:[10.20396/bjos.v21i00.8666341](https://doi.org/10.20396/bjos.v21i00.8666341). |
| 253 |
| Gbolahan OO, Ogunmuyiwa SA, Osinaike BB. Randomized Controlled Trial comparing Dressing and No Dressing of Surgical Wound after Cleft Lip Repair. *J Contemp Dent Pract* 2015; **16**: 554–8. |
| 254 |
| Bane K, Charpentier E, Bronnec F, *et al.* Randomized Clinical Trial of Intraosseous Methylprednisolone Injection for Acute Pulpitis Pain. *Journal of Endodontics* 2016; **42**: 2–7. |
| 255 |
| Gbadebo OS, Ajayi DM, Oyekunle OO, Shaba PO. Randomized clinical study comparing metallic and glass fiber post in restoration of endodontically treated teeth. *Indian J Dent Res* 2014; **25**: 58–63. |
| 256 |
| Ibhawoh LO, Enyinnaya NS, Enabulele JE. Radiographic assessment of endodontic accidents at a Nigerian tertiary health institution: a one-year retrospective study. *Ibom Medical Journal* 2020; **13**: 206–13. |
| 257 |
| Jibiri NN, Adeleye B, Kolude B. Radiation dose to the thyroid, eyes and parotid glands of patients undergoing intra-oral radiographic procedures in a teaching hospital in Ibadan, Oyo state Nigeria. *International Journal of Radiation Research* 2017; **15**: 101–6. |
| 258 |
| Azodo CC, Osahon OD. Quantitative and qualitative analysis of relative saliva viscosity among carious and non-carious young adults. *Niger J Dent Res* 2020; **5**: 131–5. |
| 259 |
| Lawal HS, Adebola RA, Arotiba JT, *et al.* Quality of life of patients surgically treated for ameloblastoma. *Niger Med J* 2016; **57**: 91–8. |
| 260 |
| Omeje KU, Rana M, Adebola AR, *et al.* Quality of life in treatment of mandibular fractures using closed reduction and maxillomandibular fixation in comparison with open reduction and internal fixation--a randomized prospective study. *J Craniomaxillofac Surg* 2014; **42**: 1821–6. |
| 261 |
| Efunkoya AA, Adebola RA, Omeje KU, Amole IO, Akhiwu BI, Osunde DO. Quality of life following surgical treatment of oral cancers. *J Korean Assoc Oral Maxillofac Surg* 2015; **41**: 19–25. |
| 262 |
| Bankole OO, Taiwo JO. Quality of care at a pediatric dental clinic in Ibadan, Nigeria. *Nigerian Journal of Clinical Practice* 2013; **16**: 5–11. |
| 263 |
| Ibiyemi O, Ibiyemi T. Quality and contents of referral letters from peripheral health centers to the dental centre of a teaching hospital, southwestern Nigeria. *Acta Odontologica Scandinavica* 2012; **70**: 165–8. |
| 264 |
| Nabirye RC, Kamulegeya A. Public knowledge about oral cancer in Uganda: a free dental camp experience. *Journal of Health Research* 2019; **33**: 270–9. |
| 265 |
| Ramnarain P, Singh S. Public healthcare practitioners’ knowledge, attitudes and practices related to oral antibiotic prescriptions for dental use in Pietermaritzburg, KwaZulu-Natal. *Health SA* 2022; **27**: 1832. |
| 266 |
| Folayan MO, El Tantawi M, Oginni A, Adeniyi A, Alade M, Finlayson TL. Psychosocial, education, economic factors, decision-making ability, and caries status of mothers of children younger than 6 years in suburban Nigeria. *BMC Oral Health* 2020; **20**. DOI:[10.1186/s12903-020-01120-8](https://doi.org/10.1186/s12903-020-01120-8). |
| 267 |
| Fadeyibi IO, Coker OA, Zacchariah MP, Fasawe A, Ademiluyi SA. Psychosocial effects of cleft lip and palate on Nigerians: The Ikeja-Lagos experience. *Journal of Plastic Surgery and Hand Surgery* 2012; **46**: 13–8. |
| 268 |
| Ibiyemi O, Taiwo JO. Psychosocial Aspect of Anterior Tooth Discoloration among Adolescents in Igbo-Ora; Southwestern Nigeria. *Annals of Ibadan Postgraduate Medicine* 2012; **9**: 94–9. |
| 269 |
| Ibiyemi O, Taiwo JO. Psychosocial aspect of anterior tooth discoloration among adolescents in igbo-ora, southwestern Nigeria. *Ann Ib Postgrad Med* 2011; **9**: 94–9. |
| 270 |
| Fashina AA, Aina OF, Adeyemo WL, Ladeinde A. Psychological Distress Before and After Surgical Resection of Benign Orofacial Tumors: A Prospective Study. *J Oral Maxillofac Surg* 2020; **78**: 1654.e1-1654.e7. |
| 271 |
| Vered Y, Soskolne V, Zini A, Livny A, Sgan-Cohen HD. Psychological distress and social support are determinants of changing oral health status among an immigrant population from Ethiopia. *Community Dentistry and Oral Epidemiology* 2011; **39**: 145–53. |
| 272 |
| Erinfolami AR, Olagunju AT, Oshodi YO, Akinbode AA, Fadipe B, Adeyemo WL. Psychological Distress and Emotional Pain Among Adult Attendees of a Dental Clinic: A Case-Control Study. *Ment Illn* 2016; **8**: 6006. |
| 273 |
| Mbodj EB, Diouf M, Faye D, *et al.* Prosthetic rehabilitation: Needs in Senegalese dental offices. *Bulletin de la Societe de Pathologie Exotique* 2011; **104**: 355–6. |
| 274 |
| Omo J, Sede M, Enabulele J. Prosthetic rehabilitation of patients with maxillary defects in a nigerian tertiary hospital. *Ann Med Health Sci Res* 2014; **4**: 630–3. |
| 275 |
| Omeje KU, Adebola AR, Efunkoya AA, *et al.* Prospective study of the quality of life after treatment of mandibular fractures. *Br J Oral Maxillofac Surg* 2015; **53**: 342–6. |
| 276 |
| Olusanya AA, Arotiba JT, Fasola OA, Akadiri AO. Prophylaxis versus pre-emptive antibiotics in third molar surgery: a randomised control study. *Niger Postgrad Med J* 2011; **18**: 105–10. |
| 277 |
| Anyanechi CE, Chukwuneke FN. Prognosis of teeth in the line of mandibular fracture: 5-year clinical and radiological follow-up. *Nigerian journal of medicine : journal of the National Association of Resident Doctors of Nigeria* 2013; **22**: 61–3. |
| 278 |
| Yadufashije C, Uwase D, Muhimpundu L, *et al.* Profiles of sugar fermenting bacteria of the oral cavity among children with dental caries attending stomatology services at Ruhengeri referral hospital in Musanze District, Northern Rwanda. *The Nigerian postgraduate medical journal* 2022; **29**: 236–43. |
| 279 |
| Singh PK, Combrinck M. Profile of the dental therapy graduate at the University of KwaZulu-Natal. *SADJ : journal of the South African Dental Association = tydskrif van die Suid-Afrikaanse Tandheelkundige Vereniging* 2011; **66**: 468, 470–4. |
| 280 |
| Benoist HM, Seck-Diallo A, Diouf A, Yabbre S, Sembene M, Diallo PD. Profile of chronic and aggressive periodontitis among Senegalese. *J Periodontal Implant Sci* 2011; **41**: 279–84. |
| 281 |
| Ayo-Yusuf OA, Booyens S. Principal motives for toothbrushing in a population of South African adolescents: implications for oral health promotion. *SADJ : journal of the South African Dental Association = tydskrif van die Suid-Afrikaanse Tandheelkundige Vereniging* 2011; **66**: 174–8. |
| 282 |
| Akpeh JO, Okechi UC, Ezeanolue BC. Primary minor salivary gland tumors: A retrospective review of cases seen in a tertiary institution in South East Nigeria. *Niger J Clin Pract* 2022; **25**: 368–72. |
| 283 |
| Folayan MO, Khami MR, Onyejaka N, Popoola BO, Adeyemo YI. Preventive oral health practices of school pupils in Southern Nigeria. *BMC Oral Health* 2014; **14**. DOI:[10.1186/1472-6831-14-83](https://doi.org/10.1186/1472-6831-14-83). |
| 284 |
| Olatosi OO, Sote EO, Akinsola OJ, Oredugba FA, Adenaike AS. Prevention of dental caries: Knowledge, practice and opinion of paediatricians in Lagos. *West African Journal of Medicine* 2013; **32**: 52–6. |
| 285 |
| Osunde OD, Anyanechi CE, Bassey GO. Prevention of alveolar osteitis after third molar surgery: Comparative study of the effect of warm saline and chlorhexidine mouth rinses. *Nigerian Journal of Clinical Practice* 2017; **20**: 470–3. |
| 286 |
| Idon PI, Enabulele JE. Prevalence, severity, and request for treatment of dental fluorosis among adults in an endemic region of Northern Nigeria. *European Journal of Dentistry* 2018; **12**: 184–90. |
| 287 |
| Ndagire B, Kutesa A, Ssenyonga R, Kiiza HM, Nakanjako D, Rwenyonyi CM. Prevalence, severity and factors associated with dental caries among school adolescents in Uganda: A cross-sectional study. *Brazilian Dental Journal* 2020; **31**: 171–8. |
| 288 |
| Oyedele TA, Folayan MO, Adekoya-Sofowora CA, Oziegbe EO, Esan TA. Prevalence, pattern and severity of molar incisor hypomineralisation in 8- to 10-year-old school children in Ile-Ife, Nigeria. *European Archives of Paediatric Dentistry* 2015; **16**: 277–82. |
| 289 |
| Kesande T, Muwazi LM, Bataringaya A, Rwenyonyi CM. Prevalence, pattern and perceptions of cleft lip and cleft palate among children born in two hospitals in Kisoro District, Uganda. *BMC Oral Health* 2014; **14**: 1–7. |
| 290 |
| Arubaku W, Kwizera G, Tusubira D, *et al.* Prevalence, correlates and treatment needs of dental caries among people on antiretroviral therapy in Uganda: a cross sectional study. *BMC Oral Health* 2022; **22**. DOI:[10.1186/s12903-022-02256-5](https://doi.org/10.1186/s12903-022-02256-5). |
| 291 |
| Folayan MO, Kolawole KA, Oziegbe EO, *et al.* Prevalence, and early childhood caries risk indicators in preschool children in suburban Nigeria. *BMC Oral Health* 2015; **15**. DOI:[10.1186/s12903-015-0058-y](https://doi.org/10.1186/s12903-015-0058-y). |
| 292 |
| Ogbebor OG, Obisesan B, Madukwe IU, Azodo CC. Prevalence of undiagnosed HIV infection among dental patients in a Nigerian secondary healthcare facility. *Journal of International Society of Preventive & Community Dentistry* 2015; **5**: 237–41. |
| 293 |
| Ogordi PU, Ize-Iyamu IN, Adeniyi EO. Prevalence of traumatic dental injury to the anterior teeth in children attending paramilitary and nonparamilitary schools in Nigeria. *Annals of African Medicine* 2019; **18**: 80–5. |
| 294 |
| Kane A, Diawara O, Niang A, *et al.* [Prevalence Of Tooth Decay In Children From Age 3 To 14 At The Department Of Odontology Of Militairy Hopital Of Bamako (IHB) In Mali]. *Mali Med* 2018; **33**: 31–5. |
| 295 |
| Sehdev B, Muruts L, Ganji KK. Prevalence of tooth decay and associated factors among ethiopian patients. *Pesquisa Brasileira em Odontopediatria e Clinica Integrada* 2020; **20**: 1–7. |
| 296 |
| Ehizele AO, Azodo CC, Ojehanon PI, Akhionbare O, Umoh AO, Adeghe HA. Prevalence of tobacco use among dental patients and their knowledge of its health effects. *Nigerian Journal of Clinical Practice* 2012; **15**: 270–5. |
| 297 |
| Bayingana C, Mambo Muvunyi C, Musemakweli A, Muyizi C, Ngoga E. Prevalence of six Periodental Pathogens in Rwandan Women’s Gingival Crevicular Fluide. *Rwanda med j (Online)* 2012; **69**: 5–10. |
| 298 |
| Oderinu OH, Savage KO, Uti OG, Adegbulugbe IC. Prevalence of self-reported hypersensitive teeth among a group of Nigerian undergraduate students. *The Nigerian postgraduate medical journal* 2011; **18**: 205–9. |
| 299 |
| Butali A, Adeyemo WL, Mossey PA, *et al.* Prevalence of orofacial clefts in Nigeria. *Cleft Palate-Craniofacial Journal* 2014; **51**: 320–5. |
| 300 |
| Mulder R, Mohamed N, Mathiba O. Prevalence of oral mucosal lesions in human immunodeficiency virus-infected children attending the Pediatric Infectious Diseases Clinic in Cape Town. *Clinical and Experimental Dental Research* 2022; **8**: 160–8. |
| 301 |
| Pontes CC, Chikte U, Kimmie-dhansay F, Erasmus RT, Kengne AP, Matsha TE. Prevalence of oral mucosal lesions and relation to serum cotinine levels—findings from a cross- sectional study in South Africa. *International Journal of Environmental Research and Public Health* 2020; **17**. DOI:[10.3390/ijerph17031065](https://doi.org/10.3390/ijerph17031065). |
| 302 |
| Akande AJ, Uti OG, Sofola OO. Prevalence of oral mucosal lesions and oral health related quality of life among adolescents in a rural Nigerian population. *Niger J Dent Res (Online)* 2022; **7**: 10–9. |
| 303 |
| Blankson P-K, Amoah G, Thadani M, *et al.* Prevalence of oral conditions and associated factors among schoolchildren in Accra, Ghana: a cross-sectional study. *International Dental Journal* 2022; **72**: 93–9. |
| 304 |
| Davidson CL, Richter KL, van Der Linde MD, Coetsee J, Boy SC. Prevalence of oral and oropharyngeal human papillomavirus in a sample of South African men: A pilot study. *South African Medical Journal* 2014; **104**: 358–61. |
| 305 |
| Tefera AT, Bekele BG, Derese K, Andualem G. Prevalence of occlusal features and their relation to sociodemographic variables in northwest ethiopia: A cross-sectional study. *Clinical, Cosmetic and Investigational Dentistry* 2021; **13**: 459–68. |
| 306 |
| Diouf M, Cisse D, Faye A, *et al.* Prevalence of necrotizing ulcerative gingivitis and associated factors in Koranic boarding schools in Senegal. *Community Dental Health* 2012; **29**: 184–7. |
| 307 |
| Otayto K, Godana W, Feleke T, Hussen S, Alemu M. Prevalence of Milk Teeth Extraction and Enabling Community Factors Among Under Five-Year-Old Children in Alle Special Woreda, SNNPR, Ethiopia, 2022: Community-Based Cross-Sectional Study; Based on Theory of Planned Behavior Model. *Pediatric Health Med Ther* 2022; **13**: 257–69. |
| 308 |
| Maduakor SN, Nwoga MC. Prevalence of mandibular and palatine tori among the Ibos in Enugu, South-East Nigeria. *Nigerian Journal of Clinical Practice* 2017; **20**: 57–60. |
| 309 |
| Goyal S. Prevalence of malocclusion in Rwandan people in a hospital-based study. *Rwanda Medical Journal* 2018; **75**: 1–8. |
| 310 |
| Adekoya MN, Ayedun OS, Adeyemi TE. Prevalence of Malocclusion in Children between the Age of 10-15 Years in Calabar Metropolis, Cross River. *West Afr J Med* 2021; **Vol. 38**: 1095–100. |
| 311 |
| Aikins EA, Onyeaso CO. Prevalence of malocclusion and occlusal traits among adolescents and young adults in Rivers State, Nigeria. *Odonto-stomatologie tropicale = Tropical dental journal* 2014; **37**: 5–12. |
| 312 |
| Mtaya M, Brudvik P, Astrom AN. Prevalence of malocclusion and its associated factors among preschoolchildren in Kinondoni and Temeke Districts, Tanzania. *Tanzania Journal of Health Research* 2017; **19**. DOI:[10.4314/thrb.v19i2.7](https://doi.org/10.4314/thrb.v19i2.7). |
| 313 |
| Haubek D, Mulli T, Kemoli A, *et al.* Prevalence of JP2 and non-JP2 genotypes of aggregatibacter actinomycetemcomitans and oral hygiene practice of kenyan adolescents in Maasai Mara. *Pathogens* 2021; **10**. DOI:[10.3390/pathogens10040488](https://doi.org/10.3390/pathogens10040488). |
| 314 |
| Aikins EA, Ututu C, Chukwuma EI. Prevalence of incidental dental anomalies seen on pre-treatment digital panoramic radiographs of a group of Nigerian orthodontic patients: A retrospective study. *Niger J Dent Res (Online)* 2022; **7**: 67–74. |
| 315 |
| Anyasodor AE, Nwose EU, Bwititi PT, *et al.* Prevalence of hyperglycemia and risk factors for orodental disease in Nigeria: Implications of opportunistic screening. *Indian Journal of Dental Research* 2017; **28**: 507–13. |
| 316 |
| Mary EO, Abiola OA, Titilola G, Mojirayo OO, Sulaimon AA. Prevalence of HIV related oral lesions in people living with HIV and on combined antiretroviral therapy: A Nigerian experience. *Pan African Medical Journal* 2018; **31**. DOI:[10.11604/pamj.2018.31.180.13574](https://doi.org/10.11604/pamj.2018.31.180.13574). |
| 317 |
| Mbodj EB, Faye B, Faye D, *et al.* [Prevalence of halitosis in patients with dental prostheses in Senegal]. *Med Trop (Mars)* 2011; **71**: 272–4. |
| 318 |
| Soroye MO, Ayanbadejo PO. Prevalence of gingivitis and perception of gingival colour among pregnant women attending the antenatal clinic of Lagos University Teaching Hospital, Idi-Araba. *Journal of Orofacial Sciences* 2016; **8**: 53–8. |
| 319 |
| Ndekero TS, Carneiro LC, Masumo RM. Prevalence of early childhood caries, risk factors and nutritional status among 3-5-yearold preschool children in Kisarawe, Tanzania. *PLoS ONE* 2021; **16**. DOI:[10.1371/journal.pone.0247240](https://doi.org/10.1371/journal.pone.0247240). |
| 320 |
| Kimmie-Dhansay F, Barrie R, Naidoo S, Roberts T. Prevalence of early childhood caries in South Africa: a systematic review. *BMC Oral Health* 2022; **22**. DOI:[10.1186/s12903-021-01982-6](https://doi.org/10.1186/s12903-021-01982-6). |
| 321 |
| Mothupi KA, Nqcobo CB, Yengopal V. Prevalence of early childhood caries among preschool children in Johannesburg, South Africa. *Journal of Dentistry for Children* 2016; **83**: 83–7. |
| 322 |
| Popoola BO, Onyejaka N, Folayan MO. Prevalence of developmental dental hard-tissue anomalies and association with caries and oral hygiene status of children in Southwestern, Nigeria. *BMC Oral Health* 2016; **17**. DOI:[10.1186/s12903-016-0236-6](https://doi.org/10.1186/s12903-016-0236-6). |
| 323 |
| Seifu B, Yigzaw N, Haile K, Reshid Z, Asfaw H. Prevalence of depression, anxiety and associated factors among patients with dental disease attending outpatient department in Addis Ababa public hospitals, Addis Ababa, Ethiopia: a multicenter cross-sectional study. *BMC Oral Health* 2021; **21**. DOI:[10.1186/s12903-021-02012-1](https://doi.org/10.1186/s12903-021-02012-1). |
| 324 |
| Adam RZ, Kimmie-Dhansay F. Prevalence of Denture-Related Stomatitis in Edentulous Patients at a Tertiary Dental Teaching Hospital. *Front Oral Health* 2021; **2**: 772679. |
| 325 |
| Okoye LO, Ekwueme O-EC, Sote EO, Amaechi BT. Prevalence of dental fluorosis among 12-15-year-old students in Enugu Metropolis, Nigeria. *Indian Journal of Dental Research* 2019; **30**: 462–7. |
| 326 |
| Aidara AW, Bourgeois D. [Prevalence of dental caries: national pilot study comparing the severity of decay (CAO) vs ICDAS index in Senegal]. *Odonto-stomatologie tropicale = Tropical dental journal* 2014; **37**: 53–63. |
| 327 |
| Que L, Jia M, You Z, *et al.* Prevalence of dental caries in the first permanent molar and associated risk factors among sixth-grade students in São Tomé Island. *BMC Oral Health* 2021; **21**. DOI:[10.1186/s12903-021-01846-z](https://doi.org/10.1186/s12903-021-01846-z). |
| 328 |
| Djossou D, Nancy J, Houinato D, Lanchoessi D. Prevalence of dental caries in school in the city of Ouidah in 2013. *Odonto-stomatologie tropicale = Tropical dental journal* 2015; **38**: 15–22. |
| 329 |
| Masumo RM, Ndekero TS, Carneiro LC. Prevalence of dental caries in deciduous teeth and oral health related quality of life among preschool children aged 4-6 years in Kisarawe, Tanzania. *BMC Oral Health* 2020; **20**. DOI:[10.1186/s12903-020-1032-x](https://doi.org/10.1186/s12903-020-1032-x). |
| 330 |
| Olabisi AA, Udo UA, Ehimen UG, Bashiru BO, Gbenga OO, Adeniyi AO. Prevalence of dental caries and oral hygiene status of a screened population in Port Harcourt, Rivers State, Nigeria. *J Int Soc Prev Community Dent* 2015; **5**: 59–63. |
| 331 |
| Masiga MA, M’imunya JM. Prevalence of dental caries and its impact on quality of life (QoL) among HIV-infected children in Kenya. *Journal of Clinical Pediatric Dentistry* 2013; **38**: 83–7. |
| 332 |
| Shitie A, Addis R, Tilahun A, Negash W. Prevalence of Dental Caries and Its Associated Factors among Primary School Children in Ethiopia. *International Journal of Dentistry* 2021; **2021**. DOI:[10.1155/2021/6637196](https://doi.org/10.1155/2021/6637196). |
| 333 |
| Uwayezu D, Gatarayiha A, Nzayirambaho M. Prevalence of dental caries and associated risk factors in children living with disabilities in Rwanda: A cross-sectional study. *Pan African Medical Journal* 2020; **36**: 1–10. |
| 334 |
| Teshome A, Muche A, Girma B. Prevalence of Dental Caries and Associated Factors in East Africa, 2000–2020: Systematic Review and Meta-Analysis. *Frontiers in Public Health* 2021; **9**. DOI:[10.3389/fpubh.2021.645091](https://doi.org/10.3389/fpubh.2021.645091). |
| 335 |
| Andegiorgish AK, Weldemariam BW, Kifle MM, *et al.* Prevalence of dental caries and associated factors among 12 years old students in Eritrea. *BMC Oral Health* 2017; **17**. DOI:[10.1186/s12903-017-0465-3](https://doi.org/10.1186/s12903-017-0465-3). |
| 336 |
| Arigbede AO, Omitola OG. Prevalence of Dental Caries among Adult Patients Attending a Tertiary Dental Institution in South-South Region of Nigeria. *port harcourt med J* 2011; **6**: 52–8. |
| 337 |
| Koleoso ON, Akhigbe KO. Prevalence of dental anxiety and the psychometric properties of modified dental anxiety scale in Nigeria. *World Journal of Dentistry* 2014; **5**: 53–9. |
| 338 |
| Friedman Rubin P, Erez A, Peretz B, Birenboim-Wilensky R, Winocur E. Prevalence of bruxism and temporomandibular disorders among orphans in southeast Uganda: A gender and age comparison. *Cranio - Journal of Craniomandibular Practice* 2018; **36**: 243–9. |
| 339 |
| Umanah A, Omogbai A-A, Osagbemiro B. Prevalence of artificially created maxillary midline diastema and its complications in a selected Nigerian population. *African Health Sciences* 2015; **15**: 226–32. |
| 340 |
| Carneiro LC, Kimambo AE. Prevalence of Acrylic Removable Partial Dentures among Adult Patients Attending Public Dental Clinics in Dar-es-Salaam, Tanzania. *International Journal of Prosthodontics and Restorative Dentistry* 2022; **16**: 61–6. |
| 341 |
| Azodo CC, Agbor MA. Prevalence and unmet treatment need of traumatized incisor among Cameroonian schoolchildren in North West Province. *Odonto-stomatologie tropicale = Tropical dental journal* 2015; **38**: 33–8. |
| 342 |
| Musinguzi N, Kemoli A, Okullo I. Prevalence and Treatment Needs for Early Childhood Caries Among 3–5-Year-Old Children From a Rural Community in Uganda. *Frontiers in Public Health* 2019; **7**. DOI:[10.3389/fpubh.2019.00259](https://doi.org/10.3389/fpubh.2019.00259). |
| 343 |
| Daniyan SY, Abalaka ME. Prevalence and susceptibility pattern of bacterial isolates of dental caries in a secondary health care institution, Nigeria. *Shiraz E Medical Journal* 2011; **12**: 125–39. |
| 344 |
| Ayo-Yusuf OA, Okagbare TE, Ayo-Yusuf IJ. Prevalence and socio-economic disparities in fissure sealant placement among adolescents in the Limpopo Province, South Africa. *SADJ : journal of the South African Dental Association = tydskrif van die Suid-Afrikaanse Tandheelkundige Vereniging* 2011; **66**: 380–3. |
| 345 |
| Masumo R, Bardsen A, Mashoto K, Astrom AN. Prevalence and socio-behavioral influence of early childhood caries, ECC, and feeding habits among 6 - 36 months old children in Uganda and Tanzania. *BMC Oral Health* 2012; **12**. DOI:[10.1186/1472-6831-12-24](https://doi.org/10.1186/1472-6831-12-24). |
| 346 |
| Koffi-Coulibaly NT, Pockpa ZAD, Mobio GS. Prevalence and severity of periodontitis among adults in Côte d’Ivoire according to the new EFP/AAP periodontal disease classification. *J Adv Periodontol Implant Dent* 2021; **13**: 76–83. |
| 347 |
| Koffi-Coulibaly NT, Pockpa ZAD, Mobio GS, Struillou X, Soueidan A. Prevalence and risk indicators for severe periodontitis in Côte d’Ivoire. *J Adv Periodontol Implant Dent* 2022; **14**: 7–12. |
| 348 |
| Belay AS, Achimano AA. Prevalence and Risk Factors for Periodontal Disease Among Women Attending Antenatal Care in Public Hospitals, Southwest Ethiopia, 2022: A Multicenter Cross-Sectional Study. *Clinical, Cosmetic and Investigational Dentistry* 2022; **14**: 153–70. |
| 349 |
| Udoye CI, Jafarzadeh H, Kinoshita J-I, Manabe A, Kobayashi M. Prevalence and reasons for extraction of endodontically treated teeth in adult Nigerians. *Journal of Contemporary Dental Practice* 2018; **19**: 1470–4. |
| 350 |
| Bello S, Olatunbosun W, Adeoye J, Adebayo A, Ikimi N. Prevalence and presentation of hyperdontia in a non-syndromic, mixed Nigerian population. *Journal of Clinical and Experimental Dentistry* 2019; **11**: e930–6. |
| 351 |
| Raj Kumar V, Yadav P, Kahsu E, Girkar F, Chakraborty R. Prevalence and pattern of mandibular third molar impaction in eritrean population: A retrospective study. *Journal of Contemporary Dental Practice* 2017; **18**: 100–6. |
| 352 |
| Iyun OI, Denloye OO, Bankole OO, Popoola BO. Prevalence and pattern of early childhood caries in Ibadan, Nigeria. *Afr J Med Med Sci* 2014; **43**: 239–44. |
| 353 |
| Adeniyi AA, Agbaje O, Onigbinde O, *et al.* Prevalence and pattern of dental caries among a sample of nigerian public primary school children. *Oral Health and Preventive Dentistry* 2012; **10**: 267–74. |
| 354 |
| Umeh OD, Sanu OO, Utomi IL, Nwaokorie FO. Prevalence and intensity of bacteraemia following orthodontic procedures. *International orthodontics* 2016; **14**: 80–94. |
| 355 |
| Kemoli A, Gjørup H, Nørregaard M-LM, *et al.* Prevalence and impact of infant oral mutilation on dental occlusion and oral health-related quality of life among Kenyan adolescents from Maasai Mara. *BMC Oral Health* 2018; **18**. DOI:[10.1186/s12903-018-0631-2](https://doi.org/10.1186/s12903-018-0631-2). |
| 356 |
| Hewlett SA, Calys-Tagoe BNL, Yawson AE, *et al.* Prevalence and geographic distribution of edentulism among older Ghanaians. *Journal of Public Health Dentistry* 2015; **75**: 74–83. |
| 357 |
| Oyedele TA, Jegede AT, Folayan MO. Prevalence and family structures related factors associated with crown trauma in school children resident in suburban Nigeria. *BMC Oral Health* 2016; **16**. DOI:[10.1186/s12903-016-0314-9](https://doi.org/10.1186/s12903-016-0314-9). |
| 358 |
| Kalanzi D, Mayanja-Kizza H, Nakanjako D, Mwesigwa CL, Ssenyonga R, Amaechi BT. Prevalence and factors associated with dental caries in patients attending an HIV care clinic in Uganda: A cross sectional study. *BMC Oral Health* 2019; **19**. DOI:[10.1186/s12903-019-0847-9](https://doi.org/10.1186/s12903-019-0847-9). |
| 359 |
| Kutesa A, Kasangaki A, Nkamba M, Muwazi L, Okullo I, Rwenyonyi CM. Prevalence and factors associated with dental caries among children and adults in selected districts in Uganda. *African Health Sciences* 2015; **15**: 1302–7. |
| 360 |
| Minja IK, Jovin AC, Mandari GJ. Prevalence and factors associated with dental anxiety among primary school teachers in Ngara district, Tanzania. *Tanzania Journal of Health Research* 2016; **18**. DOI:[10.4314/thrb.v18i1.6](https://doi.org/10.4314/thrb.v18i1.6). |
| 361 |
| Ize-Iyamu IN, Isiekwe MC. Prevalence and factors associated with anterior open bite in 2 to 5 year old children in Benin city, Nigeria. *African Health Sciences* 2012; **12**: 446–51. |
| 362 |
| Ibiyemi O, Zohoori FV, Valentine RA, Kometa S, Maguire A. Prevalence and extent of enamel defects in the permanent teeth of 8-year-old Nigerian children. *Community Dentistry and Oral Epidemiology* 2018; **46**: 54–62. |
| 363 |
| Minja IK, Astrom AN, Masalu JR. Prevalence and distribution of oral health knowledge according to sociodemographic, behavioural and clinical characteristics in selected coastal districts of Tanzania. *Tanzania Journal of Health Research* 2016; **18**: 1–13. |
| 364 |
| Bashiru BO, Omotola OE. Prevalence and determinants of dental anxiety among adult population in Benin City, Nigeria. *European Journal of General Dentistry* 2016; **5**: 99–103. |
| 365 |
| Umeizudike KA, Ayanbadejo PO, Savage KO, Akanmu AS, Nwhator SO, Emeka CI. Prevalence and Determinants of Chronic periodontitis in HIV positive patients in Nigeria. *Asian Pacific Journal of Tropical Disease* 2014; **4**: 306–12. |
| 366 |
| Musinguzi N, Kemoli A, Okullo I. Prevalence and dental effects of infant oral mutilation or Ebiino among 3-5 year-old children from a rural district in Uganda. *BMC Oral Health* 2019; **19**. DOI:[10.1186/s12903-019-0890-6](https://doi.org/10.1186/s12903-019-0890-6). |
| 367 |
| Mthethwa J, Mahomed O. Prevalence and demographic predictors of adult dental caries among at public oral health facilities in two districts in kwazulu-natal, south africa: A cross sectional study. *International Journal of Dentistry and Oral Science* 2021; **8**: 3341–6. |
| 368 |
| Oyaro B, Lokken E, Alumera H, *et al.* Prevalence and correlates of periodontitis among Kenyan women planning to conceive. *BMC Oral Health* 2022; **22**. DOI:[10.1186/s12903-022-02243-w](https://doi.org/10.1186/s12903-022-02243-w). |
| 369 |
| Oziegbe EO, Esan TA. Prevalence and clinical consequences of untreated dental caries using PUFA index in suburban Nigerian school children. *European Archives of Paediatric Dentistry* 2013; **14**: 227–31. |
| 370 |
| Mwangosi IEAT, Majenge JM. Prevalence and awareness of oral manifestations among people living with HIV/AIDS attending counselling and treatment centres in Iringa Municipality, Tanzania. *Tanzania Journal of Health Research* 2011; **13**: 205–13. |
| 371 |
| Onyejaka NK, Olatosi OO, Ndukwe NA, Amobi EO, Okoye LO, Nwamba NP. Prevalence and associated factors of dental caries among primary school children in South-East Nigeria. *Nigerian Journal of Clinical Practice* 2021; **24**: 1300–6. |
| 372 |
| Ngozi AE, Ifeyinwa OJ. Prevalence and antimycotic susceptibility profile of Candida species in the oral cavities of HIV/AIDS patients and pregnant women in Nsukka, Nigeria. *Malaysian Journal of Microbiology* 2022; **18**: 271–81. |
| 373 |
| Udoye C, Sede M. Prevalence and analysis of factors related to ooccurrence of pulp stone in adult restorative patients. *Ann Med Health Sci Res* 2011; **1**: 9–14. |
| 374 |
| Udoye CI, Sede MA. Prevalence and Analysis of Factors Related to Occurrence of Pulp Stone in Adult Restorative Patients. *Ann med health sci res (Online)* 2011; **1**: 9–14. |
| 375 |
| James O, Sabo VY, Adamson OO, *et al.* Presentation and Management of Atypical Orofacial Clefts: A Single-Institution Experience for 13 Year Period. *Cleft Palate Craniofac J* 2021; : 10556656211055012. |
| 376 |
| Ugwumba CU, Adeyemo WL, Odeniyi OM, Arotiba GT, Ogunsola FT. Preoperative administration of 0.2% chlorhexidine mouthrinse reduces the risk of bacteraemia associated with intra-alveolar tooth extraction. *J Craniomaxillofac Surg* 2014; **42**: 1783–8. |
| 377 |
| Watson GE, Lynch M, Myers GJ, *et al.* Prenatal exposure to dental amalgam: Evidence from the Seychelles Child Development Study main cohort. *Journal of the American Dental Association* 2011; **142**: 1283–94. |
| 378 |
| Watson GE, Evans K, Thurston SW, *et al.* Prenatal exposure to dental amalgam in the Seychelles Child Development Nutrition Study: Associations with neurodevelopmental outcomes at 9 and 30 months. *NeuroToxicology* 2012; **33**: 1511–7. |
| 379 |
| Onwuka C, Onwuka CI, Iloghalu EI, *et al.* Pregnant women utilization of dental services: still a challenge in low resource setting. *BMC Oral Health* 2021; **21**. DOI:[10.1186/s12903-021-01746-2](https://doi.org/10.1186/s12903-021-01746-2). |
| 380 |
| Diouf M, Cisse D, Lo CMM, Ly M, Faye D, Ndiaye O. Pregnant women living in areas of endemic fluorosis in Senegal and low birthweight newborns: Case-control study. *Revue d’Epidemiologie et de Sante Publique* 2012; **60**: 103–8. |
| 381 |
| Lasisi TJ, Ugwuadu PN. Pregnancy related changes in human salivary secretion and composition in a Nigerian population. *African journal of medicine and medical sciences* 2014; **43**: 347–51. |
| 382 |
| Adeniyi AA, Oyapero A. Predisposing, enabling and need factors influencing dental service utilization among a sample of adult Nigerians. *Population Medicine* 2020; **2**. DOI:[10.18332/popmed/128504](https://doi.org/10.18332/popmed/128504). |
| 383 |
| Ayele FA, Taye BW, Ayele TA, Gelaye KA. Predictors of Dental caries among children 7-14 years old in Northwest Ethiopia: A community based cross-sectional study. *BMC Oral Health* 2013; **13**. DOI:[10.1186/1472-6831-13-7](https://doi.org/10.1186/1472-6831-13-7). |
| 384 |
| Buwembo W, Kutesa A, Muwazi L, Rwenyonyi CM. Prediction of width of un-erupted incisors, canines and premolars in a Ugandan population: A cross sectional study. *BMC Oral Health* 2012; **12**. DOI:[10.1186/1472-6831-12-23](https://doi.org/10.1186/1472-6831-12-23). |
| 385 |
| Adesina OA, Efunkoya AA, Omeje KU, Idon PI. Postoperative complications from primary repair of cleft lip and palate in a semi-urban Nigerian teaching hospital. *Niger Med J* 2016; **57**: 155–9. |
| 386 |
| Muche A, Saniotis A. Position of mandibular foramen and its clinical implications. *Italian Journal of Anatomy and Embryology* 2019; **124**: 319–28. |
| 387 |
| Clauss A, Sie A, Zabre P, Schmoll J, Sauerborn R, Listl S. Population-Based Prevalence of Oral Conditions as a Basis for Planning Community-Based Interventions: An Epidemiological Study From Rural Burkina Faso. *Frontiers in Public Health* 2021; **9**. DOI:[10.3389/fpubh.2021.697498](https://doi.org/10.3389/fpubh.2021.697498). |
| 388 |
| Antony KM, Kazembe PN, Pace RM, *et al.* Population-Based Estimation of Dental Caries and Periodontal Disease Rates of Gravid and Recently Postpartum Women in Lilongwe, Malawi. *AJP Reports* 2019; **9**: E268–74. |
| 389 |
| Oji C, Chukwuneke F. Poor oral Hygiene may be the Sole Cause of Oral Cancer. *Journal of Maxillofacial and Oral Surgery* 2012; **11**: 379–83. |
| 390 |
| Trilisinskaya Y, Smrekova E, Komlosi M, Ondova P, Svobodova H, Voinescu B. Poor dental health resulting to dental infections among South Sudanese refugees. *Lekarsky Obzor* 2016; **65**: 278–80. |
| 391 |
| Camara SA, Bah A, Fofana L, Tolno SE. Place de la prescription médicamenteuse dans la prise en charge des pathologies bucco dentaires chez l’enfant au centre dentaire de l’université de Conakry. *African Journal of Dentistry and Implantology* 2019; **14**: 22–30. |
| 392 |
| Jordan RA, Lucaciu A, Fotouhi K, Markovic L, Gaengler P, Zimmer S. Pilot pathfinder survey of oral hygiene and periodontal conditions in the rural population of The Gambia (West Africa). *International Journal of Dental Hygiene* 2011; **9**: 53–9. |
| 393 |
| Diouf M, Bodian S, Lo CMM, *et al.* Pharmacovigilance among dentists: A survey of practitioners in Dakar, Senegal. *Sante Publique* 2013; **25**: 69–76. |
| 394 |
| Esan TA, Mothupi KA, Schepartz LA. Permanent tooth emergence: Timing and sequence in a sample of Black Southern African children. *American Journal of Physical Anthropology* 2018; **167**: 827–39. |
| 395 |
| Hewlett SA, Anto F, Blankson PK, *et al.* Periodontitis prevalence and severity in an African population: A cross-sectional study in the Greater Accra Region of Ghana. *Journal of Periodontology* 2022; **93**: 732–44. |
| 396 |
| Buwembo W, Munabi IG, Kaddumukasa M, *et al.* Periodontitis and Rheumatoid Arthritis in sub-Saharan Africa, gaps and way forward: a systematic review and meta-analysis. *Open journal of stomatology* 2019; **9**: 215–26. |
| 397 |
| Nwhator SO, Umeizudike KA, Samuel TA, Soroye MO, Umeizudike TI. Periodontitis & sub-fertility; opinions and practices of Nigerian specialists. *West Afr J Med* 2013; **32**: 267–71. |
| 398 |
| Tefera AT, Girma B, Adane A, *et al.* Periodontal status of students living with disability in Amhara region, Ethiopia: a cross-sectional study. *BMC Oral Health* 2022; **22**. DOI:[10.1186/s12903-022-02377-x](https://doi.org/10.1186/s12903-022-02377-x). |
| 399 |
| Popoola BO, Dosumu EB, Ifesanya JU. Periodontal status and treatment need among adolescents in Ibadan, Southwestern Nigeria. *Brazilian Journal of Oral Sciences* 2015; **14**: 117–21. |
| 400 |
| Onigbinde O, Sorunke M, Braimoh M, Adeniyi A. Periodontal Status and Some Variables among Pregnant Women in a Nigeria Tertiary Institution. *Ann Med Health Sci Res* 2014; **4**: 852–7. |
| 401 |
| Tormeti D, Nii-Aponsah H, Sackeyfio J, *et al.* Periodontal status and oral hygiene practices among adults in a peri-urban fishing community in Ghana. *Pan African Medical Journal* 2022; **42**. DOI:[10.11604/pamj.2022.42.126.24557](https://doi.org/10.11604/pamj.2022.42.126.24557). |
| 402 |
| Umeizudike KA, Ayanbadejo PO, Onajole AT, Umeizudike TI, Alade GO. Periodontal status and its association with self-reported hypertension in non-medical staff in a university teaching hospital in Nigeria. *Odonto-stomatologie tropicale = Tropical dental journal* 2016; **39**: 47–55. |
| 403 |
| Azodo CC, Umoh AO. Periodontal issues; tooth loss and oral hygiene measures among tobacco using drivers in Benin City. *Journal of Medicine and Biomedical Research* 2014; **13**: 51–60. |
| 404 |
| Tefera A, Bekele B. Periodontal disease status and associated risk factors in patients attending a tertiary hospital in Northwest Ethiopia. *Clinical, Cosmetic and Investigational Dentistry* 2020; **12**: 485–92. |
| 405 |
| Chikte U, Pontes CC, Karangwa I, *et al.* Periodontal disease status among adults from South Africa-Prevalence and effect of smoking. *International Journal of Environmental Research and Public Health* 2019; **16**. DOI:[10.3390/ijerph16193662](https://doi.org/10.3390/ijerph16193662). |
| 406 |
| Ouédraogo D-D, Tiendrébéogo J, Guiguimdé PLW, *et al.* Periodontal disease in patients with rheumatoid arthritis in sub-Saharan Africa: A case-control study. *Revue du Rhumatisme (Edition Francaise)* 2016; **83**: 311–2. |
| 407 |
| Azodo CC, Umoh AO. Periodontal Disease Awareness and Knowledge among Nigerian Primary School Teachers. *Ann Med Health Sci Res* 2015; **5**: 340–7. |
| 408 |
| Muwazi L, Rwenyonyi CM, Nkamba M, *et al.* Periodontal conditions, low birth weight and preterm birth among postpartum mothers in two tertiary health facilities in Uganda. *BMC Oral Health* 2014; **14**. DOI:[10.1186/1472-6831-14-42](https://doi.org/10.1186/1472-6831-14-42). |
| 409 |
| Opeodu OI, Dosumu EB, Arowojolu MO. Periodontal Condition and Treatment Needs of Some Pregnant Women in Ibadan, Nigeria. *Ann Med Health Sci Res* 2015; **5**: 213–7. |
| 410 |
| Miranda-Rius J, Brunet-Llobet L, Lahor-Soler E, Mrina O, Mashala EI, Mahande MJ. Periodontal and dental conditions of a school population in a volcanic region of tanzania with highly fluoridated community drinking water. *African Health Sciences* 2020; **20**: 476–87. |
| 411 |
| Coker M, El-Kamary SS, Enwonwu C, *et al.* Perinatal HIV Infection and Exposure and Their Association with Dental Caries in Nigerian Children. *Pediatric Infectious Disease Journal* 2018; **37**: 59–65. |
| 412 |
| Harjunmaa U, Doyle R, Järnstedt J, *et al.* Periapical infection may affect birth outcomes via systemic inflammation. *Oral Diseases* 2018; **24**: 847–55. |
| 413 |
| Omoregie FO, Ojo MA, Saheeb BDO, Odukoya O. Periapical granuloma associated with extracted teeth. *Nigerian Journal of Clinical Practice* 2011; **14**: 293–6. |
| 414 |
| Bettens K, Bruneel L, Alighieri C, *et al.* Perceptual Speech Outcomes After Early Primary Palatal Repair in Ugandan Patients With Cleft Palate. *Cleft Palate-Craniofacial Journal* 2021; **58**: 999–1011. |
| 415 |
| Khan SB, Omar R, Chikte UM. Perceptions regarding the shortened dental arch among dental practitioners in the Western Cape Province, South Africa. *SADJ : journal of the South African Dental Association = tydskrif van die Suid-Afrikaanse Tandheelkundige Vereniging* 2012; **67**: 60, 62–4, 66. |
| 416 |
| Kutesa AM, Frantz J. Perceptions of undergraduate dental students at maker ere college of health sciences; Kampala; Uganda towards patient record keeping. *Afr j health prof educ* 2016; **8**: 33–6. |
| 417 |
| Molete M, Stewart A, Moolla A, Igumbor JO. Perceptions of provincial and district level managers’ on the policy implementation of school oral health in South Africa. *BMC Health Services Research* 2021; **21**. DOI:[10.1186/s12913-020-06004-9](https://doi.org/10.1186/s12913-020-06004-9). |
| 418 |
| Kang’Ethe T, Owino RO, Macigo FG. Perceptions and experiences of dentists towards child abuse and neglect in nairobi city county, kenya. *East African Medical Journal* 2016; **93**: 655–60. |
| 419 |
| Bamise CT, Oginni AO, Adedigba MA, Olagundoye OO. Perception of patients with amalgam fillings about toxicity of mercury in dental amalgam. *Journal of Contemporary Dental Practice* 2012; **13**: 289–93. |
| 420 |
| Oketade IO, Osiro O, Ibiyemi ST, Ibiyemi O. Perception of patients attending a tertiary hospital in Nigeria about good dental practice: A pilot study. *Nigerian Journal of Clinical Practice* 2013; **16**: 478–82. |
| 421 |
| Adekoya MN, Dacosta OO, Utomi IL. Perception of dental midline deviation amongst dental professionals and lay people. *Niger J Dent Res* 2019; **4**: 13–8. |
| 422 |
| Akinyamoju AO, Adeyemi BF, Odofin AD, Balogun AO, Akinyamoju CA. Perception and utilization of oral histopathology services by general practice dentist in southwest nigeria. *Ann Ib Postgrad Med* 2017; **15**: 103–8. |
| 423 |
| Bankole OO, Lawal FB, Balogun A. Perception and Practices of Nomadic Women in a Rural Community in Southwestern Nigeria to Their Children’s Oral Health. *International Quarterly of Community Health Education* 2017; **38**: 17–26. |
| 424 |
| Ajayi DM, Gbadebo SO, Adebayo GE. Perception about tooth colour and appearance among patients seen in a tertiary hospital, south-west, nigeria. *Pan African Medical Journal* 2021; **38**: 1–15. |
| 425 |
| Nyamuryekung’e KK, Mlangwa MM, Chaffee BW. Perceived Patient Barriers to Tooth-Retaining Treatment in Tanzania. *JDR Clinical and Translational Research* 2022. DOI:[10.1177/23800844221121262](https://doi.org/10.1177/23800844221121262). |
| 426 |
| Isiekwe IG, Adeyemi TE, Aikins EA, Umeh OD. Perceived impact of the COVID-19 pandemic on orthodontic practice by orthodontists and orthodontic residents in Nigeria. *Journal of the World Federation of Orthodontists* 2020; **9**: 123–8. |
| 427 |
| Fukuda H, Hayashi Y, Toda K, Kaneko S, Wagaiyu E. Perceived general health in relation to oral health status in a rural Kenyan elderly population. *BMC Oral Health* 2021; **21**. DOI:[10.1186/s12903-021-01525-z](https://doi.org/10.1186/s12903-021-01525-z). |
| 428 |
| Amobi EO, Mafeni J, Adekoya-Sofowora CA. Perceived and normative needs of facial cleft patients seen in Nigeria. *Pesquisa Brasileira em Odontopediatria e Clinica Integrada* 2018; **18**. DOI:[10.4034/PBOCI.2018.181.13](https://doi.org/10.4034/PBOCI.2018.181.13). |
| 429 |
| Keboa MT, Madathil SA, Nicolau B. Perceived and Assessed Dental Treatment Needs of Schoolchildren in Benoe Division, Cameroon. *JDR Clinical and Translational Research* 2019; **4**: 160–6. |
| 430 |
| Osunde OD, Amole IO, Ver-Or N, *et al.* Pediatric maxillofacial injuries at a Nigerian teaching hospital: A three-year review. *Nigerian Journal of Clinical Practice* 2013; **16**: 149–54. |
| 431 |
| Oga EA, Schumaker LM, Alabi BS, *et al.* Paucity of HPV-related Head and Neck Cancers (HNC) in Nigeria. *PLoS ONE* 2016; **11**. DOI:[10.1371/journal.pone.0152828](https://doi.org/10.1371/journal.pone.0152828). |
| 432 |
| Ogunmuyiwa SA, Gbolahan OO, Ayantunde AA, Odewabi AA. Patterns, severity, and management of maxillofacial injuries in a suburban South Western Nigeria tertiary center. *Niger J Surg* 2015; **21**: 38–42. |
| 433 |
| Nguendo-Yongsi B. Patterns, Practices, and Level of Buccodental Hygiene in Individuals Aged 5 to 17 Years in Bafia, Cameroon. *International Journal of Prosthodontics and Restorative Dentistry* 2022; **16**: 9–13. |
| 434 |
| Fadare JO, Oshikoya KA, Obimakinde OS, *et al.* Patterns of drugs prescribed for dental outpatients in Nigeria: findings and implications. *Acta Odontologica Scandinavica* 2017; **75**: 496–506. |
| 435 |
| Olayemi AB, Adeniyi AO, Samuel U, Emeka OA. Pattern, severity, and management of cranio-maxillofacial soft-tissue injuries in Port Harcourt, Nigeria. *Journal of Emergencies, Trauma and Shock* 2013; **6**: 235–40. |
| 436 |
| Taiwo OAS Olujide Oladele Godwin Ugochukwu Ndubuizu. Pattern of Utilization of Dental Services at Federal Medical Centre; Katsina; Northwest Nigeria. *Sahel medical journal (Print)* 2014; **17**: 108–11. |
| 437 |
| Adedigba MA, Adekanmbi VT, Asa S, Fakande I. Pattern of utilisation of dental health care among HIV-positive adult Nigerians. *Oral Health and Preventive Dentistry* 2016; **14**: 215–25. |
| 438 |
| Enabulele JE, Oginni AO, Sede MA, Oginni FO. Pattern of traumatised anterior teeth among adult Nigerians and complications from late presentation. *BMC Research Notes* 2016; **9**. DOI:[10.1186/s13104-016-1871-3](https://doi.org/10.1186/s13104-016-1871-3). |
| 439 |
| Ashiwaju M, Folayan M, Sote E, Isikwe M. Pattern of tooth extraction in children attending tertiary health care centers in nigeria: A prospective study. *Journal of Clinical Pediatric Dentistry* 2011; **36**: 107–10. |
| 440 |
| Ibiyemi O, Ibiyemi T, Taiwo J. Pattern of tooth discoloration and care-seeking behavior among adolescents in an underserved rural community in Nigeria. *European Journal of General Dentistry* 2017; **6**: 36–41. |
| 441 |
| Oyetola EO, Mogaji IK, Agho TO, Ayilara OA. Pattern of presentation of oral ulcerations in patients attending an oral medicine clinic in nigeria. *Ann Ib Postgrad Med* 2018; **16**: 9–11. |
| 442 |
| Folaranmi N, Akaji E, Onyejaka N. Pattern of presentation of oral health conditions by children at University of Nigeria Teaching Hospital, Enugu: A retrospective study. *Nigerian Journal of Clinical Practice* 2014; **17**: 47–50. |
| 443 |
| Eigbobo JO, Onyeaso CO, Okolo NI. Pattern of presentation of oral health conditions among children at the university of port harcourt teaching hospital (UPTH), port harcourt, Nigeria. *Pesquisa Brasileira em Odontopediatria e Clinica Integrada* 2011; **11**: 105–9. |
| 444 |
| Oyetola OE, Owotade FJ, Fatusi OA, Olatunji S. Pattern of presentation and outcome of routine dental interventions in patients with halitosis. *Niger Postgrad Med J* 2016; **23**: 215–20. |
| 445 |
| Adeyemo WL, Taiwo OA, Adeyemi MO, Adewole RA, Gbotolorun OM. Pattern of presentation and management of lip injuries in a Nigerian hospital. *Nigerian Journal of Clinical Practice* 2012; **15**: 436–41. |
| 446 |
| Onyejaka NK, Lawal BN, Okechukwu RA, Osayande MO, Alamba IC. Pattern of patients’ attendance to the dental clinic of federal college of dental technology and therapy, Enugu, Nigeria. *Pan African Medical Journal* 2018; **29**. DOI:[10.11604/pamj.2018.29.151.14563](https://doi.org/10.11604/pamj.2018.29.151.14563). |
| 447 |
| Eshete M. Pattern of Orofacial Clefts at A Tertiary Care Hospital in Ethiopia. *Ethiopian journal of health sciences* 2021; **31**: 1175–84. |
| 448 |
| Lasisi TJ, Abdus-Salam RA. Pattern of Oral Health Among a Population of Pregnant Women in Southwestern Nigeria. *Arch Basic Appl Med* 2018; **6**: 99–103. |
| 449 |
| Butt FMA, Ogeng’O J, Bahra J, Chindia ML. Pattern of odontogenic and nonodontogenic cysts. *Journal of Craniofacial Surgery* 2011; **22**: 2160–2. |
| 450 |
| Msagati F, Simon ENM, Owibingire S. Pattern of occurrence and treatment of impacted teeth at the Muhimbili National Hospital, Dar es Salaam, Tanzania. *BMC Oral Health* 2013; **13**. DOI:[10.1186/1472-6831-13-37](https://doi.org/10.1186/1472-6831-13-37). |
| 451 |
| Bello S, Osodin T, Oketade I, *et al.* Pattern of maxillofacial surgical conditions in North Central Nigeria: A 5-year experience of an indigenous surgical mission. *Nigerian Journal of Clinical Practice* 2017; **20**: 1283–8. |
| 452 |
| Moshy JR, Msemakweli BS, Owibingire SS, Sohal KS. Pattern of mandibular fractures and helmet use among motorcycle crash victims in Tanzania. *African Health Sciences* 2020; **20**: 789–97. |
| 453 |
| Okoye L, Onah I, Ekwueme O, Agu K. Pattern of malocclusion and caries experience in unrepaired cleft lip and palate patients in Enugu. *Nigerian Journal of Clinical Practice* 2020; **23**: 59–64. |
| 454 |
| Mwansasu C, Liyombo E, Moshi N, Mpondo BC. Pattern of head and neck cancers among patients attending muhimbili national hospital tanzania. *Tanzania Journal of Health Research* 2015; **17**. DOI:[10.4314/thrb.v17i1.4](https://doi.org/10.4314/thrb.v17i1.4). |
| 455 |
| Ikhodaro IP, Janada Y. Pattern of endodontic treatment among Nigerian adults: A single centre study. *Pesquisa Brasileira em Odontopediatria e Clinica Integrada* 2018; **18**. DOI:[10.4034/PBOCI.2018.181.71](https://doi.org/10.4034/PBOCI.2018.181.71). |
| 456 |
| Soyele OO, Ladeji AM, Adebiyi KE, *et al.* Pattern of distribution of reactive localised hyperplasia of the oral cavity in patients at a tertiary health institution in Nigeria. *African Health Sciences* 2019; **19**: 1687–94. |
| 457 |
| Ogundana OM, Effiom OA, Odukoya O. Pattern of distribution of odontogenic tumours in sub-Saharan Africa. *International Dental Journal* 2017; **67**: 308–17. |
| 458 |
| Goyal S, Goyal S. Pattern of Dental Malocclusion in Orthodontic Patients in Rwanda: a Retrospective Hospital Based Study. *Rwanda med j (Online)* 2012; **69**: 13–8. |
| 459 |
| Umanah A, Osagbemiro B, Arigbede A. Pattern of demand for endodontic treatment by adult patients in port-harcourt, South-South Nigeria. *J West Afr Coll Surg* 2012; **2**: 12–23. |
| 460 |
| Magwesela FM, Rabiel H, Mung’Ong’O CM. Pattern of congenital anomalies among pediatric surgical patients in a tertiary care hospital in northern Tanzania. *World Journal of Pediatric Surgery* 2022; **5**. DOI:[10.1136/wjps-2021-000410](https://doi.org/10.1136/wjps-2021-000410). |
| 461 |
| Olojede A, Gbotolorun OM, Ogundana OM, *et al.* Pattern of assault-related maxillofacial injuries treated at the general hospital, Lagos, Nigeria. *J West Afr Coll Surg* 2016; **6**: 68–82. |
| 462 |
| Obimakinde OS, Okoje VN, Fasola AO. Pattern of Assault-induced Oral and Maxillofacial Injuries in Ado-Ekiti, Nigeria. *Niger J Surg* 2012; **18**: 88–91. |
| 463 |
| Yemitan TA, Adediran VE, Ogunbanjo BO. Pattern of agenesis and morphologic variation of the maxillary lateral incisors in nigerian orthodontic patients. *J West Afr Coll Surg* 2017; **7**: 71–91. |
| 464 |
| Gbadebo OS, Bamidele K. Pattern and trend of non-odontogenic orofacial pain at a tertiary health facility in Sub Saharan West Africa. *Journal of Stomatology* 2015; **68**: 59–73. |
| 465 |
| Olatosi OO, Oyapero A, Akinwande KO, Ayedun OS, Aladenika ET, Obe OI. Pattern and prevalence of dental anomalies among a paediatric population in Lagos, Nigeria. *The Nigerian postgraduate medical journal* 2022; **29**: 167–72. |
| 466 |
| Fomete B, Agbara R, Osunde DO, Bello SA, Yunus AA, Goni BA. Pattern and Presentation of tongue lesions in Kaduna, Nigeria: an 8-year Review. *Niger J Dent Res* 2018; **3**: 84–90. |
| 467 |
| Taiwo OA, Sulaiman OA, Shoremi OO, Danlami J, Adeniji OU, Olawole WO. Pattern and indications for adult permanent teeth extractions in Zamfara state, Northwest Nigeria. *Journal of Stomatology* 2015; **68**: 183–90. |
| 468 |
| Nyamuryekung’e KK, Lahti SM, Tuominen RJ. Patients’ willingness to pay for dental services in a population with limited restorative services. *Community Dental Health* 2018; **35**: 167–72. |
| 469 |
| Awotile AO, Oyapero A, Adenuga-Taiwo OA, Enone LL, Menakaya IN, Loto AO. Patients’ Management Patterns for Restorative Treatment Procedures: A 4-Year Overview at the Restorative Clinic of a Tertiary Hospital in Nigeria. *Pesquisa Brasileira em Odontopediatria e Clinica Integrada* 2022; **22**. DOI:[10.1590/pboci.2022.017](https://doi.org/10.1590/pboci.2022.017). |
| 470 |
| Damilare KA, Abass D, Antwi-Agyei D, *et al.* Patients Perceived Knowledge, Attitude, and Practice of Dental Abscess Management in Periurban District, Ghana. *BioMed research international* 2022; **2022**: 2266347. |
| 471 |
| Obilade OA, da Costa OO, Sanu OO. Patient/parent expectations of orthodontic treatment. *Int Orthod* 2017; **15**: 82–102. |
| 472 |
| Obadan-Udoh E, van der Berg-Cloete S, Ramoni R, Kalenderian E, White JG. Patient-reported dental safety events: A South African perspective. *Journal of Patient Safety* 2021; **17**: E866–73. |
| 473 |
| Deh SY, Nonvignon J, Aikins M, Agyemang SA, Ar-Yeetey GC. Patient treatment cost of oral diseases in Ghana. *Ghana Medical Journal* 2022; **56**: 176–84. |
| 474 |
| Wilson K, Bouchiba M, Vithlani G, Holmes R. Patient satisfaction with oral urgent treatment (OUT) in North West Tanzania. *British Dental Journal* 2013; **215**: 131–4. |
| 475 |
| Edetanlen EB, Babalola O, Lawani U. Patient satisfaction with intraoral examination in a Nigerian tertiary hospital. *Calabar Journal of Health Sciences* 2021; **5**: 15–20. |
| 476 |
| Desai J. Patient Perception following Alloplastic Total Temporomandibular Joint Replacement. *Ann Maxillofac Surg* 2018; **8**: 83–5. |
| 477 |
| Mothibe JV, Patel M. Pathogenic characteristics of Candida albicans isolated from oral cavities of denture wearers and cancer patients wearing oral prostheses. *Microb Pathog* 2017; **110**: 128–34. |
| 478 |
| Anyanechi CE, Ekabua KJ, Ekpenyong AB, Ekabua JE. Parturients’ Awareness and Perception of Benefits of Breast Feeding in the Prevention of Infant and Childhood Oral and Dental Diseases. *Ghana medical journal* 2017; **51**: 83–7. |
| 479 |
| Akinboboye B, Azodo C, Soroye M. Partial edentulism and unmet prosthetic needs amongst young adult Nigeria. *Odonto-stomatologie tropicale = Tropical dental journal* 2014; **37**: 47–52. |
| 480 |
| Luyten A, D’haeseleer E, Budolfsen D, *et al.* Parental satisfaction in Ugandan children with cleft lip and palate following synchronous lip and palatal repair. *Journal of Communication Disorders* 2013; **46**: 321–9. |
| 481 |
| James O, Erinoso OA, Ogunlewe AO, Adeyemo WL, Ladeinde AL, Ogunlewe MO. Parental Age and the Risk of Cleft Lip and Palate in a Nigerian Population - A Case-Control Study. *Ann Maxillofac Surg* 2020; **10**: 429–33. |
| 482 |
| Eboh DEO. Palatal rugae patterns of urhobos in Abraka, South-Southern Nigeria. *International Journal of Morphology* 2012; **30**: 709–13. |
| 483 |
| Udoye CI, Jafarzadeh H. Pain during root canal treatment: an investigation of patient modifying factors. *J Contemp Dent Pract* 2011; **12**: 301–4. |
| 484 |
| Utomi IL, Odukoya OO. Pain and discomfort associated with orthodontic separator placement in patients attending the Lagos University Teaching Hospital, Lagos, Nigeria. *Odonto-stomatologie tropicale = Tropical dental journal* 2013; **36**: 5–13. |
| 485 |
| Kolisa Y, Ayo-Yusuf OA, Makobe DC. Paedodontic general anaesthesia and compliance with follow-up visits at a tertiary oral and dental hospital, South Africa. *SADJ : journal of the South African Dental Association = tydskrif van die Suid-Afrikaanse Tandheelkundige Vereniging* 2013; **68**: 206, 208–12. |
| 486 |
| Eke CB, Akaji EA, Ukoha OM, Muoneke VU, Ikefuna AN, Onwuasigwe CN. Paediatricians’ perception about oral healthcare of children in Nigeria. *BMC Oral Health* 2015; **15**. DOI:[10.1186/s12903-015-0151-2](https://doi.org/10.1186/s12903-015-0151-2). |
| 487 |
| Omoregie FO, Akpata O. Paediatric orofacial tumours: new oral health concern in paediatric patients. *Ghana medical journal* 2014; **48**: 14–9. |
| 488 |
| Blankson PK, Amanor EM, Dai-Kosi AD, *et al.* Paediatric maxillofacial fractures in Ghana: Pattern, household cost, and distress. *International Journal of Paediatric Dentistry* 2021; **31**: 613–8. |
| 489 |
| Akhiwu BI, Osunde DO, Akhiwu HO, *et al.* Paediatric jaw tumours: experiences and findings from a resource limited tertiary health care center. *Pan Afr Med J* 2020; **36**: 111. |
| 490 |
| Akinyele AO, Israel AT, Akang EEU. Paediatric head and neck cancers in Nigeria: implications for treatment planning in resource limited settings. *Niger med j (Online)* 2012; **53**: 245–8. |
| 491 |
| Bham F, Perrie H, Scribante J, Lee C-A. Paediatric dental chair sedation: An audit of current practice in Gauteng, South Africa. *South African medical journal = Suid-Afrikaanse tydskrif vir geneeskunde* 2015; **105**: 461–4. |
| 492 |
| Anyanechi CE. Paediatric and adolescent intra-bony oro-facial tumours and allied lesions in a Nigeria tertiary health facility: An 18-year retrospective analysis. *Journal of Oral and Maxillofacial Surgery, Medicine, and Pathology* 2015; **27**: 412–7. |
| 493 |
| Khan S, Chikte UM, Omar R. Outcomes with a posterior reduced dental arch: a randomised controlled trial. *J Oral Rehabil* 2017; **44**: 870–8. |
| 494 |
| Adegbiji WA, Olajide GT, Agbesanwa AT, Banjo OO. Otological manifestation of temporomandibular joint disorder in Ekiti, a sub-Saharan African country. *Journal of International Medical Research* 2021; **49**. DOI:[10.1177/0300060521996517](https://doi.org/10.1177/0300060521996517). |
| 495 |
| Bah A, Fofana L, Sow I, Camara S. Ostéomyélite mandibulaire odontogéne chez l’enfant: aspects cliniques et thérapeutique au CHU de Conakry. *African Journal of Dentistry and Implantology* 2019; **14**: 14–21. |
| 496 |
| Bengondo C, Mindja ED, Kenna E, Mengong H, Bengono G. Ostéomes maxillo-mandibulaires : épidémiologie, diagnostic, classification et thérapeutique au centre hospitalier et universitaire de Yaoundé (Cameroun). *Health sci dis* 2018; **19**: 79–82. |
| 497 |
| Ndour O, Alumeti D, Fall M, *et al.* Osteogenesis imperfecta in a pediatric surgical center in dakar, senegal: clinical and radiological aspects. *J West Afr Coll Surg* 2011; **1**: 1–14. |
| 498 |
| Adeyemi TE, Aikins EA, Yemitan TA. Orthodontic Treatment Needs of Adolescents in North-Western Nigeria Using the Index of Orthodontic Treatment Needs. *J West Afr Coll Surg* 2021; **11**: 7–13. |
| 499 |
| Akinwonmi BA, Kolawole KA, Folayan MO, Adesunloye AM. Orthodontic treatment need of children and adolescents with special healthcare needs resident in Ile-Ife, Nigeria. *European Archives of Paediatric Dentistry* 2020; **21**: 355–62. |
| 500 |
| Aikins EA, Dacosta OO, Onyeaso CO, Isiekwe MC. Orthodontic treatment need and complexity among nigerian adolescents in rivers state, Nigeria. *International Journal of Dentistry* 2011. DOI:[10.1155/2011/813525](https://doi.org/10.1155/2011/813525). |
| 501 |
| Ernest MA, daCosta OO, Adegbite K, Yemitan T, Adeniran A. Orthodontic treatment motivation and cooperation: A cross-sectional analysis of adolescent patients’ and parents’ responses. *J Orthod Sci* 2019; **8**: 12. |
| 502 |
| Utomi IL, Onyeaso CO. Orthodontic treatment complexity and need in a Nigerian teaching hospital. *Oral Health Dent Manag* 2014; **13**: 562–7. |
| 503 |
| Onah II, Okeke AC, Folaranmi N. Orthodontic needs of patients with cleft lip and palate in enugu, five years post repair. *Ann Ib Postgrad Med* 2020; **18**: S35–8. |
| 504 |
| Chikandiwa A, Pisa PT, Chersich MF, Muller EE, Mayaud P, Delany-Moretlwe S. Oropharyngeal HPV infection: prevalence and sampling methods among HIV-infected men in South Africa. *International Journal of STD and AIDS* 2018; **29**: 776–80. |
| 505 |
| Natana GG, Kalyanyama BM, Simon ENM. Orofacial tumours and tumour-like lesions in children treated at Muhimbili National Hospital, Tanzania. *South Sudan med j* 2019; **12**: 5–8. |
| 506 |
| Agbara R, Obiadazie AC, Fomete B, Omeje KU. Orofacial soft tissue reconstruction with locoregional flaps in a health resource-depleted environment: Experiences from Nigeria. *Archives of Plastic Surgery* 2016; **43**: 265–71. |
| 507 |
| Nabbanja J, Gitta S, Peterson S, Rwenyonyi CM. Orofacial manifestations in HIV positive children attending Mildmay Clinic in Uganda. *Odontology* 2013; **101**: 116–20. |
| 508 |
| Adeyemo WL, Rabiu KA, Okoturo TM, Adebanjo AA, Adewunmi AA, Adeyemi MO. Orofacial injuries associated with eclampsia in patients presenting at a Nigerian Tertiary Hospital. *Journal of Obstetrics and Gynaecology* 2012; **32**: 54–7. |
| 509 |
| Kanmodi KK, Lawal OA, Akintola ZO, Ibiyemi O. Orofacial injuries amongst commercial motorcyclists, drivers, and bus conductors in three rural communities, south-western Nigeria. *International Journal on Disability and Human Development* 2019; **18**: 91–8. |
| 510 |
| Mutwiri KD, Dimba E, Nzioka BM. Orofacial infections in kenya: A retrospective study. *Annals of African Surgery* 2021; **18**: 45–51. |
| 511 |
| Buyu Y, Manyama M, Chandika A, Gilyoma J. Orofacial clefts at Bugando Medical Centre: Associated factors and postsurgical complications. *Cleft Palate-Craniofacial Journal* 2012; **49**: 736–40. |
| 512 |
| Agbenorku P, Agbenorku M, Klutsey E, *et al.* Orofacial cleft outreach in rural Ghana: any positive impact on the community? *European Journal of Plastic Surgery* 2015; **38**: 17–24. |
| 513 |
| Adeyemo WL, Fajolu IB, Temiye EO, Adeyemi MO, Adepoju AA. Orofacial and dental injuries associated with seizures in paediatric patients in Lagos University Teaching Hospital. *International Journal of Pediatric Otorhinolaryngology* 2011; **75**: 670–2. |
| 514 |
| Lasisi TJ, Adeyemi BF, Oluwasola AO, Lasisi OA, Akang EE. Oro-facial squamous cell carcinoma--a twenty-year retrospective clinicopathological study. *African journal of medicine and medical sciences* 2012; **41**: 265–70. |
| 515 |
| Lawal AO, Kolude B, Adisa AO, Adeyemi BF. Oro-facial sarcomas: a review of 88 cases in a Tertiary Institution in Nigeria. *West African journal of medicine* 2013; **32**: 106–9. |
| 516 |
| Adewole RA, Ojini FI, Akinwande JA, Danesi MA. Oro-dental and maxillofacial trauma in epilepsy at a tertiary hospital in Lagos. *West African Journal of Medicine* 2011; **30**: 114–7. |
| 517 |
| Morhason-Bello IO, Baisley K, Pavon MA, *et al.* Oral, genital and anal human papillomavirus infections among female sex workers in Ibadan, Nigeria. *PLoS ONE* 2022; **17**. DOI:[10.1371/journal.pone.0265269](https://doi.org/10.1371/journal.pone.0265269). |
| 518 |
| Agbor MA, Azodo CC. Oral Warts among Children Attending a Missionary Dental Clinic in Cameroon. *Nigerian Medical Practitioner* 2011; **59**. <https://search.bvsalud.org/aimafro/resource/en/biblio-1267979>. |
| 519 |
| Astatkie A, Demissie M, Berhane Y, Worku A. Oral symptoms significantly higher among long-term khat (Catha edulis) users in Ethiopia. *Epidemiol Health* 2015; **37**: e2015009. |
| 520 |
| Mohamed N, Mathiba OP, Mulder R. Oral status of HIV-infected children aged 12 years or younger who attended a Paediatric Infectious Diseases Clinic in Cape Town. *Clinical and Experimental Dental Research* 2020; **6**: 75–81. |
| 521 |
| Adeyemi BF, Olusanya AA, Lawoyin JO. Oral squamous cell carcinoma, socioeconomic status and history of exposure to alcohol and tobacco. *Journal of the National Medical Association* 2011; **103**: 498–502. |
| 522 |
| Khammissa R, Meer S, Lemmer J, Feller L. Oral squamous cell carcinoma in a South African sample: Race/ethnicity, age, gender, and degree of histopathological differentiation. *Journal of Cancer Research and Therapeutics* 2014; **10**: 908–14. |
| 523 |
| Bashiru BO, Anthony IN. Oral self-care practices among university students in Port Harcourt, Rivers State. *Niger Med J* 2014; **55**: 486–9. |
| 524 |
| Fonseca FP, Robinson L, van Heerden MB, van Heerden WFP. Oral plasmablastic lymphoma: A clinicopathological study of 113 cases. *Journal of Oral Pathology and Medicine* 2021; **50**: 594–602. |
| 525 |
| Nwoga MC, Effiom OA, Adeyemi BF, Soyele OO, Okwuosa CU. Oral mucosal melanoma in four Nigerian teaching hospitals. *Nigerian Journal of Clinical Practice* 2019; **22**: 1752–7. |
| 526 |
| Kamulegeya A, Lakor F. Oral maxillofacial tumors and tumor-like conditions: A Ugandan survey. *Pediatric Surgery International* 2011; **27**: 925–30. |
| 527 |
| Sales-Peres SHC, Mapengo MAA, de Moura-Grec PG, Marsicano JA, Sales-Peres AC, Sales-Peres A. Oral manifestations in HIV+ children in Mozambique. *Ciencia e Saude Coletiva* 2012; **17**: 55–60. |
| 528 |
| Rwenyonyi CM, Kutesa A, Muwazi L, Okullo I, Kasangaki A, Kekitinwa A. Oral manifestations in HIV/AIDS-infected children. *European Journal of Dentistry* 2011; **5**: 291–8. |
| 529 |
| Frimpong P, Amponsah EK, Abebrese J, Kim SM. Oral manifestations and their correlation to baseline CD4 count of HIV/AIDS patients in Ghana. *Journal of the Korean Association of Oral and Maxillofacial Surgeons* 2017; **43**: 29–36. |
| 530 |
| Adebola AR, Adeleke SI, Mukhtar M, Osunde OD, Akhiwu BI, Ladeinde A. Oral manifestation of HIV/AIDS infections in paediatric Nigerian patients. *Niger med j (Online)* 2012; **53**: 150–4. |
| 531 |
| Chandran R, Meer S, Feller L. Oral leukoplakia in a South African sample: A clinicopathological study. *Oral Diseases* 2013; **19**: 592–7. |
| 532 |
| Meless D, Ba B, N’Diaye C, *et al.* Oral lesions of HIV-infected children in West Africa in the era of antiretroviral treatments. *Bulletin du Groupèment international pour la recherche scientifique en stomatologie & odontologie* 2011; **50**: 3–4. |
| 533 |
| Mwangosi IEAT, Tillya J. Oral lesions associated with HIV/AIDS in HIV-seropositive patients attending a counselling and treatment centre in Dar es Salaam. *International Dental Journal* 2012; **62**: 197–202. |
| 534 |
| Oladokun RE, Okoje VN, Osinusi K, Obimakinde OS. Oral lesions and their association with CD4 count and viral load in HIV positive Nigerian children. *Oral Health Dent Manag* 2013; **12**: 200–4. |
| 535 |
| Meless D, Ba B, Faye M, *et al.* Oral lesions among HIV-infected children on antiretroviral treatment in West Africa. *Tropical Medicine and International Health* 2014; **19**: 246–55. |
| 536 |
| Simangwa LD, Johansson A-K, Johansson A, Minja IK, Åstrøm AN. Oral impacts on daily performances and its socio-demographic and clinical distribution: A cross-sectional study of adolescents living in Maasai population areas, Tanzania. *Health and Quality of Life Outcomes* 2020; **18**. DOI:[10.1186/s12955-020-01444-7](https://doi.org/10.1186/s12955-020-01444-7). |
| 537 |
| Diawara O, Ba B, Ba M, *et al.* Oral hygiene: Knowledge and practices of students in three quranic schools in Koutiala, Mali. *Pesquisa Brasileira em Odontopediatria e Clinica Integrada* 2018; **18**. DOI:[10.4034/PBOCI.2018.181.53](https://doi.org/10.4034/PBOCI.2018.181.53). |
| 538 |
| Ligali TO, Nzomiwu CL, Orenuga OO. Oral hygiene status, malocclusion and dental trauma among institutionalized visually impaired and sighted adolescents in Lagos State, Nigeria. *Special Care in Dentistry* 2022; **42**: 599–605. |
| 539 |
| Soroye MO, Onigbinde OO. Oral hygiene status, interdental cleaning and perception of gingival bleeding among a group of pregnant women in Nigeria. *Niger J Dent Res (Online)* 2022; **7**: 75–82. |
| 540 |
| Mlenga F, Mumghamba EG. Oral Hygiene Practices, Knowledge, and Self-Reported Dental and Gingival Problems with Rural-Urban Disparities among Primary School children in Lilongwe, Malawi. *International Journal of Dentistry* 2021; **2021**. DOI:[10.1155/2021/8866554](https://doi.org/10.1155/2021/8866554). |
| 541 |
| Aliyu I, Lawal TO, Akhiwu H, Ibrahim ZF. Oral hygiene practices of doctors in a tertiary hospital in North-West Nigeria. *Medical Journal of Dr DY Patil Vidyapeeth* 2018; : 146–51. |
| 542 |
| Ofili D-FC, Esu EB, Ejemot-Nwadiaro RI. Oral hygiene practices and utilization of oral healthcare services among in-school adolescents in Calabar, Cross River state, Nigeria. *Pan African Medical Journal* 2020; **36**: 1–11. |
| 543 |
| Diendéré J, Ouattara S, Kaboré J, Traoré I, Zeba AN, Kouanda S. Oral hygiene practices and their sociodemographic correlates among adults in Burkina Faso: results from the First National Survey. *BMC Oral Health* 2022; **22**. DOI:[10.1186/s12903-022-02118-0](https://doi.org/10.1186/s12903-022-02118-0). |
| 544 |
| Beyene DH, Shashamo BB, Digesa LE, Tariku EZ. Oral hygiene practices and associated factors among patients visiting private dental clinics at Hawassa City, southern Ethiopia, 2018. *International Journal of Dentistry* 2021; **2021**. DOI:[10.1155/2021/8868308](https://doi.org/10.1155/2021/8868308). |
| 545 |
| Kolawole KA, Oziegbe EO, Bamise CT. Oral hygiene measures and the periodontal status of school children. *International Journal of Dental Hygiene* 2011; **9**: 143–8. |
| 546 |
| Lawal IU, Ibrahim R, Ramphoma KJ. Oral hygiene in stroke survivors undergoing rehabilitation: does upper extremity motor function matters? *Top Stroke Rehabil* 2021; **28**: 531–6. |
| 547 |
| Abebe ME, Deressa W, Oladugba V, *et al.* Oral health–related quality of life of children born with orofacial clefts in Ethiopia and their parents. *Cleft Palate-Craniofacial Journal* 2018; **55**: 1153–7. |
| 548 |
| Kolawole KA, Ayodele-Oja MM. Oral health–related quality of life of adolescents assessed with the Malocclusion Impact and Child Perceptions questionnaires. *American Journal of Orthodontics and Dentofacial Orthopedics* 2021; **159**: e149–56. |
| 549 |
| Uguru N, Onwujekwe O, Uguru C, Ogu U, Okwuosa C, Okeke C. Oral health-seeking behavior among different population groups in Enugu Nigeria. *PLoS ONE* 2021; **16**. DOI:[10.1371/journal.pone.0246164](https://doi.org/10.1371/journal.pone.0246164). |
| 550 |
| Akinyamoju CA, Dosumu OO, Taiwo JO, Ogunrinde TJ, Akinyamoju AO. Oral health-related quality of life: acrylic versus flexible partial dentures. *Ghana Med J (Online)* 2019; **53**: 163–9. |
| 551 |
| Ibikunle AA, Adeyemo WL. Oral health-related quality of life following third molar surgery with or without application of ice pack therapy. *Oral and Maxillofacial Surgery* 2016; **20**: 239–47. |
| 552 |
| Ibikunle AA, Adeyemo WL, Ladeinde AL. Oral health-related quality of life following third molar surgery with either oral administration or submucosal injection of prednisolone. *Oral Maxillofac Surg* 2016; **20**: 343–52. |
| 553 |
| Ibikunle AA, Adeyemo WL. Oral Health-Related Quality of Life Following Third Molar Surgery in an African Population. *Contemp Clin Dent* 2017; **8**: 545–51. |
| 554 |
| Adeyemo W, Taiwo O, Oderinu O, Adeyemi M, Ladeinde A, Ogunlewe M. Oral health-related quality of life following non-surgical (routine) tooth extraction: A pilot study. *Contemporary Clinical Dentistry* 2012; **3**: 427–32. |
| 555 |
| Okeigbemen SA, Awhoregba TO, Ojuola GT. Oral health-related knowledge, attitude and practices among trainee community health officers in a nigerian tertiary health institution. *African Journal of Biomedical Research* 2021; **24**: 219–23. |
| 556 |
| Okeigbemen S, Nnawuihe C. Oral health trends and service utilization at a rural outreach dental clinic, Udo, Southern Nigeria. *Journal of International Society of Preventive and Community Dentistry* 2015; **5**: S118–22. |
| 557 |
| Koyio LN, Van Der Sanden WJM, Dimba E, *et al.* Oral health training programs for community and professional health care workers in Nairobi East District increases identification of HIV-infected patients. *PLoS ONE* 2014; **9**. DOI:[10.1371/journal.pone.0090927](https://doi.org/10.1371/journal.pone.0090927). |
| 558 |
| Burnett D, Aronson J, Asgary R. Oral health status, knowledge, attitudes and behaviours among marginalized children in Addis Ababa, Ethiopia. *Journal of Child Health Care* 2016; **20**: 252–61. |
| 559 |
| Michele Lolita Y, Ashu Michael A, Hubert N, Florence D, Jacques B. Oral Health Status of the Elderly at Tonga, West Region, Cameroon. *International Journal of Dentistry* 2015; **2015**. DOI:[10.1155/2015/820416](https://doi.org/10.1155/2015/820416). |
| 560 |
| Adesina KT, Ernest MA, Tobin AO, *et al.* Oral health status of pregnant women in Ilorin, Nigeria. *Journal of Obstetrics and Gynaecology* 2018; **38**: 1093–8. |
| 561 |
| Kebede B, Kemal T, Abera S. Oral health status of patients with mental disorders in Southwest Ethiopia. *PLoS ONE* 2012; **7**. DOI:[10.1371/journal.pone.0039142](https://doi.org/10.1371/journal.pone.0039142). |
| 562 |
| Bensel T, Erhart I, Megiroo S, Kronenberg W, Bömicke W, Hinz S. Oral health status of nursing staff in Ilembula, Wanging’ombe District, Njombe region, Tanzania: a cross-sectional study. *BMC Oral Health* 2022; **22**. DOI:[10.1186/s12903-022-02064-x](https://doi.org/10.1186/s12903-022-02064-x). |
| 563 |
| Mukhari-Baloyi N, Bhayat A, Madiba TK, Nkambule NR. Oral Health Status of Illicit Drug Users in a Health District in South Africa. *European Journal of Dentistry* 2022. DOI:[10.1055/s-0042-1750770](https://doi.org/10.1055/s-0042-1750770). |
| 564 |
| Tefera AT, Girma B, Adane A, *et al.* Oral Health Status of Hearing-Impaired Students Attending Special Need Schools in Amhara Region, Ethiopia: A Cross-Sectional Study. *Clinical, Cosmetic and Investigational Dentistry* 2022; **14**: 19–35. |
| 565 |
| Bissong M, Azodo CC, Agbor MA, Nkuo-Akenji T, Fon PN. Oral health status of diabetes mellitus patients in Southwest Cameroon. *Odonto-stomatologie tropicale = Tropical dental journal* 2015; **38**: 49–57. |
| 566 |
| Denloye O, Ajayi D, Lagunju I. Oral health status of children seen at a paediatric neurology clinic in a tertiary hospital in Nigeria. *Pediatric Dental Journal* 2012; **22**: 16–21. |
| 567 |
| Akaji E, Ashiwaju M. Oral health status of a sample of prisoners in enugu: a disadvantaged population. *Ann Med Health Sci Res* 2014; **4**: 650–3. |
| 568 |
| Africa CWJ, Turton M. Oral Health Status and Treatment Needs of Pregnant Women Attending Antenatal Clinics in KwaZulu-Natal, South Africa. *International Journal of Dentistry* 2019; **2019**. DOI:[10.1155/2019/5475973](https://doi.org/10.1155/2019/5475973). |
| 569 |
| Akinboboye BO, Olutayo J, Azodo CC, Adekunle AA, Oluwaniyi SO. Oral health status and treatment needs of in-patients of a Nigerian psychiatric hospital. *Niger J Dent Res* 2018; **3**. <https://search.bvsalud.org/aimafro/resource/en/biblio-1266967>. |
| 570 |
| Jessani A, Quadri MFA, Lefoka P, *et al.* Oral health status and patterns of dental service utilization of adolescents in lesotho, Southern Africa. *Children* 2021; **8**. DOI:[10.3390/children8020120](https://doi.org/10.3390/children8020120). |
| 571 |
| Ngan WB, Belinga LEE, Nlo’o SE, *et al.* Oral health status and cardiovascular risk profile in Cameroonian military population. *AIMS Public Health* 2021; **8**: 100–9. |
| 572 |
| Gunsam PP, Banka S. Oral health status and behaviour of Mauritians visiting private dental clinics. *Health Education* 2011; **111**: 34–48. |
| 573 |
| Ernest MA, Bolarinwa OA. Oral health seeking behaviour among patients attending outpatient clinic in University of Ilorin teaching hospital (UITH) Ilorin. *Research Journal of Heath  Sciences* 2022; **10**: 80–9. |
| 574 |
| Ocwia J, Olum R, Atim P, *et al.* Oral health seeking behaviors of adults in Nebbi District, Uganda: a community-based survey. *BMC Oral Health* 2021; **21**. DOI:[10.1186/s12903-021-01824-5](https://doi.org/10.1186/s12903-021-01824-5). |
| 575 |
| Sahile AT, Mgutshini T, Ayehu SM. Oral Health Screening Status of Diabetes Patients in Selected Hospitals of Addis Ababa, Ethiopia, 2018. *Patient Relat Outcome Meas* 2020; **11**: 173–80. |
| 576 |
| Umeizudike KA, Osagbemiro BB, Daramola OO, Adeyemo TA. Oral health related quality of life among hiv positive patients attending two hiv outpatient clinics in nigeria-a cross sectional study. *African Health Sciences* 2021; **21**: 566–75. |
| 577 |
| Braimah RO, Ndukwe KC, Owotade FJ, Aregbesola SB. Oral health related quality of life (OHRQoL) following third molar surgery in Sub-Saharan Africans: An observational study. *Pan African Medical Journal* 2016; **25**. DOI:[10.11604/pamj.2016.25.97.7656](https://doi.org/10.11604/pamj.2016.25.97.7656). |
| 578 |
| Isiekwe GI, Onigbogi OO, Olatosi OO, Sofola OO. Oral health quality of life in a nigerian university undergraduate population. *J West Afr Coll Surg* 2014; **4**: 54–74. |
| 579 |
| Lawal FB, Taiwo JO, Oke GA. Oral health practices of adult inhabitants of a traditional community in Ibadan, Nigeria. *Nigerian journal of medicine : journal of the National Association of Resident Doctors of Nigeria* 2013; **22**: 212–7. |
| 580 |
| Folayan MO, El Tantawi M, Oginni O, *et al.* Oral health practices and oral hygiene status as indicators of suicidal ideation among adolescents in Southwest Nigeria. *PLoS ONE* 2021; **16**. DOI:[10.1371/journal.pone.0247073](https://doi.org/10.1371/journal.pone.0247073). |
| 581 |
| Bukar M, Audu BM, Adesina OA, Marupa JY. Oral health practices among pregnant women in North Eastern Nigeria. *Nigerian Journal of Clinical Practice* 2012; **15**: 302–5. |
| 582 |
| Azodo CC, Ezeja EB, Omoaregba, James BO. Oral health of psychiatric patients: The nurse’s perspective. *International Journal of Dental Hygiene* 2012; **10**: 245–9. |
| 583 |
| Maharaj B, Vayej AC. Oral health of patients with severe rheumatic heart disease. *Cardiovascular Journal of Africa* 2012; **23**: 336–9. |
| 584 |
| Molete MP, Yengopal V, Moorman J. Oral health needs and barriers to accessing care among the elderly in Johannesburg. *SADJ : journal of the South African Dental Association = tydskrif van die Suid-Afrikaanse Tandheelkundige Vereniging* 2014; **69**: 352, 354–7. |
| 585 |
| Owino RO, Masiga MA, Macigo FG, Ng′Ang′A PM. Oral health knowledge, hygiene practices and treatment seeking behaviour among 12 year-old children from Kitale municipality in Kenya. *East African Medical Journal* 2011; **88**: 332–7. |
| 586 |
| Carneiro L, Kabulwa M, Makyao M, Mrosso G, Choum R. Oral health knowledge and practices of secondary school students, Tanga, Tanzania. *International Journal of Dentistry* 2011. DOI:[10.1155/2011/806258](https://doi.org/10.1155/2011/806258). |
| 587 |
| Jegede AT, Oyedele TA, Sodipo BO, Folayan MO. Oral health knowledge and practices of dentists practicing in a teaching hospital in Nigeria. *Indian Journal of Dental Research* 2016; **27**: 137–44. |
| 588 |
| Idowu EA, Afolabi AO, Nwhator SO. Oral health knowledge and practice of 12 to 14-year-old Almajaris in Nigeria: A problem of definition and a call to action. *Journal of Public Health Policy* 2016; **37**: 226–43. |
| 589 |
| Akinyamoju CA, Taiwo JO, Uwadia E, Agbogidi JM, Ambeke A. Oral health knowledge and practice among traders in ibadan. *Ann Ib Postgrad Med* 2018; **16**: 150–6. |
| 590 |
| Fantaye W, Nur A, Kifle G, Engida F. Oral health knowledge and oral hygiene practice among visually impaired subjects in Addis Ababa, Ethiopia. *BMC Oral Health* 2022; **22**. DOI:[10.1186/s12903-022-02199-x](https://doi.org/10.1186/s12903-022-02199-x). |
| 591 |
| Crittenden AN, Sorrentino J, Moonie SA, Peterson M, Mabulla A, Ungar PS. Oral health in transition: The Hadza foragers of Tanzania. *PLoS ONE* 2017; **12**. DOI:[10.1371/journal.pone.0172197](https://doi.org/10.1371/journal.pone.0172197). |
| 592 |
| Rwakatema DS, Ananduni KN, Katiti VW, Msuya M, Chugulu J, Kapanda G. Oral health in nursing students at Kilimanjaro Christian Medical Centre teaching hospital in Moshi, Tanzania. *BMC Oral Health* 2015; **15**. DOI:[10.1186/s12903-015-0008-8](https://doi.org/10.1186/s12903-015-0008-8). |
| 593 |
| Mwangosi IEAT, Kiango MM. Oral health experience during pregnancy and dental service utilization in Bariadi District, Tanzania. *Tanzania Journal of Health Research* 2012; **14**: 1–7. |
| 594 |
| Smit DA, Naidoo S. Oral health effects, brushing habits and management of methamphetamine users for the general dental practitioner. *British Dental Journal* 2015; **218**: 531–6. |
| 595 |
| Mbagwu FC, Okoye IB, Umunnakwe GC. Oral health disease and library service delivery among library staff of the universities in Nigeria. *Library Philosophy and Practice* 2019; **2019**. <https://www.scopus.com/inward/record.uri?eid=2-s2.0-85066079282&partnerID=40&md5=53929bd5a9766396661de8cafa6f23ff>. |
| 596 |
| Hackley DM, Jain S, Pagni SE, Finkelman M, Ntaganira J, Morgan JP. Oral health conditions and correlates: a National Oral Health Survey of Rwanda. *Global Health Action* 2021; **14**. DOI:[10.1080/16549716.2021.1904628](https://doi.org/10.1080/16549716.2021.1904628). |
| 597 |
| Bayingana C, Kayintenkore J, Kayonga Y, Mambo Muvunyi C, Musemakweli A. Oral Health Care Habits and its Role in Preventing Adverse Prenancy Outucomes in Rwanda. *Rwanda med j (Online)* 2012; **69**: 11–4. |
| 598 |
| Turton M, Naidoo S. Oral health care experiences of people living with HIV in Kwazulu-Natal and Western Cape, South Africa. *International Journal of Human Rights in Healthcare* 2015; **8**: 59–69. |
| 599 |
| Paurobally N, Kruger E, Tennant M. Oral Health Behaviour and Predictors of Oral Health Behaviour Among Patients With Diabetes in the Republic of Mauritius. *International Dental Journal* 2022; **72**: 106–15. |
| 600 |
| Lawal FB, Bankole OO. Oral health awareness and practices of primary school teachers in Ibadan, Nigeria. *J West Afr Coll Surg* 2014; **4**: 47–65. |
| 601 |
| Jessani A, Choi J, El-Rabbany A, Lefoka P, Quadri MFA, Laronde DM. Oral health and psychosocial predictors of quality of life and general well-being among adolescents in lesotho, southern africa. *Children* 2021; **8**. DOI:[10.3390/children8070582](https://doi.org/10.3390/children8070582). |
| 602 |
| Nouaman MN, Meless DG, Coffie PA, *et al.* Oral health and HIV infection among female sex workers in Abidjan, Côte d’Ivoire. *BMC Oral Health* 2015; **15**. DOI:[10.1186/s12903-015-0129-0](https://doi.org/10.1186/s12903-015-0129-0). |
| 603 |
| Isiekwe IG, Ligali TO, Abdullahi MAS, Adeyemo WL. Oral Habits in Children With Orofacial Clefts: A Comparative Study. *Cleft Palate Craniofac J* 2021; **58**: 984–9. |
| 604 |
| Kolawole KA, Folayan MO, Agbaje HO, Oyedele TA, Onyejaka NK, Oziegbe EO. Oral habits and malocclusion in children resident in Ile-Ife Nigeria. *European Archives of Paediatric Dentistry* 2019; **20**: 257–65. |
| 605 |
| Meless GD, Guinan J-C, Sangaré AD, *et al.* Oral epidemiological profile of patients attending public oral health services in haut Sassandra region, in Côte D’Ivoire. *Journal of Public Health in Africa* 2019; **10**. DOI:[10.4081/jphia.2019.1064](https://doi.org/10.4081/jphia.2019.1064). |
| 606 |
| Simangwa LD, Åstrøm AN, Johansson A, Minja IK, Johansson A-K. Oral diseases and socio-demographic factors in adolescents living in Maasai population areas of Tanzania: A cross-sectional study. *BMC Oral Health* 2018; **18**. DOI:[10.1186/s12903-018-0664-6](https://doi.org/10.1186/s12903-018-0664-6). |
| 607 |
| Wang Y, Ramos-Gomez F, Kemoli AM, *et al.* Oral Diseases and Oral Health-Related Quality of Life among Kenyan Children and Adolescents with HIV. *JDR Clin Trans Res* 2022; : 23800844221087950. |
| 608 |
| Simangwa LD, Åstrøm AN, Johansson A, Minja IK, Johansson A-K. Oral diseases and oral health related behaviors in adolescents living in Maasai population areas of Tanzania: A cross-sectional study. *BMC Pediatrics* 2019; **19**. DOI:[10.1186/s12887-019-1655-8](https://doi.org/10.1186/s12887-019-1655-8). |
| 609 |
| Sede MA, Ehizele AO. Oral diseases and diabetes: Nigerian medical and dental caregivers’ perspective. *Ann Afr Med* 2015; **14**: 193–9. |
| 610 |
| Arima H, Calliope AS, Fukuda H, *et al.* Oral cleaning habits and the copy number of periodontal bacteria in pregnant women and its correlation with birth outcomes: an epidemiological study in Mibilizi, Rwanda. *BMC Oral Health* 2022; **22**. DOI:[10.1186/s12903-022-02443-4](https://doi.org/10.1186/s12903-022-02443-4). |
| 611 |
| Naidu TK, Naidoo SK, Ramdial PK. Oral cavity squamous cell carcinoma metastasis to the submandibular gland. *J Laryngol Otol* 2012; **126**: 279–84. |
| 612 |
| Owotade FJ, Patel M, Ralephenya TRMD, Vergotine G. Oral Candida colonization in HIV-positive women: associated factors and changes following antiretroviral therapy. *J Med Microbiol* 2013; **62**: 126–32. |
| 613 |
| Fidele NB, Patrick SMN, Okonji OC, Kazadi EK. Oral cancer awareness and knowledge: Survey of dentists in Democratic Republic of the Congo. *Journal of Cancer Policy* 2022; **32**. DOI:[10.1016/j.jcpo.2022.100332](https://doi.org/10.1016/j.jcpo.2022.100332). |
| 614 |
| Kalema N, de n Boon S, Cattamanchi A, *et al.* Oral antimicrobial rinse to reduce mycobacterial culture contamination among tuberculosis suspects in uganda: A prospective study. *PLoS ONE* 2012; **7**. DOI:[10.1371/journal.pone.0038888](https://doi.org/10.1371/journal.pone.0038888). |
| 615 |
| Taiwo AO, Braimah RO, Ibikunle AA, *et al.* Oral and maxillofacial tumours in children and adolescents: Clinicopathologic audit of 75 cases in an academic medical centre, Sokoto, Northwest Nigeria. *African Journal of Paediatric Surgery* 2017; **14**: 37–42. |
| 616 |
| Oyedeji OA, Gbolahan OO, Oluwatoyin Abe E, Agelebe E. Oral and dental lesions in HIV infected nigerian children. *Pan African Medical Journal* 2015; **20**. DOI:[10.11604/pamj.2015.20.287.5273](https://doi.org/10.11604/pamj.2015.20.287.5273). |
| 617 |
| Faye B, Sarr M, Bane K, Aidara AW, Niang SO, Kane AW. One-year clinical evaluation of the bonding effectiveness of a one-step, self-etch adhesive in noncarious cervical lesion therapy. *Int J Dent* 2015; **2015**: 984065. |
| 618 |
| Loots E, Sartorius B, Madiba TE, Mulder CJJ, Clarke DL. Oesophageal squamous cell cancer in a South African tertiary hospital: A risk factor and presentation analysis. *South African Journal of Surgery* 2017; **55**: 42–6. |
| 619 |
| Lawal AO, Adisa AO, Olusanya AA. Odontogenic tumours: A review of 266 cases. *Journal of Clinical and Experimental Dentistry* 2013; **5**: e13–7. |
| 620 |
| Mamabolo M, Noffke C, Raubenheimer E. Odontogenic tumours manifesting in the first two decades of life in a rural African population sample: A 26 year retrospective analysis. *Dentomaxillofacial Radiology* 2011; **40**: 331–7. |
| 621 |
| Aregbesola B, Soyele O, Effiom O, Gbotolorun O, Taiwo O, Amole I. Odontogenic Tumours in Nigeria: A multicentre study of 582 cases and review of the literature. *Medicina Oral Patologia Oral y Cirugia Bucal* 2018; **23**: e761–6. |
| 622 |
| Iyogun CA, Omitola OG, Ukegheson GE. Odontogenic tumors in Port Harcourt: South-South geopolitical zone of Nigeria. *Journal of Oral and Maxillofacial Pathology* 2016; **20**: 190–3. |
| 623 |
| Kebede B, Tare D, Bogale B, Alemseged F. Odontogenic tumors in Ethiopia: Eight years retrospective study. *BMC Oral Health* 2017; **17**. DOI:[10.1186/s12903-017-0347-8](https://doi.org/10.1186/s12903-017-0347-8). |
| 624 |
| Titinchi F, Hassan BA, Morkel JA, Nortje C. Odontogenic myxoma: a clinicopathological study in a South African population. *Journal of Oral Pathology and Medicine* 2016; **45**: 599–604. |
| 625 |
| Fomete B, Agbara R, Omeje KU, Oguntayo AO. Odontogenic Cervicofacial Infection in Pregnancy: A Need for Oral Care. *J Family Reprod Health* 2021; **15**: 1–7. |
| 626 |
| Gevera P, Mouri H, Maronga G. Occurrence of fluorosis in a population living in a high-fluoride groundwater area: Nakuru area in the Central Kenyan Rift Valley. *Environmental Geochemistry and Health* 2019; **41**: 829–40. |
| 627 |
| Foche AN, Bushabu FN, Mana CM, *et al.* Occurrence of dental alveolus traumas at the university clinics of Kinshasa: Second part, preliminary study of 93 cases. *Pan African Medical Journal* 2018; **29**. DOI:[10.11604/pamj.2018.29.50.13763](https://doi.org/10.11604/pamj.2018.29.50.13763). |
| 628 |
| Osaiyuwu O. Occurrence of bacteraemia following oral and maxillofacial surgical procedures in Port Harcourt, Nigeria. *African Health Sciences* 2021; **21**: 1692–700. |
| 629 |
| Osazuwa-Peters N, Obarisiagbon A, Azodo CC, Ehizele AO, Obuekwe ON. Occupational exposure to sharp injuries among medical and dental house officers in Nigeria. *International Journal of Occupational Medicine and Environmental Health* 2013; **26**: 283–90. |
| 630 |
| Osiatuma VI, Otuyemi OD, Kolawole KA, Ogunbanjo BO, Amusa YB. Occlusal characteristics of children with hypertrophied adenoids in Nigeria. *International Orthodontics* 2015; **13**: 26–42. |
| 631 |
| Harjunmaa U, Järnstedt J, Dewey KG, *et al.* Nutrient supplementation may adversely affect maternal oral health - a randomised controlled trial in rural Malawi. *Maternal and Child Nutrition* 2016; **12**: 99–110. |
| 632 |
| Dagnew ZA, Abraham IA, Beraki GG, Tesfamariam EH, Mittler S, Tesfamichael YZ. Nurses’ attitude towards oral care and their practicing level for hospitalized patients in Orotta National Referral Hospital, Asmara-Eritrea: A cross-sectional study. *BMC Nursing* 2020; **19**. DOI:[10.1186/s12912-020-00457-3](https://doi.org/10.1186/s12912-020-00457-3). |
| 633 |
| Obilade OA, Sanu OO, Costa OO. [Not Available]. *Int Orthod* 2016; **14**: 366–85. |
| 634 |
| Ajayi EO. Normative and self-perceived orthodontic treatment need in Nigerian school children. *Acta Odontologica Scandinavica* 2014; **73**: 364–7. |
| 635 |
| Folayan MO, Ozeigbe EO, Onyejaeka N, Chukwumah NM, Oyedele T. Non-third molar related pericoronitis in a sub-urban Nigeria population of children. *Nigerian Journal of Clinical Practice* 2014; **17**: 18–22. |
| 636 |
| Mbuyi-Musanzayi S, Kayembe TJ, Kashal MK, *et al.* Non-syndromic cleft lip and/or cleft palate: Epidemiology and risk factors in Lubumbashi (DR Congo), a case-control study. *J Craniomaxillofac Surg* 2018; **46**: 1051–8. |
| 637 |
| Buwembo W, Munabi IG, Kaddumukasa M, *et al.* Non-surgical oral hygiene interventions on disease activity of Rheumatoid arthritis patients with periodontitis: A randomized controlled trial. *Journal of dental research, dental clinics, dental prospects* 2020; **14**: 26–36. |
| 638 |
| Adedapo HA, Lawal AO, Adisa AO, Adeyemi BF. Non-doctor consultations and self-medication practices in patients seen at a tertiary dental center in Ibadan. *Indian Journal of Dental Research* 2011; **22**: 795–8. |
| 639 |
| Lawal FB, Dauda MA. Non-clinical factors and predictors of self-rating of oral health among young adolescents in a rural Nigerian population. *Brazilian Journal of Oral Sciences* 2018; **17**. DOI:[10.20396/BJOS.V17I0.8651895](https://doi.org/10.20396/BJOS.V17I0.8651895). |
| 640 |
| Feller L, Khammissa RAG, Altini M, Lemmer J. Noma (cancrum oris): An unresolved global challenge. *Periodontol 2000* 2019; **80**: 189–99. |
| 641 |
| Adeyemo WL, Oderinu OH, Olojede ACO, Ayodele AOS, Fashina AA. Nigerian dentists’ knowledge of the current guidelines for preventing infective endocarditis. *Community Dental Health* 2011; **28**: 178–81. |
| 642 |
| Nwhator SO, Ijarogbe O, Agbaje O, Olojede CO, Olatunji AB. Nigerian dentists’ knowledge of aggressive periodontitis. *Journal of Indian Society of Periodontology* 2014; **18**: 78–81. |
| 643 |
| Azodo CC, Ehizele AO, Umoh A, Okechukwu R. Nigerian dental therapy students’ knowledge, attitude, and willingness to care for patients with HIV. *Journal of Dental Education* 2013; **77**: 793–800. |
| 644 |
| Popoola BO, Denloye OO. Nigerian Dental Students’ Assessment of their Clinical Learning. *African Journal of Biomedical Research* 2015; **18**: 29–35. |
| 645 |
| Idowu EA, Nwhator SO, Afolabi AO. Nigeria′s street children, epitome of oral health disparity and inequality. *Pan African Medical Journal* 2020; **36**: 1–10. |
| 646 |
| Ndjidda Bakari W, Thiam D, Mbow NL, *et al.* New classification of periodontal diseases (NCPD): an application in a sub-Saharan country. *BDJ Open* 2021; **7**: 16. |
| 647 |
| Titinchi F, Nortje CJ, Parker ME, van Rensburg LJ. Nevoid basal cell carcinoma syndrome: A 40-year study in the South African population. *Journal of Oral Pathology and Medicine* 2013; **42**: 162–5. |
| 648 |
| Geerts GAVM. Neutral zone or conventional mandibular complete dentures: a randomised crossover trial comparing oral health-related quality of life. *J Oral Rehabil* 2017; **44**: 702–8. |
| 649 |
| Agbara R, Fomete B, Omeje KU. Neurogenic Tumors of the Oral and Maxillofacial Region in a Nigerian Population. *West Afr J Med* 2022; **39**: 628–34. |
| 650 |
| Watson GE, van Wijngaarden E, Love TMT, *et al.* Neurodevelopmental outcomes at 5 years in children exposed prenatally to maternal dental amalgam: The seychelles child development nutrition study. *Neurotoxicology and Teratology* 2013; **39**: 57–62. |
| 651 |
| Adebiyi KE, Emmanuel MM. Neoplastic Salivary Gland Lesions:A Retrospective Analysis of 135 Cases from Lagos State University Teaching Hospital, Ikeja, Lagos, Nigeria. *West Afr J Med* 2014; **33**: 206–10. |
| 652 |
| Hamese K, Mashego TAB, Mzezewa S. Neonatal Cleft Lip Repair in Babies with Breastfeeding Difficulties at Polokwane Mankweng Hospital Complex. *S Afr j child health (Online)* 2014; **8**: 157–9. |
| 653 |
| Ghotane SG, Don-Davis P, Kamara D, Harper PR, Challacombe SJ, Gallagher JE. Needs-led human resource planning for Sierra Leone in support of oral health. *Human Resources for Health* 2021; **19**. DOI:[10.1186/s12960-021-00623-x](https://doi.org/10.1186/s12960-021-00623-x). |
| 654 |
| Wood NH, Blignaut E, Lemmer J, Meyerov R, Feller L. Necrotizing periodontal diseases in a semirural district of south Africa. *AIDS Research and Treatment* 2011; **2011**. DOI:[10.1155/2011/638584](https://doi.org/10.1155/2011/638584). |
| 655 |
| Ministry of Health. National oral health survey Namibia 2010/11. 2013; : 45–45. |
| 656 |
| Oludiran OO, Omotoso DR, Sakpa CL. Nasofacial indices among children in Southern Nigeria. *African Journal of Biomedical Research* 2012; **15**: 141–3. |
| 657 |
| Iyun OI, Bankole O, Denloye OO, Popoola BO. Mutans streptococci colonization in early childhood caries in Ibadan, Nigeria. *Pediatric Dental Journal* 2014; **24**: 153–8. |
| 658 |
| Dieng S, Cisse D, Lombrail P, Azogui-Lévy S. Mothers’ oral health literacy and children’s oral health status in Pikine, Senegal: A pilot study. *PLoS ONE* 2020; **15**. DOI:[10.1371/journal.pone.0226876](https://doi.org/10.1371/journal.pone.0226876). |
| 659 |
| Adimorah GN, Ubesie AC, Chinawa JM. Mothers’ beliefs about infant teething in Enugu, South-east Nigeria: A cross sectional study. *BMC Research Notes* 2011; **4**. DOI:[10.1186/1756-0500-4-228](https://doi.org/10.1186/1756-0500-4-228). |
| 660 |
| Sulaiman AO, Adebayo GE. Most frequently selected shade for advance restoration delivered in a tertiary hospital facility in south western Nigeria. *Ann Ib Postgrad Med* 2019; **17**: 157–61. |
| 661 |
| Mmbaga BT, Mwasamwaja A, Mushi G, *et al.* Missing and decayed teeth, oral hygiene and dental staining in relation to esophageal cancer risk: ESCCAPE case-control study in Kilimanjaro, Tanzania. *International Journal of Cancer* 2021; **148**: 2416–28. |
| 662 |
| Getaneh A, Derseh F, Abreha M, Yirtaw T. Misconceptions and traditional practices towards infant teething symptoms among mothers in Southwest Ethiopia. *BMC Oral Health* 2018; **18**. DOI:[10.1186/s12903-018-0619-y](https://doi.org/10.1186/s12903-018-0619-y). |
| 663 |
| Adeyemo WL, Taiwo OA, Ladeinde AL, Ogunlewe MO, Adeyemi MO, Adepoju AA. Mid-facial fractures: a 5-year retrospective review in a Nigerian teaching hospital. *Niger J Med* 2012; **21**: 31–5. |
| 664 |
| James O, Ligali TO, Abba MH, Olasoji HO. Medically compromised conditions among Dental patients: A prospective study of the attitude of patients toward medication and medical recall visits. *Nigerian quarterly journal of hospital medicine* 2015; **25**: 129–32. |
| 665 |
| Bassey GO, Osunde OD, Anyanechi CE. Maxillofacial tumors and tumor-like lesions in a nigerian teaching hospital: An eleven year retrospective analysis. *African Health Sciences* 2014; **14**: 56–63. |
| 666 |
| Bernard EK, Akama MK, Odhiambo WA, Chindia ML, Mua B. Maxillofacial soft tissue injuries in Nairobi, Kenya. *East African Medical Journal* 2012; **89**: 306–11. |
| 667 |
| Porter M, Lownie M, Cleaton-Jones P. Maxillofacial injury: A retrospective analysis of time lapse between injury and treatment in a South African academic maxillofacial and oral surgery unit. *South African Journal of Surgery* 2013; **51**: 138–42. |
| 668 |
| Adebayo ET, Iyogun CA. Maxillofacial injuries sustained during military peace keeping mission in Liberia: The Nigerian Experience (1990-1997). *Niger J Dent Res* 2020; **5**: 118–22. |
| 669 |
| Bassey GO, Anyanechi CE, Chukwuneke FN. Maxillofacial injuries in Calabar south-south, Nigeria: a 5 year study of jawbone fractures. *Nigerian journal of medicine : journal of the National Association of Resident Doctors of Nigeria* 2011; **20**: 245–9. |
| 670 |
| Adebayo ET, Fomete B, Adelusi EA, Ahaji LE, Nnawuhie UC. Maxillofacial injuries due to motorcycle accidents from suburban Nigeria. *Journal of Oral and Maxillofacial Surgery, Medicine, and Pathology* 2023; **35**: 30–5. |
| 671 |
| Krishnan UC, Byanyima RK, Faith A, Kamulegeya A. Maxillofacial injuries among trauma patients undergoing head computerized tomography; A Ugandan experience. *Int J Crit Illn Inj Sci* 2017; **7**: 236–40. |
| 672 |
| Obimakinde OS, Ogundipe KO, Rabiu TB, Okoje VN. Maxillofacial fractures in a budding teaching hospital: a study of pattern of presentation and care. *Pan Afr Med J* 2017; **26**: 218. |
| 673 |
| Oluwafemi Adewale Adesina, John Chukwudumebi Wemambu, Taofiq Olamide Opaleye, Ajibola Yussuf Salami. maxillofacial fractures a three year survey. 2019; **9**: 51–6. |
| 674 |
| Parkins G, Boamah MO, Avogo D, Ndanu T, Nuamah IK. Maxillofacial and concomitant injuries in multiple injured patients at Korle Bu Teaching Hospital, Ghana. *West African journal of medicine* 2014; **33**: 51–5. |
| 675 |
| Millogo M, Sanfo M, Idani M, Dargani MF, Coulibaly A, Konsem T. [Maxillo-facial loss of substances of traumatic origin at the Yalgado Ouedraogo University Hospital]. *Ann Chir Plast Esthet* 2022. DOI:[10.1016/j.anplas.2022.09.003](https://doi.org/10.1016/j.anplas.2022.09.003). |
| 676 |
| Akinmoladun VI, Akinyamoju CA, Olaniran FO, Olaopa OI. Maxillectomy and Quality of Life: Experience from a Nigerian Tertiary Institution. *Niger J Surg* 2018; **24**: 125–30. |
| 677 |
| Fomete B, Agbara R, Osunde OD, Ogbeifun JO. Maxillectomy and its surgical indications in a tertiary health care centre in north-western Nigeria: Analysis of 66 cases. *Journal of Oral and Maxillofacial Surgery, Medicine, and Pathology* 2017; **29**: 198–202. |
| 678 |
| Ba B, Keita K, Coulibaly A, *et al.* [Maxillary ameloblastoma at the odonto-stomatology University Hospital in Bamako]. *Mali Med* 2017; **32**: 1–8. |
| 679 |
| Eigbobo JO, Onyeaso CO. Maternal knowledge and awareness of factors affecting oral health in the paediatric population. *Odontostomatol Trop* 2013; **36**: 15–24. |
| 680 |
| Kimmie-Dhansay F, Barrie R, Roberts T, Naidoo S. Maternal and infant risk factors and risk indicators associated with early childhood caries in South Africa: a systematic review. *BMC Oral Health* 2022; **22**. DOI:[10.1186/s12903-022-02218-x](https://doi.org/10.1186/s12903-022-02218-x). |
| 681 |
| Omo JO, Sede MA, Esan TA. Masticatory efficiency of shortened dental arch subjects with removable partial denture: A comparative study. *Niger J Clin Pract* 2017; **20**: 459–63. |
| 682 |
| MacNab A, Kasangaki A. ‘Many voices, one song’: A model for an oral health programme as a first step in establishing a health promoting school. *Health Promotion International* 2012; **27**: 63–73. |
| 683 |
| Bancolé Pognon SA, Biotchané I, Zinsou R, Yèhouénou L, Zannou DM. Manifestations stomatologiques et infection à VIH au Centrenational hospitalier et universitaire de Cotonou (Bénin). *Medecine Buccale Chirurgie Buccale* 2013; **19**: 149–54. |
| 684 |
| Fadiga MS, Lama B, Nabe AB, Keita T, Loua JB. Manifestations cliniques et traitements des diastemes chez les personnes adultes à l’hopital national Donka. *African Journal of Dentistry and Implantology* 2022; **22**: 8–12. |
| 685 |
| Anyanechi CE, Saheeb BD. Mandibular sites prone to fracture: analysis of 174 cases in a Nigerian tertiary hospital. *Ghana medical journal* 2011; **45**: 111–4. |
| 686 |
| Braimah RO, Ibikunle AA, Abubakar U, *et al.* Mandibular reconstruction with autogenous non-vascularised bone graft. *African Health Sciences* 2019; **19**: 2768–77. |
| 687 |
| Anyanechi Ce. mandibular fractures associated with domestic violence in calabar nigeria. 2011; **44**: 155–8. |
| 688 |
| Makanjuola JO, Umesi DC, Ndukwe AN, *et al.* Managing the phase-down of amalgam amongst Nigerian dental professionals and students: A national survey. *European Journal of Dental Education* 2020; **24**: 666–78. |
| 689 |
| Ogunkeyede SA, Ogundoyin OO. Management outcome of thyroglossal cyst in a tertiary health center in southwest Nigeria. *Pan African Medical Journal* 2019; **34**. DOI:[10.11604/pamj.2019.34.154.18765](https://doi.org/10.11604/pamj.2019.34.154.18765). |
| 690 |
| Fomete B, Adebayo ET, Ononiwu CN. Management of salivary gland tumors in a Nigerian tertiary institution. *Annals of African Medicine* 2015; **14**: 148–54. |
| 691 |
| James O, Adekunle AA, Adamson OO, *et al.* Management of orofacial cleft in Nigeria - A retrospective study. *Annals of Maxillofacial Surgery* 2020; **10**: 434–8. |
| 692 |
| Guthua S, Kamau M, Abinya N. Management of maxillofacial osteosarcomas in Kenya. *Annals of African Surgery* 2020; **17**: 26–9. |
| 693 |
| Anyanechi CE, Osunde OD, Bassey GO. Management of Extra-Capsular Temporo-Mandibular Joint Ankylosis: Does Conservative Approach to Treatment Have a Role? *Journal of Maxillofacial and Oral Surgery* 2015; **14**: 339–43. |
| 694 |
| Nkwocha FG, Akinyamoju CA, Ogbode SO, Lawal FB. Management of dental caries with atraumatic restorative treatment under field condition in primary schools in Oyo State, Nigeria. *Ann Ib Postgrad Med* 2019; **17**: 75–80. |
| 695 |
| Akeredolu P, Akinboboye Bolanle O, Gbotolorun Olalekan M, Emeka CI, Adesida AA. Management of dental anxiety: a survey of Nigerian dentists. *Sahel medical journal (Print)* 2014; **17**: 159–63. |
| 696 |
| Nasir SB, Shuaibu IY, Labaran SA, Inusa A. Management of Deep Neck Space Infections in a Tertiary Center in North West Nigeria. *Niger J Surg* 2019; **25**: 183–7. |
| 697 |
| Akinmoladun V, Ademola S, Olusanya A. Management of cleft lip and palate in Nigeria: A survey. *Nigerian Journal of Clinical Practice* 2017; **20**: 1355–9. |
| 698 |
| Durgesh BH, Prakash P, Ramakrishnaiah R, Subashchandra Phulari B, Al Kheraif AA. Malocclusion Pattern (Angle’s) in Mauritian Orthodontic Patients. *ISRN Dent* 2012; **2012**: 210306. |
| 699 |
| Utomi Il, Onyeaso Co. Malocclusion and orthodontic treatment need of patients attending the Lagos University Teaching Hospital, Lagos, Nigeria. *Odonto-stomatologie tropicale = Tropical dental journal* 2015; **38**: 23–30. |
| 700 |
| Muasya MK, Ng’Ang’A PM, Opinya GN, Macigo FG. Malocclusion and orthodontic treatment need among 12-15-year-old children in nairobi. *East African Medical Journal* 2012; **89**: 39–44. |
| 701 |
| Dacosta O, Aikins E, Isiekwe G, Adediran V. Malocclusion and early orthodontic treatment requirements in the mixed dentitions of a population of Nigerian children. *Journal of Orthodontic Science* 2016; **5**: 81–6. |
| 702 |
| Folayan MO, El Tantawi M, Oginni AB, Alade M, Adeniyi A, Finlayson TL. Malnutrition, enamel defects, and early childhood caries in preschool children in a sub-urban Nigeria population. *PLoS ONE* 2020; **15**. DOI:[10.1371/journal.pone.0232998](https://doi.org/10.1371/journal.pone.0232998). |
| 703 |
| Bah AT, Bah A, Diallo OR, Kourouma A. Malformations congénitales oro-cervico-faciales au service d’odonto-stomatologie et chirurgie maxillo-faciale de l’hôpital national Donka. *African Journal of Dentistry and Implantology* 2020; **16**: 18–23. |
| 704 |
| Lawal FB, Taiwo JO. Making a Case for Formal School-Based Oral Health Promotion: Oral Health Knowledge, Attitude and Practices of Pupils in Ibadan, Nigeria. *International Quarterly of Community Health Education* 2018; **38**: 99–105. |
| 705 |
| Msyamboza KP, Phale E, Namalika JM, *et al.* Magnitude of dental caries, missing and filled teeth in Malawi: National Oral Health Survey. *BMC Oral Health* 2016; **16**. DOI:[10.1186/s12903-016-0190-3](https://doi.org/10.1186/s12903-016-0190-3). |
| 706 |
| Aynalem YA, Alamirew G, Shiferaw WS. Magnitude of Dental Caries and Its Associated Factors Among Governmental Primary School Children in Debre Berhan Town, North-East Ethiopia. *Pediatric Health Med Ther* 2020; **11**: 225–33. |
| 707 |
| Musalam K, Sohal KS, Owibingire SS, Kileo B. Magnitude and Determinants of Dental Anxiety among Adult Patients Attending Public Dental Clinics in Dar-Es-Salaam, Tanzania. *International Journal of Dentistry* 2021; **2021**. DOI:[10.1155/2021/9965204](https://doi.org/10.1155/2021/9965204). |
| 708 |
| Kouassi Zegbeh-N’guessan E, Djémi EM, Pornan Bérété IJ, Greberet  Crezoit E. Luxations temporo-mandibulaires au CHU de Bouaké: Aspects épidémiologiques, cliniques et thérapeutiques. *Rev int sci méd (Abidj)* 2022; **24**: 151–6. |
| 709 |
| de Berker HT, Honeyman C, Patel V, *et al.* Long-term outcomes of mandibular reconstruction using non-vascularised rib grafts following large ameloblastoma resection in Ethiopia. *Oral Surgery* 2022; **15**: 251–60. |
| 710 |
| Engh MS, Muhoozi GKM, Ngari M, *et al.* Long-Term Effects of a Randomized Maternal Education Trial in Rural Uganda: Implications for Child Oral Health. *American Journal of Tropical Medicine and Hygiene* 2022; **107**: 939–47. |
| 711 |
| Amien F, Myburgh NG, Butler N. Location of community pharmacies and prevalence of oral conditions in the Western Cape Province. *Health SA Gesondheid* 2013; **18**. DOI:[10.4102/hsag.v18i1.687](https://doi.org/10.4102/hsag.v18i1.687). |
| 712 |
| Anyanechi C, Osunde O, Saheeb B. Locally advanced orofacial malignancy: Synopsis of inoperable lesions at an urban tertiary health facility in Nigeria. *Nigerian Journal of Clinical Practice* 2020; **23**: 691–6. |
| 713 |
| Aikins EA, Onyeaso CO, Ukeghesen GE. Lip Pattern of Orthodontic Patients Seen at the Orthodontic Clinic of University of Port Harcourt Teaching Hospital; Port Harcourt. *port harcourt med J* 2013; **7**: 111–7. |
| 714 |
| Isiekwe GI, daCosta OO, Isiekwe MC. Lip dimensions of an adult Nigerian population with normal occlusion. *J Contemp Dent Pract* 2012; **13**: 188–93. |
| 715 |
| Diouf M, Boetsch G, Cissé D, Tal-Dia A, Bonfil JJ. Lifestyles and oral health in Fulani populations of Ferlo in Senegal. *Medecine et sante tropicales* 2012; **22**: 187–92. |
| 716 |
| Ba B, Keita K, Coulibaly A, *et al.* Les améloblastomes des maxillaires au centre hopsitalier universitaire d’odontosmatologie de Bamako. *Mali méd (En ligne)* 2017; **32**: 1–6. |
| 717 |
| Millogo M, Ouedraogo RW-L, Ily V, Konsem T, Ouedraogo D. Labial lesions by human bite. *Journal of Oral Medicine and Oral Surgery* 2018; **24**: 153–6. |
| 718 |
| Oderinu OH, Sede MA, Oginni AO, *et al.* Knowledge, diagnosis and management of dentine hypersensitivity: a national survey of dentists in Nigeria. *International Dental Journal* 2017; **67**: 287–93. |
| 719 |
| Sohal KS, Moris RL, Moshy JR. Knowledge, attitudes, and practices related to COVID-19 among patients attending public dental clinics in Tanzania: A cross-sectional study. *PLoS ONE* 2022; **17**. DOI:[10.1371/journal.pone.0276620](https://doi.org/10.1371/journal.pone.0276620). |
| 720 |
| Ramphoma KJ, Naidoo S. Knowledge, attitudes and practices of oral health care workers in Lesotho regarding the management of patients with oral manifestations of HIV/AIDS. *SADJ : journal of the South African Dental Association = tydskrif van die Suid-Afrikaanse Tandheelkundige Vereniging* 2014; **69**: 446, 448–53. |
| 721 |
| GI O, AJ O. Knowledge, attitude, and practices of dental patients presenting at a secondary health care facility in southern Nigeria towards covid-19. *Yenagoa Medical Journal* 2021; **3**: 172–82. |
| 722 |
| Lawal FB, Fagbule OF. Knowledge of School-Going Adolescents About the Oral Effects of Tobacco Usage in Ibadan, Southwest Nigeria. *International Quarterly of Community Health Education* 2020; **40**: 337–43. |
| 723 |
| Eigbobo JO, Aikins EA, Onyeaso CO. Knowledge of preventive child oral healthcare among expectant mothers in Port Harcourt, Nigeria. *Pediatric Dental Journal* 2013; **23**: 1–7. |
| 724 |
| Kabali TM, Mumghamba EG. Knowledge of Periodontal Diseases, Oral Hygiene Practices, and Self-Reported Periodontal Problems among Pregnant Women and Postnatal Mothers Attending Reproductive and Child Health Clinics in Rural Zambia. *International Journal of Dentistry* 2018; **2018**. DOI:[10.1155/2018/9782092](https://doi.org/10.1155/2018/9782092). |
| 725 |
| Wassihun B, Ayinalem A, Beyene K. Knowledge of oral health during pregnancy and associated factors among pregnant mothers attending antenatal care at South Omo Zone public hospitals, Southern Ethiopia, 2021. *PLoS ONE* 2022; **17**. DOI:[10.1371/journal.pone.0273795](https://doi.org/10.1371/journal.pone.0273795). |
| 726 |
| Koyio LN, Van Der Sanden WJM, Dimba EO, *et al.* Knowledge of Nairobi East District Community Health Workers concerning HIV-related orofacial lesions and other common oral lesions. *BMC Public Health* 2014; **14**. DOI:[10.1186/1471-2458-14-1066](https://doi.org/10.1186/1471-2458-14-1066). |
| 727 |
| Egbunah UP, Adekunle AA. Knowledge of Environmental and Genetic Risk Factors for Cleft Lip and Palate among Dwellers of a Suburban Community in Nigeria. *West Afr J Med* 2022; **39**: 479–85. |
| 728 |
| Bamigboye SA, Dosumu OO, Ogunrinde JT. Knowledge of Consequences of Missing Teeth in Patients Attending Prosthetic Clinic in U.C.H. Ibadan. *Annals of Ibadan Postgraduate Medicine* 2014; **12**: 42–8. |
| 729 |
| Taiwo OO, Panas RM. Knowledge of community pharmacists regarding oral health care in Plateau State, Northern Nigeria. *Journal of International Oral Health* 2017; **9**: 222–7. |
| 730 |
| Benoist FL, Ndiaye FG, Faye B, Bane K, Ngom PI, Ndong PM. Knowledge of and management attitude regarding dentin hypersensitivity among dentists from a West African country. *The journal of contemporary dental practice* 2014; **15**: 86–91. |
| 731 |
| Hoque MEH Hendry, Lukhozi V. Knowledge and Utilisation of Expanded Functions of Oral Hygiene and Barriers to Successful Implementation in Public Healthcare Services in Gauteng. *S Afr j infect dis (Online)* 2012; **27**: 126–9. |
| 732 |
| Gbotolorun OM, Ayodele ASO, Olojede ACO, Adamson OO, Emeka CI, Amao AT. Knowledge and screening practices for oral cancers amongst general dental practitioners in Lagos, Nigeria. *African Journal of Biomedical Research* 2014; **17**: 69–73. |
| 733 |
| Nyorobi JM, Carneiro LC, Kabulwa MN. Knowledge and practices on periodontal health among adults, Misungwi, Tanzania. *International Journal of Dentistry* 2018; **2018**. DOI:[10.1155/2018/7189402](https://doi.org/10.1155/2018/7189402). |
| 734 |
| Agbor AM, Naidoo S. Knowledge and practice of traditional healers in oral health in the Bui Division, Cameroon. *Journal of Ethnobiology and Ethnomedicine* 2011; **7**. DOI:[10.1186/1746-4269-7-6](https://doi.org/10.1186/1746-4269-7-6). |
| 735 |
| Onagaumah JJ, Ehizele AO, Olubamiwa TO. Knowledge and perception of dentinal hypersensitivity and its associated factors among dental resident doctors in university of Benin Teaching Hospital (UBTH). *Nigerian Journal of Periodontal Research* 2022; **3**: 56–9. |
| 736 |
| Oyetola EO, Oyewole T, Adedigba M, Aregbesola ST, Umezudike K, Adewale A. Knowledge and awareness of medical doctors, medical students and nurses about dentistry in Nigeria. *Pan African Medical Journal* 2016; **23**. DOI:[10.11604/pamj.2016.23.172.7696](https://doi.org/10.11604/pamj.2016.23.172.7696). |
| 737 |
| Enabulele J, Omo J, Ibhawoh L. Knowledge and attitude to dental implant placement amongst a group of Nigerian dentist. *Afr Health Sci* 2022; **22**: 678–89. |
| 738 |
| Olatosi OO, Iwuala SO, Isiekwe GI, Oredugba FA, Adenaike AS, Oluwo AO. Knowledge and attitude of some nigerian school teachers on the emergency management of avulsed permanent incisor. *J West Afr Coll Surg* 2013; **3**: 30–52. |
| 739 |
| Agbelusi GA, Adeola HA, Ameh PO. Knowledge and Attitude of PLWHA concerning oral lesions of HIV/AIDS among patients of PEPFAR clinic in Lagos University Teaching Hospital (LUTH) Lagos, Nigeria. *The Nigerian postgraduate medical journal* 2011; **18**: 120–5. |
| 740 |
| Kamulegeya A, William B, Rwenyonyi CM. Knowledge and Antibiotics Prescription Pattern among Ugandan Oral Health Care Providers: A Cross-sectional Survey. *J Dent Res Dent Clin Dent Prospects* 2011; **5**: 61–6. |
| 741 |
| Simiyu BN, Butt F, Dimba EA, *et al.* Keratocystic odontogenic tumours of the jaws and associated pathologies: A 10-year clinicopathologic audit in a referral teaching hospital in Kenya. *Journal of Cranio-Maxillofacial Surgery* 2013; **41**: 230–4. |
| 742 |
| Titinchi F. Juvenile ossifying fibroma of the maxillofacial region: Analysis of clinico-pathological features and management. *Medicina Oral Patologia Oral y Cirugia Bucal* 2021; **26**: e590–7. |
| 743 |
| Ba B, Coulibaly DT, Dicko Traoré F, *et al.* [Isolated mandibular fractures study’s in the CHU-OS of Bamako: 55 cases]. *Mali Med* 2014; **29**: 56–60. |
| 744 |
| Anyanechi CE. Isolated favorable compound mandibular body fractures: A retrospective review of two cohorts of patients based on the timing of treatment. *Dent Traumatol* 2022. DOI:[10.1111/edt.12786](https://doi.org/10.1111/edt.12786). |
| 745 |
| Yengopal V, Ayodeji Esan T, Joosab Z. Is there an association between viral load, CD4 count, WHO staging, and dental caries in HIV-positive children? *International Journal of Paediatric Dentistry* 2021; **31**: 231–7. |
| 746 |
| Folayan MO, Finlayson T, Oginni AB, Alade MA, Adeniyi AA, El Tantawi M. Is oral rehydration therapy associated with early childhood caries in children resident in Ile-Ife, Osun State, Nigeria? *International Journal of Paediatric Dentistry* 2022. DOI:[10.1111/ipd.13016](https://doi.org/10.1111/ipd.13016). |
| 747 |
| John CN, Stephen LX, Joyce Africa CW. Is human immunodeficiency virus (HIV) stage an independent risk factor for altering the periodontal status of HIV-positive patients? A South African study. *BMC Oral Health* 2013; **13**. DOI:[10.1186/1472-6831-13-69](https://doi.org/10.1186/1472-6831-13-69). |
| 748 |
| Adeyemi MF, Adeyemo WL, Ogunlewe MO, Ladeinde AL. Is healing outcome of 2 weeks intermaxillary fixation different from that of 4 to 6 weeks intermaxillary fixation in the treatment of mandibular fractures? *Journal of Oral and Maxillofacial Surgery* 2012; **70**: 1896–902. |
| 749 |
| Folayan MO, El Tantawi M, Oginni O, Arowolo O. Is chronotype profile a risk indicator for caries in children and adolescents in sub-urban Nigeria? *International Journal of Paediatric Dentistry* 2021; **31**: 627–33. |
| 750 |
| Fashina A, Busch T, Young M, *et al.* Investigating the relationship between cancer and orofacial clefts using GWAS significant loci for cancers: A case-control and case-triad study. *Front Oral Health* 2022; **3**: 915361. |
| 751 |
| Peerbhay F, Elsheikhomer AM. Intranasal Midazolam Sedation in a Pediatric Emergency Dental Clinic. *Anesth Prog* 2016; **63**: 122–30. |
| 752 |
| Osinaike BB, Gbolahan OO, Olusanya AA. Intra-Operative Airway Management in Patients with Maxillofacial Trauma having Reduction and Immobilization of Facial Fractures. *Niger J Surg* 2015; **21**: 26–30. |
| 753 |
| Braimoh M, Ogunbodede E, Adeniyi A. Integration of oral health into primary health care system: Views of primary health care workers in Lagos State, Nigeria. *Journal of Public Health in Africa* 2014; **5**: 35–9. |
| 754 |
| Varenne B. Integrating Oral Health with Non-Communicable Diseases as an Essential Component of General Health: WHO’s Strategic Orientation for the African Region. *Journal of Dental Education* 2015; **79**: S32–7. |
| 755 |
| Kaguru G, Ayah R, Mutave R, Mugambi C. Integrating Oral Health into Primary Health Care: A Systematic Review of Oral Health Training in Sub-Saharan Africa. *Journal of Multidisciplinary Healthcare* 2022; **15**: 1361–7. |
| 756 |
| Diouf JS, Ngom PI, Fadiga MS, *et al.* Influence of tonsil size on sagittal cephalometric measurements. *Int Orthod* 2015; **13**: 149–63. |
| 757 |
| Sekele IB, Naert I, Lutula PS, *et al.* Influence of the removable partial denture acrylic resin on oral health and quality of life. *Odonto-stomatologie tropicale = Tropical dental journal* 2016; **39**: 36–46. |
| 758 |
| Ogbeifun JO, Fomete B, Ononiwu CN. Influence of positions on the incidence and severity of middle third facial fractures in vehicular and motorcycle crashes seen in a Tertiary Health Facility. *Niger J Dent Res* 2020; **5**: 161–6. |
| 759 |
| Nepaul P, Mahomed O. Influence of parents’ oral health knowledge and attitudes on oral health practices of children (5-12 years) in a rural school in KwaZulu-Natal, South Africa. *Journal of International Society of Preventive and Community Dentistry* 2020; **10**: 605–12. |
| 760 |
| Denloye OO, Iyun OI, Popoola BO. Influence of Parental Socioeconomic Status on Caries Prevalence among Children Seen at the University College Hospital; Ibadan. *Annals of Ibadan Postgraduate Medicine* 2013; **11**: 81–6. |
| 761 |
| Ramla S, Sharma V, Patel M. Influence of cancer treatment on the Candida albicans isolated from the oral cavities of cancer patients. *Support Care Cancer* 2016; **24**: 2429–36. |
| 762 |
| Anyanechi CE, Saheeb BD. Inflammatory morbidity due to compound mandibular body fractures: Does it have a relationship with treatment outcome? *Medical Principles and Practice* 2015; **24**: 238–43. |
| 763 |
| Fomete B, Agbara R, Adeola DS, Osunde DO. Inflammatory and reactive lesions of the orofacial region in an African tertiary health setting. *Sahel medical journal (Print)* 2019; **22**: 96–101. |
| 764 |
| Benedict Odhiambo Otieno, Eunice Njeri Kihara, Bernard Nzioka Mua. Infection Control Practices Among Private Practicing Dentists in Nairobi During the Pre-coronavirus Disease 2019 Period. *Frontiers in oral health* 2020; **1**: 587603. |
| 765 |
| Folayan MO, El Tantawi M, Chukwumah NM, *et al.* Individual and familial factors associated with caries and gingivitis among adolescents resident in a semi-urban community in South-Western Nigeria. *BMC Oral Health* 2021; **21**. DOI:[10.1186/s12903-021-01527-x](https://doi.org/10.1186/s12903-021-01527-x). |
| 766 |
| Osunde O, Saheeb B, Bassey G. Indications and risk factors for complications of lower third molar surgery in a nigerian teaching hospital. *Ann Med Health Sci Res* 2014; **4**: 938–42. |
| 767 |
| Akinbami BO, Akadiri OA. Indications and outcome of mandibular condylar and ramus surgeries. *Niger J Surg* 2014; **20**: 69–74. |
| 768 |
| Ani CC, Adoga AA, Tawe GS. Incidental maxillary sinus findings on cranial computerized tomographic scan in a tropical setting. *J West Afr Coll Surg* 2016; **6**: 39–51. |
| 769 |
| Menakaya IN, Oderinu OH, Adegbulugbe IC, Shaba OP. Incidence of postoperative pain after use of calcium hydroxide mixed with normal saline or 0.2% chlorhexidine digluconate as intracanal medicament in the treatment of apical periodontitis. *Saudi Dent J* 2015; **27**: 187–93. |
| 770 |
| Frimpong P, Nguyen TTH, Sodnom-Ish B, *et al.* Incidence and management of mandibular fractures in a low-resource health facility in Ghana. *Journal of the Korean Association of Oral and Maxillofacial Surgeons* 2021; **47**: 432–7. |
| 771 |
| Grobler SR, Majeed A, Moola MH, Rossouw RJ, van Wyk Kotze T. In vivo Spectrophotometric Assessment of the Tooth Whitening Effectiveness of Nite White 10% with Amorphous Calcium Phosphate, Potassium Nitrate and Fluoride, Over a 6-month Period. *Open Dent J* 2011; **5**: 18–23. |
| 772 |
| Mulder-van Staden S, Holmes H, Hille J. In vivo investigation of diode laser application on red complex bacteria in non-surgical periodontal therapy: a split-mouth randomised control trial. *Sci Rep* 2020; **10**: 21311. |
| 773 |
| Molete M, Stewart A, Igumbor J. Implementation fidelity of school oral health programs at a District in South Africa. *PLoS ONE* 2020; **15**. DOI:[10.1371/journal.pone.0241988](https://doi.org/10.1371/journal.pone.0241988). |
| 774 |
| Kimmie-Dhansay F, Pontes CC, Chikte UME, *et al.* Impacts of tooth loss on ohrqol in an adult population in cape town, south africa. *International Journal of Environmental Research and Public Health* 2021; **18**. DOI:[10.3390/ijerph18094989](https://doi.org/10.3390/ijerph18094989). |
| 775 |
| Baidoo K, Blankson P-K, Kwakye S, *et al.* Impacted dentures in the pharynx and esophagus-Implication for advocacy. *Annals of African Medicine* 2022; **21**: 274–7. |
| 776 |
| Obilade OA, Sanu OO, Costa OO. Impact of three malocclusion traits on the quality of life of orthodontic patients. *Int Orthod* 2016; **14**: 366–85. |
| 777 |
| Mayunga GM, Lutula PS, Sekele IB, Bolenge I, Kumpanya N, Nyengele K. Impact of the edentulousness on the quality of life related to the oral health of the Congolese. *Odonto-stomatologie tropicale = Tropical dental journal* 2015; **38**: 31–6. |
| 778 |
| Umeh OD, Utomi IL, Isiekwe IG, Aladenika ET. Impact of the coronavirus disease 2019 pandemic on orthodontic patients and their attitude to orthodontic treatment. *Am J Orthod Dentofacial Orthop* 2021; **159**: e399–409. |
| 779 |
| Melo P, Fine C, Malone S, Taylor S. Impact of the Brush Day & Night Programme on Oral Health Knowledge and Behaviour in Children. *Int Dent J* 2021; **71**: S4–14. |
| 780 |
| Olabu BO, Okoro DO, Thigiti JM, Oramisi VA. Impact of socio-cultural practice of infant/young child gum lancing during teething. *Journal of Clinical Pediatric Dentistry* 2013; **37**: 355–9. |
| 781 |
| Akpasa IO, Yemitan TA, Ogunbanjo BO, Oyapero A. Impact of severity of malocclusion and self-perceived smile and dental aesthetics on self-esteem among adolescents. *Journal of the World Federation of Orthodontists* 2022; **11**: 120–4. |
| 782 |
| Khan S, Chikte U, Omar R. Impact of Removable Partial Dental Prostheses on the Oral Health-Related Quality of Life of a South African Cohort with Varied Distributions of Missing Posterior Teeth. *Journal of Prosthodontics* 2019; **28**: e434–9. |
| 783 |
| Ellapen TJ, Narsigan S, van Herdeen HJ, Pillay K, Rugbeer N. Impact of poor dental ergonomical practice. *SADJ : journal of the South African Dental Association = tydskrif van die Suid-Afrikaanse Tandheelkundige Vereniging* 2011; **66**: 272, 274–7. |
| 784 |
| Lawal FB, Fagbule OF, Akinloye SJ, Lawal TA, Oke GA. Impact of oral hygiene habits on oral health-related quality of life of in-school adolescents in Ibadan, Nigeria. *Front Oral Health* 2022; **3**: 979674. |
| 785 |
| Oyedele TA, Fadeju AD, Adeyemo YI, Nzomiwu CL, Ladeji AM. Impact of oral hygiene and socio-demographic factors on dental caries in a suburban population in Nigeria. *European Archives of Paediatric Dentistry* 2018; **19**: 155–61. |
| 786 |
| Lawal FB, Taiwo JO, Oke GA. Impact of Oral Health on The Quality of Life of Elementary School Teachers. *Ethiopian journal of health sciences* 2015; **25**: 217–24. |
| 787 |
| Braimah RO, Ndukwe KC, Owotade JF, Aregbesola SB. Impact of oral antibiotics on health-related quality of life after mandibular third molar surgery: An observational study. *Niger J Clin Pract* 2017; **20**: 1189–94. |
| 788 |
| Anthony SN, Zimba K, Subramanian B. Impact of Malocclusions on the Oral Health-Related Quality of Life of Early Adolescents in Ndola, Zambia. *International Journal of Dentistry* 2018; **2018**. DOI:[10.1155/2018/7920973](https://doi.org/10.1155/2018/7920973). |
| 789 |
| Masumo R, Birungi N, Bårdsen A, Fadnes LT, Åstrom AN. Impact of low birthweight on early childhood caries in 6-36 months old infants in Uganda: A cross-sectional study. *Acta Odontologica Scandinavica* 2014; **72**: 312–20. |
| 790 |
| Chukwumah NM, Folayan MO, Oziegbe EO, Umweni AA. Impact of dental caries and its treatment on the quality of life of 12- to 15-year-old adolescents in Benin, Nigeria. *International Journal of Paediatric Dentistry* 2016; **26**: 66–76. |
| 791 |
| Uti OG, Sofola O. Impact of an educational intervention on smoking counseling practice among Nigerian dentists and dental students. *Nigerian Journal of Clinical Practice* 2015; **18**: 75–9. |
| 792 |
| Agbaje HO, Kolawole KA, Otuyemi OD. Impact des malocclusions sur la qualité de vie en lien avec la santé bucco-dentaire chez les patients nécessitant un traitement orthodontique. *Odonto-stomatol trop* 2018; **41**: 27–36. |
| 793 |
| Coker MO, Mongodin EF, El-Kamary SS, *et al.* Immune status, and not HIV infection or exposure, drives the development of the oral microbiota. *Scientific Reports* 2020; **10**. DOI:[10.1038/s41598-020-67487-4](https://doi.org/10.1038/s41598-020-67487-4). |
| 794 |
| Simon EN, Merkx MA, Kalyanyama BM, Shubi FM, Stoelinga PJ. Immediate reconstruction of the mandible after resection for aggressive odontogenic tumours: a cohort study. *Int J Oral Maxillofac Surg* 2013; **42**: 106–12. |
| 795 |
| Sangaré AD, Samba M, Bourgeois D. Illness-related behaviour and sociodemographic determinants of oral health care use in Dabou, Côte d’Ivoire. *Community Dental Health* 2012; **29**: 78–84. |
| 796 |
| Oyedele TA, Folayan MO, Oziegbe EO. Hypomineralised second primary molars: Prevalence, pattern and associated co morbidities in 8- to 10-year-old children in Ile-Ife, Nigeria. *BMC Oral Health* 2016; **16**. DOI:[10.1186/s12903-016-0225-9](https://doi.org/10.1186/s12903-016-0225-9). |
| 797 |
| Yamori M, Njelekela M, Mtabaji J, Yamori Y, Bessho K. Hypertension, periodontal disease, and potassium intake in nonsmoking, nondrinker African women on no medication. *International Journal of Hypertension* 2011; **2011**. DOI:[10.4061/2011/695719](https://doi.org/10.4061/2011/695719). |
| 798 |
| Kane AS, Guirassy ML, Diallo B, *et al.* Hygiène bucco-dentaire des militaires des unités d’élites des forces de défense et de sécurité du Mali. *Mali Médical* 2022; **28**: 30–4. |
| 799 |
| Pengpid S, Peltzer K. Hygiene behaviour and associated factors among in-school adolescents in nine african countries. *International Journal of Behavioral Medicine* 2011; **18**: 150–9. |
| 800 |
| Wood NH, Makua KS, Lebelo RL, *et al.* Human Papillomavirus Prevalence in Oral and Oropharyngeal Rinse and Gargle Specimens of Dental Patients and of an HIV-Positive Cohort from Pretoria, South Africa. *Advances in Virology* 2020; **2020**. DOI:[10.1155/2020/2395219](https://doi.org/10.1155/2020/2395219). |
| 801 |
| Aboagye E, Agyemang-Yeboah F, Duduyemi BM, Obirikorang C. Human papillomavirus detection in head and neck squamous cell carcinomas at a tertiary hospital in Sub-Saharan Africa. *Scientific World Journal* 2019; **2019**. DOI:[10.1155/2019/2561530](https://doi.org/10.1155/2019/2561530). |
| 802 |
| Woto-Gaye G, M’Farrej MK, Doh K, *et al.* [Human papilloma viruses: other risk factor of head and neck carcinoma]. *Bull Soc Pathol Exot* 2016; **109**: 160–4. |
| 803 |
| Lawal FB, Taiwo JO, Arowojolu MO. How valid are the psychometric properties of the oral health impact profile-14 measure in adult dental patients in Ibadan, Nigeria? *Ethiopian journal of health sciences* 2014; **24**: 235–42. |
| 804 |
| Diendéré J, Bosu WK, Ouédraogo W-LR, *et al.* How alcohol and/or tobacco use and raised glycemia are associated with oral hygiene practices among Burkinabè adults: Evidence from the first national non-communicable disease risk factors survey. *Preventive Medicine Reports* 2022; **28**. DOI:[10.1016/j.pmedr.2022.101854](https://doi.org/10.1016/j.pmedr.2022.101854). |
| 805 |
| Anyanechi CE, Saheeb BD. Honey and wound dehiscence: a study of surgical wounds in the mandibular bed. *Niger J Clin Pract* 2015; **18**: 251–5. |
| 806 |
| Abe EO, Kolude B, Adeyemi BF. Hiv testing in dental practice: perception and attitude of dentists in southwestern Nigeria. *African journal of medicine and medical sciences* 2014; **43**: 201–8. |
| 807 |
| Pavlinac PB, Hawes SE, Gottlieb GS, *et al.* HIV shedding in the oral cavity: An assessment of HIV type, immunovirologic,demographic and oral factors. *Sexually Transmitted Infections* 2012; **88**: 45–50. |
| 808 |
| Rajonson N, Meless D, Ba B, *et al.* High prevalence of dental caries among HIV-infected children in West Africa compared to uninfected siblings. *Journal of Public Health Dentistry* 2017; **77**: 234–43. |
| 809 |
| Addison MJ, Rivett MO, Phiri OL, *et al.* ‘Hidden Hot Springs’ as a Source of Groundwater Fluoride and Severe Dental Fluorosis in Malawi. *Water (Switzerland)* 2021; **13**. DOI:[10.3390/w13081106](https://doi.org/10.3390/w13081106). |
| 810 |
| Azodo CC, Umoh AO. Herpes labialis and Nigerian dental health care providers: Knowledge, attitudes, behaviors, and refusal to treat. *BMC Health Services Research* 2015; **15**. DOI:[10.1186/s12913-015-1023-9](https://doi.org/10.1186/s12913-015-1023-9). |
| 811 |
| Azodo CC, Umoh AO. Herpes labialis among dental healthcare providers in Nigeria. *Indian journal of dentistry* 2015; **6**: 116–20. |
| 812 |
| Yimenu DK, Adelo ES, Siraj EA, *et al.* Health professionals oral health knowledge and practice: unleashing the hidden challenges. *Journal of Multidisciplinary Healthcare* 2020; **13**: 459–69. |
| 813 |
| Ayo-Yusuf IJ, Ayo-Yusuf OA, Olutola BG. Health insurance, socio-economic position and racial disparities in preventive dental visits in South Africa. *International Journal of Environmental Research and Public Health* 2013; **10**: 178–91. |
| 814 |
| Adekunle AA, James O, Adeyemo WL. Health Information Seeking Through Social Media and Search Engines by Parents of Children With Orofacial Cleft in Nigeria. *Cleft Palate-Craniofacial Journal* 2020; **57**: 444–7. |
| 815 |
| Nabukenya J, Hadlock TA, Arubaku W. Head and Neck Squamous Cell Carcinoma in Western Uganda: Disease of Uncertainty and Poor Prognosis. *OTO Open* 2018; **2**: 2473974X18761868. |
| 816 |
| Adriane K, Jeff O. Head and Neck Cancers Case Control Study of HIV Positive Compared to Negative Patients in a Ugandan Population Sample. *Int J Clin Oral Maxillofac Surg* 2017; **3**: 20–5. |
| 817 |
| Sombié R, Tiendrébéogo AJF, Guiguimdé WPL, *et al.* Halitosis: Multidisciplinary diagnostic and therapeutic approaches. *Pan African Medical Journal* 2018; **30**. DOI:[10.11604/pamj.2018.30.201.10951](https://doi.org/10.11604/pamj.2018.30.201.10951). |
| 818 |
| Arinola JE, Olukoju OO. Halitosis amongst students in tertiary institutions in Lagos state. *African Health Sciences* 2012; **12**: 473–8. |
| 819 |
| Rango T, Kravchenko J, Atlaw B, *et al.* Groundwater quality and its health impact: An assessment of dental fluorosis in rural inhabitants of the Main Ethiopian Rift. *Environment International* 2012; **43**: 37–47. |
| 820 |
| Gbadebo AM. Groundwater fluoride and dental fluorosis in southwestern Nigeria. *Environmental Geochemistry and Health* 2012; **34**: 597–604. |
| 821 |
| Lawal FB. Global self-rating of oral health as summary tool for oral health evaluation in low-resource settings. *J Int Soc Prev Community Dent* 2015; **5**: S1-6. |
| 822 |
| Blignaut E, Rossouw TM, Becker PJ, Mavuso DS, Feucht UD. Gingival recession and localized aggressive periodontitis among HIV-infected children and adolescents receiving antiretroviral therapy. *Pediatric Infectious Disease Journal* 2019; **38**: E112–5. |
| 823 |
| Azodo CC, Agbor AM. Gingival health and oral hygiene practices of schoolchildren in the North West Region of Cameroon. *BMC Research Notes* 2015; **8**. DOI:[10.1186/s13104-015-1350-2](https://doi.org/10.1186/s13104-015-1350-2). |
| 824 |
| Pesson DM, Bakou OD, Didia EL, Kouame A, Blohoua MR, Djeredou KB. Gingival displacement techniques in daily practice. Survey among dental surgeons in Abidjan, Ivory Coast. *Odonto-stomatologie tropicale = Tropical dental journal* 2015; **38**: 25–32. |
| 825 |
| Fomete B, Samaila MOA, Omeje KU, *et al.* Giant Ameloblastoma and the Risk of Recurrence: Experiences from a Tertiary Hospital. *West Afr J Med* 2022; **39**: 350–4. |
| 826 |
| Gowans LJJ, Comnick CL, Mossey PA, *et al.* Genome-Wide Scan for Parent-of-Origin Effects in a sub-Saharan African Cohort With Nonsyndromic Cleft Lip and/or Cleft Palate (CL/P). *Cleft Palate Craniofac J* 2022; **59**: 841–51. |
| 827 |
| Okoturo E, Osasuyi A, Opaleye T. Genetic Polymorphism of Head and Neck Cancers in African Populations: A Systematic Review. *OTO Open* 2020; **4**: 2473974X20942202. |
| 828 |
| Olawole WO, Kanmodi KK. Generally, the elderly are more dentally anxious than younger folks: A short report from a clinical study in Nigeria. *Journal of Pain Management* 2020; **13**: 29–34. |
| 829 |
| Folayan MO, Kolawole KA, Onyejaka NK, Agbaje HO, Chukwumah NM, Oyedele TA. General anxiety, dental anxiety, digit sucking, caries and oral hygiene status of children resident in a semi-urban population in Nigeria. *BMC Oral Health* 2018; **18**. DOI:[10.1186/s12903-018-0529-z](https://doi.org/10.1186/s12903-018-0529-z). |
| 830 |
| Olaleye AO. Gender distribution of Amalgam restoration and treatment pattern in regular attendees of a Teaching Hospital in Nigeria. *World Journal of Dentistry* 2014; **5**: 109–12. |
| 831 |
| Fatusi O, Ogunbodede E, Sowole C, Folayan M. Gaps in oral health-care service provision systems for children in Nigeria: A case study of a tertiary health institution. *Indian Journal of Dental Research* 2018; **29**: 622–6. |
| 832 |
| Jordan AR, Pottbrock M, Gängler P, Zimmer S. GambiaDentCare - Evaluation of a primary oral health-care programme in West Africa. *Gesundheitswesen* 2011; **73**: 849–52. |
| 833 |
| Turton M, Africa CWJ. Further evidence for periodontal disease as a risk indicator for adverse pregnancy outcomes. *International Dental Journal* 2017; **67**: 148–56. |
| 834 |
| Umesi DC, Oremosu OA, Makanjuola JO, Nwachukwu NC. Frequency and distribution of teeth treated by single- and multiple- visit root canal treatment in a Nigerian population by differently skilled operators. *Odonto-stomatologie tropicale = Tropical dental journal* 2016; **39**: 56–64. |
| 835 |
| Agbara R, Fomete B, Omeje KU. Fractures of the Mandible: Epidemiological Study of 519 Nigerian Cases. *J West Afr Coll Surg* 2021; **11**: 26–32. |
| 836 |
| Effiom OA, Adeyemo WL, Soyele OO. Focal Reactive lesions of the Gingiva: An Analysis of 314 cases at a tertiary Health Institution in Nigeria. *Niger Med J* 2011; **52**: 35–40. |
| 837 |
| Rango T, Vengosh A, Jeuland M, *et al.* Fluoride exposure from groundwater as reflected by urinary fluoride and children’s dental fluorosis in the Main Ethiopian Rift Valley. *Science of the Total Environment* 2014; **496**: 188–97. |
| 838 |
| Demelash H, Beyene A, Abebe Z, Melese A. Fluoride concentration in ground water and prevalence of dental fluorosis in Ethiopian Rift Valley: Systematic review and meta-analysis. *BMC Public Health* 2019; **19**. DOI:[10.1186/s12889-019-7646-8](https://doi.org/10.1186/s12889-019-7646-8). |
| 839 |
| Udoye CI, Jafarzadeh H, Aguwa EN, Habibi M. Flare-up incidence and related factors in Nigerian adults. *J Contemp Dent Pract* 2011; **12**: 120–3. |
| 840 |
| Ikusika OF, Idon PI, Alalade O, Sotunde AO, Akinpelu MA, Igweagu CE. Fixed-prosthodontics in Nigerian private practice settings. *Ibom Medical Journal* 2022; **15**: 116–25. |
| 841 |
| Tamí-Maury I, Coulibaly YI, Cissoko SS, Dao S, Kristensen S. First report of HIV-related oral manifestations in Mali. *Pan African Medical Journal* 2012; **11**: 18. |
| 842 |
| Lasisi TJ, Adisa AO, Olusanya AA. Fibro-osseous lesions of the jaws in Ibadan, Nigeria. *Oral Health Dent Manag* 2014; **13**: 41–4. |
| 843 |
| Fomete B, Osunde OD, Ogbeifun OJ, Adebayo TE. Fibro-osseous Lesions of the Craniofacial Region: A 14-year Experience. *West Indian Medical Journal* 2022; **69**: 539–44. |
| 844 |
| Masumo R, Bardsen A, Mashoto K, Åstrom AN. Feeding practice among 6-36 months old in Tanzania and Uganda: Reliability and relationship with early childhood caries, ECC. *Acta Odontologica Scandinavica* 2013; **71**: 1309–18. |
| 845 |
| Ola D, Gambôa ABO, Folayan MO, Marcenes W. Family structure, socioeconomic position and utilization of oral health services among Nigerian senior secondary school pupils. *Journal of Public Health Dentistry* 2013; **73**: 158–65. |
| 846 |
| Varenne B, Fournet F, Cadot E, *et al.* Family environment and dental health disparities among urban children in Burkina Faso. *Revue d’Epidemiologie et de Sante Publique* 2011; **59**: 385–92. |
| 847 |
| Dosumu EB, Onigbinde OO, Ogunsuji OO. Factors Influencing differences between patient’s self-perceived oral care needs and clinical findings in a Nigerian population. *International Journal of Dentistry and Oral Science* 2019; **6**: 685–90. |
| 848 |
| Lawal FB, Taiwo JO, Oke GA. Factors influencing awareness and attendance of traditional oral health care practices by residents of a peri-urban community in Ibadan, Nigeria. *African Health Sciences* 2015; **15**: 233–9. |
| 849 |
| Diallo B, China E, Makoutode M, Coulibaly C, Diombana ML. [Factors Associated with periodontal disease in pregnant women at the Mother-Child Hospital of Lagune (HOMEL) Cotonou]. *Mali Med* 2014; **29**: 53–8. |
| 850 |
| Ndayisabye H, Ndagijimana A, Biracyaza E, Umubyeyi A. Factors Associated With Oral Cancer Adverse Outcome at the Rwanda Military Hospital, a Retrospective Cross-Sectional Study. *Front Oral Health* 2022; **3**: 844254. |
| 851 |
| Umeh OD, Sanu OO, Utomi IL, Nwaokorie FO. Factors associated with odontogenic bacteraemia in orthodontic patients. *J West Afr Coll Surg* 2016; **6**: 52–77. |
| 852 |
| Nimako-Boateng J, Owusu-Antwi M, Nortey P. Factors affecting dental diseases presenting at the University of Ghana Hospital. *SpringerPlus* 2016; **5**. DOI:[10.1186/s40064-016-3391-y](https://doi.org/10.1186/s40064-016-3391-y). |
| 853 |
| Åstrøm AN, Mbawalla H. Factor structure of health and oral health-related behaviors among adolescents in Arusha, northern Tanzania. *Acta Odontologica Scandinavica* 2011; **69**: 299–309. |
| 854 |
| Makanjuola JO, Umesi CD, Oderinu HO. Facteurs influençant la douleur post-obturation dans le traitement en consultation unique du canal radiculaire en utilisant des instruments endodontiques rotatifs. *Odonto-stomatol trop* 2018; **41**: 17–23. |
| 855 |
| Bancolé Pognon SA, Houinato DS, Djigbenoude O, Agueh JS. Facteurs de risque communs aux maladies bucco-dentaires et aux maladies non transmissibles à Cotonou (Bénin). *Medecine Buccale Chirurgie Buccale* 2013; **19**: 155–9. |
| 856 |
| Huijing MA, Marck KW, Combes J, *et al.* Facial reconstruction in the developing world: A complicated matter. *British Journal of Oral and Maxillofacial Surgery* 2011; **49**: 292–6. |
| 857 |
| Manyama M, Larson JR, Liberton DK, *et al.* Facial morphometrics of children with NON-syndromic orofacial clefts in Tanzania. *BMC Oral Health* 2014; **14**. DOI:[10.1186/1472-6831-14-93](https://doi.org/10.1186/1472-6831-14-93). |
| 858 |
| Taiwo AO, Soyele O, Godwin N, Ibikunle A. Facial fracture management in Northwest Nigeria. *Journal of Surgical Technique and Case Report* 2013; **5**: 65–71. |
| 859 |
| Oginni FO, Oladejo T, Alake DP, Oguntoba JO, Adebayo OF. Facial Bone Fractures in Ile-Ife, Nigeria: An Update on Pattern of Presentation and Care. *Journal of Maxillofacial and Oral Surgery* 2016; **15**: 184–90. |
| 860 |
| Akhiwu BI, Efunkoya AA, Omeje KU, Amole IO, Osunde DO, Isa L. Experience with miniplate osteosynthesis in the management of mandibular fractures in northwest Nigeria. *J West Afr Coll Surg* 2015; **5**: 66–83. |
| 861 |
| Fomete B, Adebayo ET, Oginni FO. Expectations, Experiences and Perceptions of Oral and Maxillofacial Surgery Residents to Training in Nigeria. *European Journal of Dental Education* 2022; **26**: 248–53. |
| 862 |
| Huber AC, Tobias R, Mosler H-J. Evidence-based tailoring of behavior-change campaigns: Increasing fluoride-free water consumption in rural ethiopia with persuasion. *Applied Psychology: Health and Well-Being* 2014; **6**: 96–118. |
| 863 |
| Steyn NP, Temple NJ. Evidence to support a food-based dietary guideline on sugar consumption in South Africa. *BMC Public Health* 2012; **12**. DOI:[10.1186/1471-2458-12-502](https://doi.org/10.1186/1471-2458-12-502). |
| 864 |
| Lecor PA, Dia-Tine S, Gaye PM, *et al.* Évaluation quantitative de la sécrétion et du pouvoir tampon de la salive au cours de la radiothérapie des cancers des voies aéro-digestives supérieures au Sénégals. *Medecine Buccale Chirurgie Buccale* 2013; **19**: 227–33. |
| 865 |
| Akinmoladun VI, Gbolahan OO, Akadiri OA, Akinyamoju CA. Evaluation of the scope and practice of oral and maxillofacial surgery in Nigeria. *Nigerian Journal of Clinical Practice* 2015; **18**: 282–6. |
| 866 |
| Randrianarivony J, Ravelomanantsoa JJ, Razanamihaja N. Evaluation of the reliability and validity of the Early Childhood Oral Health Impact Scale (ECOHIS) questionnaire translated into Malagasy. *Health and Quality of Life Outcomes* 2020; **18**. DOI:[10.1186/s12955-020-01296-1](https://doi.org/10.1186/s12955-020-01296-1). |
| 867 |
| Akinmoladun VI, Akintububo OB, Adisa AO, Ojo EO, Ayuba D. Evaluation of the histopathology of orofacial lesions in a North-East Nigerian tertiary centre. *Annals of African Medicine* 2013; **12**: 105–9. |
| 868 |
| Akinmoladun VI, Okoje VN, Akinosun OM, Adisa AO, Uchendu OC. Evaluation of the haemodynamic and metabolic effects of local anaesthetic agent in routine dental extractions. *J Maxillofac Oral Surg* 2013; **12**: 424–8. |
| 869 |
| Osagie O, Saheeb BD, Egbor EP. Evaluation of the Efficacy of Platelet-Rich Plasma versus Platelet-Rich Fibrin in Alleviating Postoperative Inflammatory Morbidities after Lower Third Molar Surgery: A Double-Blind Randomized Study. *West Afr J Med* 2022; **39**: 343–9. |
| 870 |
| Etetafia MO, Obaroefe M. Evaluation of the clinical effects of impacted lower third molar tooth on adjacent oral tissues. *Brazilian Dental Science* 2022; **25**. DOI:[10.4322/bds.2022.e3024](https://doi.org/10.4322/bds.2022.e3024). |
| 871 |
| Adesola UK, Okhiabigie AP, Adeola A, Omowunmi AP, Ayodeji TO. Evaluation of the Attached Gingival Width and Sulcus Depth in an Adult Nigerian Population - A Pilot Study. *J Int Acad Periodontol* 2018; **20**: 78–85. |
| 872 |
| Diouf M, Faye A, Cisse D, Faye D, Lo CMM. Evaluation of preventive care in the dentistry department clinics of the University Cheikh Anta Diop in Dakar. *Eastern Mediterranean Health Journal* 2011; **17**: 69–73. |
| 873 |
| Omorogbe OS, Orhue O kelvin, Osayande E. Evaluation of patients’ compliance to recall visits after tooth extraction at the Dental Centre, University of Benin Teaching Hospital (UBTH): A two-year retrospective study. *Niger J Dent Res (Online)* 2022; **7**: 45–52. |
| 874 |
| Kanoute A, Carrouel F, Gare J, *et al.* Evaluation of Oral Hygiene-Related Mobile Apps for Children in Sub-Saharan Africa. *International Journal of Environmental Research and Public Health* 2022; **19**. DOI:[10.3390/ijerph191912565](https://doi.org/10.3390/ijerph191912565). |
| 875 |
| Josephin S, Nzala S, Baboo KS. Evaluation of oral hygiene services in selected public health facilities in Lusaka district of Zambia. *J Public Health Afr* 2018; **9**: 820. |
| 876 |
| Beugre-Kouassi AML, Ogou ND, Diouf JS, Beugre JB, Ngom PI. [Evaluation of motivational factors of adolescent patients for orthodontic treatment using the Q methodology]. *Orthod Fr* 2021; **92**: 453–60. |
| 877 |
| Leye Benoist F, Gaye Ndiaye F, Kane AW, Benoist HM, Farge P. Evaluation of mineral trioxide aggregate (MTA) versus calcium hydroxide cement (Dycal(®) ) in the formation of a dentine bridge: a randomised controlled trial. *Int Dent J* 2012; **62**: 33–9. |
| 878 |
| Singh R, Jayaprakash PK, Yadav A, Dawar M, Grewal H, Mishra A. Evaluation of levels of Interleukin-1b, intensity of pain and tooth movement during canine retraction using different magnitudes of continuous orthodontic force. *J Family Med Prim Care* 2019; **8**: 2373–7. |
| 879 |
| Agbaje HO, Kolawole KA, Otuyemi OD. Evaluation of early changes in oral health-related quality of life amongst Nigerian patients undergoing fixed orthodontic appliance therapy. *International Orthodontics* 2018; **16**: 571–85. |
| 880 |
| Diouf JS, Badiane A, Ngom PI, *et al.* Evaluation of dentoskeletal parameters in Senegalese subjects using orthodontic architectural analysis. *International Orthodontics* 2011; **9**: 235–47. |
| 881 |
| Umeanuka OT, Saheeb BD, Uguru CC, Chukwuneke FN. Evaluation of cortisol concentrations in saliva as a measure of stress in patients having routine dental extractions. *Br J Oral Maxillofac Surg* 2015; **53**: 557–60. |
| 882 |
| Anta S, Diouma N, Ousmane NS, Fatou LB, Florence F, Babacar T. Evaluation of Complete Pulpotomy With Biodentine on Mature Permanent Molars With Signs and Symptoms of Symptomatic Irreversible Pulpitis: 12-months Follow-up. *J Endod* 2022; **48**: 312–9. |
| 883 |
| Kolisa Y, Ayo-Yusuf O. Evaluation of caregivers’ knowledge, beliefs and practices regarding oral lesions in HIV-patients: A pilot study. *Health SA Gesondheid* 2013; **18**. DOI:[10.4102/hsag.v18i1.704](https://doi.org/10.4102/hsag.v18i1.704). |
| 884 |
| Sefah IA, Sneddon J, Essah DO, *et al.* Evaluation of antibiotic prescribing for ambulatory patients seeking primary dental care services in a public hospital in Ghana: A clinical audit study. *JAC-Antimicrobial Resistance* 2022; **4**. DOI:[10.1093/jacamr/dlac079](https://doi.org/10.1093/jacamr/dlac079). |
| 885 |
| Ajayi YO, Akinboboye BO. Evaluation of aesthetic outcome of single tooth implant borne restoration. *Niger J Dent Res* 2018; **3**. <https://search.bvsalud.org/aimafro/resource/en/biblio-1266964>. |
| 886 |
| Essama Eno Belinga L, Ngan WBKM Joseph Stephane Choukem, Simeon Pierre. Evaluation de la sante bucco-dentaire des patients diabetiques camerounais. *Health sci dis* 2013; **14**: 1–5. |
| 887 |
| Kane AST, Guirassy ML, Touré K, *et al.* Évaluation de l’hygiène buccodentaire des patients consultant le service d’odontostomatologie du centre de santé de référence de Ouelessebougou au Mali. *African Journal of Dentistry and Implantology* 2020; **17**: 22–32. |
| 888 |
| Tesfalul M, Littman-Quinn R, Antwi C, *et al.* Evaluating the potential impact of a mobile telemedicine system on coordination of specialty care for patients with complicated oral lesions in Botswana. *Journal of the American Medical Informatics Association* 2016; **23**: e142–5. |
| 889 |
| Tesfalul M, Littman-Quinn R, Antwi C, *et al.* Evaluating the impact of a mobile oral telemedicine system on medical management and clinical outcomes of patients with complicated oral lesions in Botswana. 2013 <https://www.scopus.com/inward/record.uri?eid=2-s2.0-84894301173&doi=10.3233%2f978-1-61499-289-9-1074&partnerID=40&md5=cdf9f7164d08e714e3d0b9c5d35f4b60>. |
| 890 |
| Dosumu EB, Bamishe CT, Dosumu OO, Ogunsuji OO. Evaluating dentine hypersensitivity severity in Nigerians using cumulative hypersensitivity index. *International Journal of Dentistry and Oral Science* 2019; **6**: 671–5. |
| 891 |
| Sohal KS, Moshy JR. Etiology, pattern and outcome of management of facial lacerations in dar es salaam, tanzania. *Tanzania Journal of Health Research* 2019; **21**: 1–10. |
| 892 |
| Chalya PL, Mchembe M, Mabula JB, Kanumba ES, Gilyoma JM. Etiological spectrum, injury characteristics and treatment outcome of maxillofacial injuries in a Tanzanian teaching hospital. *Journal of Trauma Management and Outcomes* 2011; **5**. DOI:[10.1186/1752-2897-5-7](https://doi.org/10.1186/1752-2897-5-7). |
| 893 |
| Ashu Agbor M, Naidoo S. Ethnomedicinal Plants Used by Traditional Healers to Treat Oral Health Problems in Cameroon. *Evidence-based Complementary and Alternative Medicine* 2015; **2015**. DOI:[10.1155/2015/649832](https://doi.org/10.1155/2015/649832). |
| 894 |
| Kolude B, Akinyele A, Joshua OT, Ahmed L. Ethnic and gender comparison of rugae patterns among clinical dental trainees in Ibadan, Nigeria. *Pan African Medical Journal* 2016; **23**. DOI:[10.11604/pamj.2016.23.204.8584](https://doi.org/10.11604/pamj.2016.23.204.8584). |
| 895 |
| Uys A, Bernitz H, Pretorius S, Steyn M. Estimating age and the probability of being at least 18 years of age using third molars: a comparison between Black and White individuals living in South Africa. *International Journal of Legal Medicine* 2018; **132**: 1437–46. |
| 896 |
| Bello SA, Adeoye JA, Oketade I, Akadiri OA. Estimated incidence and Prevalence of noma in north central Nigeria, 2010–2018: A retrospective study. *PLoS Neglected Tropical Diseases* 2019; **13**. DOI:[10.1371/journal.pntd.0007574](https://doi.org/10.1371/journal.pntd.0007574). |
| 897 |
| Maïga S, Siga Diom E, Palou EJR, *et al.* Epidemiology, clinical features and treatment of lip cancers in Senegal. *Health sci dis* 2017; **18**: 74–8. |
| 898 |
| Tolessa M, Singel AT, Merga H. Epidemiology of orthodontic treatment need in southwestern Ethiopian children: A cross sectional study using the index of orthodontic treatment need. *BMC Oral Health* 2020; **20**. DOI:[10.1186/s12903-020-01196-2](https://doi.org/10.1186/s12903-020-01196-2). |
| 899 |
| Mumena CH, Ingabire EH, Hakizimana JB, *et al.* Epidemiology of orofacial clefts in rwanda. *Rwanda Medical Journal* 2020; **77**: 8–13. |
| 900 |
| Abram MH, van Heerden WF, Rheeder P, Girdler-Brown BV, van Zyl AW. Epidemiology of oral squamous cell carcinoma. *SADJ : journal of the South African Dental Association = tydskrif van die Suid-Afrikaanse Tandheelkundige Vereniging* 2012; **67**: 550–3. |
| 901 |
| Hlongwa P, Levin J, Rispel LC. Epidemiology and clinical profile of individuals with cleft lip and palate utilising specialised academic treatment centres in South Africa. *PLoS One* 2019; **14**: e0215931. |
| 902 |
| Thema LK, Singh S. Epidemiological profile of patients utilising public oral health services in Limpopo province, South Africa. *African Journal of Primary Health Care and Family Medicine* 2017; **9**. DOI:[10.4102/phcfm.v9i1.1206](https://doi.org/10.4102/phcfm.v9i1.1206). |
| 903 |
| Folayan MO, Oginni AB, El Tantawi M, Finlayson TL, Adeniyi A. Epidemiological profile of early childhood caries in a sub-urban population in Nigeria. *BMC Oral Health* 2021; **21**. DOI:[10.1186/s12903-021-01780-0](https://doi.org/10.1186/s12903-021-01780-0). |
| 904 |
| Sanogo S, Kouma A, Cissé I, *et al.* Epidemiological and tomodensitometric aspects of post-traumatic maxillofacial fractures in Mopti, Mali. *Pan African Medical Journal* 2022; **41**. DOI:[10.11604/pamj.2022.41.309.28752](https://doi.org/10.11604/pamj.2022.41.309.28752). |
| 905 |
| Bobe Alifi Leta P, Sekele Isouradi Bourley J-P, Nyimi Bushabu F, Vinckier F, Lunguya Metila O, Situakibanza Nani-Tuma H. Epidemiologic analysis of dental cellulitis in Kinshasa city (the Democratic Republic of the Congo). *Journal of Oral Medicine and Oral Surgery* 2019; **25**. DOI:[10.1051/mbcb/2018038](https://doi.org/10.1051/mbcb/2018038). |
| 906 |
| Agholor CN, Sede MA. Endodontic treatment: an analysis of demand by adult patients in a tertiary hospital in Southern Nigeria. *Niger J Dent Res* 2018; **3**. <https://search.bvsalud.org/aimafro/resource/en/biblio-1266973>. |
| 907 |
| Lalloo R, Solanki G, Ramphoma K, Myburgh NG. Endodontic treatment-related antibiotic prescribing patterns of South African oral health professionals. *International Endodontic Journal* 2017; **50**: 1027–33. |
| 908 |
| Enabulele J, Ibhawoh L. Endodontic Treatment of the Pregnant Patient: Knowledge; Attitude and Practices of Dental Residents. *Niger med j (Online)* 2015; **56**: 311–6. |
| 909 |
| Gbadebo SO, Ajayi DM. Endodontic practice amongst nigerian dentists undergoing postgraduate training. *Pan African Medical Journal* 2021; **39**. DOI:[10.11604/pamj.2021.39.218.23205](https://doi.org/10.11604/pamj.2021.39.218.23205). |
| 910 |
| Ameh PO, Uti OG, Daramola OO. Empathy among dental students in a Nigerian institution. *European Journal of Dental Education* 2019; **23**: 135–42. |
| 911 |
| Erinoso OA, Okoturo E, Gbotolorun OM, *et al.* Emerging Trends in the Epidemiological Pattern of Head and Neck Cancers in Lagos, Nigeria. *Ann Med Health Sci Res* 2016; **6**: 301–7. |
| 912 |
| Jooma Z, Perrie H, Scribante J, Kleyenstuber T. Emergence delirium in children undergoing dental surgery under general anesthesia. *Paediatric Anaesthesia* 2020; **30**: 1020–6. |
| 913 |
| Chepkwony F, Kemoli AM, Owino R, Muasya M. Effects of socio-economic and behavioural factors on early childhood caries among children attending a dental clinic in nairobi, kenya. *East African Medical Journal* 2016; **93**: 378–85. |
| 914 |
| Olabintan AA, Otuyemi OD, Kolawole KA. Effects of Recent/Healed Post-Extraction Protocols on Incisor and Canine Alignment During Fixed Orthodontic Appliance Therapy. *Turk J Orthod* 2022; **35**: 84–93. |
| 915 |
| Kemoli AM, Amerongen WE. Effects of oral hygiene, residual caries and cervical Marginal-gaps on the survival of proximal atraumatic restorative treatment approach restorations. *Contemporary clinical dentistry* 2011; **2**: 318–23. |
| 916 |
| Muhoozi GKM, Atukunda P, Skaare AB, *et al.* Effects of nutrition and hygiene education on oral health and growth among toddlers in rural Uganda: follow-up of a cluster-randomised controlled trial. *Tropical Medicine and International Health* 2018; **23**: 391–404. |
| 917 |
| Tsobgny-Tsague N-F, Lontchi-Yimagou E, Nana ARN, *et al.* Effects of nonsurgical periodontal treatment on glycated haemoglobin on type 2 diabetes patients (PARODIA 1 study): A randomized controlled trial in a sub-Saharan Africa population. *BMC Oral Health* 2018; **18**. DOI:[10.1186/s12903-018-0479-5](https://doi.org/10.1186/s12903-018-0479-5). |
| 918 |
| Nana Nana AR, Tsobgny Tsague N-F, Lontchi-Yimagou E, *et al.* Effects of non-surgical treatment of chronic periodontitis on insulin resistance and glucose tolerance in subjects without diabetes (PARODIA 2 study). *Journal of Investigative Medicine* 2021; **69**: 1377–81. |
| 919 |
| Famurewa BA, Oginni FO, Aregbesola SB, Erhabor GE. Effects of maxillomandibular fixation and rigid internal fixation on pulmonary function in patients with mandibular fractures. *Int J Oral Maxillofac Surg* 2020; **49**: 1193–8. |
| 920 |
| Olubunmi B, Olushola I. Effects of information dissemination using video of indigenous language on 11-12 years children’s dental health. *Ethiopian journal of health sciences* 2013; **23**: 201–8. |
| 921 |
| Bankole OO, Lawal FB. Effectiveness of an oral health education program to improve mothers’ awareness of natal teeth: A randomized controlled study. *Pesquisa Brasileira em Odontopediatria e Clinica Integrada* 2020; **20**: 1–9. |
| 922 |
| Bankole OO, Dedeke AA, Denloye O, *et al.* Effectiveness of an Oral Health Care Training Workshop for School Teachers: A Pilot Study. *Annals of Ibadan Postgraduate Medicine* 2013; **11**: 18–21. |
| 923 |
| Ibikunle AA, Adeyemo WL, Ladeinde AL. Effect of submucosal or oral administration of prednisolone on postoperative sequelae following surgical extraction of impacted mandibular third molar: A randomized controlled study. *Niger Med J* 2016; **57**: 272–9. |
| 924 |
| Onyejaka NK, Folayan MO, Folaranmi N. Effect of referral for dental service on dental-service utilization by primary school children aged 8 to 11 years in Enugu, Nigeria. *Brazilian Journal of Oral Sciences* 2016; **15**: 151–7. |
| 925 |
| Kanoute A, Gare J, Meda N, *et al.* Effect of Oral Prophylactic Measures on the Occurrence of Pre-Eclampsia (OP-PE) in High-Risk Pregnant Women: A Cluster Randomized Controlled Trial. *Methods Protoc* 2021; **4**. DOI:[10.3390/mps4030061](https://doi.org/10.3390/mps4030061). |
| 926 |
| Ehizele A, Akhionbare O. Effect of non-surgical periodontal therapy on the concentration of volatile sulfur compound in mouth air of a group of nigerian young adults. *Ann Med Health Sci Res* 2013; **3**: 433–7. |
| 927 |
| Olatosi OO, Sote EO, Orenuga OO. Effect of mineral trioxide aggregate and formocresol pulpotomy on vital primary teeth: a clinical and radiographic study. *Niger J Clin Pract* 2015; **18**: 292–6. |
| 928 |
| Adeniyi AA, Oyapero A, Ajieroh V, Sofola O, Asiyanbi O. Effect of health education primaryinterventionon oral healthhealthconductedknowledgecare workersbyand lagospracticesstateof nursing mothers in. *Journal of Public Health in Africa* 2018; **9**. DOI:[10.4081/jphia.2018.833](https://doi.org/10.4081/jphia.2018.833). |
| 929 |
| Gathece LW, Wang’Ombe JK, Ng’Ang’A PM, Wanzala PN. Effect of health education on oral hygiene and gingival status of persons living with HIV attending comprehensive care centres in Nairobi, Kenya. *African Journal of AIDS Research* 2011; **10**: 495–500. |
| 930 |
| Koyio LN, van der Sanden WJ, van der Ven A, Creugers N, Merkx MA, Frencken JE. Effect of Education of Primary Health Care Workers on HIV-related Oral Lesions in Nairobi East District. *Journal of public health research* 2012; **1**: 137–40. |
| 931 |
| Adeyemo YI, Bankole OO, Aladelusi TO, Denloye OO. Effect of Dental Treatment on the Quality of Life of Children with Traumatic Dental Injuries in Ibadan, Nigeria. *J West Afr Coll Surg* 2019; **9**: 1–7. |
| 932 |
| Stanford-Moore GB, Niyigaba G, Tuyishimire G, *et al.* Effect of Delay of Care for Patients with Craniomaxillofacial Trauma in Rwanda. *OTO Open* 2022; **6**. DOI:[10.1177/2473974X221096032](https://doi.org/10.1177/2473974X221096032). |
| 933 |
| Birungi N, Fadnes LT, Okullo I, *et al.* Effect of breastfeeding promotion on early childhood caries and breastfeeding duration among 5 year old children in Eastern Uganda: A cluster randomized trial. *PLoS ONE* 2015; **10**. DOI:[10.1371/journal.pone.0125352](https://doi.org/10.1371/journal.pone.0125352). |
| 934 |
| Osunde OD, Saheeb BD. Effect of age, sex and level of surgical difficulty on inflammatory complications after third molar surgery. *J Maxillofac Oral Surg* 2015; **14**: 7–12. |
| 935 |
| Bello SA, Adeyemo WL, Bamgbose BO, Obi EV, Adeyinka AA. Effect of age, impaction types and operative time on inflammatory tissue reactions following lower third molar surgery. *Head and Face Medicine* 2011; **7**. DOI:[10.1186/1746-160X-7-8](https://doi.org/10.1186/1746-160X-7-8). |
| 936 |
| Esan A, Folayan MO, Egbetade GO, Oyedele TA. Effect of a school-based oral health education programme on use of recommended oral self-care for reducing the risk of caries by children in Nigeria. *International Journal of Paediatric Dentistry* 2015; **25**: 282–90. |
| 937 |
| Hewlett SA, Yawson AE, Calys-Tagoe BNL, *et al.* Edentulism and quality of life among older Ghanaian adults. *BMC Oral Health* 2015; **15**. DOI:[10.1186/s12903-015-0034-6](https://doi.org/10.1186/s12903-015-0034-6). |
| 938 |
| Machiri S, Kambondo G, Katena NA, January J, Maradzika J. Ecological risk factors for unmet needs in oral health care in Chegutu rural district in Zimbabwe a cross-sectional study. *International Journal of Health Promotion and Education* 2021; **59**: 83–92. |
| 939 |
| Omoregie OF, Okoh M. Early response to medical treatment of trigeminal neuralgia in a Nigerian population. *Niger Med J* 2015; **56**: 381–4. |
| 940 |
| Hudson AP, Harris AM, Mohamed N. Early identification and management of mandibular canine ectopia. *SADJ* 2011; **66**: 462–4, 466. |
| 941 |
| Alade M, Folayan MO, El Tantawi M, Oginni AB, Adeniyi AA, Finlayson TL. Early childhood caries: Are maternal psychosocial factors, decision-making ability, and caries status risk indicators for children in a sub-urban Nigerian population? *BMC Oral Health* 2021; **21**. DOI:[10.1186/s12903-020-01324-y](https://doi.org/10.1186/s12903-020-01324-y). |
| 942 |
| Mohamed N, Barnes JM. Early childhood caries and dental treatment need in low socio-economic communities in cape town, South Africa. *Health SA Gesondheid* 2018; **23**. DOI:[10.4102/hsag.v23i0.1039](https://doi.org/10.4102/hsag.v23i0.1039). |
| 943 |
| Njoroge NW, Kemoli A, Gathece LW. Early childhood caries amongst pre-school children and their caregivers’ perceptions of oral health in a Kenyan rural setting. *East African Medical Journal* 2015; **92**: 389–93. |
| 944 |
| Akinbami BO, Godspower T. Dry socket: incidence, clinical features, and predisposing factors. *Int J Dent* 2014; **2014**: 796102. |
| 945 |
| Amoo AT, James O, Adeyemi M, Taiwo AO, Adeyemo WL. Does the Initial Width of Cleft Lip Play a Role in the Occurrence of Immediate Local Complications Following Primary Cleft Lip Repairs? *J Craniofac Surg* 2021; **32**: 670–4. |
| 946 |
| Folayan MO, Arije OO. Does social vulnerability for caries predict caries status of children in sub-urban Nigeria? *Brazilian Journal of Oral Sciences* 2016; **15**: 79–85. |
| 947 |
| Azodo CC, Onyeagba MI, Odai CD. Does concern about halitosis influence individual’s oral hygiene practices? *Niger Med J* 2011; **52**: 254–9. |
| 948 |
| Azodo CC, Ojehanon PI. Does any Relationship Exist between Self Reported Gingival Bleeding; Oral Health Perception; Practices and Concerns? *Niger med j (Online)* 2012; **53**: 161–5. |
| 949 |
| Osagie O, Saheeb BD, Egbor EP. Do the bioactive effects of platelet-rich plasma and platelet-rich fibrin influence the oral health related quality of life following impacted third molar surgery? A randomized comparative study. *Niger J Clin Pract* 2021; **24**: 712–7. |
| 950 |
| Åstrøm AN, Lie SA, Mbawalla H. Do self-efficacy and depression predict oral impacts on daily performances across time? A 2-yr follow-up of students in Tanzania. *European Journal of Oral Sciences* 2016; **124**: 358–67. |
| 951 |
| Adesina A, Olufemi E, Oluwatosin O, *et al.* Do Health-Seeking Populations Know the Link Between Human Papillomavirus and Oropharyngeal Cancer? A Cross-Sectional Study in a Nigerian Population. *Community Health Equity Res Policy* 2023; **43**: 153–60. |
| 952 |
| Oyetola EO, Adesina OM, Ogunbameru K, Egunjobi S, Adejobi AF. Distribution of Medical Conditions among Dental Patients. *Niger Med J* 2020; **61**: 129–35. |
| 953 |
| Shaw L, Harjunmaa U, Doyle R, *et al.* Distinguishing the signals of gingivitis and periodontitis in supragingival plaque: A cross-sectional cohort study in Malawi. *Applied and Environmental Microbiology* 2016; **82**: 6057–67. |
| 954 |
| Temilola OD, Folayan MO. Distinguishing predisposing factors for enamel hypoplasia and molar-incisor hypomineralization in children in Ile-Ife, Nigeria. *Brazilian Journal of Oral Sciences* 2015; **14**: 318–22. |
| 955 |
| Motlokwa PK, Tsima BM, Martei YM, *et al.* Disparities in Oral Cancer Stage at Presentation in a High HIV Prevalence Setting In Sub-Saharan Africa. *JCO global oncology* 2022; **8**: e2100439. |
| 956 |
| Olatosi OO, Oyapero A, Onyejaka NK. Disparities in caries experience and socio-behavioural risk indicators among private school children in Lagos, Nigeria. *Pesquisa Brasileira em Odontopediatria e Clinica Integrada* 2020; **20**: 1–13. |
| 957 |
| Kahabuka FK, Mugonzibwa EA, Mwalutambi S, Kikwilu EN. Diseases and conditions falsely linked with “nylon teeth” myth: a cross sectional study of Tanzanian adults. *Tanzania Journal of Health Research* 2015; **17**. DOI:[10.4314/thrb.v17i2.7](https://doi.org/10.4314/thrb.v17i2.7). |
| 958 |
| Mbawalla HS, Mtaya M, Masalu JR, Brudvik P, Astrom AN. Discriminative ability of the generic and condition-specific Child-Oral Impacts on Daily Performances (Child-OIDP) by the Limpopo-Arusha School Health (LASH) Project: A cross-sectional study. *BMC Pediatrics* 2011; **11**. DOI:[10.1186/1471-2431-11-45](https://doi.org/10.1186/1471-2431-11-45). |
| 959 |
| Adou-Assoumou MN, Djolé SX, Krah-Sinan AA, Adou JA, Siendou D, Mansilla EC. Direct technique premolar coronal restorations: From metallic material to ‘complete adhesive restoration’. *J Conserv Dent* 2019; **22**: 568–72. |
| 960 |
| Agbaje HO, Kolawole KA, Folayan MO, *et al.* Digit sucking, age, sex, and socioeconomic status as determinants of oral hygiene status and gingival health of children in suburban Nigeria. *Journal of Periodontology* 2016; **87**: 1047–56. |
| 961 |
| Kolawole KA, Folayan MO, Agbaje HO, *et al.* Digit sucking habit and association with dental caries and oral hygiene status of children aged 6 months to 12 years resident in semi-urban Nigeria. *PLoS ONE* 2016; **11**. DOI:[10.1371/journal.pone.0148322](https://doi.org/10.1371/journal.pone.0148322). |
| 962 |
| Alade O, Folayan MO, Adeniyi A, *et al.* Differences in Oral Lesions Associated with Tobacco Smoking, E-Cigarette Use and COVID-19 Infection among Adolescents and Young People in Nigeria. *International Journal of Environmental Research and Public Health* 2022; **19**. DOI:[10.3390/ijerph191710509](https://doi.org/10.3390/ijerph191710509). |
| 963 |
| Kebede A, Retta N, Abuye C, *et al.* Dietary fluoride intake and associated skeletal and dental fluorosis in school age children in rural Ethiopian Rift Valley. *International Journal of Environmental Research and Public Health* 2016; **13**. DOI:[10.3390/ijerph13080756](https://doi.org/10.3390/ijerph13080756). |
| 964 |
| Kabore W, Garé J, Koama C. Diagnostic and therapeutic approaches to endodontic-periodontal lesions: A survey among dental surgeons in Ouagadougou, Burkina Faso. *Saudi Endodontic Journal* 2022; **12**: 195–203. |
| 965 |
| Leppilahti JM, Harjunmaa U, Järnstedt J, *et al.* Diagnosis of newly delivered mothers for periodontitis with a novel oral-rinse aMMP-8 point-of-care test in a rural Malawian population. *Diagnostics* 2018; **8**. DOI:[10.3390/diagnostics8030067](https://doi.org/10.3390/diagnostics8030067). |
| 966 |
| Folayan MO, Chukwumah NM, Popoola BO, *et al.* Developmental defects of the enamel and its impact on the oral health quality of life of children resident in Southwest Nigeria. *BMC Oral Health* 2018; **18**. DOI:[10.1186/s12903-018-0622-3](https://doi.org/10.1186/s12903-018-0622-3). |
| 967 |
| Masumo R, Bårdsen A, Åstrøm AN. Developmental defects of enamel in primary teeth and association with early life course events: A study of 6-36 month old children in Manyara, Tanzania. *BMC Oral Health* 2013; **13**. DOI:[10.1186/1472-6831-13-21](https://doi.org/10.1186/1472-6831-13-21). |
| 968 |
| Etiaba E, Uguru N, Ebenso B, *et al.* Development of oral health policy in Nigeria: An analysis of the role of context, actors and policy process. *BMC Oral Health* 2015; **15**. DOI:[10.1186/s12903-015-0040-8](https://doi.org/10.1186/s12903-015-0040-8). |
| 969 |
| Postma TC, White JG. Developing students’ clinical reasoning skills: correlates of perceived relevance of two teaching and learning approaches. *Eur J Dent Educ* 2017; **21**: 52–7. |
| 970 |
| Ibiyemi O, Lawal F, Osuh M, *et al.* Developing an Oral Hygiene Education Song for Children and Teenagers in Nigeria. *International Dental Journal* 2022; **72**: 866–71. |
| 971 |
| Adeola AO, Oladimeji AA. Developing a visual rating chart for the esthetic outcome of unilateral cleft lip and palate repair. *Ann Maxillofac Surg* 2015; **5**: 55–61. |
| 972 |
| Folayan MO, Khami MR, Folaranmi N, *et al.* Determinants of preventive oral health behaviour among senior dental students in Nigeria. *BMC Oral Health* 2013; **13**. DOI:[10.1186/1472-6831-13-28](https://doi.org/10.1186/1472-6831-13-28). |
| 973 |
| Folayan MO, Khami MR, Folaranmi N, *et al.* Determinants of preventive dental practice for children among final-year dental students in Nigeria. *International Journal of Paediatric Dentistry* 2014; **24**: 43–50. |
| 974 |
| Wandera M, Åstrøm AN, Okullo I, Tumwine JK. Determinants of periodontal health in pregnant women and association with infants’ anthropometric status: a prospective cohort study from Eastern Uganda. *BMC Pregnancy and Childbirth* 2012; **12**. DOI:[10.1186/1471-2393-12-90](https://doi.org/10.1186/1471-2393-12-90). |
| 975 |
| Chidzonga MM, Carneiro LC, Kalyanyama BM, Kwamin F, Oginni FO. Determinants of Oral Diseases in the African and Middle East Region. *Advances in dental research* 2015; **27**: 26–31. |
| 976 |
| Shitu K, Alemayehu M, Buunk-Werkhoven YAB, Handebo S. Determinants of intention to improve oral hygiene behavior among students based on the theory of planned behavior: A structural equation modelling analysis. *PLoS ONE* 2021; **16**. DOI:[10.1371/journal.pone.0247069](https://doi.org/10.1371/journal.pone.0247069). |
| 977 |
| Derseh BT, Mekonnen K, Kibret T, Beyene A, Mihretie A. Determinants of Dental Health Problems Among Adult Patients at Dental Clinic, Debre Berhan Comprehensive Specialized Hospital, Ethiopia: Unmatched Case–Control Study. *Clinical, Cosmetic and Investigational Dentistry* 2021; **13**: 495–505. |
| 978 |
| Guracho TT, Atomssa EM, Megersa OA, Tolossa T. Determinants of dental caries among adolescent patients attending Hospitals in West Wollega Zone, Western Ethiopia: A case-control study. *PLoS ONE* 2021; **16**. DOI:[10.1371/journal.pone.0260427](https://doi.org/10.1371/journal.pone.0260427). |
| 979 |
| Bolenge IJ, Lutula PN, Bieleli EI, *et al.* Déterminants de la maladie parodontale en milieux hospitaliers à Kinshasa. *Odonto-stomatol trop* 2018; **41**: 25–35. |
| 980 |
| Ameh PO, Nwaokorie FO, Ayanbadejo PO. Detection of six periodontopathogens in the subgingival plaque of patients with chronic periodontitis in Lagos, Nigeria. *Niger J Dent Res* 2020; **5**: 145–54. |
| 981 |
| Blankson P-K, Blankson HNA, Obeng-Nkrumah N, *et al.* Detection of herpes viruses in Ghanaian patients with periodontitis. *Journal of investigative and clinical dentistry* 2019; **10**: e12386. |
| 982 |
| Ajayi YO, Sofola OO. Descriptors of permanent teeth with cariously exposed pulp in patients presenting at a Nigerian hospital. *Acta Odontologica Scandinavica* 2013; **71**: 1348–50. |
| 983 |
| Omitola OG, Soyele OO, Butali A, *et al.* Descriptive epidemiology of salivary gland neoplasms in Nigeria: An AOPRC multicenter tertiary hospital study. *Oral Diseases* 2019; **25**: 142–9. |
| 984 |
| Eshete M, Butali A, Deressa W, *et al.* Descriptive Epidemiology of Orofacial Clefts in Ethiopia. *Journal of Craniofacial Surgery* 2017; **28**: 334–7. |
| 985 |
| Okoh DS, Akinshipo AO, Butali A, *et al.* Descriptive Epidemiology of Odontogenic Tumors in Nigeria: An African Oral Pathology Research Consortium Multicenter Study. *Nigerian Journal of Clinical Practice* 2020; **23**: 1695–701. |
| 986 |
| Adeyemo AA, Ogunkeyede SA. Denture impaction in the oesophagus: correlation of site and duration of impaction with sequelae. *Ann Ib Postgrad Med* 2019; **17**: 65–70. |
| 987 |
| Ogunrinde TJ, Opeodu OI. Denture care practice among patients attending the prosthetic clinic in a Nigerian teaching hospital. *Niger Med J* 2015; **56**: 199–203. |
| 988 |
| Agbor AM, Chinedu AC, Ebot-Tabil B, Naidoo S. Dentofacial injuries in commercial motorcycle accidents in Cameroon: Pattern and cost implication of care. *African Health Sciences* 2014; **14**. DOI:[10.4314/ahs.v14i1.12](https://doi.org/10.4314/ahs.v14i1.12). |
| 989 |
| Chetty M, Roberts T, Shaik S, Beighton P. Dentinogenesis imperfecta in Osteogenesis imperfecta type XI in South Africa: a genotype–phenotype correlation. *BDJ Open* 2019; **5**. DOI:[10.1038/s41405-019-0014-z](https://doi.org/10.1038/s41405-019-0014-z). |
| 990 |
| Savage KO, Oderinu OH, Oginni AO, Uti OG, Adegbulugbe IC, Dosumu OO. Dentine hypersensitivity and associated factors: A Nigerian cross-sectional study. *Pan African Medical Journal* 2019; **33**. DOI:[10.11604/pamj.2019.33.272.18056](https://doi.org/10.11604/pamj.2019.33.272.18056). |
| 991 |
| Azodo CC, Amayo AC. Dentinal sensitivity among a selected group of young adults in Nigeria. *Niger Med J* 2011; **52**: 189–92. |
| 992 |
| Eigbobo JO, Nzomiwu CL. Dental visit behaviour among primary school children in Port Harcourt, Nigeria. *Journal of International Dental and Medical Research* 2015; **8**: 104–9. |
| 993 |
| Rubin PF, Winocur E, Erez A, Birenboim-Wilensky R, Peretz B. Dental Treatment Needs among Children and Adolescents Residing in an Ugandan Orphanage. *The Journal of clinical pediatric dentistry* 2016; **40**: 486–9. |
| 994 |
| Otoh EC, Taiwo OO, Adeleke OA, Majekodunmi OJ, Ajike SO. Dental Trauma in Adult and Elderly Nigerians: A National Survey. *West Afr J Med* 2021; **38**: 313–20. |
| 995 |
| Adeniyi AA, Ola BA, Edeh CE, Ogunbanjo BO, Adewuya AO. Dental status of patients with mental disorders in a Nigerian teaching hospital: A preliminary survey. *Special Care in Dentistry* 2011; **31**: 134–7. |
| 996 |
| Asuzu MC, Oke GA, Osuh ME. Dental Services and Attitudes towards its Regular Utilization among Civil Servants in Ibadan; Nigeria. *Annals of Ibadan Postgraduate Medicine* 2014; **12**: 7–14. |
| 997 |
| Ndagire B, Muwazi L, Nabaggala GS, Kutesa A, Rwenyonyi CM. Dental Practitioners’ Knowledge, Attitude, and Practice in Caries Risk Assessment and Management: A Cross-sectional Survey in Kampala Metropolitan, Uganda. *Journal of Contemporary Dental Practice* 2021; **22**: 1377–85. |
| 998 |
| Roberts T, Chetty M, Kimmie-Dhansay F, Fieggen K, Stephen LXG. Dental needs of intellectually disabled children attending six special educational facilities in Cape Town. *South African Medical Journal* 2016; **106**: S94–7. |
| 999 |
| Kouassi AJF, Sonan NK, Soumahoro S, Kouadio KJ, Djaha K. Dental maturity and determination of the chronological age at the Ivorians girls. *Revue de Medecine Legale* 2018; **9**: 57–60. |
| 1000 |
| Taiwo OO, Jalo HP. Dental Injuries in 12-year Old Nigerian students. *Dental Traumatology* 2011; **27**: 230–4. |
| 1001 |
| Ajayi DM, Abiodun-Solanke I, Gbadebo SO, Fasola AO, Dosumu OO, Arotiba JT. Dental implant treatment at a Nigerian teaching hospital. *J West Afr Coll Surg* 2014; **4**: 89–99. |
| 1002 |
| Gbadebo OS, Lawal FB, Sulaiman AO, Ajayi DM. Dental implant as an option for tooth replacement: The awareness of patients at a tertiary hospital in a developing country. *Contemp Clin Dent* 2014; **5**: 302–6. |
| 1003 |
| Tefera AT, Girma B, Adane A, *et al.* Dental health problems and treatment-seeking behavior among special need school students in Amhara region, Ethiopia. *BMC Oral Health* 2021; **21**. DOI:[10.1186/s12903-021-01856-x](https://doi.org/10.1186/s12903-021-01856-x). |
| 1004 |
| Ogundele BO, Ogunsile SE. Dental health knowledge, attitude and practice on the occurrence of dental caries among adolescents in a local government area (LGA) of Oyo state, Nigeria. *Asian Journal of Epidemiology* 2011; **3**: 165–72. |
| 1005 |
| Ize-Iyamu IN, Otaren J, Oserivwoja OO. Dental habits among 2 to 5 years old children in Benin city. *Niger J Dent Res* 2018; **3**. <https://search.bvsalud.org/aimafro/resource/en/biblio-1266968>. |
| 1006 |
| Menya D, Maina SK, Kibosia C, *et al.* Dental fluorosis and oral health in the African Esophageal Cancer Corridor: Findings from the Kenya ESCCAPE case–control study and a pan-African perspective. *International Journal of Cancer* 2019; **145**: 99–109. |
| 1007 |
| Habiyakare T, Schurer JM, Poole B, *et al.* Dental fluorosis among people and livestock living on Gihaya Island in Lake Kivu, Rwanda. *One Health Outlook* 2021; **3**: 23. |
| 1008 |
| Sohal KS, Mtaya-Mlangwa M, Essajee S. Dental fear and its related factors among patients managed in a paediatric dental clinic of a university hospital in Tanzania. *Medical Journal of Zambia* 2022; **49**: 59–66. |
| 1009 |
| Isiekwe GI, Sofola OO, Onigbogi OO, Utomi IL, Sanu OO, daCosta OO. Dental esthetics and oral health-related quality of life in young adults. *Am J Orthod Dentofacial Orthop* 2016; **150**: 627–36. |
| 1010 |
| Bationo R, Guiguimdé WPL, Ouédraogo H, Somé B. Dental emergencies in Burkina Faso armed forces. *Dental and Medical Problems* 2017; **54**: 49–51. |
| 1011 |
| Titinchi F, Behardien N. Dental development in a sample of South African HIV-positive children. *Special Care in Dentistry* 2019; **39**: 135–9. |
| 1012 |
| Okolo CC, Oredugba FA, Denloye OO, Adeyemo YI. Dental Caries, Traumatic Dental Injuries and Gingivitis among Street-Children in Kano, Nigeria. *West Afr J Med* 2022; **39**: 1040–4. |
| 1013 |
| Nkambule NR, Madiba TK, Bhayat A. Dental caries, body mass index, and diet among learners at selected primary schools in pretoria, Gauteng Province, South Africa. *Journal of Contemporary Dental Practice* 2019; **20**: 1241–8. |
| 1014 |
| Carneiro LC, Kabulwa MN. Dental Caries, and Supragingival Plaque and Calculus among Students, Tanga, Tanzania. *ISRN Dent* 2012; **2012**: 245296. |
| 1015 |
| Olatosi OO, Alade AA, Naicker T, *et al.* Dental Caries Severity and Nutritional Status of Nigerian Preschool Children. *JDR Clinical and Translational Research* 2022; **7**: 154–62. |
| 1016 |
| Nqcobo CB, Yengopal V, Rudolph MJ, Thekiso M, Joosab Z. Dental caries prevalence in children attending special needs schools in Johannesburg, Gauteng Province, South Africa. *SADJ : journal of the South African Dental Association = tydskrif van die Suid-Afrikaanse Tandheelkundige Vereniging* 2012; **67**: 308–13. |
| 1017 |
| Denloye OO, Ajayi DM, Popoola BO. Dental caries prevalence and bilateral occurrence in premolars and molars of adolescent school children in Ibadan, Nigeria. *Odonto-stomatologie tropicale = Tropical dental journal* 2015; **38**: 46–50. |
| 1018 |
| Ndagire B, Mwesigwa CL, Ntuulo JM, Mayanja-Kizza H, Nakanjako D, Rwenyonyi CM. Dental Caries Pattern and Treatment Needs among Ugandan Adolescent Students: A Cross-Sectional Study. *International Journal of Dentistry* 2020; **2020**. DOI:[10.1155/2020/8135865](https://doi.org/10.1155/2020/8135865). |
| 1019 |
| Mukashyaka C, Uzabakiriho B, Amoroso CL, *et al.* Dental caries management at a rural district hospital in northern Rwanda: a neglected disease. *Public Health Action* 2015; **5**: 158–61. |
| 1020 |
| Agbaje MO, Adeniyi AA, Salisu MA, Animashaun AB, Ogunbanjo BO. Dental caries in children--an assessment of the knowledge of Nigerian paediatricians. *Afr J Med Med Sci* 2013; **42**: 157–63. |
| 1021 |
| Agbaje MO. Dental caries in children--an assessment of the knowledge of Nigerian paediatricians. *African journal of medicine and medical sciences* 2013; **42**: 157–63. |
| 1022 |
| Chikte U, Pontes CC, Karangwa I, *et al.* Dental caries in a South African adult population: findings from the Cape Town Vascular and Metabolic Health Study. *International Dental Journal* 2020; **70**: 176–82. |
| 1023 |
| Lawal F, Alade O. Dental caries experience and treatment needs of an adult female population in Nigeria. *African Health Sciences* 2017; **17**: 905–11. |
| 1024 |
| Bogale B, Engida F, Hanlon C, Prince MJ, Gallagher JE. Dental caries experience and associated factors in adults: a cross-sectional community survey within Ethiopia. *BMC Public Health* 2021; **21**. DOI:[10.1186/s12889-021-10199-9](https://doi.org/10.1186/s12889-021-10199-9). |
| 1025 |
| Akinyamoju CA, Dairo DM, Adeoye IA, Akinyamoju AO. Dental caries and oral hygiene status: Survey of schoolchildren in rural communities, Southwest Nigeria. *The Nigerian postgraduate medical journal* 2018; **25**: 239–45. |
| 1026 |
| Mafuvadze BT, Mahachi L, Mafuvadze B. Dental caries and oral health practice among 12 year old school children from low socio-economic status background in Zimbabwe. *Pan African Medical Journal* 2013; **14**: 164. |
| 1027 |
| Adeniyi AA, Oyapero OA, Ekekezie OO, Braimoh MO. Dental caries and nutritional status of school children in Lagos, Nigeria - A preliminary survey. *Journal of the West African College of Surgeons* 2016; **6**: 15–38. |
| 1028 |
| Coker MO, Akhigbe P, Osagie E, *et al.* Dental caries and its association with the oral microbiomes and HIV in young children—Nigeria (DOMHaIN): a cohort study. *BMC Oral Health* 2021; **21**. DOI:[10.1186/s12903-021-01944-y](https://doi.org/10.1186/s12903-021-01944-y). |
| 1029 |
| Zewdu T, Abu D, Agajie M, Sahilu T. Dental caries and associated factors in Ethiopia: systematic review and meta-analysis. *Environmental Health and Preventive Medicine* 2021; **26**. DOI:[10.1186/s12199-021-00943-3](https://doi.org/10.1186/s12199-021-00943-3). |
| 1030 |
| Mwakayoka H, Masalu JR, Namakuka Kikwilu E. Dental Caries and Associated Factors in Children Aged 2-4 Years Old in Mbeya City, Tanzania. *J Dent (Shiraz)* 2017; **18**: 104–11. |
| 1031 |
| Mulu W, Demilie T, Yimer M, Meshesha K, Abera B. Dental caries and associated factors among primary school children in Bahir Dar city: a cross-sectional study. *BMC Res Notes* 2014; **7**: 949. |
| 1032 |
| Geleto A, Sinba E, Ali MM. Dental caries and associated factors among patients visiting Shashamane Comprehensive Specialized Hospital. *PLoS ONE* 2022; **17**. DOI:[10.1371/journal.pone.0265000](https://doi.org/10.1371/journal.pone.0265000). |
| 1033 |
| Teshome A, Andualem G, Derese K. Dental caries and associated factors among patients attending the university of gondar comprehensive hospital dental clinic, north west ethiopia: A hospital-based cross-sectional study. *Clinical, Cosmetic and Investigational Dentistry* 2020; **12**: 191–8. |
| 1034 |
| Shiferaw A, Alem G, Tsehay M, Kibret GD. Dental caries and associated factors among diabetic and nondiabetic adult patients attending Bichena Primary Hospital’s Outpatient Department. *Front Oral Health* 2022; **3**: 938405. |
| 1035 |
| Anthony SN, Mbawalla HS, Kahabuka FK, Siziya S. Dental caries according to CAST among Zambian adolescents; pattern, socio-demographic and behavioral correlates. *BMC Oral Health* 2022; **22**. DOI:[10.1186/s12903-022-02217-y](https://doi.org/10.1186/s12903-022-02217-y). |
| 1036 |
| Mbacké Lo CM, Diop M, Kanouté A, *et al.* Dental care risk management provided by social protection institutions of Senegal. *Journal of Public Health in Africa* 2016; **6**: 80–2. |
| 1037 |
| Amuh V, Okojie O, Ehizele A. Dental care knowledge and practice of a group of health workers in benin city, Nigeria. *Ann Med Health Sci Res* 2014; **4**: S307-10. |
| 1038 |
| Osiatuma VI, Otuyemi OD, Kolawole KA, Amusa YB, Ogunbanjo BO. Dental Arch Dimensions of Nigerian Children with Hypertrophied Adenoids. *Turk J Orthod* 2017; **30**: 42–9. |
| 1039 |
| Nzomiwu CL, Fomete B, Omisakin OO. Dental Anomalies Associated with Orofacial Cleft among a Group of Individuals in Northwestern Nigeria. *West Afr J Med* 2021; **38**: 3–7. |
| 1040 |
| Kutesa AM, Rwenyonyi CM, Mwesigwa CL, Muhammad M, Nabaggala GS, Kalyango J. Dental age estimation using radiographic assessment of third molar eruption among 10-20-year-old Ugandan population. *J Forensic Dent Sci* 2019; **11**: 16–21. |
| 1041 |
| Kihara EN, Gichangi P, Liversidge HM, Butt F, Gikenye G. Dental age estimation in a group of Kenyan children using Willems’ method: a radiographic study. *Annals of Human Biology* 2017; **44**: 614–21. |
| 1042 |
| Tshimbila Kabangu JMV, Hodges AM, Galiwango GW. Demographic and clinical profile of craniofacial clefts at comprehensive rehabilitation service in Uganda. *Afr j health issues* 2017; **1**: 1–7. |
| 1043 |
| Umeh O, Utomi I, Ndukwe A, Izuka M. Demineralization preventive practices among Nigerian orthodontists-An evidence-based approach? *Nigerian Journal of Clinical Practice* 2020; **23**: 589–95. |
| 1044 |
| Lawal A, Adisa A, Sigbeku O. Cysts of the oro-facial region: A Nigerian experience. *J Oral Maxillofac Pathol* 2012; **16**: 167–71. |
| 1045 |
| Olojede ACO, Effiom OA, Adisa AO, *et al.* Cysts of the oro-facial region: A clinico-pathologic review of 403 nigerian cases. *East African Medical Journal* 2017; **94**: 116–24. |
| 1046 |
| Meer S, Dulabh S. Cystic lymphoid hyperplasia: an orofacial lesion strongly associated with HIV and AIDS. *Histopathology* 2013; **62**: 1067–74. |
| 1047 |
| Oladega AA, James O, Adeyemo WL. Cyanoacrylate tissue adhesive or silk suture for closure of surgical wound following removal of an impacted mandibular third molar: A randomized controlled study. *J Craniomaxillofac Surg* 2019; **47**: 93–8. |
| 1048 |
| Beshtawi K, Qirresh E, Parker M, Shaik S. Custom Focal Trough in Cone-Beam Computed Tomography Reformatted Panoramic Versus Digital Panoramic for Mental Foramen Position to Aid Implant Planning. *J Clin Imaging Sci* 2020; **10**: 34. |
| 1049 |
| Mugonzibwa EA, Kahabuka FK, Mwalutambi SC, Kikwilu EN. Current status of nylon teeth myth in Tanzania: A cross sectional study. *BMC Oral Health* 2018; **18**. DOI:[10.1186/s12903-017-0462-6](https://doi.org/10.1186/s12903-017-0462-6). |
| 1050 |
| Yalcouyé A, Esoh K, Guida L, Wonkam A. Current profile of Charcot-Marie-Tooth disease in Africa: A systematic review. *Journal of the Peripheral Nervous System* 2022; **27**: 100–12. |
| 1051 |
| Fadeyibi IO, Adeniyi AA, Jewo PI, Saalu LC, Fasawe AA, Ademiluyi SA. Current pattern of cleft lip and palate deformities in Lagos, Nigeria. *Cleft Palate-Craniofacial Journal* 2012; **49**: 730–5. |
| 1052 |
| Omeje KU, Efunkoya AA, Amole OI, Akhiwu BI, Osunde OD, Agbara R. Culture and patients’ perception to maxillofacial surgical practices in kano, Nigeria. *Niger J Dent Res* 2018; **3**: 79–83. |
| 1053 |
| Ize-Iyamu IN, Saheeb BD. Cultural constraints in the use of dental implants and orthodontic mini-implants in Benin City, Nigeria. *Nigerian Journal of Clinical Practice* 2019; **22**: 885–90. |
| 1054 |
| Bilal S, Nzabandora JP, Uwamahoro DL, Meisner L, Purkayastha S, Aluisio AR. Cross-sectional survey of treatments and outcomes among injured adult patients in Kigali, Rwanda. *African Journal of Emergency Medicine* 2021; **11**: 299–302. |
| 1055 |
| Hendricks R, Vicatos G. Creation of Bone and Soft Tissue in Postmaxillectomy Patients Using Curvilinear Transport Distraction Osteogenesis. *Ann Maxillofac Surg* 2019; **9**: 319–25. |
| 1056 |
| Isiekwe GI, Oguchi CO, Dacosta OO, Utomi IL. Craniofacial orthodontics and postgraduate orthodontic training in Nigeria. *Nigerian Journal of Clinical Practice* 2016; **19**: 375–9. |
| 1057 |
| Adekoya MN, Adeyemi TE, Aikins EA. COVID-19 risks and extra-protective measures practised among Nigerian orthodontists and orthodontic residents. *The Nigerian postgraduate medical journal* 2021; **28**: 88–93. |
| 1058 |
| Aladelusi TO, Atiba FA, Gbadebo SO, Adeyemo YI, Olusanya AA, Akadiri OA. COVID-19 outbreak and dental health care provision in Nigeria: a national survey. *BMC Oral Health* 2021; **21**. DOI:[10.1186/s12903-021-01860-1](https://doi.org/10.1186/s12903-021-01860-1). |
| 1059 |
| Nwhator S, Opeodu O, Ayanbadejo P, *et al.* Could periodontitis affect time to conception? *Ann Med Health Sci Res* 2014; **4**: 817–22. |
| 1060 |
| Klint NK, Satu L, Risto T. Costs of dental care and its financial impacts on patients in a population with low availability of services. *Community Dental Health* 2019; **36**: 131–6. |
| 1061 |
| Molete MP, Chola L, Hofman KJ. Costs of a school-based dental mobile service in South Africa. *BMC Health Services Research* 2016; **16**. DOI:[10.1186/s12913-016-1827-2](https://doi.org/10.1186/s12913-016-1827-2). |
| 1062 |
| Effenberger S, Greenwall L, Cebula M, *et al.* Cost-effectiveness and efficacy of fluoride varnish for caries prevention in South African children: A cluster-randomized controlled community trial. *Community Dentistry and Oral Epidemiology* 2021. DOI:[10.1111/cdoe.12702](https://doi.org/10.1111/cdoe.12702). |
| 1063 |
| Seck A, Guèye M, Dieng L, *et al.* [Correlations between colorimetric parameters of teeth, eyes and skin. Perspectives in the choice of tooth shade for complete denture]. *Odontostomatol Trop* 2013; **36**: 17–25. |
| 1064 |
| Masiga MA, Machoki JM. Correlation of oral health home-care practices, snacking habits and dental caries experience among HIV-positive children in Nairobi, Kenya. *East African Medical Journal* 2012; **89**: 217–23. |
| 1065 |
| Diouf JS, Touré B, Ndiaye M, *et al.* Correlation between sagittal photogrammetric measurements of the soft tissue profile and dental arches measurements. *L’ Orthodontie française* 2015; **86**: 303–11. |
| 1066 |
| Adeyemo WL, James O, Oladega AA, *et al.* Correlation Between Height and Impacted Third Molars and Genetics Role in Third Molar Impaction. *J Maxillofac Oral Surg* 2021; **20**: 149–53. |
| 1067 |
| de Tove MMS, Bakayoko-Ly R, N’guessan KA, Kolomdou K, N’cho-Oka Affiba E. Correlation between anxiety and the dental pain in children: Investigations at the Center for consultation and stomatology treatment of Abidjan (Ivory Cost). *Medecine Buccale Chirurgie Buccale* 2012; **18**: 333–7. |
| 1068 |
| Adekunle AA, Uti OG, Sofola OO. Correlates of illness behaviour related to orofacial infections of odontogenic origin among adults in a semi urban community in Nigeria. *Ghana Medical Journal* 2019; **53**: 294–8. |
| 1069 |
| Sulaiman AO, Shaba OP, Dosumu OO, Ajayi DM. Coronal tissue loss in endodontically treated teeth. *Afr J Med Med Sci* 2012; **41**: 437–44. |
| 1070 |
| Hlongwa P, Rispel LC. Coproduction in the management of individuals with cleft lip and palate in south africa: The ekhaya lethu model. *International Journal for Quality in Health Care* 2021; **33**: RE. |
| 1071 |
| Sales N, Sohal KS, Moshy JR, Owibingire SS, Deoglas DK, Laizer PJ. Conscious sedation in dentistry: knowledge and practice among dental professionals in Tanzania. *J Dent Anesth Pain Med* 2021; **21**: 557–64. |
| 1072 |
| Sangaré AD, Samba M, Meless Guanga D, Da Danho V, Guinan J-C, Bakayoko-Ly R. Connaissances et pratiques en hygiène hospitalière des personnels du centre de consultations et de traitements odontostomatologiques du CHU de Cocody d’Abidjan (Côte d’Ivoire). *Revue Ivoirienne d’Odonto-Stomatologie* 2018; **20**: 16–21. |
| 1073 |
| Guirassy ML, Thiam D, Diallo AM, *et al.* Connaissances et attitudes face aux péricoronarites: enquête auprès des chirurgiens-dentistes de Dakar (Sénégal). *African Journal of Dentistry and Implantology* 2019; **14**: 37–44. |
| 1074 |
| Nagalo K, Ouédraogo I, Laberge J-M, Caouette-Laberge L, Turgeon J. Congenital malformations and medical conditions associated with orofacial clefts in children in Burkina Faso. *BMC Pediatrics* 2017; **17**. DOI:[10.1186/s12887-017-0833-9](https://doi.org/10.1186/s12887-017-0833-9). |
| 1075 |
| Gakonyo J, Mohamedali AJ, Mungure EK. Cone beam computed tomography assessment of the buccal bone thickness in anterior maxillary teeth: Relevance to immediate implant placement. *International Journal of Oral and Maxillofacial Implants* 2018; **33**: 880–7. |
| 1076 |
| Soyele OO, Aborisade A, Adesina OM, *et al.* Concordance between clinical and histopathologic diagnosis and an audit of oral histopathology service at a Nigerian tertiary hospital. *Pan African Medical Journal* 2019; **34**. DOI:[10.11604/pamj.2019.34.100.19388](https://doi.org/10.11604/pamj.2019.34.100.19388). |
| 1077 |
| Anyanechi CE, Osunde OD, Saheeb BD. Complications of the use of trans-osseous wire osteosynthesis in the managementof compound, unfavorable and non-comminuted mandibular angle fractures. *Ghana medical journal* 2016; **50**: 172–9. |
| 1078 |
| Anyanechi CE, Saheeb BD. Complications of mandibular fracture: Study of the treatment methods in Calabar, Nigeria. *West Indian Medical Journal* 2014; **63**: 349–53. |
| 1079 |
| Nri-Ezedi CA, Ofiaeli OC, Nwaneli EI, Ugochukwu JU, Ulasi TO, Ilika AL. Compendium of Oral Pathologies in Children Presenting to A Tertiary Hospital in Nigeria. *Orient Journal of Medicine* 2020; **32**: 23–7. |
| 1080 |
| Lawal FB, Taiwo JO, Arowojolu MO. Comparison of two oral health-related quality of life measures among adult dental patients. *Oral Health and Preventive Dentistry* 2015; **13**: 65–74. |
| 1081 |
| Ayedun OS, Oredugba FA, Sote EO. Comparison of the treatment outcomes of the conventional stainless steel crown restorations and the hall technique in the treatment of carious primary molars. *Niger J Clin Pract* 2021; **24**: 584–94. |
| 1082 |
| Waweru LW, Opinya GN, Ng’Ang’A PM. Comparison of the prevalence of dental caries in 12-15 year-old children with dental fluorosis and those without dental fluorosis from juja in rural Kenya. *East African Medical Journal* 2016; **93**: 416–20. |
| 1083 |
| Makanjuola JO, Umesi DC, Oderinu OH. Comparison of the incidence of flare-up and time efficiency in single visit root canal treatment employing either rotary or manual step-back canal preparatory technique. *The Nigerian postgraduate medical journal* 2018; **25**: 100–4. |
| 1084 |
| Adetayo AM, Adetayo MO, Funmi A O, Somoye MS, Adeyemi MO, Adeyemo WL. Comparison of professional and laypeople evaluation of nasolabial esthetics following unilateral cleft lip repair. *Eur J Dent* 2018; **12**: 516–22. |
| 1085 |
| Ogundipe O, Njokanma AR. Comparison of Post-Operative Symptom Severity (PoSSe) Scores in patients undergoing Mandibular Third Molar surgery in Ile-Ife, Nigeria. *Ann Health Res (Onabanjo Univ Teach Hosp)* 2019; **5**: 29–35. |
| 1086 |
| Adetayo AM, Adetayo MO, Somoye MS, Adeyemi MO. Comparison of operative ‘difficulty’ with post-operative sequelae in lower third molar surgery. *Ann Health Res (Onabanjo Univ Teach Hosp)* 2019; **5**: 73–84. |
| 1087 |
| Kerre N, Ngesa JL, Ng’ang’a P, Kemoli AM, Bermudez J, Seminario AL. Comparison of measured and predicted mesiodistal tooth-widths of 13–17 years old Kenyans: a descriptive cross-sectional study to develop a new prediction equation for use in the mixed dentition in a Kenyan population. *BMC Oral Health* 2022; **22**. DOI:[10.1186/s12903-022-02368-y](https://doi.org/10.1186/s12903-022-02368-y). |
| 1088 |
| Oyedele TA, Adeyemo YI, Ladeji AM, Adetayo AM, Nzomiwu CL. Comparison of dental caries and oral hygiene status of children in suburban with those in rural population of southwestern Nigeria. *Pesquisa Brasileira em Odontopediatria e Clinica Integrada* 2021; **21**: 1–11. |
| 1089 |
| Diouf JS, Ouédraogo Y, Souaré N, *et al.* Comparison of dental arch measurements according to the grade and the obstructive character of adenoids. *Int Orthod* 2019; **17**: 333–41. |
| 1090 |
| Ize-Iyamu IN, Saheeb BD, Edetanlen BE. Comparing the 810nm diode laser with conventional surgery in orthodontic soft tissue procedures. *Ghana Med J* 2013; **47**: 107–11. |
| 1091 |
| Ajayi JO, Abiodun-Solanke IMF, Olusile OA, Oginni AO, Esan TA. Comparative study of treatment outcome in apicectomies with or without root-end filling. *Ann Ib Postgrad Med* 2018; **16**: 109–14. |
| 1092 |
| Osunde OD, Adebola RA, Adeoye JB, Bassey GO. Comparative study of the effect of warm saline mouth rinse on complications after dental extractions. *Int J Oral Maxillofac Surg* 2014; **43**: 649–53. |
| 1093 |
| Aikulola OO, Okoje VN, Adesina OA. Comparative study of oral health and microbial flora in pregnant and non-pregnant women in Ibadan, Nigeria. *African Journal of Biomedical Research* 2020; **23**: 31–5. |
| 1094 |
| Osunde OD, Saheeb BD, Adebola RA. Comparative study of effect of single and multiple suture techniques on inflammatory complications after third molar surgery. *J Oral Maxillofac Surg* 2011; **69**: 971–6. |
| 1095 |
| Braimah RO, Ukpong DI, Ndukwe KC, Akinyoola AL. Comparative study of anxiety and depression following maxillofacial and orthopedic injuries. Study from a Nigerian University Teaching Hospital. *Clin Exp Dent Res* 2017; **3**: 215–9. |
| 1096 |
| Umeizudike KA, Ayanbadejo PO, Savage KO, Nwhator SO, Akanmu AS, Ogunleye O. Comparative periodontal status of human immunodeficiency virus-positive patients and controls in a dedicated human immunodeficiency virus clinic in Nigeria. *Nigerian Journal of Clinical Practice* 2016; **19**: 35–40. |
| 1097 |
| Ndukwe KC, Braimah RO, Owotade JF, Aregbesola SB. Comparative Efficacy of Amoxicillin/Clavulanic Acid and Levofloxacin in the Reduction of Postsurgical Sequelae After Third Molar Surgery: A Randomized, Double-Blind, Clinical Trial in a Nigerian University Teaching Hospital. *Niger J Surg* 2016; **22**: 70–6. |
| 1098 |
| Ndukwe KC, Braimah RO, Owotade JF, Aregbesola SB. Comparative efficacy of amoxicillin/clavulanic acid and levofloxacin in the reduction of postsurgical sequelae after third molar surgery: a randomized, double-blind, clinical trial in a Nigerian university teaching hospital. *Niger j surg (Online)* 2017; **22**: 70–6. |
| 1099 |
| Hlongwa P, Dandajena TC, Rispe LC. Comparative analysis of healthcare provision to individuals with cleft lip and/or palate at specialised academic centres in South Africa. *South African Medical Journal* 2019; **109**: 426–30. |
| 1100 |
| Akinbade AO, Ndukwe KC, Owotade FJ. Comparative analgesic efficacy and tolerability of celecoxib and tramadol on postoperative pain after mandibular third molar extraction: A double blind randomized controlled trial. *Niger J Clin Pract* 2019; **22**: 796–800. |
| 1101 |
| Akinbade AO, Ndukwe KC, Owotade FJ. Comparative Analgesic Effects of Ibuprofen, Celecoxib and Tramadol after third Molar Surgery: A Randomized Double Blind Controlled Trial. *J Contemp Dent Pract* 2018; **19**: 1334–40. |
| 1102 |
| Adebola RA, Bamgbose BO, Adeoye JB. Community Mobilization and Awareness Creation for Orofacial Cleft Services: A Survey of Nigerian Cleft Service Providers. *Int Sch Res Notices* 2014; **2014**: 140713. |
| 1103 |
| Kebede A, Retta N, Abuye C, Malde MK. Community knowledge, attitude and practices (KAP) on fluorosis and its mitigation in endemic areas of Ethiopia. *African Journal of Food, Agriculture, Nutrition and Development* 2016; **16**: 10711–22. |
| 1104 |
| Tobin AO, Ajayi IO. Common oral conditions and correlates: An oral health survey in Kwara State Nigeria. *BMC Research Notes* 2017; **10**. DOI:[10.1186/s13104-017-2894-0](https://doi.org/10.1186/s13104-017-2894-0). |
| 1105 |
| Oyedele TA, Folayan MO, Adekoya-Sofowora CA, Oziegbe EO. Co-morbidities associated with molar-incisor hypomineralisation in 8 to 16 year old pupils in Ile-Ife, Nigeria. *BMC Oral Health* 2015; **15**. DOI:[10.1186/s12903-015-0017-7](https://doi.org/10.1186/s12903-015-0017-7). |
| 1106 |
| Effiom OA, Adewole RA, Odukoya O. Clinicopathological characteristics of odontogenic myxoma in Nigerians. *West African Journal of Medicine* 2011; **30**: 255–61. |
| 1107 |
| Adisa AO, Adeyemi BF, Oluwasola AO, Kolude B, Akang EEU, Lawoyin JO. Clinico-pathological profile of head and neck malignancies at University College Hospital, Ibadan, Nigeria. *Head and Face Medicine* 2011; **7**. DOI:[10.1186/1746-160X-7-9](https://doi.org/10.1186/1746-160X-7-9). |
| 1108 |
| Mujtaba B, Chimezie CB, Adebayo AI, Taiwo AO, Ndubuizu GU, Fawa AS. Clinico-pathological analysis of osteomyelitis in cancrum ois (Noma) patients seen in Noma children hospital, Northwest Nigeria. *Niger J Dent Res (Online)* 2022; **7**: 29–34. |
| 1109 |
| Kamau MW, Chindia ML, Dimba EAO, Awange D, Gathece L. Clinico-histopathologic types of maxillofacial malignancies with emphasis on sarcomas: A 10-year review. *East African Medical Journal* 2011; **88**: 39–45. |
| 1110 |
| Afolabi AO, Shaba OP, Adegbulugbe IC. Clinical investigation of patient related factors in non carious cervical lesions. *Nig Q J Hosp Med* 2013; **23**: 129–34. |
| 1111 |
| Okumu SB, Chindia ML, Gathece LW, Dimba EAO, Odhiambo W. Clinical features and types of paediatric orofacial malignant neoplasms at two hospitals in Nairobi, Kenya. *Journal of Cranio-Maxillofacial Surgery* 2012; **40**: e8. |
| 1112 |
| Braimah R, Taiwo A, Ibikunle A, *et al.* Clinical experience in managing temporomandibular joint ankylosis: Five-year appraisal in a Nigerian subpopulation. *Journal of the Korean Association of Oral and Maxillofacial Surgeons* 2018; **44**: 112–9. |
| 1113 |
| Anyanechi CE, Chukwuneke FN, Ngim N. Clinical Evaluation of the Efficacy of Arthocare Forte, a Chondro-Protective and Anti-Arthritic Drug in the Management of Bacterial Plaque-Induced Chronic Periodontitis. *Ann Med Health Sci Res* 2015; **5**: 157–62. |
| 1114 |
| Adeleke None, Oginni A. Clinical evaluation of resin composite and resin-modified glass ionomer cement in non-carious cervical lesions. *Journal of the West African College of Surgeons* 2012; **2**: 21–37. |
| 1115 |
| Okechi UC, Uguru C, Obiechina A. Clinical evaluation of postoperative fever in patients that had oral and maxillofacial surgery in university of Nigeria Teaching Hospital, Ituku-Ozalla, Enugu, Nigeria. *Nigerian Journal of Clinical Practice* 2019; **22**: 181–5. |
| 1116 |
| Onwudiwe UV, Umesi DC, Orenuga OO, Shaba OP. Clinical evaluation of 16% and 35% carbamide peroxide as in-office vital tooth whitening agents. *Nig Q J Hosp Med* 2013; **23**: 80–4. |
| 1117 |
| Ayo-Yusuf OA, Postma TC, van Wyk C. Clinical correlates of oral malodour in a population of patients attending a preventive clinic in Pretoria, South Africa. *SADJ : journal of the South African Dental Association = tydskrif van die Suid-Afrikaanse Tandheelkundige Vereniging* 2011; **66**: 326, 328–31. |
| 1118 |
| Nwhator SO, Ayanbadejo PO, Umeizudike KA, *et al.* Clinical correlates of a lateral-flow immunoassay oral risk indicator. *J Periodontol* 2014; **85**: 188–94. |
| 1119 |
| Chukwuneke FN, Anyanechi CE, Akpeh JO, Chukwuka A, Ekwueme OC. Clinical characteristics and presentation of ameloblastomas: An 8-year retrospective study of 240 cases in Eastern Nigeria. *British Journal of Oral and Maxillofacial Surgery* 2016; **54**: 384–7. |
| 1120 |
| Ayele BA, Mengesha AT, Zewde YZ. Clinical characteristics and associated factors of trigeminal neuralgia: Experience from Addis Ababa, Ethiopia. *BMC Oral Health* 2020; **20**. DOI:[10.1186/s12903-020-01227-y](https://doi.org/10.1186/s12903-020-01227-y). |
| 1121 |
| Lawal FB, Oke GA. Clinical and sociodemographic factors associated with oral health knowledge, attitude, and practices of adolescents in Nigeria. *SAGE Open Med* 2020; **8**: 2050312120951066. |
| 1122 |
| Kalala-Kazadi E, Toma S, Lasserre JF, Nyimi-Bushabu F, Ntumba-Mulumba H, Brecx MC. Clinical and Microbiological Profiles of Aggressive and Chronic Periodontitis in Congolese Patients: A Cross-sectional Study. *J Int Soc Prev Community Dent* 2020; **10**: 491–7. |
| 1123 |
| Acevedo FMA, Orish VN, Kwesi AJ, Kojo OE, Emmanuel OU, Afeke I. Clinical and microbial presentations of ludwig angina in the volta regional hospital Ho Ghana. *Pakistan Journal of Medical and Health Sciences* 2018; **12**: 463–7. |
| 1124 |
| Fortes NAH, Yohannan P. Clinical and Epidemiological Profile of Oral and Maxillofacial Trauma at Two Quaternary Hospitals in Mozambique in 2016. *Annals of African Surgery* 2021; **18**: 85–9. |
| 1125 |
| Adeyemo WL, James O, Butali A. Cleft lip and palate: Parental experiences of stigma, discrimination, and social/structural inequalities. *Ann Maxillofac Surg* 2016; **6**: 195–203. |
| 1126 |
| Rakotoarison RA, Rakotoarivony AE, Rabesandratana N, *et al.* Cleft lip and palate in Madagascar 1998-2007. *British Journal of Oral and Maxillofacial Surgery* 2012; **50**: 430–4. |
| 1127 |
| daCosta OO, Isiekwe IG, Ogbonna CM. Cleft Care in a Developing Country: An Assessment of Knowledge and Attitudes of Patients/Parents of Children With an Orofacial Cleft to Orthodontic Treatment. *Cleft Palate-Craniofacial Journal* 2022; **59**: 192–9. |
| 1128 |
| Jordan RA, Hetzel P, Franke M, Markovic L, Gaengler P, Zimmer S. Class III Atraumatic Restorative Treatment (ART) in adults living in West Africa - Outcomes after 48 months. *Community Dentistry and Oral Epidemiology* 2011; **39**: 164–70. |
| 1129 |
| Bashiru BO, Udo UA. Cigarette smoking and awareness of oral health problems of tobacco use among students at the University of Port Harcourt, South-South Nigeria. *World Journal of Dentistry* 2014; **5**: 209–12. |
| 1130 |
| Eregie UJ, Enabulele JE. Choice of dental specialties among intending residents in a tertiary health institution in Nigeria. *Niger J Dent Res* 2019; **4**: 5–12. |
| 1131 |
| Masumo R, Bardsen A, Mashoto K, Åstrøm AN. Child- and family impacts of infants’ oral conditions in Tanzania and Uganda- a cross sectional study. *BMC Research Notes* 2012; **5**. DOI:[10.1186/1756-0500-5-538](https://doi.org/10.1186/1756-0500-5-538). |
| 1132 |
| Muhoozi GKM, Li K, Atukunda P, *et al.* Child saliva microbiota and caries: a randomized controlled maternal education trial in rural Uganda. *Scientific Reports* 2022; **12**. DOI:[10.1038/s41598-022-11979-y](https://doi.org/10.1038/s41598-022-11979-y). |
| 1133 |
| Aikins EA, Eigbobo JO. Child and maternal oral healthcare: An assessment of the knowledge of Nigerian and Ghanaian nurses. *East African Medical Journal* 2014; **91**: 37–43. |
| 1134 |
| Akhiwu BI, Akhiwu HO, Afolaranmi T, *et al.* Characterization of high risk human papilloma virus genotypes associated with oropharyngeal cancers in a Nigerian population. *Pan Afr Med J* 2021; **38**: 40. |
| 1135 |
| Kityamuwesi R, Muwaz L, Kasangaki A, Kajumbula H, Rwenyonyi CM. Characteristics of pyogenic odontogenic infection in patients attending Mulago Hospital, Uganda: A cross-sectional study. *BMC Microbiology* 2015; **15**. DOI:[10.1186/s12866-015-0382-z](https://doi.org/10.1186/s12866-015-0382-z). |
| 1136 |
| Dieng SN, Kanouté A, Lombrail P, Diouf M, Azogui-Levy S. Characteristics of oral health literacy in Senegal: A cross-sec-tional study among women in the Department of Pikine. *Journal of Public Health in Africa* 2022; **13**. DOI:[10.4081/jphia.2022.2114](https://doi.org/10.4081/jphia.2022.2114). |
| 1137 |
| Sofola OO, Folayan MO, Oginni AB. Changes in the prevalence of dental caries in primary school children in Lagos State, Nigeria. *Nigerian Journal of Clinical Practice* 2014; **17**: 127–33. |
| 1138 |
| Åstrøm AN, Mashoto KO. Changes in oral health related knowledge, attitudes and behaviours following school based oral health education and atraumatic restorative treatment in rural Tanzania. *Norsk Epidemiologi* 2012; **22**: 21–30. |
| 1139 |
| Mbawalla H, Masalu JR, Masatu M, Åstrom AN. Changes in adolescents’ oral health status following oral health promotion activities in Tanzania. *Acta Odontologica Scandinavica* 2013; **71**: 333–42. |
| 1140 |
| Reddy M. Challenges Implementing Oral Health Promotion at Schools: Perspectives of Teachers and Health Managers. *Early Childhood Education Journal* 2019; **47**: 207–16. |
| 1141 |
| Mtenga AA, Kalyanyama BM, Owibingire SS, Sohal KS, Simon ENM. Cervicofacial necrotizing fasciitis among patients attending the Muhimbili National Hospital, Dar es Salaam, Tanzania. *BMC Infectious Diseases* 2019; **19**. DOI:[10.1186/s12879-019-4267-x](https://doi.org/10.1186/s12879-019-4267-x). |
| 1142 |
| Fomete B, Agbara R, Osunde DO, Ononiwu CN. Cervicofacial infection in a Nigerian tertiary health institution: a retrospective analysis of 77 cases. *J Korean Assoc Oral Maxillofac Surg* 2015; **41**: 293–8. |
| 1143 |
| Lawson Afouda S, Avakoudjo F, Alamou S, *et al.* [Cervicofacial cellulitis of dental origin: etiology, epidemiological and therapeutic aspects]. *Revue de laryngologie - otologie - rhinologie* 2012; **133**: 197–200. |
| 1144 |
| Ouédraogo Y, Benyahia H, Diouf JS, Camara T, Bationo R, Ngom PI. Cephalometric norms of a Burkina Faso population. *International Orthodontics* 2019; **17**: 136–42. |
| 1145 |
| Adesina BA, Otuyemi OD, Ogunbanjo BO, Otuyemi DO. Cephalometric assessment of hyoid bone position in Nigerian patients with bimaxillary incisor proclination. *J West Afr Coll Surg* 2016; **6**: 117–35. |
| 1146 |
| Idowu AE, Adedapo AO, Akhiwu BI, Agbara R, Olaniyi TO, Alufohai OO. Causes of Dental Trauma: Results of Findings Among Patients in a Secondary Oral Healthcare Center, Jos, Nigeria. *J West Afr Coll Surg* 2021; **11**: 19–24. |
| 1147 |
| Olatosi OO, Sote EO. Causes and pattern of tooth loss in children and adolescents in a Nigerian tertiary hospital. *Nigerian quarterly journal of hospital medicine* 2012; **22**: 258–62. |
| 1148 |
| Jordan RA, Schulte A, Bockelbrink AC, *et al.* Caries-Preventive Effect of Salt Fluoridation in Preschool Children in the Gambia: A Prospective, Controlled, Interventional Study. *Caries Research* 2018; **51**: 596–604. |
| 1149 |
| Thekiso M, Yengopal V, Rudolph MJ, Bhayat A. Caries status among children in the West Rand District of Gauteng Province, South Africa. *SADJ : journal of the South African Dental Association = tydskrif van die Suid-Afrikaanse Tandheelkundige Vereniging* 2012; **67**: 318–20. |
| 1150 |
| Folayan MO, Sofola OO, Oginni AB. Caries incidence in a cohort of primary school students in Lagos state, Nigeria followed up over a 3 years period. *European Archives of Paediatric Dentistry* 2012; **13**: 312–8. |
| 1151 |
| Ligali TO, Orenuga OO, Oredugba FA. Caries impact on quality of life among visually impaired adolescents: A cross-sectional study. *Special Care in Dentistry* 2020; **40**: 184–91. |
| 1152 |
| Birungi N, Fadnes LT, Engebretsen IMS, Tumwine JK, Lie SA, Åstrøm AN. Caries experience by socio-behavioural characteristics in HIV-1-infected and uninfected Ugandan mothers–a multilevel analysis. *Acta Odontologica Scandinavica* 2022; **80**: 91–8. |
| 1153 |
| Songo BF, Declerck D, Vinckier F, Mbuyi MD, Pilipili CM, Kayembe KP. Caries experience and related factors in 4-6 year-olds attending dental clinics in Kinshasa, DR of Congo. *Community Dental Health* 2013; **30**: 257–62. |
| 1154 |
| Birungi N, Fadnes LT, Engebretsen IMS, Lie SA, Tumwine JK, Åstrøm AN. Caries experience and oral health related quality of life in a cohort of Ugandan HIV-1 exposed uninfected children compared with a matched cohort of HIV unexposed uninfected children. *BMC Public Health* 2020; **20**. DOI:[10.1186/s12889-020-08564-1](https://doi.org/10.1186/s12889-020-08564-1). |
| 1155 |
| Cheng J, Campbell K. Caries and dental erosion: Are soroti children and adolescents at risk from increased soft-drink availability in Uganda? *African Health Sciences* 2016; **16**: 943–6. |
| 1156 |
| Birungi N, Fadnes LT, Nankabirwa V, Tumwine JK, Åstrøm AN. Caretaker’s caries experience and its association with early childhood caries and children’s oral health-related quality of life: A prospective two-generation study. *Acta Odontologica Scandinavica* 2016; **74**: 605–12. |
| 1157 |
| Adeniyi AA, Diaku-Akinwumi IN, Ola BA. Caregivers′ perception of oral health-related quality of life in a group of Nigerian children living with human immunodeficiency virus. *Nigerian Journal of Clinical Practice* 2016; **19**: 368–74. |
| 1158 |
| Nqcobo C, Ralephenya T, Kolisa YM, Esan T, Yengopal V. Caregivers’ perceptions of the oral-health-related quality of life of children with special needs in johannesburg, south africa. *Health SA Gesondheid* 2019; **24**. DOI:[10.4102/hsag.v24i0.1056](https://doi.org/10.4102/hsag.v24i0.1056). |
| 1159 |
| Njunda AL, Nsagha DS, Assob JCN, Kamga HLN, Palle JN, Teyim P. Candidiasis in HIV and AIDS patients attending the Nylon Health District hospital in Douala, Cameroon. *TAF Preventive Medicine Bulletin* 2011; **10**: 701–6. |
| 1160 |
| Esebelahie NO, Enweani IB, Omoregie R. Candida colonisation in asymptomatic HIV patients attending a tertiary hospital in Benin City, Nigeria. *Libyan Journal of Medicine* 2013; **8**. DOI:[10.3402/ljm.v8i0.20322](https://doi.org/10.3402/ljm.v8i0.20322). |
| 1161 |
| Singh S. Can undergraduate student learning in prevention influence oral health self-care practices? – a report from a South African University. *International Journal of Dental Hygiene* 2017; **15**: e100–12. |
| 1162 |
| Oyapero A, Edomwonyi AI, Adeniyi AA, Olatosi OO. Can oral health-related quality of life be worsened by dental appointments? *Dent Res J (Isfahan)* 2020; **17**: 395–403. |
| 1163 |
| Ogundipe OK, Ugboko VI, Owotade FJ. Can autologous platelet-rich plasma gel enhance healing after surgical extraction of mandibular third molars? *J Oral Maxillofac Surg* 2011; **69**: 2305–10. |
| 1164 |
| Agbor MA, Azodo CC. Cameroonian dentists’ opinion on training and quality of dental services rendered by dental auxiliaries. *Annals of African Surgery* 2011; **7**: 38–41. |
| 1165 |
| Achembong LN, Ashu AM, Hagopian A, Downer A, Barnhart S. Cameroon mid-level providers offer a promising public health dentistry model. *Human Resources for Health* 2012; **10**. DOI:[10.1186/1478-4491-10-46](https://doi.org/10.1186/1478-4491-10-46). |
| 1166 |
| Gbolahan OO, Olowookere SA, Aladelusi TO, *et al.* Burdens and predictors of the Burden experienced by Family Caregivers of Patients with Oral and Maxillofacial Tumours. *J West Afr Coll Surg* 2021; **11**: 18–25. |
| 1167 |
| Oyapero A, Oyapero O, Akinleye AI. Burden of tobacco, kola nut and alcohol consumption and its association with periodontal disease, potentially malignant lesions and quality of life among bus drivers, Lagos State, Nigeria. *Population Medicine* 2020; **2**. DOI:[10.18332/popmed/118726](https://doi.org/10.18332/popmed/118726). |
| 1168 |
| Morgan JP, Isyagi M, Ntaganira J, *et al.* Building oral health research infrastructure: the first national oral health survey of Rwanda. *Global Health Action* 2018; **11**. DOI:[10.1080/16549716.2018.1477249](https://doi.org/10.1080/16549716.2018.1477249). |
| 1169 |
| Oziegbe EO, Esan TA, Oyedele TA. Brief communication: Emergence chronology of permanent teeth in Nigerian children. *American Journal of Physical Anthropology* 2014; **153**: 506–11. |
| 1170 |
| Adekunle AA, Adamson O, James O, Ogunlewe OM, Butali A, Adeyemo WL. Breastfeeding Practices Among Mothers of Children With Orofacial Clefts in an African Cohort. *Cleft Palate-Craniofacial Journal* 2020; **57**: 1018–23. |
| 1171 |
| Agbaje JO, Olumuyiwa Adisa A, Ivanova Petrova M, *et al.* Biological profile of ameloblastoma and its location in the jaw in 1246 Nigerians. *Oral Surgery, Oral Medicine, Oral Pathology and Oral Radiology* 2018; **126**: 424–31. |
| 1172 |
| Abdennour S, Benhalima H. [Benign odontogenic tumours: epidemiological analysis of 97 cases in the Algerian population]. *Rev Stomatol Chir Maxillofac Chir Orale* 2013; **114**: 67–71. |
| 1173 |
| Dube NC, Moshy JR, Vuhahula EA, Sohal KS. Benign fibro-osseous lesions of the jaws: A clinicopathologic study of 98 Tanzanian patients. *Journal of Oral Medicine and Oral Surgery* 2019; **25**. DOI:[10.1051/mbcb/2019026](https://doi.org/10.1051/mbcb/2019026). |
| 1174 |
| Mbawalla HS, Khamis SM, Kahabuka FK. Behavioural and sociodemographic determinants of oral health-related quality of life among adolescents in Zanzibar, Tanzania. *Oral Health and Preventive Dentistry* 2019; **17**: 219–25. |
| 1175 |
| Workie MS, Belay DB. Bayesian model with application to a study of dental caries. *BMC Oral Health* 2019; **19**. DOI:[10.1186/s12903-018-0687-z](https://doi.org/10.1186/s12903-018-0687-z). |
| 1176 |
| Esan TA, Oziegbe EO. Barriers to restorative care as perceived by dentists in Nigeria. *Oral Health and Preventive Dentistry* 2013; **11**: 303–7. |
| 1177 |
| Ajayi DM, Arigbede AO. Barriers to oral health care utilization in Ibadan, South West Nigeria. *African Health Sciences* 2012; **12**: 507–13. |
| 1178 |
| Onyejaka NK, Folayan MO, Folaranmi N. Barriers and facilitators of dental service utilization by children aged 8 to 11 years in Enugu State, Nigeria. *BMC Health Services Research* 2016; **16**. DOI:[10.1186/s12913-016-1341-6](https://doi.org/10.1186/s12913-016-1341-6). |
| 1179 |
| John CN, Xavier Graham Stephen L, Wilma Joyce Africa C. BANA-Positive Plaque Samples Are Associated with Oral Hygiene Practices and Not CD4+ T Cell Counts in HIV-Positive Patients. *Int J Dent* 2012; **2012**: 157641. |
| 1180 |
| Maduakor UC, Onyemelukwe NF, Maduakor SN, Azubuike NC, Onyemelukwe AO, Nnedu EB. Bacterial etiology and risk factors of periodontal diseases in enugu metropolis, south east nigeria. *Internet Journal of Microbiology* 2019; **16**. DOI:[10.5580/IJMB.54104](https://doi.org/10.5580/IJMB.54104). |
| 1181 |
| Owotade FJ, Ogundipe OK, Ugboko VI, *et al.* Awareness, knowledge and attitude on cleft lip and palate among antenatal clinic attendees of tertiary hospitals in Nigeria. *Niger J Clin Pract* 2014; **17**: 6–9. |
| 1182 |
| Obuna JA, Ugboma HAA, Igbinedion H, Ejikeme BN, Agwu UM, Ugboma EW. Awareness of pregnancy-related oral diseases in women attending antenatal clinics in a university teaching hospital in Nigeria. *International Journal of Tropical Medicine* 2012; **7**: 61–3. |
| 1183 |
| Ajayi YO, Akinboboye BO, Dosumu OO, Akeredolu PA. Awareness of dental implants among dental patients in Nigeria. *Journal of Medicine and Biomedical Research* 2016; **15**: 40–8. |
| 1184 |
| Sulaiman AO, Kanmodi KK, Amoo BA. Awareness and perceived need of Nigerians on the restorative treatment for a defective tooth: A school survey. *Journal of Pain Management* 2018; **11**: 417–23. |
| 1185 |
| Paurobally N, Kruger E, Tennant M. Awareness About the Oral and Systemic Complications of Diabetes Among a Cohort of Diabetic Patients of the Republic of Mauritius. *International Dental Journal* 2021; **71**: 438–48. |
| 1186 |
| Koyio LN, Kikwilu E, Mulder J, Frencken JE. Attitudes, subjective norms, and intention to perform routine oral examination for oropharyngeal candidiasis as perceived by primary health-care providers in Nairobi Province. *Journal of Public Health Dentistry* 2013; **73**: 127–34. |
| 1187 |
| Nyamuryekung’e KK, Lahti SM, Tuominen RJ. Attitudes towards tooth fillings in Tanzanian adults and its association with previous filling experience. *BMC Oral Health* 2018; **18**. DOI:[10.1186/s12903-018-0474-x](https://doi.org/10.1186/s12903-018-0474-x). |
| 1188 |
| Bankole OO, Olanloye OM, Ayebameru OE, Popoola BO. Attitude of Some Nigerian Parents toward their Presence in the Operatory during Dental Treatment of their Children. *Int J Clin Pediatr Dent* 2021; **14**: S167–72. |
| 1189 |
| Adeyemi AT, Bankole OO. Attitude of cleft care specialists in africa towards presurgical orthopaedics. *East African Medical Journal* 2012; **89**: 414–20. |
| 1190 |
| Gbolahan OO, Fasola AO, Aladelusi TO. Attitude and Behavior to Oral Health of 456 Patients Who Presented for Tooth Extraction at 2 Health Facilities in Southwestern Nigeria. *J Patient Exp* 2019; **6**: 157–63. |
| 1191 |
| Akaji EA, Chukwuneke FN, Okeke UF. Attendance pattern amongst patients at the Dental Clinic of the University of Nigeria Teaching Hospital, Enugu, Nigeria. *Nigerian journal of medicine : journal of the National Association of Resident Doctors of Nigeria* 2012; **21**: 74–7. |
| 1192 |
| Fadeju AD, Otuyemi OD, Ngom PI, Newman-Nartey M. Astudy of cephalometric soft tissue profile among adolescents from the three West African countries of Nigeria, Ghana and Senegal. *Journal of Orthodontics* 2013; **40**: 53–61. |
| 1193 |
| Wagaiyu EG, Bulimo WD, Wanzala PN, Kaimenyi JT. Associations of Interleukin-1 Polymorphisms with Chronic Periodontitis in Two Different Kenyan Ethnic Groups: A Case Control Study. *J Int Acad Periodontol* 2018; **20**: 65–76. |
| 1194 |
| Folayan MO, Ibigbami OI, Oloniniyi IO, Oginni O, Aloba O. Associations between psychological wellbeing, depression, general anxiety, perceived social support, tooth brushing frequency and oral ulcers among adults resident in Nigeria during the first wave of the COVID-19 pandemic. *BMC Oral Health* 2021; **21**. DOI:[10.1186/s12903-021-01871-y](https://doi.org/10.1186/s12903-021-01871-y). |
| 1195 |
| Adeniyi AA, Folayan MO, Arowolo O, Oziegbe EO, Chukwumah NM, El-Tantawi M. Associations between oral habits, dental anxiety, dental service utilization, and maternal mental health status among 6- to 12-year-old children in Ile-Ife, Nigeria. *European Archives of Paediatric Dentistry* 2022. DOI:[10.1007/s40368-022-00767-x](https://doi.org/10.1007/s40368-022-00767-x). |
| 1196 |
| Folayan MO, Arowolo O, Mapayi B, *et al.* Associations between mental health problems and risky oral and sexual behaviour in adolescents in a sub-urban community in Southwest Nigeria. *BMC Oral Health* 2021; **21**. DOI:[10.1186/s12903-021-01768-w](https://doi.org/10.1186/s12903-021-01768-w). |
| 1197 |
| Folayan MO, Tantawi ME, Chukwumah NM, *et al.* Associations between depression and gingivitis among adolescents resident in semi-urban South-West Nigeria. *BMC Oral Health* 2021; **21**. DOI:[10.1186/s12903-021-01421-6](https://doi.org/10.1186/s12903-021-01421-6). |
| 1198 |
| Diendéré J, Zeba AN, Kiemtoré S, *et al.* Associations between dental problems and underweight status among rural women in Burkina Faso: results from the first WHO Stepwise Approach to Surveillance (STEPS) survey. *Public Health Nutr* 2021; : 1–11. |
| 1199 |
| Folayan MO, El Tantawi M, Aly NM, *et al.* Associations between a history of sexual abuse and dental anxiety, caries experience and oral hygiene status among adolescents in sub-urban South West Nigeria. *BMC Oral Health* 2021; **21**. DOI:[10.1186/s12903-021-01562-8](https://doi.org/10.1186/s12903-021-01562-8). |
| 1200 |
| Yadufashije C, Mucumbitsi J, Uwimana M, *et al.* Association with oral microbial alteration and oral disease among patients attending ruhengeri referral hospital, Rwanda: A case-control study. *Biomedical and Biotechnology Research Journal* 2022; **6**: 126–31. |
| 1201 |
| Ochanji AA, Matu NK, Mulli TK. Association of salivary RANKL and osteoprotegerin levels with periodontal health. *Clin Exp Dent Res* 2017; **3**: 45–50. |
| 1202 |
| Birungi N, Fadnes LT, Engebretsen IMS, Lie SA, Tumwine JK, Åstrøm AN. Association of maternal HIV-1 severity with dental caries: an observational study of uninfected 5- to 7-yr-old children of HIV-1-infected mothers without severe immune suppression. *European Journal of Oral Sciences* 2020; **128**: 46–54. |
| 1203 |
| Amoah PA, Koduah AO, Gyasi RM, Nyamekye KA, Phillips DR. Association of Health Literacy and Socioeconomic Status with Oral Health Among Older Adults in Ghana: A Moderation Analysis of Social Capital. *Journal of Applied Gerontology* 2022; **41**: 671–9. |
| 1204 |
| Alade G, Ayanbadejo P, Umeizudike K, Ajuluchukwu J. Association of elevated c-reactive protein with severe periodontitis in hypertensive patients in Lagos, Nigeria: A pilot study. *Contemporary Clinical Dentistry* 2018; **9**: S95–9. |
| 1205 |
| Olatosi OO, Sote EO. Association of early childhood caries with breastfeeding and bottle feeding in southwestern Nigerian children of preschool age. *J West Afr Coll Surg* 2014; **4**: 31–53. |
| 1206 |
| Gideon MN, Masiga MA, Owino R, Kahabuka FK. Association of early childhood caries experience with oral hygiene status and oral health practices of preschool children in Tandale, Tanzania. *East African Medical Journal* 2018; **95**: 1548–59. |
| 1207 |
| Mulualem D, Hailu D, Tessema M, Whiting SJ. Association of Dietary Calcium Intake with Dental, Skeletal and Non-Skeletal Fluorosis among Women in the Ethiopian Rift Valley. *International Journal of Environmental Research and Public Health* 2022; **19**. DOI:[10.3390/ijerph19042119](https://doi.org/10.3390/ijerph19042119). |
| 1208 |
| Folayan MO, Obiyan MO, Olaleye AO. Association between water, sanitation, general hygiene and oral hygiene practices of street-involved young people in Southwest Nigeria. *BMC Oral Health* 2020; **20**. DOI:[10.1186/s12903-020-1022-z](https://doi.org/10.1186/s12903-020-1022-z). |
| 1209 |
| Pockpa ZAD, Soueidan A, Koffi-Coulibaly NT, *et al.* Association Between Periodontitis and Preterm Birth in a Cohort of Pregnant Women in Ivory Coast. *Oral Health Prev Dent* 2022; **20**: 363–8. |
| 1210 |
| Umoh A, Azodo C. Association between Periodontal Status, Oral Hygiene Status and Tooth Wear among Adult Male Population in Benin City, Nigeria. *Ann Med Health Sci Res* 2013; **3**: 149–54. |
| 1211 |
| Umeizudike KA, Iwuala SO, Ozoh OB, Ayanbadejo PO, Fasanmade OA. Association between periodontal diseases and systemic illnesses: A survey among internal medicine residents in Nigeria. *Saudi Dental Journal* 2016; **28**: 24–30. |
| 1212 |
| Eno Belinga LE, Ngan WB, Lemougoum D, *et al.* Association between periodontal diseases and cardiovascular diseases in Cameroon. *Journal of Public Health in Africa* 2018; **9**: 70–3. |
| 1213 |
| Oziegbe EO, Schepartz LA. Association between parity and tooth loss among northern Nigerian Hausa women. *American Journal of Physical Anthropology* 2021; **174**: 451–62. |
| 1214 |
| Nanteza M, Tusiime JB, Kalyango J, Kasangaki A. Association between oral candidiasis and low CD4+ count among HIV positive patients in Hoima Regional Referral Hospital. *BMC Oral Health* 2014; **14**. DOI:[10.1186/1472-6831-14-143](https://doi.org/10.1186/1472-6831-14-143). |
| 1215 |
| Kayamba V, Mulenga C, Mubbunu M, Kazhila L, Hodges P, Kelly P. Association between oesophageal cancer and biomass smoke exposure: a case-control study. *Ecancermedicalscience* 2022; **16**: 1422. |
| 1216 |
| Folayan MO, Oginni AB, El Tantawi M, Alade M, Adeniyi AA, Finlayson TL. Association between nutritional status and early childhood caries risk profile in a suburban Nigeria community. *International Journal of Paediatric Dentistry* 2020; **30**: 798–804. |
| 1217 |
| El Tantawi M, Folayan MO, Oginni O, *et al.* Association between mental health, caries experience and gingival health of adolescents in sub-urban Nigeria. *BMC Oral Health* 2021; **21**. DOI:[10.1186/s12903-021-01589-x](https://doi.org/10.1186/s12903-021-01589-x). |
| 1218 |
| Folayan MO, Alade M, Adeniyi A, El Tantawi M, Finlayson TL. Association between maternal socioeconomic factors, decision-making status, and dental utilization by children with early childhood caries in sub-urban Nigeria. *Journal of Public Health Dentistry* 2020; **80**: 288–96. |
| 1219 |
| Harjunmaa U, Järnstedt J, Alho L, *et al.* Association between maternal dental periapical infections and pregnancy outcomes: results from a cross-sectional study in Malawi. *Tropical Medicine and International Health* 2015; **20**: 1549–58. |
| 1220 |
| Kolawole KA, Folayan MO. Association between malocclusion, caries and oral hygiene in children 6 to 12 years old resident in suburban Nigeria. *BMC Oral Health* 2019; **19**. DOI:[10.1186/s12903-019-0959-2](https://doi.org/10.1186/s12903-019-0959-2). |
| 1221 |
| Folayan MO, Kolawole KA, Oyedele T, *et al.* Association between knowledge of caries preventive practices, preventive oral health habits of parents and children and caries experience in children resident in sub-urban Nigeria. *BMC Oral Health* 2014; **14**. DOI:[10.1186/1472-6831-14-156](https://doi.org/10.1186/1472-6831-14-156). |
| 1222 |
| Folayan MO, Kolawole KA, Oziegbe EO, *et al.* Association between family structure and oral health of children with mixed dentition in suburban Nigeria. *Journal of Indian Society of Pedodontics and Preventive Dentistry* 2017; **35**: 134–42. |
| 1223 |
| Folayan MO, Arije O, El Tantawi M, *et al.* Association between early childhood caries and malnutrition in a sub-urban population in Nigeria. *BMC Pediatrics* 2019; **19**. DOI:[10.1186/s12887-019-1810-2](https://doi.org/10.1186/s12887-019-1810-2). |
| 1224 |
| Tefera N, Mulualem D, Baye K, *et al.* Association Between Dietary Fluoride and Calcium Intake of School-Age Children With Symptoms of Dental and Skeletal Fluorosis in Halaba, Southern Ethiopia. *Front Oral Health* 2022; **3**: 853719. |
| 1225 |
| Folayan MO, Alade M, Adeniyi A, El Tantawi M, Finlayson TL. Association between developmental dental anomalies, early childhood caries and oral hygiene status of 3-5-year-old children in Ile-Ife, Nigeria. *BMC Oral Health* 2019; **20**. DOI:[10.1186/s12903-019-0991-2](https://doi.org/10.1186/s12903-019-0991-2). |
| 1226 |
| Oyapero A, Adenaike A, Edomwonyi A, Adeniyi A, Olatosi O. Association between dental caries, odontogenic infections, oral hygiene status and anthropometric measurements of children in Lagos, Nigeria. *Brazilian Journal of Oral Sciences* 2020; **19**: 1–14. |
| 1227 |
| Denloye O, Popoola B, Ifesanya J. Association between dental caries and body mass index in 12-15 year old private school children in Ibadan, Nigeria. *Pediatric Dental Journal* 2016; **26**: 28–33. |
| 1228 |
| Folayan MO, Sowole CA. Association between breastfeeding and eruption of the first tooth in preschool children in Nigeria. *European Journal of Paediatric Dentistry* 2013; **14**: 51–4. |
| 1229 |
| Akpata ES, Adeniyi AA, Enwonwu CO, Adeleke OA, Otoh EC. Association between alcohol consumption and periodontal disease among older Nigerians in plateau state: a preliminary study. *Gerodontology* 2016; **33**: 386–94. |
| 1230 |
| Folayan MO, Oginni O, Arowolo O, El Tantawi M. Association between adverse childhood experiences, bullying, self-esteem, resilience, social support, caries and oral hygiene in children and adolescents in sub-urban Nigeria. *BMC Oral Health* 2020; **20**. DOI:[10.1186/s12903-020-01160-0](https://doi.org/10.1186/s12903-020-01160-0). |
| 1231 |
| Adesina OA, Efunkoya AA, Olaitan A, Adesina OO. Associated anomalies in cleft lip and palate: Analysis of 811 consecutive patients. *South Sudan med j* 2017; **10**: 60–3. |
| 1232 |
| Ligali TO, Folayan MO, Sheiham A. Assessment of time taken to treat dental trauma in Nigerian children. *European Archives of Paediatric Dentistry* 2011; **12**: 37–40. |
| 1233 |
| Adoga AA, Silas OA, Yaro JP, Okwori ET, Iduh AA, Mgbachi CJ. Assessment of the sociodemographic characteristics and efficacy of screening for oral, head and neck potential malignant lesions in apparently healthy adults in Jos Nigeria. *Indian Journal of Cancer* 2016; **53**: 252–5. |
| 1234 |
| Nono D, Mapley E, Rwenyonyi CM, Okullo I. Assessment of the informed consent process in the provision of dental care in Mulago hospital, Uganda. *BMC Oral Health* 2022; **22**: 501. |
| 1235 |
| Adesina BA, Otuyemi OD, Kolawole KA, Adeyemi AT. Assessment of the impact of tongue size in patients with bimaxillary protrusion. *International Orthodontics* 2013; **11**: 221–32. |
| 1236 |
| Adeosun PO, Fatusi OA, Adedeji TA. Assessment of Severity of Illness and Monitoring Response to Treatment of Odontogenic Space Infection Using Serum Prealbumin. *Journal of Maxillofacial and Oral Surgery* 2019; **18**: 106–11. |
| 1237 |
| Tafere Y, Chanie S, Dessie T, Gedamu H. Assessment of prevalence of dental caries and the associated factors among patients attending dental clinic in Debre Tabor general hospital: A hospital-based cross-sectional study. *BMC Oral Health* 2018; **18**. DOI:[10.1186/s12903-018-0581-8](https://doi.org/10.1186/s12903-018-0581-8). |
| 1238 |
| Adamson OO, Gbotolorun OM, Odeniyi O, Oduyebo OO, Adeyemo WL. Assessment of predictors of treatment outcome among patients with bacterial odontogenic infection. *Saudi Dent J* 2018; **30**: 337–41. |
| 1239 |
| Odai ED, Ehizele AO, Enabulele JE. Assessment of pain among a group of Nigerian dental patients Oral Health. *BMC Research Notes* 2015; **8**. DOI:[10.1186/s13104-015-1226-5](https://doi.org/10.1186/s13104-015-1226-5). |
| 1240 |
| Kolawole KA, Otuyemi OD, Oluwadaisi AM. Assessment of oral health-related quality of life in Nigerian children using the Child Perceptions Questionnaire (CPQ 11-14). *European Journal of Paediatric Dentistry* 2011; **12**: 55–9. |
| 1241 |
| Hewlett SA, Blankson P-K, Aheto JMK, *et al.* Assessment of oral health status in a Ghanaian population: rationale, methods, and population characteristics. *BMC Oral Health* 2022; **22**. DOI:[10.1186/s12903-022-02090-9](https://doi.org/10.1186/s12903-022-02090-9). |
| 1242 |
| Kolisa Y. Assessment of oral health promotion services offered as part of maternal and child health services in the Tshwane Health District, Pretoria, South Africa. *African Journal of Primary Health Care and Family Medicine* 2016; **8**. DOI:[10.4102/phcfm.v8i1.794](https://doi.org/10.4102/phcfm.v8i1.794). |
| 1243 |
| Alagba GY, Benson Chukwunweike E-E, Dotimi DA. Assessment of Oral Health Problems among the Elderly Populace of Ikibiri Community; Yenagoa Local Government Area of Bayelsa State and Possible Solutions. *cont j nurs sci* 2013; **5**: 21–9. |
| 1244 |
| Goyal S, Muhigana A. Assessment of malocclusion severity levels and orthodontic treatment needs using the Dental Aesthetic Index (DAI): A retrospective study. *Rwanda Medical Journal* 2013; **70**: 20–7. |
| 1245 |
| Akinbami BO, Onajin-Obembe B. Assessment of Intraoperative Blood Loss during Oral and Maxillofacial Surgical Procedures in a Nigerian Tertiary Health Care Center. *J Blood Transfus* 2014; **2014**: 301467. |
| 1246 |
| Giwa AS, Memon AG, Ahmad J, *et al.* Assessment of high fluoride in water sources and endemic fluorosis in the North-Eastern communities of Gombe State, Nigeria. *Environmental Pollutants and Bioavailability* 2021; **33**: 31–40. |
| 1247 |
| Rwenyonyi CM, Muwazi LM, Buwembo W. Assessment of factors associated with dental caries in rural communities in Rakai District, Uganda. *Clinical Oral Investigations* 2011; **15**: 75–80. |
| 1248 |
| Daouda F, Aida K, Mbacke L, Mamadou M. Assessment of dental caries prevention program applied to a cohort of elementary school children of Kebemer, a city in Senegal. *Journal of International Society of Preventive and Community Dentistry* 2016; **6**: S105–10. |
| 1249 |
| Ibiyemi O, Bankole OO, Oke GA. Assessment of Atraumatic Restorative Treatment (ART) on the permanent dentition in a primary care setting in Nigeria. *International Dental Journal* 2011; **61**: 2–6. |
| 1250 |
| Olayemi AB. Assessment and determination of human mandibular and dental arch profiles in subjects with lower third molar impaction in Port Harcourt, Nigeria. *Ann Maxillofac Surg* 2011; **1**: 126–30. |
| 1251 |
| Razanamihaja N, Ranivoharilanto E. Assessing the validity and reliability of the Malagasy version of Oral Impacts on Daily Performance (OIDP): a cross-sectional study. *Biopsychosoc Med* 2017; **11**: 2. |
| 1252 |
| Uwambaye P, Munyanshongore C, Rulisa S, Shiau H, Nuhu A, Kerr MS. Assessing the association between periodontitis and premature birth: a case-control study. *BMC Pregnancy and Childbirth* 2021; **21**. DOI:[10.1186/s12884-021-03700-0](https://doi.org/10.1186/s12884-021-03700-0). |
| 1253 |
| Akaji E, Ikechebelu Q, Osadolor O. Assessing dental caries and related factors in 12-year-old Nigerian school children: Report from a Southeastern State. *European Journal of General Dentistry* 2020; **9**: 11–6. |
| 1254 |
| Birungi N, Fadnes LT, Kasangaki A, *et al.* Assessing causal effects of early life-course factors on early childhood caries in 5-year-old Ugandan children using directed acyclic graphs (DAGs): A prospective cohort study. *Community Dentistry and Oral Epidemiology* 2017; **45**: 512–21. |
| 1255 |
| Assogba SD, Djossou DM, Adjibi S, Alao MJ. Aspects épidemiologiques des anomalies dentaires chez les enfants porteurs de syndrome de down au CNHU-HKM de Cotonou. *Journal de la Société de Biologie Clinique du Bénin* 2018; **28**: 26–9. |
| 1256 |
| Idon PI, Ikusika OF, Sotunde OA, Ogundare TO. Are there associations between the occurrence of dental fluorosis and the experience of dentine hypersensitivity? A cross-sectional study. *The Nigerian postgraduate medical journal* 2022; **29**: 161–6. |
| 1257 |
| Gbotolorun OM, Dipo-Fagbemi IM, Olojede AO, Ebigwei S, Adetoye JO. Are systemic antibiotics necessary in the prevention of wound healing complications after intra-alveolar dental extraction? *Int J Oral Maxillofac Surg* 2016; **45**: 1658–64. |
| 1258 |
| Oyapero A, Olatosi OO, Olagundoye O. Are Nigerian oral health workers overlooking opportunities to promote interventions for tobacco smoking cessation? *Population Medicine* 2021; **3**. DOI:[10.18332/popmed/132292](https://doi.org/10.18332/popmed/132292). |
| 1259 |
| Paurobally N, Kruger E, Tennant M. Are diabetes and dental care providers in the Republic of Mauritius advising patients about the importance of oral health in diabetes management? *International Journal of Dental Hygiene* 2021; **19**: 184–92. |
| 1260 |
| De Tove MMS, Bakayoko-Ly R, N’guessan KA, Koné K, Affiba EN-O, Aka LK. Appréciation du préjudice de la malnutrition protéino-énergétique sur la santé bucco-dentaire de l’enfant. *Medecine Buccale Chirurgie Buccale* 2013; **19**: 7–10. |
| 1261 |
| Onyejaka NK, Emele IE, Eboh OF. Appointment failure among dental patients attending a government dental centre in Enugu, Nigeria. *Pesquisa Brasileira em Odontopediatria e Clinica Integrada* 2018; **18**. DOI:[10.4034/PBOCI.2018.181.56](https://doi.org/10.4034/PBOCI.2018.181.56). |
| 1262 |
| Kawia HM, Mbawalla HS, Kahabuka FK. Application of behavior management techniques for paediatric dental patients by Tanzanian dental practitioners. *Open Dentistry Journal* 2015; **9**: 455–61. |
| 1263 |
| Birungi N, Fadnes LT, Engebretsen IMS, Tumwine JK, Åstrøm AN. Antiretroviral treatment and its impact on oral health outcomes in 5 to 7 year old Ugandan children: A 6 year follow-up visit from the ANRS 12174 randomized trial. *Medicine* 2020; **99**: e22352. |
| 1264 |
| Okoje-Adesomoju VN, Ifesanya JU, Alonge TO. Antimicrobial susceptibility pattern of oral microbial isolates among pregnant women in Ibadan South-East Local Government Area, Nigeria. *Afr j biomed res* 2016; **18**: 181–7. |
| 1265 |
| Lalloo R, Solanki G, Ramphoma K, Myburgh NG. Antibiotic-prescribing patterns of South African dental practitioners following tooth extractions. *Journal of investigative and clinical dentistry* 2017; **8**. DOI:[10.1111/jicd.12247](https://doi.org/10.1111/jicd.12247). |
| 1266 |
| Krah-Sinan A, Adou-Assoumou M, Adou J, Traoré DK, Faye B, Mansilla E. Antibiotic therapy in Endodontics: A survey from dental surgeons in Ivory Coast. *Giornale Italiano di Endodonzia* 2019; **33**: 21–6. |
| 1267 |
| Mbuyi-Musanzayi S, Tshilombo Katombe F, Lukusa Tshilobo P, Kalenga Mwenze Kayamba P, Devriendt K, Reychler H. Anthropometric and aesthetic outcomes for the nasolabial region in 101 consecutive African children with unilateral cleft lip one year after repair using the anatomical subunit approximation technique. *Int J Oral Maxillofac Surg* 2017; **46**: 1338–45. |
| 1268 |
| Utomi IL, Onyeaso CO. Anteroposterior, vertical and space malocclusions in adolescents with special needs in Lagos, Nigeria. *Odonto-stomatologie tropicale = Tropical dental journal* 2011; **34**: 17–23. |
| 1269 |
| Schnetler C, Todorovic VS, van Zyl AW. Anterior Mandibular Lingual Defect As a Possible Cause of Near-Fatal Bleeding During Routine Dental Implant Surgery: A Retrospective Computed Tomography Study. *Implant Dent* 2018; **27**: 254–9. |
| 1270 |
| Nwhator SO, Umeizudike KA, Ayanbadejo PO, Opeodu o. I, Olamijulo JA, Sorsa T. Another reason for impeccable oral hygiene: Oral hygiene-sperm count link. *Journal of Contemporary Dental Practice* 2015; **15**: 352–8. |
| 1271 |
| Adenekan AT, Faponle AF, Oginni FO. Anesthetic challenges in oro-facial cleft repair in ile-ife, Nigeria. *Middle East Journal of Anesthesiology* 2011; **21**: 335–40. |
| 1272 |
| Osemwenkhae CE, Ize­Iyamu IN, Otaren J. Analyzing dental arch shape in untreated orthodontic patients with anterior arch crowding. *Niger J Dent Res* 2020; **5**: 108–17. |
| 1273 |
| Chukwumah N, Azodo C, Orikpete E. Analysis of tooth mortality among nigerian children in a tertiary hospital setting. *Ann Med Health Sci Res* 2014; **4**: 345–9. |
| 1274 |
| Songa MAS, Saliba NA, Saliba TA, Chiba FY, Moimaz SAS. Analysis of the Dental Caries Epidemiological Profile in Children of Benguela city, Angola. *Oral Health and Preventive Dentistry* 2022; **20**: 141–8. |
| 1275 |
| Ademe D, Admassu D, Balakrishnan S. Analysis of salivary level Lactobacillus spp. And associated factors as determinants of dental caries amongst primary school children in Harar town, eastern Ethiopia. *BMC Pediatrics* 2020; **20**. DOI:[10.1186/s12887-020-1921-9](https://doi.org/10.1186/s12887-020-1921-9). |
| 1276 |
| Touré B, Faye B, Kane AW, Lo CM, Niang B, Boucher Y. Analysis of reasons for extraction of endodontically treated teeth: a prospective study. *J Endod* 2011; **37**: 1512–5. |
| 1277 |
| Akinbami BO, Nsirim PE. Analysis of occlusal vertical dimension and mandibular Basal bone height in a nigerian population. *Anat Res Int* 2014; **2014**: 584508. |
| 1278 |
| Umanah AU, Azodo C. Analysis of Nigerian dentists’ opinion and consequences on expanded function dental auxiliaries. *Odonto-stomatologie tropicale = Tropical dental journal* 2015; **38**: 37–45. |
| 1279 |
| Benjamin F, Ezekiel AT, Kelvin OU, Albert OU, Rowlan A, Emmanuel AR. Analysis of 10 cases of orbital infections arising from odontogenic cervicofacial infections. *Niger J Clin Pract* 2021; **24**: 546–50. |
| 1280 |
| Rai A, Rai V, Berhanu T. Analysing the Social Responsibility among Dentists of Ethiopia: A Cross-sectional Study. *Open Dentistry Journal* 2022; **16**. DOI:[10.2174/18742106-V16-E2205190](https://doi.org/10.2174/18742106-V16-E2205190). |
| 1281 |
| Butali A, Adeyemo WL. An overview of cleft care in Nigeria. *The Nigerian postgraduate medical journal* 2011; **18**: 151–3. |
| 1282 |
| Egbor PE, Akpata O. An evaluation of the sociodemographic determinants of dental anxiety in patients scheduled for intra-alveolar extraction. *Libyan Journal of Medicine* 2014; **9**. DOI:[10.3402/ljm.v9.25433](https://doi.org/10.3402/ljm.v9.25433). |
| 1283 |
| Arowojolu MO, Oladapo O, Opeodu OI, Nwhator SO. an evaluation of the possible relationship between chronic periodontitis and hypertension. *J West Afr Coll Surg* 2016; **6**: 20–38. |
| 1284 |
| Thomas E, Evans WG, Becker P. An evaluation of root resorption after orthodontic treatment. *SADJ* 2012; **67**: 384–9. |
| 1285 |
| Ozoemena SP, Onyejaka NK, Ani EF, Eboh OF, Odo EO. An Evaluation of a Supervised School Tooth Brushing Program on Plaque and Gingival Scores of a Group of Rural Nigerian Primary School Children. *West Afr J Med* 2022; **39**: 459–64. |
| 1286 |
| Fomete B, Agbara R, Adebayo ET, Osunde OD, Adeola DS. An epidemiological study of 270 cases of carcinomas of the head and neck region in a Nigerian tertiary health care facility. *Egyptian Journal of Ear, Nose, Throat and Allied Sciences* 2017; **18**: 251–5. |
| 1287 |
| Lawal F, Taiwo J. An audit of school oral health education program in a developing country. *Journal of International Society of Preventive and Community Dentistry* 2014; **4**: S49–55. |
| 1288 |
| Olusanya AA, Aladelusi TO, Osinaike BB, Akinloye S, Arotiba JT. An audit of oral and maxillofacial procedures under general anaesthesia at the University College Hospital Ibadan, Nigeria. *J West Afr Coll Surg* 2017; **7**: 32–56. |
| 1289 |
| Arotiba JT, Obimakinde OS, Ogunlade SO, *et al.* An audit of mandibular defect reconstruction methods in a Nigerian Tertiary Hospital. *The Nigerian postgraduate medical journal* 2011; **18**: 172–6. |
| 1290 |
| Gbotolorun OM, Emeka CI, Effiom O, Adewole RA, Ayodele AS. An Audit of Malignant Oro-facial Tumors Presenting at a Tertiary Hospital in Lagos. *Ann Med Health Sci Res* 2016; **6**: 133–6. |
| 1291 |
| Manyama M, Rolian C, Gilyoma J, *et al.* An assessment of orofacial clefts in Tanzania. *BMC Oral Health* 2011; **11**. DOI:[10.1186/1472-6831-11-5](https://doi.org/10.1186/1472-6831-11-5). |
| 1292 |
| Opeodu OI, Okechukwu A, Arowojolu MO. An assessment of knowledge of relationship between periodontal disease with pregnancy outcome and fertility among some pregnant women in Nigeria. *Niger J Dent Res* 2018; **3**. <https://search.bvsalud.org/aimafro/resource/en/biblio-1266965>. |
| 1293 |
| Salawu MM, Omitoye R. An assessment of dental care practices among undergraduate students of Adeleke University, Ede, Osun State, Nigeria. *Ann Ib Postgrad Med* 2019; **17**: 24–9. |
| 1294 |
| Ishwarkumar S, Pillay P, Chetty M, Satyapal KS. An assessment of dental age in a select South African sample using the Willems scoring systems. *Forensic Science International: Reports* 2022; **6**. DOI:[10.1016/j.fsir.2022.100296](https://doi.org/10.1016/j.fsir.2022.100296). |
| 1295 |
| Reinprecht S, van Staden PJ, Jordaan J, Bernitz H. An analysis of dental intercanine distance for use in court cases involving bite marks. *International Journal of Legal Medicine* 2017; **131**: 459–64. |
| 1296 |
| Omoregie FO, Sede MA, Ojo AM. Ameloblastomatous Change in Radicular Cyst of The Jaw in a Nigerian Population. *Ghana medical journal* 2015; **49**: 107–11. |
| 1297 |
| Okechi UC, Akpeh JO, Chukwuneke FN, *et al.* Ameloblastoma of the jaws in children: An evaluation of cases seen in a tertiary hospital in South-Eastern Nigeria. *Ghana Medical Journal* 2020; **54**: 36–41. |
| 1298 |
| Umesi DC, Oremosu OA, Makanjuola JO. Amalgam phase down: baseline data preceding implementation in Nigeria. *International Dental Journal* 2020; **70**: 161–6. |
| 1299 |
| Akpata O, Omoregie OF, Owotade F. Alveolar Osteitis: Patients’ compliance to post-extraction instructions following extraction of molar teeth. *Niger Med J* 2013; **54**: 335–8. |
| 1300 |
| Rikhotso RE, Sekhoto MG. Alloplastic Total Temporomandibular Joint Reconstruction: A 10-Year Experience of the University of the Witwatersrand, Johannesburg. *Journal of Craniofacial Surgery* 2021; **32**: 1658–63. |
| 1301 |
| Atim F, Nagaddya T, Nakaggwa F, N-Mboowa MG, Kirabira P, Okiria JC. Agony resulting from cultural practices of canine bud extraction among children under five years in selected slums of Makindye: A cross sectional study. *BMC Oral Health* 2018; **18**. DOI:[10.1186/s12903-018-0599-y](https://doi.org/10.1186/s12903-018-0599-y). |
| 1302 |
| Groenewald H, Kritzinger A, Viviers M. Age-specific communication functioning of young children with cleft lip and palate in a South African database. *Cleft Palate-Craniofacial Journal* 2013; **50**: 717–29. |
| 1303 |
| Akhigbe P, Chukwumah NM, Folayan MO, *et al.* Age-specific associations with dental caries in HIV-infected, exposed but uninfected and HIV-unexposed uninfected children in Nigeria. *BMC Oral Health* 2022; **22**. DOI:[10.1186/s12903-022-02421-w](https://doi.org/10.1186/s12903-022-02421-w). |
| 1304 |
| Olatosi OO, Onyejaka NK, Oyapero A, Ashaolu JF, Abe A. Age and reasons for first dental visit among children in Lagos, Nigeria. *The Nigerian postgraduate medical journal* 2019; **26**: 158–63. |
| 1305 |
| Fagan JJ, Otiti J, Onakoya PA, *et al.* AfHNS fellowship: Model to improve access to head and neck cancer care in Africa and developing countries. *Head and Neck* 2021; **43**: 2907–12. |
| 1306 |
| Tugaineyo EI, Odhiambo WA, Akama MK, Guthua SW, Dimba EAO. Aetiology, pattern and management of oral and maxillofacial injuries at mulago national referral hospital. *East African Medical Journal* 2012; **89**: 351–8. |
| 1307 |
| Camille A, Evelyne A-K, Martial AE, Denise K, Marie-Josée T-A, Emmanuel K. Advantages of early management of facial clefts in Africa. *International Journal of Pediatric Otorhinolaryngology* 2014; **78**: 504–6. |
| 1308 |
| Oyapero A, Bakare TI, Goncalves TF. Advancing oral health policy for mandatory dental screening before admission into public primary and secondary schools in Lagos, Nigeria. *J Family Med Prim Care* 2020; **9**: 5988–94. |
| 1309 |
| Eberlin KR, Vyas RM, Abi-Haidar Y, Sethna N, Hamdan US. Adult cleft lip repair under local anesthesia: An effective technique in resource-poor settings. *Cleft Palate-Craniofacial Journal* 2013; **50**: 59–63. |
| 1310 |
| Baratti-Mayer D, Gayet-Ageron A, Cionca N, Mossi MA, Pittet D, Mombelli A. Acute necrotising gingivitis in young children from villages with and without noma in Niger and its association with sociodemographic factors, nutritional status and oral hygiene practices: Results of a population-based survey. *BMJ Global Health* 2017; **2**. DOI:[10.1136/bmjgh-2016-000253](https://doi.org/10.1136/bmjgh-2016-000253). |
| 1311 |
| Umanah AU, Otakhoigbogie U, Soroye MO. Activated Charcoal-based Dental Products: Awareness, Knowledge and Opinion of Dental Practitioners in a Tertiary Hospital in Nigeria. *West African journal of medicine* 2020; **37**: 732–9. |
| 1312 |
| Uguru N, Onwujekwe O, Uguru CC, Ogu UU. Achieving universal health coverage in Nigeria: the dilemma of accessing dental care in Enugu state, Nigeria, a mixed methods study. *Heliyon* 2021; **7**. DOI:[10.1016/j.heliyon.2021.e05977](https://doi.org/10.1016/j.heliyon.2021.e05977). |
| 1313 |
| Isiekwe GI, DaCosta OO, Isiekwe MC. Acephalometric investigation of horizontal lip position in adult nigerians. *Journal of Orthodontics* 2012; **39**: 160–9. |
| 1314 |
| Mwesigwa CL, Kutesa AM, Munabi IG, Kabenge CA, Buwembo W. Accuracy of the lower third molar radiographic imaging to estimate age among Ugandan young people. *BMC Research Notes* 2019; **12**. DOI:[10.1186/s13104-019-4686-1](https://doi.org/10.1186/s13104-019-4686-1). |
| 1315 |
| Okoh DS, Omoregie FO, Ojo MA. Accuracy of fine needle aspiration cytology in the preoperative diagnosis of malignant non-odontogenic tumors of the orofacial region. *Diagn Cytopathol* 2021; **49**: 381–7. |
| 1316 |
| Idon PI, Sotunde OA, Ogundare TO, *et al.* Access to root canal treatment in a Nigerian sub-population: assessment of the effect of dental health insurance. *Afr Health Sci* 2021; **21**: 470–7. |
| 1317 |
| Uguru N, Onwujekwe O, Ogu UU, Uguru C. Access to Oral health care: A focus on dental caries treatment provision in Enugu Nigeria. *BMC Oral Health* 2020; **20**. DOI:[10.1186/s12903-020-01135-1](https://doi.org/10.1186/s12903-020-01135-1). |
| 1318 |
| Garcia-Marin F. Access to oral & maxillofacial surgery in Sub-Saharan African countries. *Journal of Oral Biology and Craniofacial Research* 2021; **11**: 608–11. |
| 1319 |
| Omo JO, Enablable JE. Acceptance of removable partial denture for replacement of missing teeth among partially edentulous. *Niger J Dent Res* 2019; **4**. <https://search.bvsalud.org/aimafro/resource/en/biblio-1266984>. |
| 1320 |
| Agholor CN, Ndeanaefo SU, Itimi E, Sede MA. Aberrant anatomy in endodontically treated teeth in a Nigerian Teaching Hospital. *Niger J Dent Res* 2020; **5**: 99–107. |
| 1321 |
| Teshome A, Muche A. A two-year retrospective study on the pattern of dental trauma and its etiology, Northwest Ethiopia. *Journal of Health Care for the Poor and Underserved* 2017; **28**: 216–27. |
| 1322 |
| Omeje K, Efunkoya A, Amole I, Akhiwu B, Osunde D. A two-year audit of non-vascularized iliac crest bone graft for mandibular reconstruction: technique, experience and challenges. *Journal of the Korean Association of Oral and Maxillofacial Surgeons* 2014; **40**: 272–7. |
| 1323 |
| Bhayat A, Madiba T, Nkambule N. A three-year audit of dental services at primary health care facilities in Gauteng, South Africa: 2017 to 2019. *Journal of International Society of Preventive and Community Dentistry* 2020; **10**: 452–7. |
| 1324 |
| Fomete B, Osunde OD, Ogbeifun JO. A thirteen-year retrospective analysis of 179 odontogenic tumours in a Nigerian tertiary healthcare facility. *West Indian Medical Journal* 2018; **67**: 233–7. |
| 1325 |
| Ogordi PU, Ize­Iyamu IN. A ten year audit of traumatic dental injuries in children in a Tertiary Hospital in Southern Nigeria. *Niger J Dent Res* 2020; **5**: 177–84. |
| 1326 |
| Abiola A, Olayinka A, Mathilda B, Ogunbiyi O, Modupe S, Olubunmi O. A survey of the oral health knowledge and practices of pregnant women in a Nigerian teaching hospital. *African journal of reproductive health* 2011; **15**: 14–9. |
| 1327 |
| Mgbeokwere U, Okoye L, Ekwueme O. A Survey of the Knowledge of Dental Implants as a Choice in Treatment of Edentulous Jaws among Health Workers in Government Dental Clinics in Enugu. *Ann Med Health Sci Res* 2011; **1**: 91–5. |
| 1328 |
| Azodo CC, Odai CD, Osazuwa-Peters N, Obuekwe ON. A survey of orofacial injuries among basketball players. *International Dental Journal* 2011; **61**: 43–6. |
| 1329 |
| Perrie H, Scribante J, Windsor S. A survey of oral care practices in South African intensive care units. *Southern African Journal of Critical Care* 2011; **27**: 42–6. |
| 1330 |
| Udoye CI, Sede MA, Jafarzadeh H, Abbott PV. A survey of endodontic practices among dentists in Nigeria. *Journal of Contemporary Dental Practice* 2013; **14**: 293–8. |
| 1331 |
| Kaboré WAD, Chevalier V, Gnagne-Koffi Y, Ouédraogo CDW, Ndiaye D, Faye B. A survey of endodontic practices among dentists in Burkina Faso. *Journal of Contemporary Dental Practice* 2017; **18**: 641–6. |
| 1332 |
| Okoh DS, Okoh M, Omoregie FO, Ojo MA. A study on the clinical indicators in the diagnosis of orofacial nonodontogenic tumors. *J Cancer Res Ther* 2019; **15**: 608–14. |
| 1333 |
| Edetanlen EB, Saheeb BD. A study on shotgun injuries to the craniomaxillofacial Region in a Nigerian Tertiary Health Center. *Nigerian Journal of Clinical Practice* 2018; **21**: 356–61. |
| 1334 |
| Buchanan GD, Gamieldien MY, Fabris-Rotelli I, van Schoor A, Uys A. A study of mandibular premolar root and canal morphology in a Black South African population using cone-beam computed tomography and two classification systems. *Journal of Oral Science* 2022; **64**: 300–6. |
| 1335 |
| Turton MS, Henkel RR, Africa CWJ. A simple point of care test can indicate the need for periodontal therapy to reduce the risk for adverse pregnancy outcomes in mothers attending antenatal clinics. *Biomarkers* 2017; **22**: 740–6. |
| 1336 |
| Ibiyemi O, Lawal FB. A short report on tooth replacement in an older suburban population in Nigeria. *Gerodontology* 2017; **34**: 508–11. |
| 1337 |
| Owobu T, Ojukwu BT, Azah OO, *et al.* A review of noma cases in a tertiary hospital located in a conflict endemic region in Nigeria. *Medicine, Conflict and Survival* 2022. DOI:[10.1080/13623699.2022.2114121](https://doi.org/10.1080/13623699.2022.2114121). |
| 1338 |
| Lawal A-O, Adisa A-O, Effiom O-A. A review of 640 Oral squamous cell carcinoma cases in Nigeria. *Journal of Clinical and Experimental Dentistry* 2017; **9**: e767–71. |
| 1339 |
| Anyanechi CE, Saheeb BD. A review of 156 odontogenic tumours in Calabar, Nigeria. *Ghana medical journal* 2014; **48**: 163–7. |
| 1340 |
| Ajayi DM, Abiodun-Solanke IM, Sulaiman AO, Ekhalufoh EF. A retrospective study of traumatic injuries to teeth at a Nigerian tertiary hospital. *Nigerian Journal of Clinical Practice* 2012; **15**: 320–5. |
| 1341 |
| Lawal AO, Soyele OO, Akinyamoju AO. A retrospective study of 21 cases of malignant odontogenic tumours from two tertiary health centres in Nigeria. *Pan African Medical Journal* 2015; **20**. DOI:[10.11604/pamj.2015.20.371.6611](https://doi.org/10.11604/pamj.2015.20.371.6611). |
| 1342 |
| Adisa AO, Lawal AO, Effiom OA, *et al.* A retrospective review of 61 cases of adenomatoid odontogenic tumour seen in five tertiary health facilities in Nigeria. *Pan African Medical Journal* 2016; **24**. DOI:[10.11604/pamj.2016.24.102.9400](https://doi.org/10.11604/pamj.2016.24.102.9400). |
| 1343 |
| Adeyemi BF, Kolude BM, Akang EE. A retrospective histopathological review of oral squamous cell carcinoma in a Nigerian teaching hospital. *African journal of medicine and medical sciences* 2011; **40**: 153–8. |
| 1344 |
| Folaranmi N, Okeke A. A Retrospective Evaluation of the Class of Malocclusion amongst Orthodontic Patients at the University Of Nigeria Teaching Hospital (UNTH), Enugu, Nigeria. *Ann Med Health Sci Res* 2011; **1**: 103–6. |
| 1345 |
| Benaessa MM, Mahomed F, Ngwenya SP. A retrospective clinico-pathologic analysis of cemento-osseous dysplasia in a South African patient population. *African health sciences* 2019; **19**: 3154–9. |
| 1346 |
| Gebretsadikid HG, de Kiev LC. A retrospective clinical, multi-center cross-sectional study to assess the severity and sequela of Noma/Cancrum oris in Ethiopia. *PLoS Neglected Tropical Diseases* 2022; **16**. DOI:[10.1371/JOURNAL.PNTD.0010372](https://doi.org/10.1371/JOURNAL.PNTD.0010372). |
| 1347 |
| Bello SA, Olaitan AA, Ladeinde AL. A randomized comparison of the effect of partial and total wound closure techniques on postoperative morbidity after mandibular third molar surgery. *Journal of Oral and Maxillofacial Surgery* 2011; **69**: e24–30. |
| 1348 |
| Egbor PE, Saheeb BD. A Prospective Randomized Clinical Study of the Influence of Primary Closure or Dressing on Post-operative Morbidity after Mandibular Third Molar Surgery. *Niger J Surg* 2014; **20**: 59–63. |
| 1349 |
| Oginni FO, Stoelinga PJW, Ajike SA, *et al.* A prospective epidemiological study on odontogenic tumours in a black African population, with emphasis on the relative frequency of ameloblastoma. *International Journal of Oral and Maxillofacial Surgery* 2015; **44**: 1099–105. |
| 1350 |
| Fomete B, Saheeb BD, Obiadazie AC. A prospective clinical evaluation of the longevity of resorbable sutures in oral surgical procedures. *Niger J Clin Pract* 2013; **16**: 334–8. |
| 1351 |
| Fomete B, Saheeb BD, Obiadazie AC. A prospective clinical evaluation of the effects of chlorhexidine, warm saline mouth washes and microbial growth on intraoral sutures. *J Maxillofac Oral Surg* 2015; **14**: 448–53. |
| 1352 |
| Okeigbemen SA, Jeboda SO, Umweni AA. A preliminary assessment of the periodontal status of elderly pensioners in Benin City, Nigeria. *Gerodontology* 2012; **29**: e1244–8. |
| 1353 |
| Enone L, Oyapero A, Awotile A, Ijarogbe O, Akinleye A, Dahunsi M. A preliminary assessment of endodontic difficulty encountered at a tertiary health center in Lagos, Nigeria. *Journal of International Oral Health* 2018; **10**: 303–9. |
| 1354 |
| Olaide Savage K, Oderinu OH, Adegbulugbe IC, Uti OG, Dosumu OO, Olusile AO. A national survey of tooth wear on facial and oral surfaces and risk factors in young Nigerian adults. *Eur J Dent* 2018; **12**: 292–9. |
| 1355 |
| Omitola OG, Soyele OO, Sigbeku O, *et al.* A multi-centre evaluation of oral cancer in southern and Western Nigeria: An African oral pathology research consortium initiative. *Pan African Medical Journal* 2017; **28**. DOI:[10.11604/pamj.2017.28.64.13089](https://doi.org/10.11604/pamj.2017.28.64.13089). |
| 1356 |
| Soyele OO, Effiom OA, Lawal AO, *et al.* A multi-centre evaluation of malignant odontogenic tumours in Nigeria. *Pan African Medical Journal* 2019; **33**. DOI:[10.11604/pamj.2019.33.18.16179](https://doi.org/10.11604/pamj.2019.33.18.16179). |
| 1357 |
| Matundu B, Adefolaju GA, Manda J, Mwakikunga A. A morphometric study of the mandibular foramen in dry adult human mandibles in a black malawian population. *International Journal of Morphology* 2021; **39**: 390–5. |
| 1358 |
| Bankole OO, Ibiyemi O, Lawal FB. A health education video in an indigenous Nigerian language to dispel misconceptions associated with reversal of eruption sequence of anterior teeth. *African Journal of Biomedical Research* 2019; **22**: 97–100. |
| 1359 |
| Adisa AO, Osayomi T, Effiom OA, *et al.* A geographical analysis of ethnic distribution of jaw ameloblastoma in Nigerians. *African Health Sciences* 2019; **19**: 1677–86. |
| 1360 |
| Uchenna KO, Akhiwu IB, Agbara R, Akinwale AE. A five year audit of cases admitted in maxillofacial surgery wards in a Nigerian teaching hospital. *Niger J Dent Res* 2018; **3**. <https://search.bvsalud.org/aimafro/resource/en/biblio-1266966>. |
| 1361 |
| Thema L, Singh S. A conceptual framework to guide public oral health planning in limpopo province. *Health SA Gesondheid* 2019; **24**. DOI:[10.4102/hsag.v24i0.1109](https://doi.org/10.4102/hsag.v24i0.1109). |
| 1362 |
| Tootla S, Owen CP. A comparison of endodontic treatment outcomes between HIV-positive and HIV-negative patients. *SADJ* 2012; **67**: 322–5. |
| 1363 |
| Khammissa R, Feller L, Altini M, Fatti P, Lemmer J. A comparison of chronic periodontitis in HIV-seropositive subjects and the general population in the Ga-Rankuwa Area, South Africa. *AIDS Research and Treatment* 2012; **2012**. DOI:[10.1155/2012/620962](https://doi.org/10.1155/2012/620962). |
| 1364 |
| Arowojolu MO, Fawole OI, Dosumu EB, Opeodu OI. A comparative study of the oral hygiene status of smokers and non-smokers in Ibadan, Oyo state. *Niger Med J* 2013; **54**: 240–3. |
| 1365 |
| Osunde OD, Adebola RA, Saheeb BD. A comparative study of the effect of suture-less and multiple suture techniques on inflammatory complications following third molar surgery. *International Journal of Oral and Maxillofacial Surgery* 2012; **41**: 1275–9. |
| 1366 |
| Emeka CI, Adeyemo WL, Ladeinde AL, Butali A. A comparative study of quality of life of families with children born with cleft lip and/or palate before and after surgical treatment. *J Korean Assoc Oral Maxillofac Surg* 2017; **43**: 247–55. |
| 1367 |
| Ibeachu PC, Didia BC, Arigbede AO. A Comparative Study of Palatal Rugae Patterns among Igbo and Ikwerre Ethnic Groups of Nigeria: A University of Port Harcourt Study. *Anat Res Int* 2014; **2014**: 123925. |
| 1368 |
| Anslem O, Eyituoyo O, Olabode OV, Ademola OA, Adesina AO. A comparative study of intermaxillary fixation screws and noncompression miniplates in the treatment of mandibular fractures: a prospective clinical study. *Oral and Maxillofacial Surgery* 2017; **21**: 233–40. |
| 1369 |
| Maharaj B, Coovadia Y, Vayej AC. A comparative study of amoxicillin, clindamycin and chlorhexidine in the prevention of post-extraction bacteraemia. *Cardiovasc J Afr* 2012; **23**: 491–4. |
| 1370 |
| Olurotimi AO, Gbotolorun OM, Ibikunle AA, Emeka CI, Arotiba GT, Akinwande JA. A comparative clinical evaluation of the effect of preoperative and postoperative antimicrobial therapy on postoperative sequelae after impacted mandibular third molar extraction. *J Oral Maxillofac Res* 2014; **5**: e2. |
| 1371 |
| Umeanuka OT, Saheeb BD, Chukwuneke FN, Uguru CC. A comparative analysis of the level of cortisol and the number of teeth extracted among patients undergoing routine dental extraction. *Niger J Clin Pract* 2016; **19**: 700–3. |
| 1372 |
| Koyio LN, van der Sanden WJ, van der Ven A, *et al.* A Community-based Oral Health Promotion Model for HIV Patients in Nairobi, East District in Kenya: a Study Protocol. *Journal of public health research* 2013; **2**: 22–8. |
| 1373 |
| Olatosi OO, Oyapero A, Boyede GO. A community survey on maternal perception about the initiation of dental home for infants in lagos, nigeria. *Pan African Medical Journal* 2021; **40**. DOI:[10.11604/pamj.2021.40.78.24441](https://doi.org/10.11604/pamj.2021.40.78.24441). |
| 1374 |
| Grobler SR, Majeed A, Hayward R, Rossouw RJ, Moola MH, van W Kotze TJ. A clinical study of the effectiveness of two different 10% carbamide peroxide bleaching products: a 6-month followup. *Int J Dent* 2011; **2011**: 167525. |
| 1375 |
| Hayward R, Osman Y, Grobler SR. A clinical study of the effectiveness of a light emitting diode system on tooth bleaching. *Open Dent J* 2012; **6**: 143–7. |
| 1376 |
| Fomete B, Osunde OD, Ogbeifun J, Agbara R, Ononiwu CN. A 10-year retrospective analysis of 64 cases of cystic lesions of the oral and maxillofacial region in a Nigerian tertiary hospital. *Oman Medical Journal* 2016; **31**: 434–8. |
| 1377 |
| Ajayi Deborah M, Abiodun-Solanke Iyabode MF, Gbadebo Shakeerah O. A 5-year retrospective study of rampant dental caries among adult patients in a Nigerian Teaching Hospital. *Indian Journal of Dental Research* 2015; **26**: 267–70. |
| 1378 |
| Otuyemi OD, Olaniyi EA. A 5-year retrospective evaluation of undergraduate dental research projects in a Nigerian University: Graduates’ perceptions of their learning experiences. *European Journal of Dental Education* 2020; **24**: 292–300. |
| 1379 |
| Ibikunle AA, Taiwo AO, Braimah RO. A 5-year audit of major maxillofacial surgeries at Usmanu Danfodiyo university teaching hospital, Nigeria. *BMC Health Services Research* 2018; **18**. DOI:[10.1186/s12913-018-3236-1](https://doi.org/10.1186/s12913-018-3236-1). |

# **Appendix Table 4. Taxonomy of oral health topics and subcategories of articles published in the WHO African region countries between 2011 and 2022.**

| **Topic** | **Subcategories** |
| --- | --- |
| 1. Dental caries | - Enamel caries  - Dentin caries  - Root surface caries  - Early childhood caries |
| 1. Periodontal diseases | - Gingivitis  - Periodontitis  - Aggressive periodontitis  - Chronic periodontitis  - Necrotizing periodontal diseases  - Periodontal abscess  - Acute herpetic gingivostomatitis |
| 1. Oral infections (excluding dental caries and periodontal diseases) | - Candidiasis  - Oral herpes  - Herpetic gingivostomatitis  - Dental abscess  - Ludwig's angina (Cellulitis of the floor of the mouth)  - Necrotizing fasciitis  - Noma (*Cancrum oris*) |
| 1. Oral lesions | - Aphthous ulcers  - Leukoplakia  - Erythroplakia  - Oral lichen planus  - Mucocele  - Pyogenic granuloma  - Oral fibroma  - Eosinophilic ulcer  - Geographic tongue  - Hairy tongue  - Parulis  - Ameloblastoma  - Odontogenic myxoma |
| 1. Malocclusions | - Overbite  - Underbite  - Crossbite  - Open bite  - Crowding  - Spacing  - Edge-to-edge bite  - Scissor bite |
| 1. Oral cancer | - Squamous cell carcinoma  - Adenocarcinoma  - Mucoepidermoid carcinoma  - Verrucous carcinoma  - Oropharyngeal carcinoma  - Minor salivary gland tumors |
| 1. Temporomandibular disorders | - TMJ pain  - TMJ dysfunction (TMD)  - TMJ clicking or popping  - TMJ arthritis  - Disc displacement (Internal derangement)  - Bruxism (Teeth grinding) |
| 1. Dental anomalies | - Impacted teeth  - Ectopic eruption  - Anodontia  - Hypodontia  - Supernumerary teeth  - Tooth dysplasia  - Amelogenesis imperfecta  - Dentinogenesis imperfecta  - Taurodontism  - Gemination  - Fusion  - Dilaceration  - Enamel pearls  - Dens invaginatus  - Turner's hypoplasia  - Dental Fluorosis |
| 1. Endodontic conditions | - Irreversible pulpitis  - Pulpal abscess  - Periapical abscess  - Apical periodontitis  - Pulpal necrosis  - Pulpal hyperemia (Reversible pulpitis)  - Root resorption (Internal and external)  - Periapical granuloma |
| 1. Dental trauma | - Tooth fractures  - Crown-root fractures  - Avulsed teeth  - Luxated teeth  - Root resorption  - Concussion  - Subluxation  - Dentoalveolar fractures |
| 1. Salivary gland disorders | - Sialadenitis  - Sialolithiasis (Salivary stones)  - Salivary gland tumors |
| 1. Halitosis | Halitosis |
| 1. Edentulism | - Complete tooth loss (Total edentulism)  - Shortened dental arch |
| 1. Craniofacial anomalies | - Cleft lip  - Cleft palate |
| 1. Maxillofacial trauma | - Facial fractures  - Soft tissue injuries  - Mandibular fractures  - Maxillary fractures  - Zygomatic fractures  - Orbital fractures  - Nasal fractures |
| 1. Oral Health Needs | - Oral health need assessment  - Workforce studies  - Policymaking |
| 1. Oral Health Systems | - Oral health services (primary, secondary, tertiary care)  - Oral health access  - Oral health interventions |
| 1. Oral Health Education | - Community outreach and promotion  - Preventive education  - Healthcare professionals education |
| 1. Oral Health Status | - Oral health situation (epidemiology)  - Burden of disease  - Oral health related quality of life |
| 1. Anthropometrics | - Cephalometrics  - Age estimation methods  - Palatal rugae analysis |
| 1. Psychological Factors and Behavioral Issues | - Dental anxiety  - Esthetic perception |

# **Appendix Table 5. Ranking of the top 20 journals with the highest number of publications in oral health research about the WHO African region countries between 2011 and 2022 (n=1,379).**

| **Journal title** | **Articles**  **n %** | |
| --- | --- | --- |
| BMC Oral Health 91 | | 6.61 |
| Nigerian Journal of Clinical Practice 52 | | 3.78 |
| Pan African Medical Journal 30 | | 2.18 |
| Journal of The West African College of Surg. 29 | | 2.11 |
| Nigerian Journal of Dental Research 27 | | 1.96 |
| African Health Sciences 24 | | 1.74 |
| PLoS ONE 24 | | 1.74 |
| Tropical Dental Journal 23 | | 1.67 |
| Annals of Medical and Health Science Res. 22 | | 1.60 |
| Annals of Ibadan Postgraduate Medicine 21 | | 1.53 |
| West African Journal of Medicine 20 | | 1.45 |
| International Dental Journal 19 | | 1.38 |
| International Journal of Dentistry 19 | | 1.38 |
| The Journal of Contemporary Dental Practice 19 | | 1.38 |
| East African Medical Journal 18 | | 1.31 |
| The Nigerian Postgraduate Medical Journal 18 | | 1.31 |
| Tanzania Journal of Health Research 16 | | 1.16 |
| Ghana Medical Journal 13 | | 0.94 |
| International Orthodontics 13 | | 0.94 |
| BMC Research Notes 12 | | 0.87 |
